# Epigenetic marking of sperm by post-translational modification of histones and protamines

**DOI:** 10.1186/1756-8935-7-2

**Published:** 2014-01-20

**Authors:** Andrea M Brunner, Paolo Nanni, Isabelle M Mansuy

**Affiliations:** 1 Department of Health Science and Technology of ETH Zürich, Neuroscience Centre Zürich, Brain Research Institute, Medical Faculty of the University of Zürich, Winterthurerstrasse 190, Zürich CH-8057, Switzerland; 2 Functional Genomics Centre Zürich, University of Zürich and ETH Zürich, Winterthurerstrasse 190, Zürich CH-8057, Switzerland; 3 Biomolecular Mass Spectrometry and Proteomics, Utrecht University, Padualaan 8, 3584 CH, Utrecht, The Netherlands

**Keywords:** Epigenetics, Mouse sperm, Histones, Protamines, Post-translational modifications, Mass spectrometry, Electron transfer dissociation, Intact protein analysis, Top-down, Proteoforms

## Abstract

**Background:**

The concept that individual traits can be acquired and transmitted by the germline through epigenetic mechanisms has gained recognition in the past years. However, epigenetic marks in sperm have not been are not well identified.

**Results:**

Using a novel proteomic approach that combines peptide-based bottom-up and intact protein top-down tandem mass spectrometry, we report the identification of epigenetic marks on histones and protamines in adult mouse sperm. We identified a total of 26 post-translational modifications (PTMs) on specific residues of the core histones H2B, H3 and H4, and the linker histone H1, four of which had not been described previously in any tissue or cell line. We also detected 11 novel PTMs on the protamines PRM1 and PRM2 and observed that they are present in specific combinations on individual protamines.

**Conclusions:**

Both histones and protamines carry multiple PTMs in the adult mouse sperm. On protamines, specific PTM combinations might form a ‘protamine code’ similar to the ‘histone code’. These findings suggest a potential role for PTMs on sperm histones and protamines in epigenetic signatures underlying transgenerational inheritance.

## Background

The epigenetic status of the genome in eukaryotes strongly influences chromatin structure and remodeling, and determines the level of gene regulation. Typically, the epigenetic profile of a cell is conferred by DNA methylation and post-translational modifications (PTMs) of histones H1, H2A, H2B, H3 and H4, which together form a code that controls gene expression. These epigenetic marks are specific to each gene, and are dynamically regulated during development and adulthood. They are also influenced by various factors throughout life, in particular by environmental conditions. In sperm cells, these marks are extremely important because they provide an identity to each cell and, as they can carry information from parent to offspring, may be involved in the maintenance and the inheritance of innate or acquired epigenetic signatures [1].

Sperm cells are highly specialized cells, produced by spermatogenesis, a process that involves extensive cellular, epigenetic and chromatin changes. Spermatogenesis starts with the replication and differentiation of spermatogonial stem cells into primary spermatocytes, which through genetic recombination during meiosis develop into haploid secondary spermatocytes [2]. In the haploid phase of spermatogenesis, round spermatids mature into spermatozoa. During this process, nucleosomes are disassembled upon histone H4 hyperacetylation and incorporation of non-canonical histone variants. Histones are widely replaced by highly basic proteins, first by transition proteins and subsequently by the two protamines PRM1 and PRM2 [3,4]. In contrast to PRM1, PRM2 associates with the DNA in a precursor form that is processed proteolytically to give rise to the mature PRM2 protamine with approximately 40% of the N-terminus cleaved off [5]. In human sperm, protamines have been shown to be phosphorylated at PRM1S9, PRM1S11, and PRM2S59. However, the function of this phosphorylation remains unknown. Further post-translational processing of PRM1 and PRM2 occurs during transit of the spermatozoa through the epididymis. Protamines form disulfide bonds and zinc bridges, both within individual proteins and among different proteins [6,7].

The association between DNA and protamines leads to substantial molecular remodeling and ultimately to 10-fold compaction of the male genome in toroidal nucleoprotamine structures. The association with protamines might facilitate the hydrodynamic shape of the sperm head and protect the paternal genome from physical and chemical damage, while the protamines themselves could play a role in epigenetic processes [8]. In mature spermatozoa, about 99% of histones are replaced by the protamines PRM1 and PRM2 in mice, and about 85% in humans.

Intriguingly, the genome-wide distribution of protamine- and nucleosome-associated DNA regions is not random, but nucleosomes retained in sperm are significantly enriched at loci important for embryogenesis, at specific promoters including those of miRNAs, and at imprinted genes. Furthermore, histone PTMs localize to specific developmental loci: H3K4me2/3 is enriched at certain developmental promoters; H3K4me3 localizes to HOX gene clusters, noncoding RNAs, and paternally expressed imprinted loci; and H3K27me3 is enriched at developmental promoters, which are repressed in early embryos [9,10]. Most recently, it was shown that throughout the genome, the retained nucleosomes are enriched at CpG-rich sequences that lack DNA methylation. The non-canonical histone H3.3 variant was shown to be abundant and trimethylated at K4 in these nucleosomes, while the canonical histones H3.1 and H3.2 were trimethylated at K27 [11]. Other non-canonical histone variants had been reported previously to be present in the retained nucleosomes of mature sperm: TH2B was observed in human, H2A-Bbd, H2AL1/L2, and H2BL1 in mammalian sperm [8]. Further, overall acetylation of histones H3 and H4, ubiquitination of H2A and H2B, and H3K9me3 had been described [12].

After fertilization, histones seem to remain associated with the paternal genome and to contribute to zygotic chromatin despite the extensive reorganization of the sperm chromatin [13]. In postfertilization, the sperm DNA is decondensed from its highly compacted and transcriptionally quiescent state, and expands to the inducible state found in the paternal pronucleus [14]. The paternal histones could therefore serve as a template for the incorporation of newly synthesized histones during replication in the zygote. Accordingly, the programmatic chromatin packaging in sperm could potentially deliver epigenetic information to the oocyte and the zygote postfertilization.

While histone PTMs have been described in developing male germ cells previously [15,16], little is known about the histone PTM status in mature sperm. Further, it is also not known whether protamines are subjected to PTMs in mouse sperm. Here, using a novel proteomic approach combining peptide-based bottom-up and intact protein top-down mass spectrometry (MS), we qualitatively characterize mouse sperm cells. These analyses reveal novel PTMs on histones and protamines, and are the first to show distinct combinations of protamine PTMs, reminiscent of the histone code.

## Results

**Figure 1:**
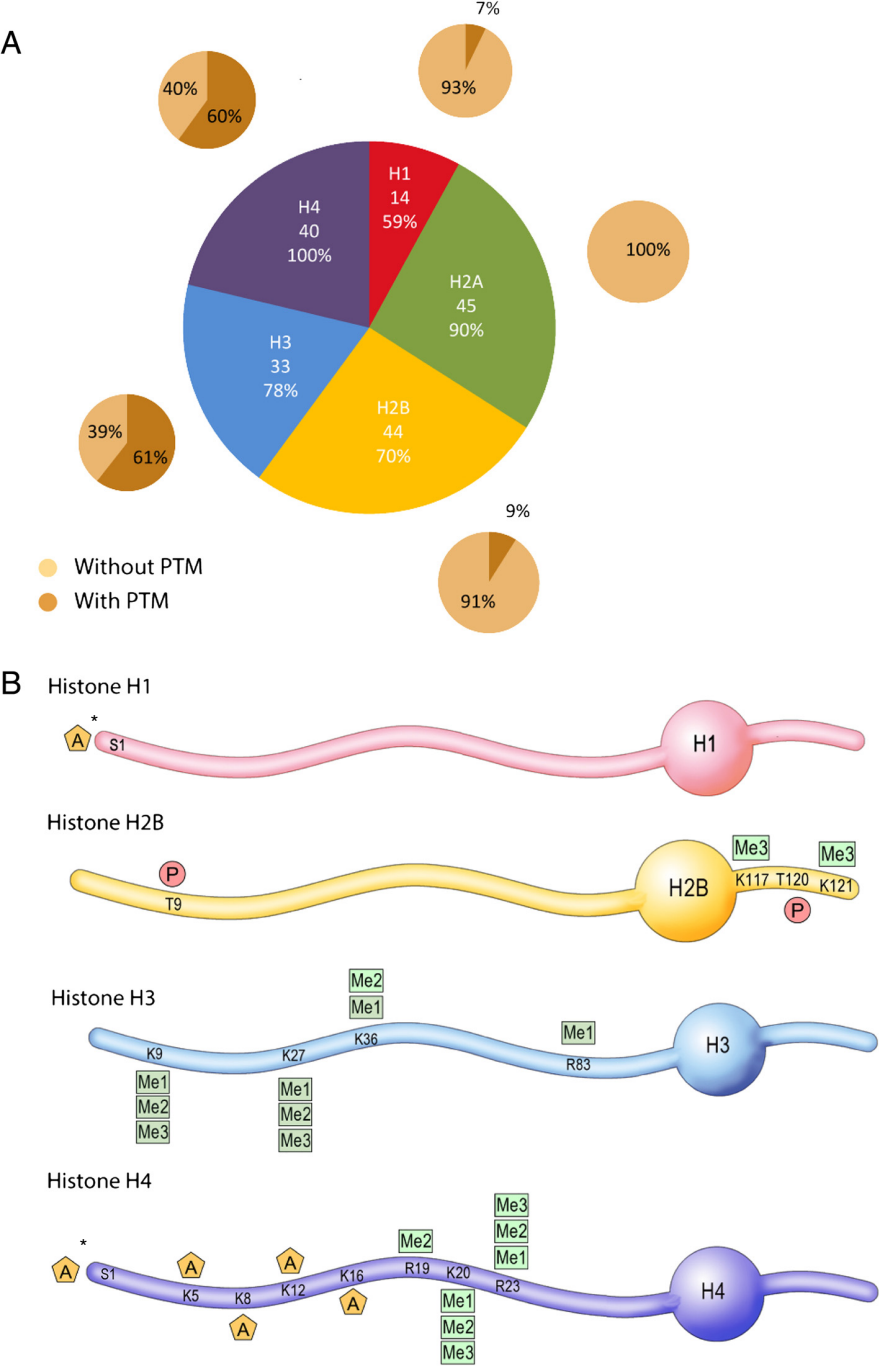
**Histone peptides and post-translational modifications (PTMs) detected in mouse sperm. A)** Proportion of detected histone peptides. The number of different peptides identified for each histone type, and sequence coverage (%) observed. The percentage of peptides with and without PTM is indicated. **B)** 27 PTMs identified on histones H1, H2B, H3 and H4. PTMs are indicated by A for acetylation, Me1, Me2 and Me3 for mono-, di- and trimethylation, and P for phosphorylation. Residues are numbered starting with the first residue after the cleaved methionine according to histone field standard. * indicates that the acetylation site could not be assigned to a single residue, (that is, N-terminus or serine acetylation).

To identify chromatin PTMs in mouse sperm, we used a peptide-based bottom-up MS/MS strategy. We developed a protocol for isolating sperm histone and protamine peptides. It consists of nuclear isolation from whole sperm cells, acid and high-salt extraction of basic nuclear proteins, digestion with various enzymes to optimize MS sequence coverage, and strong cation exchange chromatography (SCX) to enrich for acetylated and phosphorylated peptides. Peptides and PTMs were then identified using a high mass accuracy LTQ-Orbitrap XL mass spectrometer with a combination of electron transfer dissociation (ETD) and collision-induced dissociation (CID) for peptide fragmentation. The CID and ETD fragmentation spectra of all identified histone peptides with PTM are shown in Additional file 1: Figure S2. With this workflow, we identified all five histone types, and detected 176 different peptides from these histones, including 40 to 45 peptides from H2A, H2B and H4, 33 from H3 and 14 from H1, with a false discovery rate (FDR) of less than 5% (Figure 1A and [see Additional file 2: Table S1]). On these peptides, the extent of PTM varied greatly depending on the histone type, and ranged from over 60% for H3 and H4 peptides to 7% for H1 peptides and 0% for H2A peptides (Figure 1A). Overall, a total of 26 PTMs was detected on H1, H2B, H3 and H4, including lysine and arginine methylation, N-terminal and lysine acetylation and threonine phosphorylation (Figure 1B).

**Figure 2:**
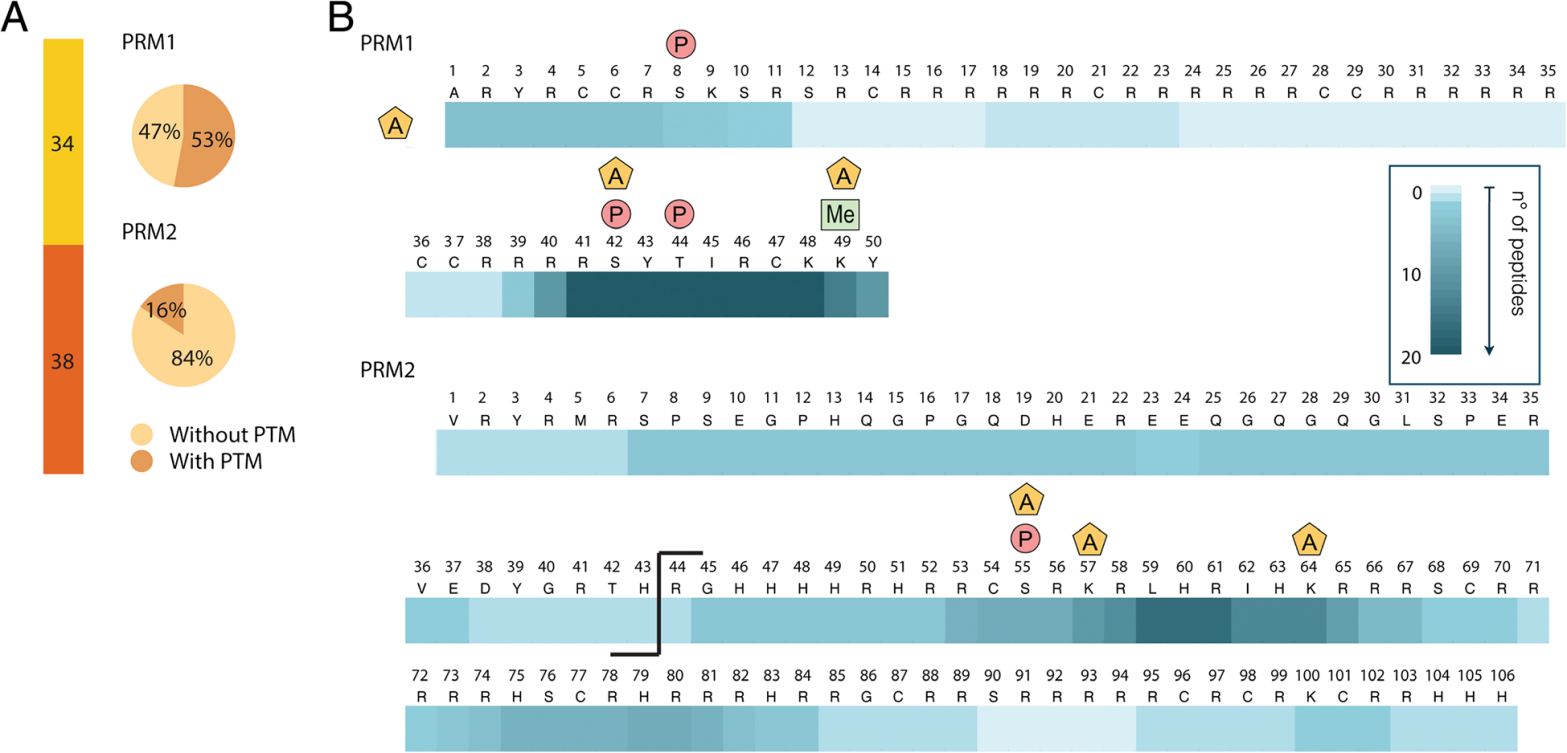
**Protamine peptides and post-translational modifications (PTMs) detected in mouse sperm. A)** Proportion of detected PRM1 and PRM2 peptides. Number of different peptides identified for each protamine, and percentage of peptides with and without PTM are indicated. **B)** A total of 11 PTMs newly identified on protamines PRM1 and PRM2. PTMs are indicated by A for acetylation, Me for methylation and P for phosphorylation. Residues are numbered starting with the first residue after the cleaved methionine according to histone field standard. The protamine sequence coverage is color coded and indicates the number of peptides that covered a given residue. The cleavage site of the PRM2 precursor at R44 is indicated in the sequence.

**Figure 3:**
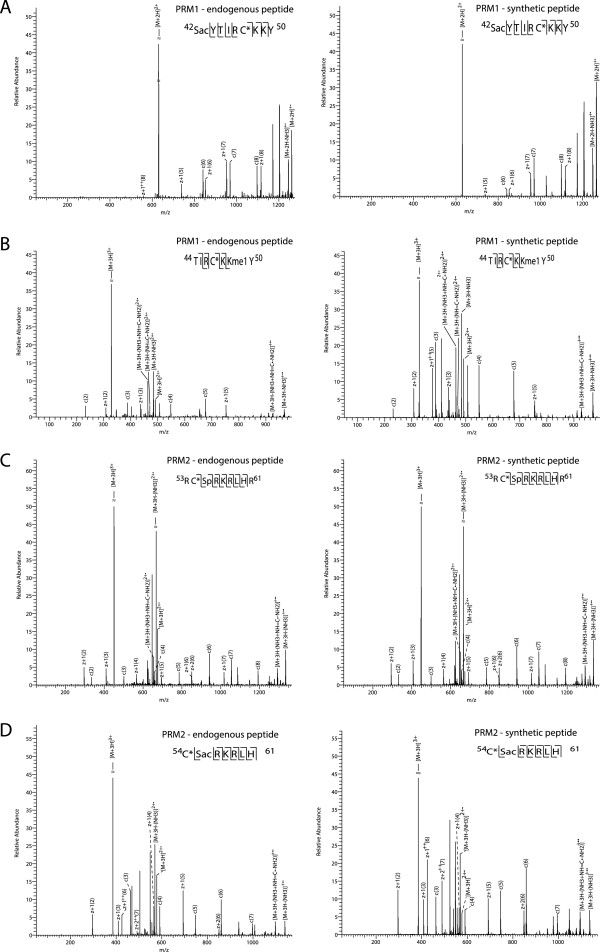
**Protamine post-translational modification (PTM) site validation by spectral comparison with synthetic peptides.** Mass spectra of identified endogenous protamine peptides with novel PTM sites and their synthetic counterparts. Major peaks are labeled in the spectra and the fragment ions indicated in the peptide sequence. **A)** A novel site of serine acetylation on residue S42 of PRM1. **B)** A novel site of lysine methylation on residue K49 of PRM2. **C)** A novel site of serine phosphorylation on residue S55 of PRM2. **D)** A novel site of serine acetylation on residue S55 of PRM2.

In addition to histone peptides, we also identified 34 peptides from protamine PRM1 and 38 from PRM2 (Figure 2A and [see Additional file 3: Table S2]). A total of 53% of PRM1 peptides and 16% of PRM2 peptides carried PTMs. In total, 11 novel PTMs could be identified (7 on PRM1 and 4 on PRM2), including 3 serine phosphorylation sites, 1 threonine phosphorylation site, 1 N-terminal acetylation, 3 lysine acetylation sites, 2 serine acetylation sites and 1 lysine mono-methylation site (Figure 2B). These novel sites were confirmed using synthetic peptides carrying the same PTMs. For all 11 protamine PTMs, comparing the spectrum of the synthetic peptides and the spectrum of the endogenous peptides revealed a high similarity, pointing to the validity of the newly detected PTMs on protamines (Figure 3 and [see Additional file 4: Figure S1]).

**Figure 4:**
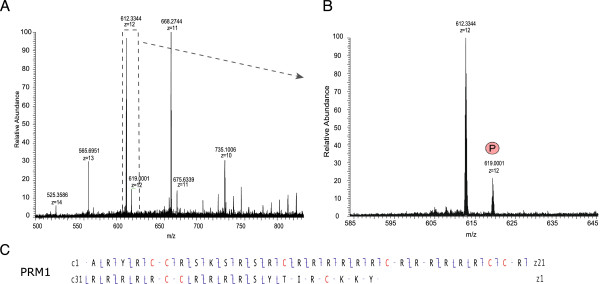
**PRM1 forms identified with a top-down strategy for intact protein analysis. A)** MS1 trace of the most abundant charge states of PRM1 (+10 to +14). **B)** MS1 trace of the 12+ charged unmodified PRM1 (no post-translational modification (PTM)), and PRM1 modified by phosphorylation (p). **C)** MS2 sequence coverage of unmodified PRM1. The sequence coverage is indicated and shows c ions from the right (**⌉**) and z ions from the left (**⌊**).

**Figure 5:**
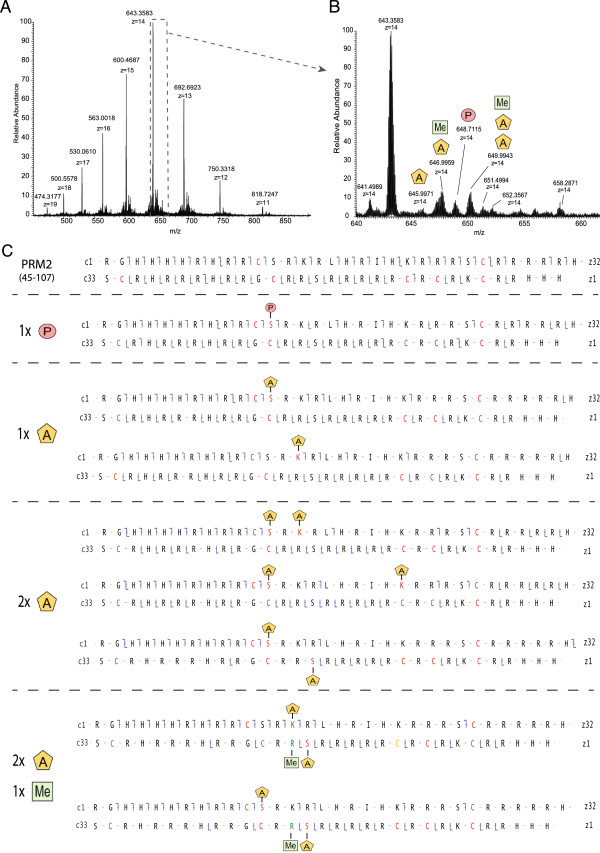
**PRM2 forms identified with a top-down strategy for intact protein analysis. A)** MS1 trace of the most abundant charge states of PRM2 (+11 to +19). **B)** MS1 trace of the 14+ charged unmodified PRM2 (no post-translational modification (PTM)), and PRM2 modified by acetylation (A), acetylation and methylation (A + Me), phosphorylation (P), and double acetylation and methylation (2A + Me). **C)** MS2 sequence coverage of PRM2 forms. The sequence coverage is indicated and shows c ions from the right (**⌉**) and z ions from the left (**⌊**).

In these analyses, the ensemble of PRM2 peptides spanned nearly the entire sequence, resulting in good PRM2 sequence coverage. The sequence coverage for PRM1, however, was limited, due to long stretches of arginine in the core region of this protamine (residues 13 to 41) that were difficult to sequence (Figure 2). To improve sequence coverage and identify PTMs in the unsequenced regions, we developed a top-down approach based on the analysis of intact nuclear sperm proteins by liquid chromatography coupled to an LTQ-Orbitrap Velos ETD. Using this approach, we could identify full-length PRM1 and PRM2 proteins. PRM1 was detected both as unmodified and phosphorylated (Figure 4 and [see Additional file 5: Figure S3]) while PRM2 was mainly present as a processed form with the first 44 amino acids cleaved off. Various modified forms of processed PRM2 were also detected including monomethylated, acetylated and phosphorylated forms, and various combinations of these PTMs such as one acetylation combined with one methylation site, two acetylation sites together, and two acetylation and one methylation sites (Figure 5B and C, and [see Additional file 3: Figure S3]). The PTMs could be assigned to specific PRM2 residues on the basis of specific fragment ions. Thus, PRM2 di-acetylation was on S55 and K57, S55 and K64, or S55 and S90, while di-acetylation and mono-methylation occurred on S55ac, S90ac and R88/89me1, or K57ac, S90ac and R88/89me1 (Figure 5C). These results reveal that multiple PTMs co-occur on individual protamines.

## Discussion

### Sperm cell specificity of novel histone post-translational modifications?

This study reports the identification of all five histone types in mature mouse sperm, and 26 PTMs on the four histone types H1, H2B, H3 and H4. It is the first study to investigate mouse sperm histones and their PTMs without the use of antibodies, but with tandem mass spectrometry-based proteomics. It allowed the identification of four novel PTMs (H2BT9p, H2BK117me3, H2BK121me3, and H3R83me1), which had not been described previously in any tissue or cell line. This suggests that the novel PTMs might be sperm-cell specific. This possibility is supported by the fact that H2BT9p could be specifically assigned to the testis-specific variant, TH2B, based on the presence of three H2B peptides unique for this variant [see Additional file 2: Table S1]. TH2B has recently been shown to replace canonical H2B during spermatogenesis, thereby directing histone to protamine transition [17]. At fertilization, TH2B is present in the male pronucleus and remains associated with embryonic cell chromatin. TH2B and its PTMs could therefore be involved in chromatin destabilization during early embryonic development, facilitating genome plasticity. An alternative explanation to why the novel PTMs were not reported previously could be that three of the four PTMs (H2BK117me3, H2BK121me3, and H3R83me1) occur in the core regions of histones H2B and H3, which are less well studied than their terminal tails.

### Functional roles of newly identified sperm histone post-translational modifications

In the case of sperm histones, only overall acetylation of histones H3 and H4, and ubiquitination of H2A and H2B, and H3K4me2/3, H3K9me3 and H3K27me3 have been reported previously [12]. Our data confirm the methylation of H3K9 and H3K27, but newly show that these residues can be mono-, di- or tri-methylated. Our data also reveal additional methylation sites on H3 (H3K36me1/2, H3R83me1). Moreover, H3K27 and H3K36 methylation frequently co-occur on the same peptide, consistent with previous reports [18]. Interestingly in our data, H3K36 methylation is only observed when H3K27 is also methylated, indicating potential crosstalk in H3 methylation (that is, that H3K27me contributes to the control of H3K36me). Regarding the functional role of these methylation sites, H3K9me2, H3K9me3 and H3K27me3 are known to be involved in transcriptional repression. In contrast, H3K36me3 is a transcriptional activator [19]. In sperm in particular, H3K27me3 was shown to mark developmental regulators [9], which are repressed in early embryos [10]. The specific roles of the mono- and di-methylated forms in sperm, and whether the PTMs are enriched at distinct genetic loci, remain to be investigated. It is interesting to note that while the core PTM H3R83me1 was only detected on the H3 variant H3.3, all N-terminal tail PTMs (H3K9me1/2/3, H3K27me1/2/3, and H3K36me1/2) and combinations thereof were identified on both canonical H3 (H3.1/H3.2) and the H3.3 variant [see Additional file 2: Table S1].

In addition to H3, we identified methylation sites on H4 (R19me2, K20me1/2/3, R23me1/2/3) and H2B (K117me3, K121me3). H4K20me is known to be implicated in chromatin compaction and transcriptional repression [19]. Recently, H4R19me and H4R23me, and in particular their combination, have been suggested to regulate the interaction of H4K20me with its binding proteins [20]. Correspondingly, we detected H4K20me1R23me1, HK29me1R23me2 and H4K20me2R23me1 peptides [see Additional file 2: Table S1]. The functional role of the identified H2B methylation sites remains to be elucidated, as methylation in general can be associated with both transcriptional repression and activation.

Our data also newly reveal multiple sites of modification including N-terminal or S1, K5, K8, K12 and K16 acetylation on H4, N-terminal or S1 acetylation on H1, and T9 and T120 phosphorylation on H2B. Both acetylation and phosphorylation are prominent activation mark on histones. Acetylation is known to neutralize the positive charge of the lysine residue, weakening the DNA-histone interaction. It is also involved in DNA repair, replication and condensation [21]. Similarly, phosphorylation creates a repellent force to the DNA, opening the chromatin and activating gene transcription [22]. The identification of PTMs associated with transcriptional activation is unexpected, as sperm cells are thought to be transcriptionally inert. However, these marks in paternal chromatin could contribute to the rapid activation of specific genes after fertilization. Alternatively, the presence of H4K8ac and H4K12ac could indicate centromeric heterochromatin [23]. N-terminal acetylation of H4 has been shown to regulate arginine methylation and chromatin silencing [24], while more generally, N-terminal protein acetylation is known to regulate protein-protein interactions, protein stability and protein localization [25].

The absence of PTMs on H2A corroborates a previous study reporting a low level of H2A modification site occupancy in somatic cells [26]. This suggests the possibility that H2A, if not regulated by PTM, may be controlled by exchange of variants such as H2AX, H2AL1, H2AL2 and H2BL1 previously reported [27,28]. Indeed, we detected multiple peptides unique for the testis-specific expressed gene 1 protein (H2A-Bbd), an atypical histone H2A variant associated with active transcription and mRNA processing [see Additional file 2: Table S1]. This variant was recently shown to be highly expressed in adult testis, mainly in spermatocytes [29].

Overall, this MS-based study provides a novel map of histone PTMs in adult mouse sperm, generating a global picture of possible modified histone forms. Further studies on the presence of sperm-specific histone PTMs and their combinatorial patterns in specific gene and promoter regions are needed to better understand the contribution of these histone forms to epigenetic inheritance. The importance of this endeavor is supported by two recent studies providing evidence that histone PTMs are involved in the transgenerational transmission of acquired traits [30,31].

### Evidence for a ‘protamine code’?

Our findings are the first to report the identification of multiple PTMs on protamines in mature mouse sperm (Figure 2B). They reveal that PRM1 is phosphorylated on S8 and PRM2 on S55, confirming human data (based on sequence homology) [32-34]. The data also identify unpredicted PTMs such as S42 and T44 phosphorylation on PRM1, N-terminal, S42 and K49 acetylation on PRM1, S55, K57, and K64 acetylation on PRM2, and K49 methylation on PRM1. The function of these PTMs is not known, but as histone PTMs, they may modulate protamine-DNA interactions [5]. On histones, PTMs are known to change protein structure and DNA binding by altering the electrostatic properties of histones. Acetylation, for instance, counteracts the positive charge of the epsilon-amino group of the lysine. Similarly, phosphorylation adds a negative charge to the histone. Both PTMs reduce the overall charge of histones and decrease their affinity for the negatively charged DNA, leading to chromatin opening and facilitation of recruitment of the transcriptional machinery [22]. The fact that protamines are modified by PTMs classically associated with transcriptional activation is, however, intriguing because these proteins are thought to ensure tight packaging of the DNA in sperm cells, and contribute to transcriptional silencing. This apparent contradiction can be reconciled by the possibility that PTMs on protamines may be involved in other regulatory functions. For instance, since PTMs in sperm are thought to provide paternal contribution to the epigenetic reprogramming of the zygote [13], protamine PTMs could control the incorporation of maternal histones after fertilization, with specific combinations of PTMs favoring the recruitment of selected histones. Interestingly in these combinations, while acetylation and methylation can co-occur on a given protamine, acetylation and phosphorylation appear to be exclusive. We did not detect proteins that were both acetylated and phosphorylated. Our data also suggest that the acetylation of residues PRM1 S42 and PRM2 S55, which can be modified by both PTMs, likely prevents their phosphorylation, consistent with a previous report [35]. Finally, the possibility that a protamine code plays a role in sperm cells and in the zygote shortly after fertilization is appealing, but needs to be investigated. It would be interesting for instance, to examine whether as is the case for histones, protamines with PTMs are enriched at specific promoters or loci important for early embryonic development [9].

### Analysis of protamine forms by high mass accuracy mass spectrometry and electron transfer dissociation fragmentation

An important parameter for the efficient detection and identification of protamines and PTMs in this study was the use of ETD fragmentation. ETD is a powerful method for the analysis of peptides with multiple basic residues such as protamines (rich in arginines), and in contrast to CID is not restricted to low charged peptides [36,37]. However, despite ETD, some arginine-rich regions, such as the PRM1 core (residues 13 to 41) that contains roughly 75% arginines, could not be sequenced using the bottom-up approach. We thus developed a top-down method for intact protein analysis to obtain sequence coverage of these regions, as well as to investigate the presence of specific modified protamine forms. This method allowed us to demonstrate the presence of both the full-length and processed PRM2 protein in mature mouse sperm. As expected, PRM2 was mainly present in its mature processed form with the first 44 amino acids cleaved off. This form originates by association of the PRM2 full-length precursor with the DNA and successive proteolytic cleavage. The cleaved mature PRM2 protein comprises the amino acid residues 45 to 107 and has been shown to be the predominant form in the mature sperm head [5]. Furthermore, the four PTMs identified with the peptide-based approach (that is, S55, K57 and K64 acetylation, and S55 phosphorylation) could also be confirmed.

## Conclusions

The presented study introduces a novel proteomic workflow for an aspect of epigenetic marking that had remained unexplored to date: the characterization of chromatin PTMs in adult mouse sperm. The workflow allowed the identification of novel histone PTMs in the male germline, and, for the first time, of PTMs on protamines, including serine, lysine and N-terminal protein acetylation, serine and threonine phosphorylation, and lysine methylation. Only three phosphorylation sites had been described in human sperm before, but no PTM on mouse protamines was known. Furthermore, combinations of PTMs on individual protamines were identified, providing an unexpected picture of the epigenetic landscape in sperm chromatin, These results suggest the existence of a ‘protamine code’ in addition to the ‘histone code’ in sperm, the role of which in transgenerational epigenetic inheritance remains to be investigated.

## Methods

### C57BL/6 mice

Mature sperm was collected from the caudal epididymis of adult 3- to 12-month-old C57BL/6 males as previously described [38]. Mice were housed under a reversed light cycle (dark phase, 7:00 to 19:00) in standard conditions. All experiments were ethically approved by the Swiss Cantonal Veterinary Office under license No. 55–2012, Title ‘Study of the impact of early trauma on behavior across generations in the mouse’.

### Isolation of nuclear proteins from mouse sperm

Histones and protamines were purified from adult sperm as previously described for brain histones [26]. In brief, sperm cells from the sperm of 5 to 15 mice per sample were homogenized in lysis buffer (Sigma Nuclei Pure isolation kit, Sigma-Aldrich, Buchs, Switzerland) with 1 × protease inhibitor cocktail and phosphatase inhibitor cocktail I and II (Sigma-Aldrich). Nuclei were isolated by sucrose gradient centrifugation. Two volumes of 1.8 M sucrose were added to the homogenates and the mixture was layered on top of one volume of 1.8 M sucrose. Nuclei were then pelleted by centrifugation at 30,000 g for 45 min, and snap frozen at −80˚C until analyzed. To separate histones from other chromatin components, acid and high-salt extraction was performed as described previously [39]. For acid extraction, isolated nuclei were resuspended in 0.2 M sulphuric acid (H_2_SO_4_) and incubated for >2 h at 4˚C with end-over-end rotation. For high salt extraction, isolated nuclei were lysed by re-suspension in 3 mM EDTA, 0.2 mM EGTA and incubated at 4°C for 30 min with end-over-end rotation. After centrifugation at 6,500 g for 5 min, the nucleoplasm-containing supernatant was removed and snap frozen at −80˚C until analyzed. The chromatin pellet containing DNA, histones and protamines was re-suspended in solubilization buffer containing 50 mM Tris-Cl pH 8.0, 2.5 M NaCl, 0.05% NP40, and incubated for 30 min at 4°C with end-over-end rotation. After centrifugation at 16,000 g for 10 min, both acid- and salt-extracted histones and protamines were precipitated with trichloroacetic acid (TCA) followed by a 30-min incubation on ice. After centrifugation at 16,000 g for 10 min, the pellet containing histones and protamines was washed twice with ice-cold acetone and centrifuged a second time.

### Reduction of disulphide bridges and alkylation of cysteines

Histones and protamines were resuspended in an appropriate buffer for subsequent enzymatic digestion (see below) or in 100 μl 25 mM NH_4_HCO_3_, pH 8 for intact protein analysis. Disulphide bridges were reduced by incubation with 10 mM dithiothreitol (DTT) for 45 minutes at 50°C, and cysteines alkylated by incubation with 50 mM iodoacetamide (IAA) for 1 hr at room temperature in the dark. The reaction was blocked with 50 mM DTT. Samples for intact protein analyses were desalted with ZipTip C18 columns (Millipore, Billerica, MA, USA) and lyophilized dry prior to MS analysis. Samples for peptide-based analyses were enzymatically digested (see below).

### In-solution digestion of histone proteins

Proteins were digested into peptides with trypsin (Promega, Madison, WI, USA) in 50 mM ammonium bicarbonate, pH 8.0 at 37˚C for 2 h (1:200 enzyme:substrate), with Glu-C (Roche Applied Science, Penzberg, Germany) in 25 mM ammonium carbonate, pH 7.8, at 24˚C for 18 h (1:20 enzyme:substrate), and with AspN (Roche Applied Science) and chymotrypsin (Roche Applied Science) in 100 mM Tris–HCl, 10 mM CaCl_2_, pH 7.8, at 25˚C for 25 h (1:200 enzyme:substrate), as described previously [26]. Enzymatic digests were stopped by adding 10% trifluoroacetic acid (TFA) to a final pH <3. Peptides were then desalted with ZipTip C18 columns (Millipore) and lyophilized dry prior to analysis by ETD/CID-MS/MS.

### Acetylated and phospho-peptide enrichment

To enrich for acetylated and phospho-peptides, digested peptides were fractionated by SCX [40]. Peptides were loaded onto a 12 μm, 300 Å pore size ZipTip SCX column (Millipore) and eluted in four fractions with increasing KCl concentration (50 mM, 150 mM and 300 mM KCl in 0.1% TFA, and 5%NH_4_OH).

### Synthetic peptides

Lyophilized synthetic peptides were ordered from 21st Century Biochemicals, Boston, MA, USA, resolubilized in 50% acetonitrile (ACN), 0.1% formic acid (FA) and analyzed by direct infusion.

### Peptide-based bottom-up collision induced dissociation/electron transfer dissociation mass spectrometry/mass spectrometry analysis

Peptide samples were analyzed on an LTQ-Orbitrap XL-ETD mass spectrometer (Thermo Scientific, Germany) coupled to an Eksigent Nano-HPLC system (Eksigent, AB Science, Redwood City, CA, USA). Solvent composition at the two channel was 0.2% formic acid, 1% ACN for channel A and 0.2% formic acid, 80% ACN for channel B. Peptides were resolubilized in 3% ACN and 0.2% formic acid and loaded on a 10 cm fused silica column packed with 3 μm 200 Å pore size C18 resin. Peptides were eluted with a flow rate of 200 nl/min by an ACN gradient of 5-30% ACN over 35 min and 30 to 80% ACN over the subsequent 13 min. Full-scan mass spectra (m/z 300 to 2000) were acquired in the Orbitrap with a resolution of 60,000 at 400 m/z, after accumulation to a target value of 2e5. Six sequential CID and ETD MS/MS scans were acquired in the ion trap on the three most intense signals above a threshold of 500. The AGC target value for ion trap MSn scans was set to 1e4. CID was performed using a normalized collision energy of 35 and activation time of 30 ms. The ETD reaction time was 120 ms and isolation width was 2 m/z. The ETD anion target value was set at 1e6 and the activation time at 100 ms. Supplementary activation was employed and charge state dependent ETD time enabled. For all experiments, the precursor masses already selected for MS/MS were excluded for further selection for 30 s. The exclusion window was set to 20 ppm and the size of the exclusion list was set to 500. Samples were acquired using internal lock mass calibration set on m/z 429.0877 and 445.1200 m/z. The synthetic peptides were analyzed by direct infusion on an LTQ-Orbitrap XL-ETD. For every peptide, fragmentation was performed by CID and ETD, and spectra acquired in both the linear ion trap and the FT-Orbitrap. Twenty scans were collected for every acquisition. For CID/ETD fragmentation and for the acquisition of ion trap MS/MS spectra the same parameters used for LC-MS analysis were applied. For FT MS/MS spectra an AGC target value of 5E5 and an injection time of 200 ms were set.

### Peptide and post-translational modification identification

Mascot generic format (mgfs) files were generated from MS and MS/MS raw data. Mgfs were searched against a mouse protein database from the European Bioinformatics Institute (EBI, 48,564 sequences) using Mascot version 2.3 (Matrix Science, London, UK) with a peptide mass tolerance of 6 ppm and a fragment mass tolerance of 0.6 Da. The following PTMs were included in the searches: carbamidomethylation (C, fixed, 57.021464 Da), phosphorylation (S, T, and Y, variable, 79.966331 Da), acetylation (protein N-term, S, T, Y and K, variable, 42.010565 Da), mono-, di- and tri-methylation (R and K, variable, 14.015650 Da, 28.031300 Da and 42.046950 Da), and oxidation (M, variable, 15.994915 Da). Only peptides with the Mascot parameter rank 1 were accepted. In the case of histone peptides, a peptide expect cut-off of 0.05 and an FDR <5% was applied, and all PTMs identified by only one spectrum were discarded. For spectra of all peptides with PTM(s), confident PTM site placement was based on the Mascot site analysis probability [41] and manual validation. When alternative site localizations were possible for a given PTM, the ambiguous sites were indicated by parentheses. All identified peptides with PTMs are listed in Additional file 2: Tables S1 and Additional file 3: Table S2. Residues are numbered starting with the first residue after the cleaved methionine according to histone field standard.

### Intact protein top-down electron transfer tandem mass spectrometry analysis

Protein samples were analyzed on an LTQ-Orbitrap Velos ETD mass spectrometer (Thermo Scientific, Germany) coupled to an Eksigent Nano-HPLC system (Eksigent). Lyophilized intact proteins were resolubilized in 0.1% TFA and loaded on a 10 cm fused silica column packed with 3 μm 200 Å pore size C18 resin. Peptides were eluted via an ACN gradient of 1 to 55% ACN over 36 min in a buffer containing 0.2% FA at flow rate of 250 nl/min. Both full scan MS and MS/MS spectra were acquired in the Orbitrap analyzer, at a resolution of 60,000 for MS1 and 30,000 for MS2 scans. The AGC target values were set to 1E6 for MS1 and 1E5 for MS2. The maximum injection time was 250 ms for MS1 and 200 ms for MS2 scans. Fragmentation of the three most intense ions above a threshold of 5,000 was performed by ETD with an activation time of 15 ms. In order to improve the efficiency of ETD fragmentation, the precursor ions were isolated in a 10 Da m/z window. For each fragmentation event, supplementary activation was employed and 5 μscans were summed.

### Protein and proteoform identification

Spectra were deconvoluted with Mascot Distiller v 2.3.2. Protein and PTM identification was performed with Mascot version 2.3 and ProSight PTM 2.0 [42]. Mascot searches were performed against a protein database containing histones, protamines and their processed forms, and E. coli protein sequences. Peptide and fragment mass tolerance were set to 2 Da to account for incorrect isotope picking. The same PTMs as in the peptide-based approach were included in the searches: carbamidomethylation (C, fixed), phosphorylation (S, T, and Y, variable), acetylation (protein N-term, S, T, Y and K, variable), mono-, di- and tri-methylation (R and K, variable), and oxidation (M, variable). In ProSight PTM 2.0, the intact and fragment ion masses of selected scans were searched using the single protein search. PTM sites were manually placed and scored using sequence gazer.

## Abbreviations

Ac: acetylation; ACN: acetonitrile; CID: collision induced dissociation; Da: Dalton; ETD: electron transfer dissociation; FA: formic acid; FDR: false discovery rate; H2SO4: sulphuric acid; IAA: iodoacetamide; me1/me2/me3: mono-/di-/tri-methylation; mgf: Mascot generic format; MS: mass spectrometry; p: phosphorylation; PTM: post-translational modification; SCX: strong cation exchange; TFA: trifluoroacetic acid.

## Competing interests

The authors declare that they have no competing interests.

## Authors’ contributions

AMB conceived the project, planned and performed experiments, did data analysis and wrote the manuscript. AMB and PN planned and performed MS analyses. PN and IMM critically read the manuscript. IMM directed and supervised experiments, and contributed reagents, materials and analysis tools. All authors read and approve the final manuscript.

## Supplementary Material

Additional file 1: Figure S2 MS2 spectra of all modified histone peptides obtained from histone digests across all experiments. The Mascot peptide view, fragment ion table and the annotated peptide sequence with post-translational modification (PTM) are shown. Click here for file

Additional file 2: Table S1 List of all histone peptides derived from mouse sperm found in the peptide-based bottom-up experiments. In the peptide sequence the site/s of N-terminal acetylation are designated by ‘ac-’, before the modified residue, acetylation by ‘ac’, phosphorylation by ‘p’, and mono-/di-/tri-methylation by ‘me1, me2 or me3’ respectively. Percentages from the Mascot site analysis indicate the Mascot Delta score as a post-translational modification (PTM) site probability when alternative site localizations are possible for given PTM(s). For four peptides, the PTM is in parenthesis because the PTM site could not be assigned to a single residue. Click here for file

Additional file 3: Table S2 List of all protamine peptides derived from mouse sperm found in the peptide-based bottom-up experiments. In the peptide sequence the site/s of N-terminal acetylation are designated by ‘ac-’, before the modified residue, acetylation by ‘ac’, mono-methylation by ‘me1’, and phosphorylation by ‘p’. Click here for file

Additional file 4: Figure S1 Protamine post-translational modification (PTM) site validation by spectral comparison with synthetic peptides. Mass spectra of identified endogenous protamine peptides with novel PTM sites and their synthetic counterparts. Major peaks are labeled in the spectra and the fragment ions indicated in the peptide sequence. A) A novel site of acetylation at the N-terminus of PRM1. B) A novel site of serine phosphorylation on residue S8 of PRM1. C) A novel site of serine phosphorylation on residue S42 of PRM1. D) A novel site of threonine phosphorylation on residue T44 of PRM1. E) A novel site of lysine acetylation on residue K49 of PRM1. F) A novel site of lysine acetylation on residue K57 of PRM2. G) A novel site of lysine acetylation on residue K64 of PRM2. Click here for file

Additional file 5: Figure S3 MS2 spectra of intact PRM1 and PRM2 forms. Click here for file
