# Supplementary material for: Epigenetic marking of sperm by post-translational modification of histones and protamines
Source: Epigenetics Chromatin. 2014 Jan 20;7:2. doi: 10.1186/1756-8935-7-2 (PMC3904194; doi:10.1186/1756-8935-7-2)
Supplement: Additional file 1: Figure S2 — MS2 spectra of all modified histone peptides obtained from histone digests across all experiments. The Mascot peptide view, fragment ion table and the annotated peptide sequence with post-translational modification (PTM) are shown. [file 1756-8935-7-2-S1.pdf]

Supplementary Figure 2, Brunner et al.

MS/MS Fragmentation of SETAPAAPAAPAPAEKTPVKKKAR
Found in sp|P43274|H14_MOUSE in fgcz_10090, Histone H1.4 OS=Mus musculus GN=Hist1h1e

Match to Query 3848: 2428.340216 from(608.092330,4+) intensity(257960.0000) scans(3555) rtinseconds(2051.5263) index(2238)
Title: 2239: Scan 3555 (rt=34.1921) [s:\p749\Proteomics\ORBI_2\andreamizzi_20110517_sperm_trypsin_GluC_ETD_CID\20110517_01_NaCl_1.RAW]
Data file 20110517_01_NaCl_1.mgf

Click mouse within plot area to zoom in by factor of two about that point
Or, Plot from 200 to 2000 Da Full range
Label all possible matches Label matches used for scoring
Show Y-axis

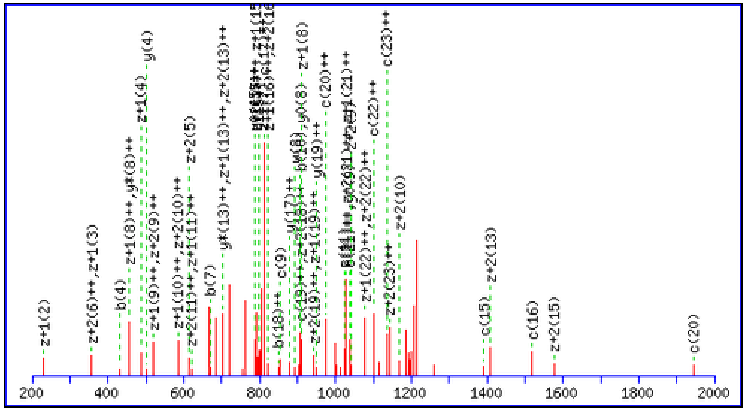

#	b	b^{++}	b^{*}	b^{*++}	b^{0}	b^{0++}	c	c^{++}	Seq.	w	w'	y	y^{++}	y^{*}	y^{*++}	y^{0}	y^{0++}	z+1	$z+1^{++}$	z+2	$z+2^{++}$	#
1	130.0499	65.5286			112.0393	56.5233	147.0764	74.0418	S													24
2	259.0925	130.0499			241.0819	121.0446	276.1190	138.5631	E	2225.2765		2300.3085	1150.6579	2283.2819	1142.1446	2282.2979	1141.6526	2284.2898	1142.6485	2285.2976	1143.1524	23
3	360.1401	180.5737			342.1296	171.5684	377.1667	189.0870	T	2138.2444	2140.2237	2171.2659	1086.1366	2154.2394	1077.6233	2153.2553	1077.1313	2155.2472	1078.1272	2156.2550	1078.6311	22
4	431.1773	216.0923			413.1667	207.0870	448.2038	224.6055	A			2070.2182	1035.6127	2053.1917	1027.0995	2052.2077	1026.6075	2054.1995	1027.6034	2055.2073	1028.1073	21
5	528.2300	264.6186			510.2195	255.6134	545.2566	273.1319	P	1956.1389		1999.1811	1000.0942	1982.1546	991.5809	1981.1705	991.0889	1983.1624	992.0848	1984.1702	992.5887	20
6	599.2671	300.1372			581.2566	291.1319	616.2937	308.6505	A			1902.1283	951.5678	1885.1018	943.0545	1884.1178	942.5625	1886.1096	943.5584	1887.1174	944.0624	19
7	670.3042	335.6558			652.2937	326.6505	687.3308	344.1690	A			1831.0912	916.0493	1814.0647	907.5360	1813.0807	907.0440	1815.0725	908.0399	1816.0803	908.5438	18
8	767.3570	384.1821			749.3464	375.1769	784.3836	392.6954	P	1717.0119		1760.0541	880.5307	1743.0276	872.0174	1742.0436	871.5254	1744.0354	872.5213	1745.0432	873.0252	17
9	838.3941	419.7007			820.3836	410.6954	855.4207	428.2140	A			1663.0014	832.0043	1645.9748	823.4910	1644.9908	822.9990	1646.9826	823.9950	1647.9905	824.4989	16
10	909.4312	455.2193			891.4207	446.2140	926.4578	463.7325	A			1591.9642	796.4858	1574.9377	787.9725	1573.9537	787.4805	1575.9455	788.4764	1576.9533	788.9803	15
11	1006.4840	503.7456			988.4734	494.7404	1023.5106	512.2589	P	1477.8849		1520.9271	760.9672	1503.9006	752.4539	1502.9166	751.9619	1504.9084	752.9578	1505.9162	753.4618	14
12	1077.5211	539.2642			1059.5106	530.2589	1094.5477	547.7775	A			1423.8744	712.4408	1406.8478	703.9275	1405.8638	703.4355	1407.8556	704.4315	1408.8635	704.9354	13
13	1174.5739	587.7906			1156.5633	578.7853	1191.6004	596.3039	P	1309.7950		1352.8372	676.9223	1335.8107	668.4090	1334.8267	667.9170	1336.8185	668.9129	1337.8263	669.4168	12
14	1245.6110	623.3091			1227.6004	614.3039	1262.6375	631.8224	A			1255.7845	628.3959	1238.7579	619.8826	1237.7739	619.3906	1239.7658	620.3865	1240.7736	620.8904	11
15	1374.6536	687.8304			1356.6430	678.8251	1391.6801	696.3437	E	1109.7153		1184.7474	592.8773	1167.7208	584.3640	1166.7368	583.8720	1168.7286	584.8680	1169.7365	585.3719	10
16	1502.7486	751.8779	1485.7220	743.3646	1484.7380	742.8726	1519.7751	760.3912	K	981.6204		1055.7048	528.3560	1038.6782	519.8428	1037.6942	519.3507	1039.6861	520.3467	1040.6939	520.8506	9
17	1603.7962	802.4018	1586.7697	793.8885	1585.7857	793.3965	1620.8228	810.9150	T	894.5883	896.5676	927.6098	464.3085	910.5833	455.7953	909.5992	455.3033	911.5911	456.2992	912.5989	456.8031	8
18	1700.8490	850.9281	1683.8224	842.4149	1682.8384	841.9229	1717.8755	859.4414	P	783.5199		826.5621	413.7847	809.5356	405.2714			810.5434	405.7753	811.5512	406.2793	7
19	1799.9174	900.4623	1782.8909	891.9491	1781.9068	891.4571	1816.9440	908.9756	V	698.4672		729.5094	365.2583	712.4828	356.7450			713.4906	357.2490	714.4985	357.7529	6
20	1928.0124	964.5098	1910.9858	955.9965	1910.0018	955.5045	1945.0389	973.0231	K	556.3566		630.4410	315.7241	613.4144	307.2108			614.4222	307.7148	615.4301	308.2187	5
21	2056.1073	1028.5573	2039.0808	1020.0440	2038.0968	1019.5520	2073.1339	1037.0706	K	428.2616		502.3460	251.6766	485.3194	243.1634			486.3273	243.6673	487.3351	244.1712	4
22	2184.2023	1092.6048	2167.1757	1084.0915	2166.1917	1083.5995	2201.2288	1101.1181	K	300.1666		374.2510	187.6292	357.2245	179.1159			358.2323	179.6198	359.2401	180.1237	3
23	2255.2394	1128.1233	2238.2129	1119.6101	2237.2288	1119.1181	2272.2660	1136.6366	A			246.1561	123.5817	229.1295	115.0684			230.1373	115.5723	231.1452	116.0762	2
24									R	73.0284		175.1190	88.0631	158.0924	79.5498			159.1002	80.0538	160.1081	80.5577	1

Monoisotopic mass of neutral peptide Mr(calc): 2428.3437
Fixed modifications: Carbamidomethyl (C) (apply to specified residues or termini only)
Variable modifications:
S1 : Acetyl_STY (STY)
Ions Score: 70 Expect: 2.7e-05
Matches : 58/387 fragment ions using 61 most intense peaks (help)

Mascot Search Results: Peptide View

(ac-S)ETAPAAPAAPAPAEKTPVKKKAR

MS/MS Fragmentation of **LLLPGELAKHAVSEGTKAVTK**
Found in **sp|P70696|H2B1A_MOUSE** in **fgcz_10090**, Histone H2B type 1-A OS=Mus musculus GN=Hist1h2ba

Match to Query 3867: 2325.306220 from(466.068520,5+) intensity(69789.6330) scans(5938) rtinseconds(3109.7809) index(4186)
Title: 4187: Scan 5938 (rt=51.8297) [s:\p749\Proteomics\ORBI_2\andreamizzi_20110517_sperm_trypsin_GluC_ETD_CID\20110517_02_NaCl_2.RAW]
Data file 20110517_02_NaCl_2.mgf

Click mouse within plot area to zoom in by factor of two about that point
Or, Plot from 100 to 1700 Da Full range
Label all possible matches ○ Label matches used for scoring ◉
Show Y-axis ☐

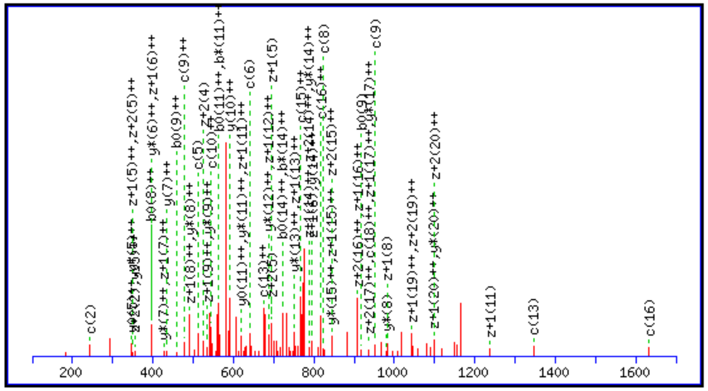

#	b	b^{++}	b^{*}	b^{*++}	b^{0}	b^{0++}	c	c^{++}	Seq.	w	w'	y	y^{++}	y^{*}	y^{*++}	y^{0}	y^{0++}	z+1	$z+1^{++}$	z+2	$z+2^{++}$	#
1	114.0913	57.5493					131.1179	66.0626	L													21
2	227.1754	114.0913					244.2020	122.6046	L	2154.1570		2213.2305	1107.1189	2196.2039	1098.6056	2195.2199	1098.1136	2197.2118	1099.1095	2198.2196	1099.6134	20
3	340.2595	170.6334					357.2860	179.1466	L	2041.0729		2100.1464	1050.5769	2083.1199	1042.0636	2082.1359	1041.5716	2084.1277	1042.5675	2085.1355	1043.0714	19
4	437.3122	219.1598					454.3388	227.6730	P	1944.0202		1987.0624	994.0348	1970.0358	985.5215	1969.0518	985.0295	1971.0436	986.0255	1972.0515	986.5294	18
5	494.3337	247.6705					511.3602	256.1838	G			1890.0096	945.5084	1872.9831	936.9952	1871.9990	936.5032	1873.9909	937.4991	1874.9987	938.0030	17
6	623.3763	312.1918			605.3657	303.1865	640.4028	320.7051	E	1757.9561		1832.9881	916.9977	1815.9616	908.4844	1814.9776	907.9924	1816.9694	908.9883	1817.9772	909.4923	16
7	736.4604	368.7338			718.4498	359.7285	753.4869	377.2471	L	1644.8721		1703.9456	852.4764	1686.9190	843.9631	1685.9350	843.4711	1687.9268	844.4671	1688.9347	844.9710	15
8	807.4975	404.2524			789.4869	395.2471	824.5240	412.7656	A			1590.8615	795.9344	1573.8349	787.4211	1572.8509	786.9291	1574.8428	787.9250	1575.8506	788.4289	14
9	935.5924	468.2999	918.5659	459.7866	917.5819	459.2946	952.6190	476.8131	K	1445.7400		1519.8244	760.4158	1502.7978	751.9025	1501.8138	751.4105	1503.8056	752.4065	1504.8135	752.9104	13
10	1072.6513	536.8293	1055.6248	528.3160	1054.6408	527.8240	1089.6779	545.3426	H			1391.7294	696.3683	1374.7029	687.8551	1373.7188	687.3631	1375.7107	688.3590	1376.7185	688.8629	12
11	1143.6885	572.3479	1126.6619	563.8346	1125.6779	563.3426	1160.7150	580.8611	A			1254.6705	627.8389	1237.6439	619.3256	1236.6599	618.8336	1238.6518	619.8295	1239.6596	620.3334	11
12	1242.7569	621.8821	1225.7303	613.3688	1224.7463	612.8768	1259.7834	630.3953	V	1152.5912		1183.6334	592.3203	1166.6068	583.8071	1165.6228	583.3150	1167.6147	584.3110	1168.6225	584.8149	10
13	1329.7889	665.3981	1312.7623	656.8848	1311.7783	656.3928	1346.8154	673.9114	S	1051.5435		1084.5650	542.7861	1067.5384	534.2728	1066.5544	533.7808	1068.5462	534.7768	1069.5541	535.2807	9
14	1458.8315	729.9194	1441.8049	721.4061	1440.8209	720.9141	1475.8580	738.4327	E	922.5009		997.5329	499.2701	980.5064	490.7568	979.5224	490.2648	981.5142	491.2607	982.5220	491.7647	8
15	1515.8530	758.4301	1498.8264	749.9168	1497.8424	749.4248	1532.8795	766.9434	G			868.4903	434.7488	851.4638	426.2355	850.4798	425.7435	852.4716	426.7395	853.4794	427.2434	7
16	1616.9006	808.9540	1599.8741	800.4407	1598.8901	799.9487	1633.9272	817.4672	T	778.4474	780.4267	811.4689	406.2381	794.4423	397.7248	793.4583	397.2328	795.4502	398.2287	796.4580	398.7326	6
17	1787.0425	894.0249	1770.0160	885.5116	1769.0320	885.0196	1804.0691	902.5382	K	594.2899		710.4212	355.7142	693.3947	347.2010	692.4106	346.7090	694.4025	347.7049	695.4103	348.2088	5
18	1858.0797	929.5435	1841.0531	921.0302	1840.0691	920.5382	1875.1062	938.0567	A			540.2793	270.6433	523.2527	262.1300	522.2687	261.6380	524.2606	262.6339	525.2684	263.1378	4
19	1957.1481	979.0777	1940.1215	970.5644	1939.1375	970.0724	1974.1746	987.5910	V	438.2000		469.2422	235.1247	452.2156	226.6115	451.2316	226.1194	453.2235	227.1154	454.2313	227.6193	3
20	2138.1621	1069.5847	2121.1355	1061.0714	2120.1515	1060.5794	2155.1886	1078.0980	T	257.1860	259.1652	370.1738	185.5905	353.1472	177.0772	352.1632	176.5852	354.1550	177.5812	355.1629	178.0851	2
21									K	73.0284		189.1598	95.0835	172.1332	86.5702			173.1410	87.0742	174.1489	87.5781	1

Monoisotopic mass of neutral peptide Mr(calc): 2325.3073
Fixed modifications: Carbamidomethyl (C) (apply to specified residues or termini only)
Variable modifications:
K17 : Trimethyl (K)
T20 : Phospho_STY (STY)
K21 : Trimethyl (K)
Ions Score: 74 **Expect:** 0.00017
Matches : 67/348 fragment ions using 77 most intense peaks (help)

Mascot Search Results: Peptide View

LLLPGELAKHAVSEGTKme3AVpTKme3

MS/MS Fragmentation of PEVAVKGATISKK
Found in sp|P70696|H2B1A_MOUSE in fgcz_10090, Histone H2B type 1-A OS=Mus musculus GN=Hist1h2ba

Match to Query 1680: 1406.749602 from(469.923810,3+) intensity(14895.2300) scans(3553) rtinseconds(2184.8056) index(2146)
Title: 2147: Scan 3553 (rt=36.4134) [s:\p749\Proteomics\ORBI_2\andreamizzi_20110517_sperm_trypsin_GluC_ETD_CID\20110517_05_swimup_NaCl_1.RAW]
Data file 20110517_05_swimup_NaCl_1.mgf

Click mouse within plot area to zoom in by factor of two about that point
Or, Plot from 200 to 1400 Da Full range
Label all possible matches ○ Label matches used for scoring ◉
Show Y-axis ☐

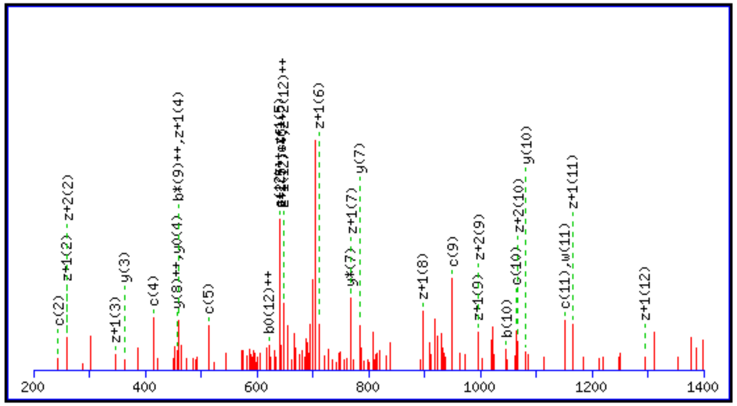

#	b	b++	b*	b*++	b0	b0++	c	c++	Seq.
1	98.0600	49.5337					115.0866	58.0469	P
2	227.1026	114.0550			209.0921	105.0497	244.1292	122.5682	E
3	326.1710	163.5892			308.1605	154.5839	343.1976	172.1024	V
4	397.2082	199.1077			379.1976	190.1024	414.2347	207.6210	A
5	496.2766	248.6419			478.2660	239.6366	513.3031	257.1552	V
6	624.3715	312.6894	607.3450	304.1761	606.3610	303.6841	641.3981	321.2027	K
7	681.3930	341.2001	664.3665	332.6869	663.3824	332.1949	698.4196	349.7134	G
8	752.4301	376.7187	735.4036	368.2054	734.4196	367.7134	769.4567	385.2320	A
9	933.4441	467.2257	916.4176	458.7124	915.4336	458.2204	950.4707	475.7390	T
10	1046.5282	523.7677	1029.5016	515.2545	1028.5176	514.7625	1063.5547	532.2810	I
11	1133.5602	567.2837	1116.5337	558.7705	1115.5497	558.2785	1150.5868	575.7970	S
12	1261.6552	631.3312	1244.6286	622.8180	1243.6446	622.3259	1278.6817	639.8445	K
13									K

Seq.	w	w'	y	y++	y*	y*++	y0	y0++	z+1	z+1++	z+2	z+2++	#
P													13
E	1235.6759		1310.7079	655.8576	1293.6814	647.3443	1292.6974	646.8523	1294.6892	647.8482	1295.6970	648.3522	12
V	1150.6232		1181.6654	591.3363	1164.6388	582.8230	1163.6548	582.3310	1165.6466	583.3270	1166.6545	583.8309	11
A			1082.5969	541.8021	1065.5704	533.2888	1064.5864	532.7968	1066.5782	533.7927	1067.5860	534.2967	10
V	980.5176		1011.5598	506.2836	994.5333	497.7703	993.5493	497.2783	995.5411	498.2742	996.5489	498.7781	9
K	838.4070		912.4914	456.7493	895.4649	448.2361	894.4808	447.7441	896.4727	448.7400	897.4805	449.2439	8
G			784.3964	392.7019	767.3699	384.1886	766.3859	383.6966	768.3777	384.6925	769.3855	385.1964	7
A			727.3750	364.1911	710.3484	355.6779	709.3644	355.1858	711.3563	356.1818	712.3641	356.6857	6
T	543.3501	545.3293	656.3379	328.6726	639.3113	320.1593	638.3273	319.6673	640.3191	320.6632	641.3270	321.1671	5
I	430.2660	444.2817	475.3239	238.1656	458.2973	229.6523	457.3133	229.1603	459.3051	230.1562	460.3130	230.6601	4
S	329.2183		362.2398	181.6235	345.2132	173.1103	344.2292	172.6183	346.2211	173.6142	347.2289	174.1181	3
K	201.1234		275.2078	138.1075	258.1812	129.5942			259.1890	130.0982	260.1969	130.6021	2
K	73.0284		147.1128	74.0600	130.0863	65.5468			131.0941	66.0507	132.1019	66.5546	1

Monoisotopic mass of neutral peptide Mr(calc): 1406.7534
Fixed modifications: Carbamidomethyl (C) (apply to specified residues or termini only)
Variable modifications:
T9 : Phospho_STY (STY)
Ions Score: 54 **Expect:** 0.0034
Matches : 34/211 fragment ions using 58 most intense peaks (help)

Mascot Search Results: Peptide View

PEVAVKGApTISKK

MS/MS Fragmentation of PEVAVKGATISKKGF
Found in sp|P70696|H2B1A_MOUSE in fgcz_10090, Histone H2B type 1-A OS=Mus musculus GN=Hist1h2ba

Match to Query 5144: 1610.843256 from(403.718090,4+) intensity(10058.1250) scans(3030) rtinseconds(2446.7592) index(8592)
Title: 944: Scan 3030 (rt=40.7793) [s:\p749\Proteomics\ORBI_2\andreamizzi_20110310_sperm_trypsin_chymo_AspN_ETD_CID\20110311_44_NaCl_chymotrypsin_SCX_4.RAW]
Data file Y:\p749\Proteomics\Mascot\mgf\53 20110426 sperm rerun chymotrypsin 2nd ETD run NaCl SCX\mascot_daemon_merge.mgf

Click mouse within plot area to zoom in by factor of two about that point
Or, Plot from 100 to 1700 Da Full range
Label all possible matches ○ Label matches used for scoring ⦿
Show Y-axis ☐

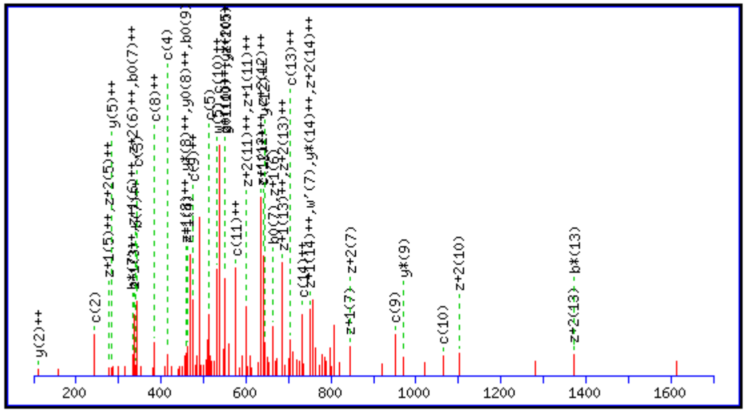

#	b	b^{++}	b^{*}	b^{*++}	b^{0}	b^{0++}	c	c^{++}	Seq.	w	w'	y	y^{++}	y^{*}	y^{*++}	y^{0}	y^{0++}	z+1	$z+1^{++}$	z+2	$z+2^{++}$	#
1	98.0600	49.5337					115.0866	58.0469	P													15
2	227.1026	114.0550			209.0921	105.0497	**244.1292**	122.5682	E	1439.7658		1514.7978	757.9025	1497.7713	749.3893	1496.7873	748.8973	1498.7791	***749.8932***	1499.7869	**750.3971**	14
3	326.1710	163.5892			308.1605	154.5839	**343.1976**	172.1024	V	1354.7130		1385.7552	693.3813	1368.7287	684.8680	1367.7447	684.3760	1369.7365	***685.3719***	**1370.7443**	**685.8758**	13
4	397.2082	199.1077			379.1976	190.1024	**414.2347**	207.6210	A			1286.6868	643.8470	1269.6603	635.3338	1268.6763	634.8418	1270.6681	***635.8377***	1271.6759	**636.3416**	12
5	496.2766	248.6419			478.2660	239.6366	**513.3031**	257.1552	V	1184.6075		1215.6497	608.3285	1198.6232	599.8152	1197.6391	599.3232	1199.6310	***600.3191***	1200.6388	**600.8230**	11
6	624.3715	312.6894	607.3450	304.1761	606.3610	303.6841	641.3981	321.2027	K	1042.4969		1116.5813	558.7943	1099.5547	550.2810	1098.5707	549.7890	1100.5626	***550.7849***	**1101.5704**	**551.2888**	10
7	681.3930	341.2001	664.3665	332.6869	663.3824	332.1949	698.4196	349.7134	G			988.4863	494.7468	971.4598	486.2335	970.4758	485.7415	972.4676	486.7374	973.4754	487.2414	9
8	752.4301	376.7187	735.4036	368.2054	734.4196	367.7134	769.4567	***385.2320***	A			931.4649	466.2361	914.4383	457.7228	913.4543	457.2308	915.4461	***458.2267***	916.4540	458.7306	8
9	933.4441	467.2257	916.4176	458.7124	915.4336	458.2204	**950.4707**	***475.7390***	T	747.4400	749.4192	860.4277	430.7175	843.4012	422.2042	842.4172	421.7122	**844.4090**	422.7081	**845.4168**	423.2121	7
10	1046.5282	523.7677	1029.5016	515.2545	1028.5176	514.7625	**1063.5547**	***532.2810***	I	634.3559	648.3715	679.4137	340.2105	662.3872	331.6972	661.4032	331.2052	**663.3950**	***332.2011***	664.4028	**332.7051**	6
11	1133.5602	567.2837	1116.5337	558.7705	1115.5497	558.2785	1150.5868	***575.7970***	S	533.3082		566.3297	283.6685	549.3031	275.1552	548.3191	274.6632	**550.3109**	***275.6591***	**551.3188**	**276.1630**	5
12	1261.6552	631.3312	1244.6286	622.8180	1243.6446	622.3259	1278.6817	***639.8445***	K	405.2132		479.2976	240.1525	462.2711	231.6392			**463.2789**	232.1431	464.2867	232.6470	4
13	1389.7501	695.3787	1372.7236	686.8654	1371.7396	686.3734	1406.7767	***703.8920***	K	277.1183		351.2027	176.1050	334.1761	167.5917			**335.1840**	168.0956	336.1918	168.5995	3
14	1446.7716	723.8894	1429.7451	715.3762	1428.7610	714.8842	1463.7982	***732.4027***	G			223.1077	112.0575					207.0890	104.0481	208.0968	104.5520	2
15									F	73.0284		166.0863	83.5468					150.0675	75.5374	151.0754	76.0413	1

Monoisotopic mass of neutral peptide Mr(calc): 1610.8433
Fixed modifications: Carbamidomethyl (C) (apply to specified residues or termini only)
Variable modifications:
T9 : Phospho_STY (STY)
Ions Score: 59 **Expect:** 0.003
Matches : 54/240 fragment ions using 54 most intense peaks (help)

Mascot Search Results: Peptide View

PEVAVKGApTISKKGF

MS/MS Fragmentation of PEVAVKGATISKKGFKK
Found in sp|P70696|H2B1A_MOUSE in fgcz_10090, Histone H2B type 1-A OS=Mus musculus GN=Hist1h2ba

Match to Query 3912: 1867.033456 from(467.765640,4+) intensity(156393.5800) scans(2753) rtinseconds(2088.5125) index(4118)
Title: 1092: Scan 2753 (rt=34.8085) [s:\p749\Proteomics\ORBI_2\andreamizzi_20110310_sperm_trypsin_chymo_AspN_ETD_CID\20110311_34_NaCl_trypsin_SCX_FT.RAW]
Data file Y:\p749\Proteomics\Mascot\mgf\50 20110426 sperm rerun trypsin 2nd ETD run NaCl SCX\mascot_daemon_merge.mgf

Click mouse within plot area to zoom in by factor of two about that point
Or, Plot from 200 to 1800 Da Full range
Label all possible matches ○ Label matches used for scoring ⦿
Show Y-axis ☐

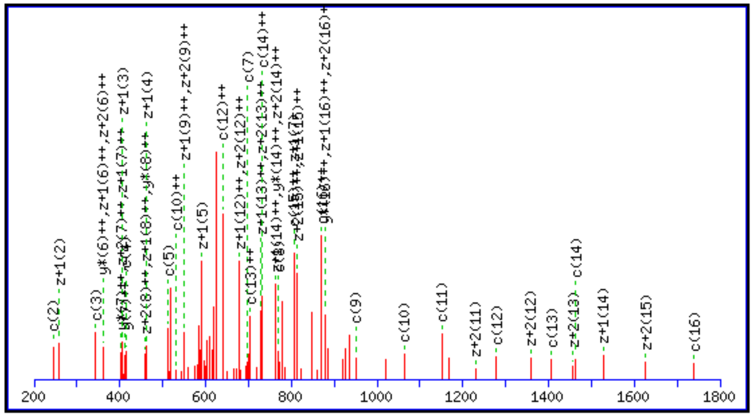

#	b	b^{++}	b^{*}	b^{*++}	b^{0}	b^{0++}	c	c^{++}	Seq.	w	w'	y	y^{++}	y^{*}	y^{*++}	y^{0}	y^{0++}	z+1	$z+1^{++}$	z+2	$z+2^{++}$	#
1	98.0600	49.5337					115.0866	58.0469	P													17
2	227.1026	114.0550			209.0921	105.0497	244.1292	122.5682	E	1695.9557		1770.9877	885.9975	1753.9612	877.4842	1752.9772	876.9922	1754.9690	877.9882	1755.9769	878.4921	16
3	326.1710	163.5892			308.1605	154.5839	343.1976	172.1024	V	1610.9030		1641.9452	821.4762	1624.9186	812.9629	1623.9346	812.4709	1625.9264	813.4669	1626.9343	813.9708	15
4	397.2082	199.1077			379.1976	190.1024	414.2347	207.6210	A			1542.8767	771.9420	1525.8502	763.4287	1524.8662	762.9367	1526.8580	763.9326	1527.8658	764.4366	14
5	496.2766	248.6419			478.2660	239.6366	513.3031	257.1552	V	1440.7974		1471.8396	736.4235	1454.8131	727.9102	1453.8291	727.4182	1455.8209	728.4141	1456.8287	728.9180	13
6	624.3715	312.6894	607.3450	304.1761	606.3610	303.6841	641.3981	321.2027	K	1298.6868		1372.7712	686.8892	1355.7447	678.3760	1354.7606	677.8840	1356.7525	678.8799	1357.7603	679.3838	12
7	681.3930	341.2001	664.3665	332.6869	663.3824	332.1949	698.4196	349.7134	G			1244.6763	622.8418	1227.6497	614.3285	1226.6657	613.8365	1228.6575	614.8324	1229.6654	615.3363	11
8	752.4301	376.7187	735.4036	368.2054	734.4196	367.7134	769.4567	385.2320	A			1187.6548	594.3310	1170.6282	585.8178	1169.6442	585.3257	1171.6361	586.3217	1172.6439	586.8256	10
9	933.4441	467.2257	916.4176	458.7124	915.4336	458.2204	950.4707	475.7390	T	1003.6299	1005.6091	1116.6177	558.8125	1099.5911	550.2992	1098.6071	549.8072	1100.5989	550.8031	1101.6068	551.3070	9
10	1046.5282	523.7677	1029.5016	515.2545	1028.5176	514.7625	1063.5547	532.2810	I	890.5458	904.5615	935.6037	468.3055	918.5771	459.7922	917.5931	459.3002	919.5849	460.2961	920.5928	460.8000	8
11	1133.5602	567.2837	1116.5337	558.7705	1115.5497	558.2785	1150.5868	575.7970	S	789.4981		822.5196	411.7634	805.4931	403.2502	804.5090	402.7582	806.5009	403.7541	807.5087	404.2580	7
12	1261.6552	631.3312	1244.6286	622.8180	1243.6446	622.3259	1278.6817	639.8445	K	661.4032		735.4876	368.2474	718.4610	359.7341			719.4688	360.2381	720.4767	360.7420	6
13	1389.7501	695.3787	1372.7236	686.8654	1371.7396	686.3734	1406.7767	703.8920	K	533.3082		607.3926	304.1999	590.3661	295.6867			591.3739	296.1906	592.3817	296.6945	5
14	1446.7716	723.8894	1429.7451	715.3762	1428.7610	714.8842	1463.7982	732.4027	G			479.2976	240.1525	462.2711	231.6392			463.2789	232.1431	464.2867	232.6470	4
15	1593.8400	797.4236	1576.8135	788.9104	1575.8295	788.4184	1610.8666	805.9369	F			422.2762	211.6417	405.2496	203.1285			406.2575	203.6324	407.2653	204.1363	3
16	1721.9350	861.4711	1704.9084	852.9579	1703.9244	852.4658	1738.9615	869.9844	K	201.1234		275.2078	138.1075	258.1812	129.5942			259.1890	130.0982	260.1969	130.6021	2
17									K	73.0284		147.1128	74.0600	130.0863	65.5468			131.0941	66.0507	132.1019	66.5546	1

Monoisotopic mass of neutral peptide Mr(calc): 1867.0332
Fixed modifications: Carbamidomethyl (C) (apply to specified residues or termini only)
Variable modifications:
T9 : Phospho_STY (STY)
Ions Score: 104 **Expect:** 2.4e-07
Matches : 53/277 fragment ions using 59 most intense peaks (help)

Mascot Search Results: Peptide View

PEVAVKGApTISKKGFKK

MS/MS Fragmentation of IAQDFKTDLRFQSAAIGALQE
Found in tr|E0CZ27|E0CZ27_MOUSE in fgcz_10090, Histone H3 OS=Mus musculus GN=H3f3a

Match to Query 4076: 2335.223472 from(779.415100,3+) intensity(270293.0000) scans(5883) rtinseconds(3102.4268) index(4219)
Title: 4220: Scan 5883 (rt=51.7071) [s:\p749\Proteomics\ORBI_2\andreamizzi_20110517_sperm_trypsin_GluC_ETD_CID\20110524_10_NaCl_GluC_2_II.RAW]
Data file 20110524_10_NaCl_GluC_2_II.mgf

Click mouse within plot area to zoom in by factor of two about that point
Or, Plot from 200 to 1100 Da Full range
Label all possible matches ○ Label matches used for scoring ◉
Show Y-axis ☐

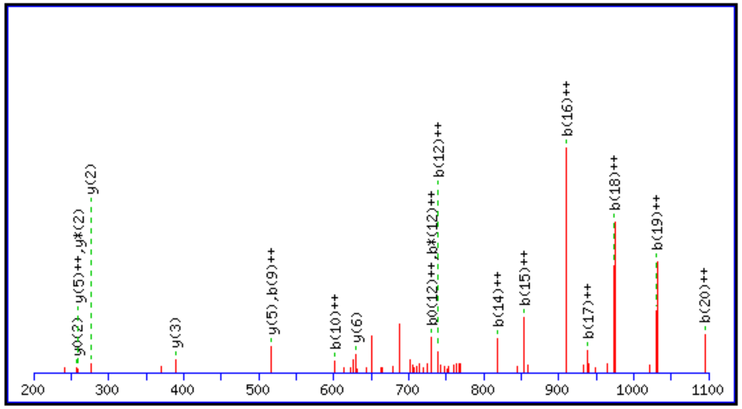

#	b	b^{++}	b^*	b^{*++}	b^0	b^{0++}	c	c^{++}	Seq.	w	w'	y	y^{++}	y^*	y^{*++}	y^0	y^{0++}	z+1	$z+1^{++}$	z+2	$z+2^{++}$	#
1	114.0913	57.5493					131.1179	66.0626	I													21
2	185.1285	93.0679					202.1550	101.5811	A			2223.1404	1112.0739	2206.1139	1103.5606	2205.1299	1103.0686	2207.1217	1104.0645	2208.1295	1104.5684	20
3	313.1870	157.0972	296.1605	148.5839			330.2136	165.6104	Q	2078.0553		2152.1033	1076.5553	2135.0768	1068.0420	2134.0927	1067.5500	2136.0846	1068.5459	2137.0924	1069.0498	19
4	428.2140	214.6106	411.1874	206.0974	410.2034	205.6053	445.2405	223.1239	D	1963.0284		2024.0447	1012.5260	2007.0182	1004.0127	2006.0342	1003.5207	2008.0260	1004.5166	2009.0338	1005.0206	18
5	575.2824	288.1448	558.2558	279.6316	557.2718	279.1396	592.3089	296.6581	F			1909.0178	955.0125	1891.9912	946.4993	1891.0072	946.0073	1892.9991	947.0032	1894.0069	947.5071	17
6	703.3774	352.1923	686.3508	343.6790	685.3668	343.1870	720.4039	360.7056	K	1687.8650		1761.9494	881.4783	1744.9228	872.9651	1743.9388	872.4730	1745.9307	873.4690	1746.9385	873.9729	16
7	804.4250	402.7162	787.3985	394.2029	786.4145	393.7109	821.4516	411.2294	T	1600.8329	1602.8122	1633.8544	817.4308	1616.8279	808.9176	1615.8438	808.4256	1617.8357	809.4215	1618.8435	809.9254	15
8	919.4520	460.2296	902.4254	451.7164	901.4414	451.2243	936.4785	468.7429	D	1471.7904		1532.8067	766.9070	1515.7802	758.3937	1514.7962	757.9017	1516.7880	758.8976	1517.7958	759.4016	14
9	1032.5360	**516.7717**	1015.5095	508.2584	1014.5255	507.7664	1049.5626	525.2849	L	1358.7063		1417.7798	709.3935	1400.7532	700.8803	1399.7692	700.3883	1401.7611	701.3842	1402.7689	701.8881	13
10	1202.6528	**601.8300**	1185.6263	593.3168	1184.6422	592.8248	1219.6793	610.3433	R	1188.5895		1304.6957	652.8515	1287.6692	644.3382	1286.6852	643.8462	1288.6770	644.8421	1289.6848	645.3461	12
11	1349.7212	675.3642	1332.6947	666.8510	1331.7106	666.3590	1366.7478	683.8775	F			1134.5790	567.7931	1117.5524	559.2798	1116.5684	558.7878	1118.5602	559.7838	1119.5681	560.2877	11
12	1477.7798	**739.3935**	1460.7532	730.8803	1459.7692	730.3883	1494.8063	747.9068	Q	913.4625		987.5106	494.2589	970.4840	485.7456	969.5000	485.2536	971.4918	486.2496	972.4997	486.7535	10
13	1564.8118	782.9095	1547.7853	774.3963	1546.8013	773.9043	1581.8384	791.4228	S	826.4305		859.4520	430.2296	842.4254	421.7164	841.4414	421.2243	843.4333	422.2203	844.4411	422.7242	9
14	1635.8489	**818.4281**	1618.8224	809.9148	1617.8384	809.4228	1652.8755	826.9414	A			772.4199	386.7136	755.3934	378.2003	754.4094	377.7083	756.4012	378.7042	757.4090	379.2082	8
15	1706.8860	**853.9467**	1689.8595	845.4334	1688.8755	844.9414	1723.9126	862.4599	A			701.3828	351.1951	684.3563	342.6818	683.3723	342.1898	685.3641	343.1857	686.3719	343.6896	7
16	1819.9701	**910.4887**	1802.9436	901.9754	1801.9595	901.4834	1836.9967	919.0020	I	585.2879	599.3035	**630.3457**	315.6765	613.3192	307.1632	612.3352	306.6712	614.3270	307.6671	615.3348	308.1710	6
17	1876.9916	**938.9994**	1859.9650	930.4862	1858.9810	929.9941	1894.0181	947.5127	G			**517.2617**	259.1345	500.2351	250.6212	499.2511	250.1292	501.2429	251.1251	502.2508	251.6290	5
18	1948.0287	**974.5180**	1931.0021	966.0047	1930.0181	965.5127	1965.0552	983.0313	A			460.2402	230.6237	443.2136	222.1105	442.2296	221.6185	444.2215	222.6144	445.2293	223.1183	4
19	2061.1128	**1031.0600**	2044.0862	1022.5467	2043.1022	1022.0547	2078.1393	1039.5733	L	330.1296		**389.2031**	195.1052	372.1765	186.5919	371.1925	186.0999	373.1844	187.0958	374.1922	187.5997	3
20	2189.1713	**1095.0893**	2172.1448	1086.5760	2171.1608	1086.0840	2206.1979	1103.6026	Q	202.0710		**276.1190**	138.5631	259.0925	130.0499	258.1084	129.5579	260.1003	130.5538	261.1081	131.0577	2
21									E	73.0284		148.0604	74.5339			130.0499	65.5286	132.0417	66.5245	133.0495	67.0284	1

Monoisotopic mass of neutral peptide Mr(calc): 2335.2172
Fixed modifications: Carbamidomethyl (C) (apply to specified residues or termini only)
Variable modifications:
R10 : Methyl_KR (KR)
Ions Score: 55 **Expect:** 0.017
Matches : 19/363 fragment ions using 29 most intense peaks (help)

Mascot Search Results: Peptide View

IAQDFKTDLRme1FQSAAIGALQE

MS/MS Fragmentation of KSAPATGGVKKPHR
Found in sp|P68433|H31_MOUSE in fgcz_10090, Histone H3.1 OS=Mus musculus GN=Hist1h3a

Match to Query 1715: 1446.841096 from(362.717550,4+) intensity(51074.3520) scans(1971) rtinseconds(1308.4027) index(1009)
Title: 1010: Scan 1971 (rt=21.8067) [s:\p749\Proteomics\ORBI_2\andreamizzi_20110310_sperm_trypsin_chymo_AspN_ETD_CID\20110310_11_NaCl_trypsin.RAW]
Data file \\fgcz-ms.uzh.ch\p749\Proteomics\ORBI_2\andreamizzi_20110310_sperm_trypsin_chymo_AspN_ETD_CID\mgf__low_res_MS2_nomerge\20110310_11_NaCl_trypsin.mgf

Click mouse within plot area to zoom in by factor of two about that point
Or, Plot from 100 to 1500 Da Full range
Label all possible matches ○ Label matches used for scoring ◉
Show Y-axis ☐

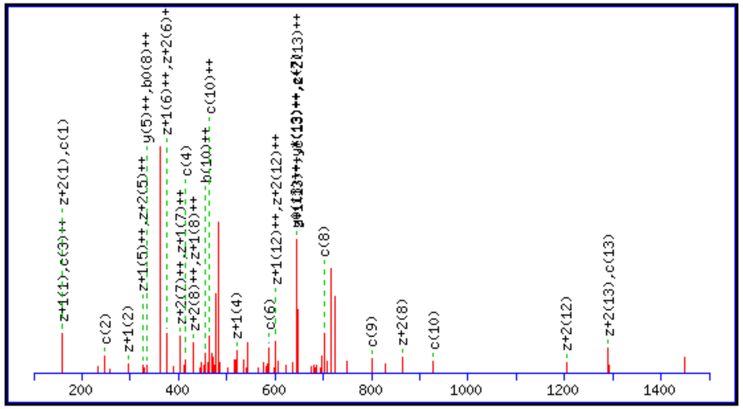

#	b	b++	b*	b*++	b0	b0++	c	c++	Seq.	w	w'	y	y++	y*	y*++	y0	y0++	z+1	z+1++	z+2	z+2++	#
1	143.1179	72.0626	126.0913	63.5493			160.1444	80.5759	K													14
2	230.1499	115.5786	213.1234	107.0653	212.1394	106.5733	247.1765	124.0919	S	1272.7171		1305.7386	653.3729	1288.7120	644.8597	1287.7280	644.3677	1289.7199	645.3636	1290.7277	645.8675	13
3	301.1870	151.0972	284.1605	142.5839	283.1765	142.0919	318.2136	159.6104	A			1218.7066	609.8569	1201.6800	601.3436	1200.6960	600.8516	1202.6878	601.8476	1203.6957	602.3515	12
4	398.2398	199.6235	381.2132	191.1103	380.2292	190.6183	415.2663	208.1368	P	1104.6273		1147.6695	574.3384	1130.6429	565.8251	1129.6589	565.3331	1131.6507	566.3290	1132.6586	566.8329	11
5	469.2769	235.1421	452.2504	226.6288	451.2663	226.1368	486.3035	243.6554	A			1050.6167	525.8120	1033.5901	517.2987	1032.6061	516.8067	1034.5980	517.8026	1035.6058	518.3065	10
6	570.3246	285.6659	553.2980	277.1527	552.3140	276.6606	587.3511	294.1792	T	946.5581	948.5374	979.5796	490.2934	962.5530	481.7802	961.5690	481.2881	963.5609	482.2841	964.5687	482.7880	9
7	627.3461	314.1767	610.3195	305.6634	609.3355	305.1714	644.3726	322.6899	G			878.5319	439.7696	861.5053	431.2563			862.5132	431.7602	863.5210	432.2641	8
8	684.3675	342.6874	667.3410	334.1741	666.3570	333.6821	701.3941	351.2007	G			821.5104	411.2589	804.4839	402.7456			805.4917	403.2495	806.4995	403.7534	7
9	783.4359	392.2216	766.4094	383.7083	765.4254	383.2163	800.4625	400.7349	V	733.4468		764.4890	382.7481	747.4624	374.2348			748.4702	374.7388	749.4781	375.2427	6
10	911.5309	456.2691	894.5043	447.7558	893.5203	447.2638	928.5574	464.7824	K	591.3362		665.4206	333.2139	648.3940	324.7006			649.4018	325.2046	650.4097	325.7085	5
11	1039.6259	520.3166	1022.5993	511.8033	1021.6153	511.3113	1056.6524	528.8298	K	463.2412		537.3256	269.1664	520.2990	260.6532			521.3069	261.1571	522.3147	261.6610	4
12	1136.6786	568.8429	1119.6521	560.3297	1118.6681	559.8377	1153.7052	577.3562	P	366.1884		409.2306	205.1190	392.2041	196.6057			393.2119	197.1096	394.2197	197.6135	3
13	1273.7375	637.3724	1256.7110	628.8591	1255.7270	628.3671	1290.7641	645.8857	H			312.1779	156.5926	295.1513	148.0793			296.1591	148.5832	297.1670	149.0871	2
14									R	73.0284		175.1190	88.0631	158.0924	79.5498			159.1002	80.0538	160.1081	80.5577	1

Monoisotopic mass of neutral peptide Mr(calc): 1446.8419
Fixed modifications: Carbamidomethyl (C) (apply to specified residues or termini only)
Variable modifications:
K1 : Methyl_KR (KR)
Ions Score: 56 **Expect:** 0.00041
Matches : 36/225 fragment ions using 40 most intense peaks (help)

Mascot Search Results: Peptide View

Kme1SAPATGGVKKPHR

MS/MS Fragmentation of KSAPATGGVKKPHR
Found in sp|P68433|H31_MOUSE in fgcz_10090, Histone H3.1 OS=Mus musculus GN=Hist1h3a

Match to Query 1767: 1474.874736 from(369.725960,4+) intensity(191721.0800) scans(1996) rtinseconds(1319.3775) index(1025)
Title: 1026: Scan 1996 (rt=21.9896) [s:\p749\Proteomics\ORBI_2\andreamizzi_20110310_sperm_trypsin_chymo_AspN_ETD_CID\20110310_11_NaCl_trypsin.RAW]
Data file \\fgcz-ms.uzh.ch\p749\Proteomics\ORBI_2\andreamizzi_20110310_sperm_trypsin_chymo_AspN_ETD_CID\mgf__low_res_MS2_nomerge\20110310_11_NaCl_trypsin.mgf

Click mouse within plot area to zoom in by factor of two about that point
Or, Plot from 100 to 1500 Da Full range
Label all possible matches ○ Label matches used for scoring ◉
Show Y-axis ☐

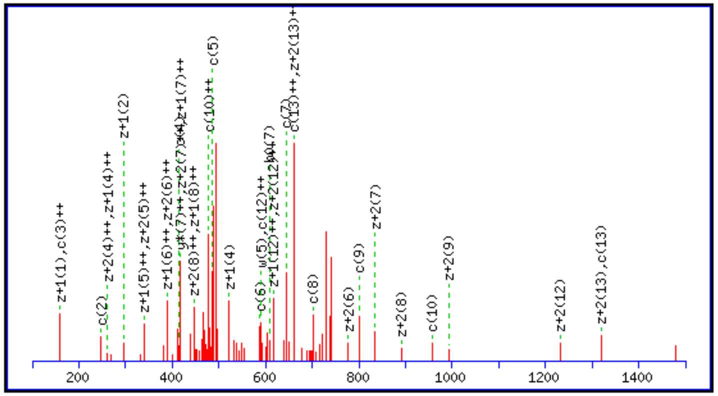

#	b	b^{++}	b^{*}	b^{*++}	b^{0}	b^{0++}	c	c^{++}	Seq.	w	w'	y	y^{++}	y^{*}	y^{*++}	y^{0}	y^{0++}	z+1	$z+1^{++}$	z+2	$z+2^{++}$	#
1	143.1179	72.0626	126.0913	63.5493			160.1444	80.5759	K													14
2	230.1499	115.5786	213.1234	107.0653	212.1394	106.5733	**247.1765**	124.0919	S	1300.7484		1333.7699	667.3886	1316.7433	658.8753	1315.7593	658.3833	1317.7512	659.3792	**1318.7590**	**659.8831**	13
3	301.1870	151.0972	284.1605	142.5839	283.1765	142.0919	318.2136	**159.6104**	A			1246.7379	623.8726	1229.7113	615.3593	1228.7273	614.8673	1230.7191	**615.8632**	**1231.7270**	**616.3671**	12
4	398.2398	199.6235	381.2132	191.1103	380.2292	190.6183	**415.2663**	208.1368	P	1132.6586		1175.7008	588.3540	1158.6742	579.8407	1157.6902	579.3487	1159.6820	580.3447	1160.6899	580.8486	11
5	469.2769	235.1421	452.2504	226.6288	451.2663	226.1368	**486.3035**	243.6554	A			1078.6480	539.8276	1061.6214	531.3144	1060.6374	530.8223	1062.6293	531.8183	1063.6371	532.3222	10
6	570.3246	285.6659	553.2980	277.1527	552.3140	276.6606	**587.3511**	294.1792	T	974.5894	976.5687	1007.6109	504.3091	990.5843	495.7958	989.6003	495.3038	991.5922	496.2997	**992.6000**	496.8036	9
7	627.3461	314.1767	610.3195	305.6634	609.3355	305.1714	**644.3726**	322.6899	G			906.5632	453.7852	889.5366	445.2720			890.5445	**445.7759**	**891.5523**	**446.2798**	8
8	684.3675	342.6874	667.3410	334.1741	666.3570	333.6821	**701.3941**	351.2007	G			849.5417	425.2745	832.5152	416.7612			833.5230	**417.2651**	**834.5308**	**417.7691**	7
9	783.4359	392.2216	766.4094	383.7083	765.4254	383.2163	**800.4625**	400.7349	V	761.4781		792.5203	396.7638	775.4937	388.2505			776.5015	**388.7544**	**777.5094**	**389.2583**	6
10	939.5622	470.2847	922.5356	461.7715	921.5516	461.2795	**956.5887**	**478.7980**	K	591.3362		693.4519	347.2296	676.4253	338.7163			677.4331	**339.2202**	678.4410	**339.7241**	5
11	1067.6572	534.3322	1050.6306	525.8189	1049.6466	525.3269	1084.6837	542.8455	K	463.2412		537.3256	269.1664	520.2990	260.6532			**521.3069**	**261.1571**	522.3147	**261.6610**	4
12	1164.7099	582.8586	1147.6834	574.3453	1146.6994	573.8533	1181.7365	**591.3719**	P	366.1884		409.2306	205.1190	392.2041	196.6057			393.2119	197.1096	394.2197	197.6135	3
13	1301.7688	651.3881	1284.7423	642.8748	1283.7583	642.3828	**1318.7954**	**659.9013**	H			312.1779	156.5926	295.1513	148.0793			**296.1591**	148.5832	297.1670	149.0871	2
14									R	73.0284		175.1190	88.0631	158.0924	79.5498			**159.1002**	80.0538	160.1081	80.5577	1

Monoisotopic mass of neutral peptide Mr(calc): 1474.8732
Fixed modifications: Carbamidomethyl (C) (apply to specified residues or termini only)
Variable modifications:
K1 : Methyl_KR (KR)
K10 : Dimethyl_KR (KR)
Ions Score: 63 **Expect:** 0.0006
Matches : 38/225 fragment ions using 43 most intense peaks (help)

Mascot Search Results: Peptide View

Kme1SAPATGGVKme2KPHR

MS/MS Fragmentation of **KSAPATGGVKKPHR**

Found in **sp|P68433|H31_MOUSE** in **fgcz_10090**, Histone H3.1 OS=Mus musculus GN=Hist1h3a

Match to Query 2777: 1460.859616 from(366.222180,4+) intensity(13951.9530) scans(1376) rtinseconds(1331.7536) index(3207)

Title: 181: Scan 1376 (rt=22.1959) [s:\p749\Proteomics\ORBI_2\andreamizzi_20110310_sperm_trypsin_chymo_AspN_ETD_CID\20110311_34_NaCl_trypsin_SCX_FT.RAW]

Data file Y:\p749\Proteomics\Mascot\mgf\50 20110426 sperm rerun trypsin 2nd ETD run NaCl SCX\mascot_daemon_merge.mgf

Click mouse within plot area to zoom in by factor of two about that point

Or, Plot from 100 to 1400 Da Full range

Label all possible matches ○ Label matches used for scoring ◉

Show Y-axis ☐

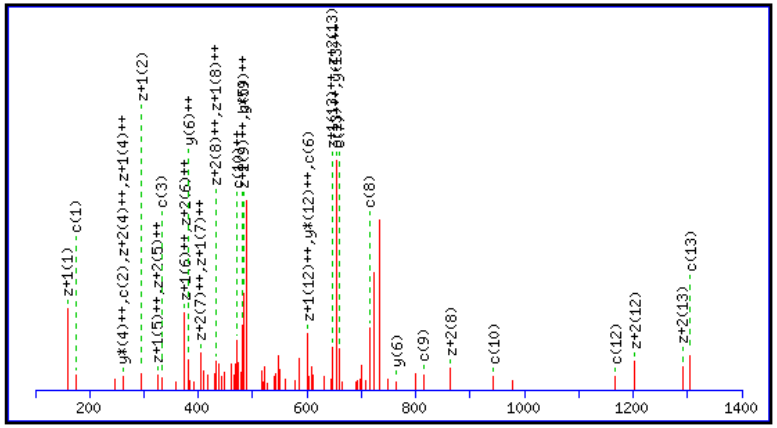

#	b	b^{++}	b^{*}	b^{*++}	b^{0}	b^{0++}	c	c^{++}	Seq.	w	w'	y	y^{++}	y^{*}	y^{*++}	y^{0}	y^{0++}	z+1	$z+1^{++}$	z+2	$z+2^{++}$	#
1	157.1335	79.0704	140.1070	70.5571			***174.1601***	87.5837	K													14
2	244.1656	122.5864	227.1390	114.0731	226.1550	113.5811	***261.1921***	131.0997	S	1272.7171		1305.7386	653.3729	1288.7120	644.8597	1287.7280	644.3677	1289.7199	**645.3636**	**1290.7277**	**645.8675**	13
3	315.2027	158.1050	298.1761	149.5917	297.1921	149.0997	***332.2292***	166.6183	A			1218.7066	609.8569	1201.6800	601.3436	1200.6960	600.8516	1202.6878	**601.8476**	**1203.6957**	602.3515	12
4	412.2554	206.6314	395.2289	198.1181	394.2449	197.6261	429.2820	215.1446	P	1104.6273		1147.6695	574.3384	1130.6429	565.8251	1129.6589	565.3331	1131.6507	566.3290	1132.6586	566.8329	11
5	483.2926	242.1499	466.2660	233.6366	465.2820	233.1446	500.3191	250.6632	A			1050.6167	525.8120	1033.5901	517.2987	1032.6061	516.8067	1034.5980	517.8026	1035.6058	518.3065	10
6	584.3402	292.6738	567.3137	284.1605	566.3297	283.6685	***601.3668***	301.1870	T	946.5581	948.5374	979.5796	490.2934	962.5530	481.7802	961.5690	481.2881	963.5609	**482.2841**	964.5687	**482.7880**	9
7	641.3617	321.1845	624.3352	312.6712	623.3511	312.1792	***658.3883***	329.6978	G			878.5319	439.7696	861.5053	431.2563			862.5132	**431.7602**	**863.5210**	**432.2641**	8
8	698.3832	349.6952	681.3566	341.1819	680.3726	340.6899	***715.4097***	358.2085	G			821.5104	411.2589	804.4839	402.7456			805.4917	**403.2495**	806.4995	**403.7534**	7
9	797.4516	399.2294	780.4250	390.7162	779.4410	390.2241	***814.4781***	407.7427	V	733.4468		764.4890	382.7481	747.4624	374.2348			748.4702	**374.7388**	749.4781	**375.2427**	6
10	925.5465	463.2769	908.5200	454.7636	907.5360	454.2716	***942.5731***	**471.7902**	K	591.3362		665.4206	333.2139	648.3940	324.7006			649.4018	**325.2046**	650.4097	**325.7085**	5
11	1053.6415	527.3244	1036.6150	518.8111	1035.6309	518.3191	1070.6681	535.8377	K	463.2412		537.3256	269.1664	520.2990	260.6532			521.3069	**261.1571**	522.3147	**261.6610**	4
12	1150.6943	575.8508	1133.6677	567.3375	1132.6837	566.8455	***1167.7208***	584.3640	P	366.1884		409.2306	205.1190	392.2041	196.6057			393.2119	197.1096	394.2197	197.6135	3
13	1287.7532	644.3802	1270.7266	635.8670	1269.7426	635.3749	***1304.7797***	**652.8935**	H			312.1779	156.5926	295.1513	148.0793			**296.1591**	148.5832	297.1670	149.0871	2
14									R	73.0284		175.1190	88.0631	158.0924	79.5498			**159.1002**	80.0538	160.1081	80.5577	1

Monoisotopic mass of neutral peptide Mr(calc): 1460.8576

Fixed modifications: Carbamidomethyl (C) (apply to specified residues or termini only)

Variable modifications:

K1 : Dimethyl_KR (KR)

Ions Score: 89 **Expect:** 1e-06

Matches : 39/225 fragment ions using 33 most intense peaks (help)

Mascot Search Results: Peptide View

Kme2SAPATGGVKKPHR

MS/MS Fragmentation of KSAPATGGVKKPHR
Found in sp|P68433|H31_MOUSE in fgcz_10090, Histone H3.1 OS=Mus musculus GN=Hist1h3a

Match to Query 2340: 1488.889336 from(373.229610,4+) intensity(23718.8750) scans(2307) rtinseconds(1399.9765) index(1369)
Title: 1370: Scan 2307 (rt=23.3329) [s:\p749\Proteomics\ORBI_2\andreamizzi_20110517_sperm_trypsin_GluC_ETD_CID\20110517_01_NaCl_1.RAW]
Data file 20110517_01_NaCl_1.mgf

Click mouse within plot area to zoom in by factor of two about that point
Or, Plot from 100 to 1400 Da Full range
Label all possible matches ○ Label matches used for scoring ◉
Show Y-axis ☐

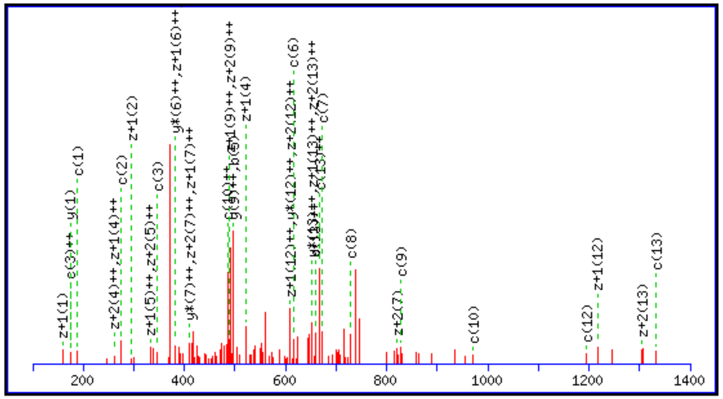

#	b	b^{++}	b^{*}	b^{*++}	b^{0}	b^{0++}	c	c^{++}	Seq.	w	w'	y	y^{++}	y^{*}	y^{*++}	y^{0}	y^{0++}	z+1	$z+1^{++}$	z+2	$z+2^{++}$	#
1	171.1492	86.0782	154.1226	77.5650			**188.1757**	94.5915	K													14
2	258.1812	129.5942	241.1547	121.0810	240.1707	120.5890	**275.2078**	138.1075	S	1286.7328		1319.7542	660.3808	1302.7277	651.8675	1301.7437	651.3755	1303.7355	**652.3714**	1304.7433	**652.8753**	13
3	329.2183	165.1128	312.1918	156.5995	311.2078	156.1075	**346.2449**	**173.6261**	A			1232.7222	616.8647	1215.6957	608.3515	1214.7117	607.8595	**1216.7035**	**608.8554**	1217.7113	**609.3593**	12
4	426.2711	213.6392	409.2445	205.1259	408.2605	204.6339	443.2976	222.1525	P	1118.6429		1161.6851	581.3462	1144.6586	572.8329	1143.6745	572.3409	1145.6664	573.3368	1146.6742	573.8407	11
5	497.3082	249.1577	480.2817	240.6445	479.2976	240.1525	514.3348	257.6710	A			1064.6323	532.8198	1047.6058	524.3065	1046.6218	523.8145	1048.6136	524.8104	1049.6214	525.3144	10
6	598.3559	299.6816	581.3293	291.1683	580.3453	290.6763	**615.3824**	308.1949	T	960.5738	962.5530	993.5952	497.3013	976.5687	488.7880	975.5847	488.2960	977.5765	**489.2919**	978.5843	**489.7958**	9
7	655.3774	328.1923	638.3508	319.6790	637.3668	319.1870	**672.4039**	336.7056	G			892.5475	446.7774	875.5210	438.2641			876.5288	438.7680	877.5366	439.2720	8
8	712.3988	356.7030	695.3723	348.1898	694.3883	347.6978	**729.4254**	365.2163	G			835.5261	418.2667	818.4995	409.7534			819.5074	**410.2573**	820.5152	**410.7612**	7
9	811.4672	406.2373	794.4407	397.7240	793.4567	397.2320	**828.4938**	414.7505	V	747.4624		778.5046	389.7559	761.4781	381.2427			762.4859	**381.7466**	763.4937	**382.2505**	6
10	953.5778	477.2926	936.5513	468.7793	935.5673	468.2873	**970.6044**	**485.8058**	K	591.3362		679.4362	340.2217	662.4097	331.7085			663.4175	**332.2124**	664.4253	**332.7163**	5
11	1081.6728	541.3400	1064.6463	532.8268	1063.6622	532.3348	1098.6994	549.8533	K	463.2412		537.3256	269.1664	520.2990	260.6532			**521.3069**	**261.1571**	522.3147	**261.6610**	4
12	1178.7256	589.8664	1161.6990	581.3531	1160.7150	580.8611	**1195.7521**	598.3797	P	366.1884		409.2306	205.1190	392.2041	196.6057			393.2119	197.1096	394.2197	197.6135	3
13	1315.7845	658.3959	1298.7579	649.8826	1297.7739	649.3906	**1332.8110**	**666.9092**	H			312.1779	156.5926	295.1513	148.0793			**296.1591**	148.5832	297.1670	149.0871	2
14									R	73.0284		175.1190	88.0631	158.0924	79.5498			**159.1002**	80.0538	160.1081	80.5577	1

Monoisotopic mass of neutral peptide Mr(calc): 1488.8889
Fixed modifications: Carbamidomethyl (C) (apply to specified residues or termini only)
Variable modifications:
K1 : Trimethyl (K)
K10 : Methyl_KR (KR)
Ions Score: 52 **Expect:** 0.0041
Matches : 41/225 fragment ions using 58 most intense peaks (help)

Mascot Search Results: Peptide View

Kme3SAPATGGVKme1KPHR

MS/MS Fragmentation of KSAPATGGVKKPHR
Found in sp|P68433|H31_MOUSE in fgcz_10090, Histone H3.1 OS=Mus musculus GN=Hist1h3a

Match to Query 2294: 1474.872656 from(369.725440,4+) intensity(22652.7930) scans(2291) rtinseconds(1392.7874) index(1360)
Title: 1361: Scan 2291 (rt=23.2131) [s:\p749\Proteomics\ORBI_2\andreamizzi_20110517_sperm_trypsin_GluC_ETD_CID\20110517_01_NaCl_1.RAW]
Data file 20110517_01_NaCl_1.mgf

Click mouse within plot area to zoom in by factor of two about that point
Or, Plot from 100 to 1400 Da Full range
Label all possible matches ○ Label matches used for scoring ◉
Show Y-axis ☐

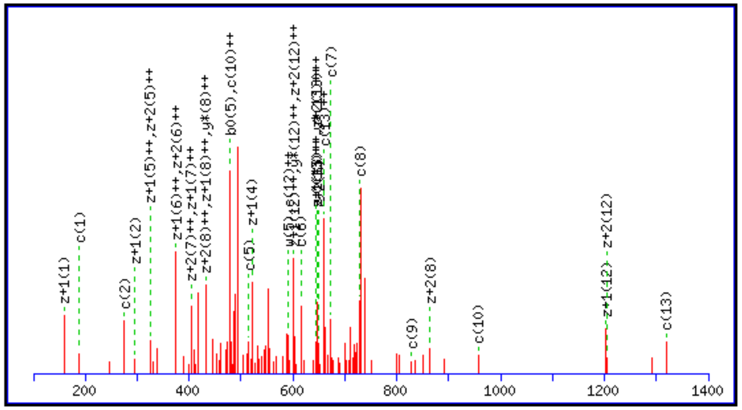

#	b	b^{++}	b^{*}	b^{*++}	b^{0}	b^{0++}	c	c^{++}	Seq.	w	w'	y	y^{++}	y^{*}	y^{*++}	y^{0}	y^{0++}	z+1	$z+1^{++}$	z+2	$z+2^{++}$	#
1	171.1492	86.0782	154.1226	77.5650			***188.1757***	94.5915	K													14
2	258.1812	129.5942	241.1547	121.0810	240.1707	120.5890	***275.2078***	138.1075	S	1272.7171		1305.7386	653.3729	1288.7120	644.8597	1287.7280	644.3677	1289.7199	**645.3636**	1290.7277	**645.8675**	13
3	329.2183	165.1128	312.1918	156.5995	311.2078	156.1075	346.2449	173.6261	A			1218.7066	609.8569	1201.6800	601.3436	1200.6960	600.8516	**1202.6878**	**601.8476**	**1203.6957**	**602.3515**	12
4	426.2711	213.6392	409.2445	205.1259	408.2605	204.6339	443.2976	222.1525	P	1104.6273		1147.6695	574.3384	1130.6429	565.8251	1129.6589	565.3331	1131.6507	566.3290	1132.6586	566.8329	11
5	497.3082	249.1577	480.2817	240.6445	479.2976	240.1525	***514.3348***	257.6710	A			1050.6167	525.8120	1033.5901	517.2987	1032.6061	516.8067	1034.5980	517.8026	1035.6058	518.3065	10
6	598.3559	299.6816	581.3293	291.1683	580.3453	290.6763	***615.3824***	308.1949	T	946.5581	948.5374	979.5796	490.2934	962.5530	481.7802	961.5690	481.2881	963.5609	482.2841	964.5687	482.7880	9
7	655.3774	328.1923	638.3508	319.6790	637.3668	319.1870	***672.4039***	336.7056	G			878.5319	439.7696	861.5053	431.2563			862.5132	**431.7602**	**863.5210**	**432.2641**	8
8	712.3988	356.7030	695.3723	348.1898	694.3883	347.6978	***729.4254***	365.2163	G			821.5104	411.2589	804.4839	402.7456			805.4917	**403.2495**	806.4995	**403.7534**	7
9	811.4672	406.2373	794.4407	397.7240	793.4567	397.2320	***828.4938***	414.7505	V	733.4468		764.4890	382.7481	747.4624	374.2348			748.4702	**374.7388**	749.4781	**375.2427**	6
10	939.5622	470.2847	922.5356	461.7715	921.5516	461.2795	***956.5887***	**478.7980**	K	591.3362		665.4206	333.2139	648.3940	324.7006			649.4018	**325.2046**	**650.4097**	**325.7085**	5
11	1067.6572	534.3322	1050.6306	525.8189	1049.6466	525.3269	1084.6837	542.8455	K	463.2412		537.3256	269.1664	520.2990	260.6532			**521.3069**	261.1571	522.3147	261.6610	4
12	1164.7099	582.8586	1147.6834	574.3453	1146.6994	573.8533	1181.7365	**591.3719**	P	366.1884		409.2306	205.1190	392.2041	196.6057			393.2119	197.1096	394.2197	197.6135	3
13	1301.7688	651.3881	1284.7423	642.8748	1283.7583	642.3828	***1318.7954***	**659.9013**	H			312.1779	156.5926	295.1513	148.0793			**296.1591**	148.5832	297.1670	149.0871	2
14									R	73.0284		175.1190	88.0631	158.0924	79.5498			**159.1002**	80.0538	160.1081	80.5577	1

Monoisotopic mass of neutral peptide Mr(calc): 1474.8732
Fixed modifications: Carbamidomethyl (C) (apply to specified residues or termini only)
Variable modifications:
K1 : Trimethyl (K)
Ions Score: 44 **Expect:** 0.03
Matches : 37/225 fragment ions using 61 most intense peaks (help)

Mascot Search Results: Peptide View

Kme3SAPATGGVKKPHR

MS/MS Fragmentation of KSAPATGGVKKPHR
Found in sp|P68433|H31_MOUSE in fgcz_10090, Histone H3.1 OS=Mus musculus GN=Hist1h3a

Match to Query 1594: 1488.888816 from(373.229480,4+) intensity(92494.2340) scans(1888) rtinseconds(1397.7518) index(1014)
Title: 1015: Scan 1888 (rt=23.2959) [s:\p749\Proteomics\ORBI_2\andreamizzi_20110517_sperm_trypsin_GluC_ETD_CID\20110519_01_NaCl_ziptipped_1.RAW]
Data file 20110519_01_NaCl_ziptipped_1.mgf

Click mouse within plot area to zoom in by factor of two about that point
Or, Plot from 100 to 1500 Da Full range
Label all possible matches ○ Label matches used for scoring ◉
Show Y-axis ☐

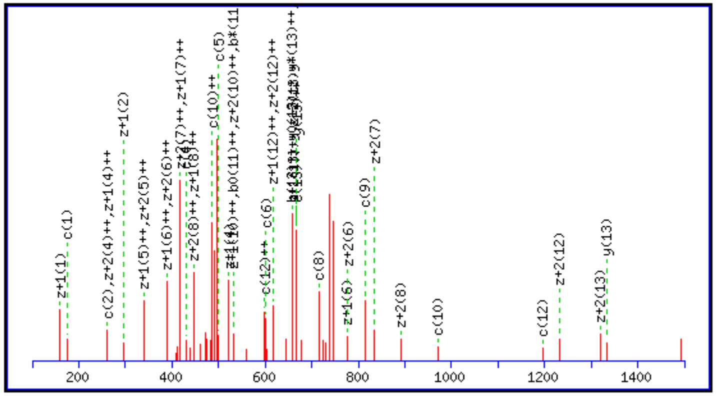

#	b	b^{++}	b^{*}	b^{*++}	b^{0}	b^{0++}	c	c^{++}	Seq.	w	w'	y	y^{++}	y^{*}	y^{*++}	y^{0}	y^{0++}	z+1	$z+1^{++}$	z+2	$z+2^{++}$	#
1	157.1335	79.0704	140.1070	70.5571			***174.1601***	87.5837	K													14
2	244.1656	122.5864	227.1390	114.0731	226.1550	113.5811	***261.1921***	131.0997	S	1300.7484		1333.7699	667.3886	1316.7433	658.8753	1315.7593	658.3833	1317.7512	**659.3792**	**1318.7590**	**659.8831**	13
3	315.2027	158.1050	298.1761	149.5917	297.1921	149.0997	332.2292	166.6183	A			1246.7379	623.8726	1229.7113	615.3593	1228.7273	614.8673	1230.7191	**615.8632**	**1231.7270**	**616.3671**	12
4	412.2554	206.6314	395.2289	198.1181	394.2449	197.6261	***429.2820***	215.1446	P	1132.6586		1175.7008	588.3540	1158.6742	579.8407	1157.6902	579.3487	1159.6820	580.3447	1160.6899	580.8486	11
5	483.2926	242.1499	466.2660	233.6366	465.2820	233.1446	***500.3191***	250.6632	A			1078.6480	539.8276	1061.6214	531.3144	1060.6374	530.8224	1062.6293	**531.8183**	1063.6371	**532.3222**	10
6	584.3402	292.6738	567.3137	284.1605	566.3297	283.6685	***601.3668***	301.1870	T	974.5894	976.5687	1007.6109	504.3091	990.5843	495.7958	989.6003	495.3038	991.5922	496.2997	992.6000	496.8036	9
7	641.3617	321.1845	624.3352	312.6712	623.3511	312.1792	***658.3883***	329.6978	G			906.5632	453.7852	889.5366	445.2720			890.5445	**445.7759**	**891.5523**	**446.2798**	8
8	698.3832	349.6952	681.3566	341.1819	680.3726	340.6899	***715.4097***	358.2085	G			849.5417	425.2745	832.5152	416.7612			833.5230	**417.2651**	**834.5308**	**417.7691**	7
9	797.4516	399.2294	780.4250	390.7162	779.4410	390.2241	***814.4781***	407.7427	V	761.4781		792.5203	396.7638	775.4937	388.2505			**776.5015**	**388.7544**	**777.5094**	**389.2583**	6
10	953.5778	477.2926	936.5513	468.7793	935.5673	468.2873	***970.6044***	**485.8058**	K	591.3362		693.4519	347.2296	676.4253	338.7163			677.4331	**339.2202**	678.4410	**339.7241**	5
11	1081.6728	541.3400	1064.6463	532.8268	1063.6622	532.3348	1098.6994	549.8533	K	463.2412		537.3256	269.1664	520.2990	260.6532			**521.3069**	**261.1571**	522.3147	**261.6610**	4
12	1178.7256	589.8664	1161.6990	581.3531	1160.7150	580.8611	***1195.7521***	**598.3797**	P	366.1884		409.2306	205.1190	392.2041	196.6057			393.2119	197.1096	394.2197	197.6135	3
13	1315.7845	658.3959	1298.7579	649.8826	1297.7739	649.3906	1332.8110	**666.9092**	H			312.1779	156.5926	295.1513	148.0793			**296.1591**	148.5832	297.1670	149.0871	2
14									R	73.0284		175.1190	88.0631	158.0924	79.5498			**159.1002**	80.0538	160.1081	80.5577	1

Monoisotopic mass of neutral peptide Mr(calc): 1488.8889
Fixed modifications: Carbamidomethyl (C) (apply to specified residues or termini only)
Variable modifications:
K1 : Dimethyl_KR (KR)
K10 : Dimethyl_KR (KR)
Ions Score: 77 **Expect:** 2.4e-05
Matches : 45/225 fragment ions using 43 most intense peaks (help)

Mascot Search Results: Peptide View

Kme2SAPATGGVKme2KPHR

MS/MS Fragmentation of KSAPATGGVKKPHRYRPGTVALR
Found in sp|P68433|H31_MOUSE in fgcz_10090, Histone H3.1 OS=Mus musculus GN=Hist1h3a

Match to Query 2924: 2502.457320 from(501.498740,5+) intensity(451162.3800) scans(2374) rtinseconds(1798.6516) index(1072)
Title: 1073: Scan 2374 (rt=29.9775) [s:\p749\Proteomics\ORBI_2\andreamizzi_20110310_sperm_trypsin_chymo_AspN_ETD_CID\20110310_04_rep_2h7.RAW]
Data file \\fgcz-ms.uzh.ch\p749\Proteomics\ORBI_2\andreamizzi_20110310_sperm_trypsin_chymo_AspN_ETD_CID\mgf__low_res_MS2_nomerge\20110310_04_rep_2h7.mgf

Click mouse within plot area to zoom in by factor of two about that point
Or, Plot from 100 to 1600 Da Full range
Label all possible matches ○ Label matches used for scoring ◉
Show Y-axis ☐

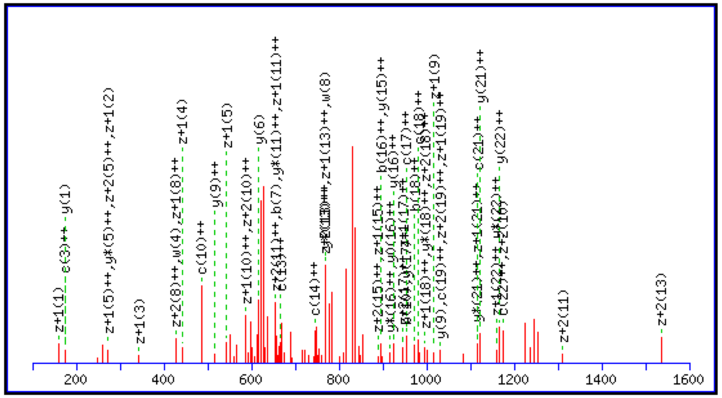

#	b	b++	b*	b*++	b0	b0++	c	c++	Seq.	w	w'	y	y++	y*	y*++	y0	y0++	z+1	z+1++	z+2	z+2++	#
1	171.1492	86.0782	154.1226	77.5650			188.1757	94.5915	K													23
2	258.1812	129.5942	241.1547	121.0810	240.1707	120.5890	275.2078	138.1075	S	2300.3098		2333.3313	1167.1693	2316.3047	1158.6560	2315.3207	1158.1640	2317.3126	1159.1599	2318.3204	1159.6638	22
3	329.2183	165.1128	312.1918	156.5995	311.2078	156.1075	346.2449	173.6261	A			2246.2993	1123.6533	2229.2727	1115.1400	2228.2887	1114.6480	2230.2805	1115.6439	2231.2884	1116.1478	21
4	426.2711	213.6392	409.2445	205.1259	408.2605	204.6339	443.2976	222.1525	P	2132.2200		2175.2622	1088.1347	2158.2356	1079.6214	2157.2516	1079.1294	2159.2434	1080.1254	2160.2513	1080.6293	20
5	497.3082	249.1577	480.2817	240.6445	479.2976	240.1525	514.3348	257.6710	A			2078.2094	1039.6083	2061.1828	1031.0951	2060.1988	1030.6030	2062.1907	1031.5990	2063.1985	1032.1029	19
6	598.3559	299.6816	581.3293	291.1683	580.3453	290.6763	615.3824	308.1949	T	1974.1508	1976.1301	2007.1723	1004.0898	1990.1457	995.5765	1989.1617	995.0845	1991.1536	996.0804	1992.1614	996.5843	18
7	655.3774	328.1923	638.3508	319.6790	637.3668	319.1870	672.4039	336.7056	G			1906.1246	953.5659	1889.0980	945.0527	1888.1140	944.5607	1890.1059	945.5566	1891.1137	946.0605	17
8	712.3988	356.7030	695.3723	348.1898	694.3883	347.6978	729.4254	365.2163	G			1849.1031	925.0552	1832.0766	916.5419	1831.0926	916.0499	1833.0844	917.0458	1834.0922	917.5498	16
9	811.4672	406.2373	794.4407	397.7240	793.4567	397.2320	828.4938	414.7505	V	1761.0395		1792.0817	896.5445	1775.0551	888.0312	1774.0711	887.5392	1776.0629	888.5351	1777.0708	889.0390	15
10	953.5778	477.2926	936.5513	468.7793	935.5673	468.2873	970.6044	485.8058	K	1604.9132		1693.0133	847.0103	1675.9867	838.4970	1675.0027	838.0050	1676.9945	839.0009	1678.0024	839.5048	14
11	1081.6728	541.3400	1064.6463	532.8268	1063.6622	532.3348	1098.6994	549.8533	K	1476.8182		1550.9026	775.9550	1533.8761	767.4417	1532.8921	766.9497	1534.8839	767.9456	1535.8917	768.4495	13
12	1178.7256	589.8664	1161.6990	581.3531	1160.7150	580.8611	1195.7521	598.3797	P	1379.7655		1422.8077	711.9075	1405.7811	703.3942	1404.7971	702.9022	1406.7890	703.8981	1407.7968	704.4020	12
13	1315.7845	658.3959	1298.7579	649.8826	1297.7739	649.3906	1332.8110	666.9092	H			1325.7549	663.3811	1308.7284	654.8678	1307.7443	654.3758	1309.7362	655.3717	1310.7440	655.8756	11
14	1471.8856	736.4464	1454.8590	727.9332	1453.8750	727.4412	1488.9121	744.9597	R	1086.6055		1188.6960	594.8516	1171.6695	586.3384	1170.6854	585.8464	1172.6773	586.8423	1173.6851	587.3462	10
15	1634.9489	817.9781	1617.9224	809.4648	1616.9384	808.9728	1651.9755	826.4914	Y			1032.5949	516.8011	1015.5683	508.2878	1014.5843	507.7958	1016.5762	508.7917	1017.5840	509.2956	9
16	1791.0500	896.0287	1774.0235	887.5154	1773.0395	887.0234	1808.0766	904.5419	R	767.4410		869.5316	435.2694	852.5050	426.7561	851.5210	426.2641	853.5128	427.2601	854.5207	427.7640	8
17	1888.1028	944.5550	1871.0762	936.0418	1870.0922	935.5498	1905.1293	953.0683	P	670.3883		713.4305	357.2189	696.4039	348.7056	695.4199	348.2136	697.4117	349.2095	698.4196	349.7134	7
18	1945.1243	973.0658	1928.0977	964.5525	1927.1137	964.0605	1962.1508	981.5790	G			616.3777	308.6925	599.3511	300.1792	598.3671	299.6872	600.3590	300.6831	601.3668	301.1870	6
19	2046.1719	1023.5896	2029.1454	1015.0763	2028.1614	1014.5843	2063.1985	1032.1029	T	526.3348	528.3140	559.3562	280.1817	542.3297	271.6685	541.3457	271.1765	543.3375	272.1724	544.3453	272.6763	5
20	2145.2404	1073.1238	2128.2138	1064.6105	2127.2298	1064.1185	2162.2669	1081.6371	V	427.2663		458.3085	229.6579	441.2820	221.1446			442.2898	221.6485	443.2976	222.1525	4
21	2216.2775	1108.6424	2199.2509	1100.1291	2198.2669	1099.6371	2233.3040	1117.1556	A			359.2401	180.1237	342.2136	171.6104			343.2214	172.1143	344.2292	172.6183	3
22	2329.3615	1165.1844	2312.3350	1156.6711	2311.3510	1156.1791	2346.3881	1173.6977	L	229.1295		288.2030	144.6051	271.1765	136.0919			272.1843	136.5958	273.1921	137.0997	2
23									R	73.0284		175.1190	88.0631	158.0924	79.5498			159.1002	80.0538	160.1081	80.5577	1

Monoisotopic mass of neutral peptide Mr(calc): 2502.4659
Fixed modifications: Carbamidomethyl (C) (apply to specified residues or termini only)
Variable modifications:
K1 : Trimethyl (K)
K10 : Methyl_KR (KR)
Ions Score: 60 **Expect:** 0.0039
Matches : 61/402 fragment ions using 66 most intense peaks (help)

Mascot Search Results: Peptide View

Kme3SAPATGGVKme1KPHRYRPGTVALR

MS/MS Fragmentation of KSAPATGGVKKPHRYRPGTVALR
Found in sp|P68433|H31_MOUSE in fgcz_10090, Histone H3.1 OS=Mus musculus GN=Hist1h3a

Match to Query 5413: 2502.467136 from(626.624060,4+) intensity(60804.8090) scans(2553) rtinseconds(1890.4771) index(1122)
Title: 978: Scan 2553 (rt=31.508) [s:\p749\Proteomics\ORBI_2\andreamizzi_20110310_sperm_trypsin_chymo_AspN_ETD_CID\20110311_37_NaCl_trypsin_SCX_3.RAW]
Data file Y:\p749\Proteomics\Mascot\mgf\50 20110426 sperm rerun trypsin 2nd ETD run NaCl SCX\mascot_daemon_merge.mgf

Click mouse within plot area to zoom in by factor of two about that point
Or, Plot from 200 to 2000 Da Full range
Label all possible matches ○ Label matches used for scoring ◉
Show Y-axis ☐

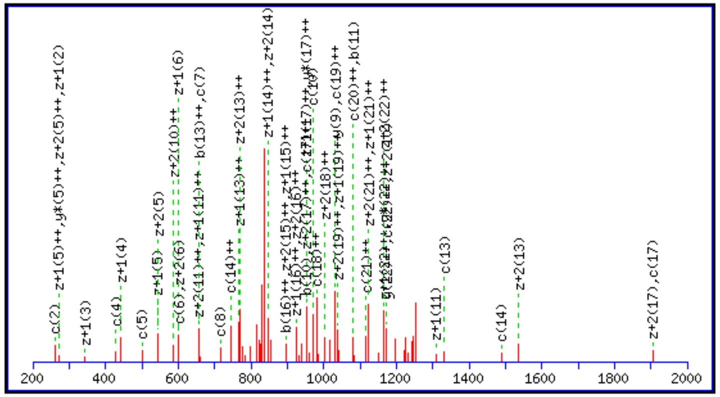

#	b	b^{++}	b^{*}	b^{*++}	b^{0}	b^{0++}	c	c^{++}	Seq.	w	w'	y	y^{++}	y^{*}	y^{*++}	y^{0}	y^{0++}	z+1	$z+1^{++}$	z+2	$z+2^{++}$	#
1	157.1335	79.0704	140.1070	70.5571			174.1601	87.5837	K													23
2	244.1656	122.5864	227.1390	114.0731	226.1550	113.5811	261.1921	131.0997	S	2314.3255		2347.3469	1174.1771	2330.3204	1165.6638	2329.3364	1165.1718	2331.3282	1166.1677	2332.3360	1166.6717	22
3	315.2027	158.1050	298.1761	149.5917	297.1921	149.0997	332.2292	166.6183	A			2260.3149	1130.6611	2243.2884	1122.1478	2242.3044	1121.6558	2244.2962	1122.6517	2245.3040	1123.1556	21
4	412.2554	206.6314	395.2289	198.1181	394.2449	197.6261	429.2820	215.1446	P	2146.2356		2189.2778	1095.1425	2172.2513	1086.6293	2171.2672	1086.1373	2173.2591	1087.1332	2174.2669	1087.6371	20
5	483.2926	242.1499	466.2660	233.6366	465.2820	233.1446	500.3191	250.6632	A			2092.2250	1046.6162	2075.1985	1038.1029	2074.2145	1037.6109	2076.2063	1038.6068	2077.2141	1039.1107	19
6	584.3402	292.6738	567.3137	284.1605	566.3297	283.6685	601.3668	301.1870	T	1988.1665	1990.1457	2021.1879	1011.0976	2004.1614	1002.5843	2003.1774	1002.0923	2005.1692	1003.0882	2006.1770	1003.5922	18
7	641.3617	321.1845	624.3352	312.6712	623.3511	312.1792	658.3883	329.6978	G			1920.1402	960.5738	1903.1137	952.0605	1902.1297	951.5685	1904.1215	952.5644	1905.1293	953.0683	17
8	698.3832	349.6952	681.3566	341.1819	680.3726	340.6899	715.4097	358.2085	G			1863.1188	932.0630	1846.0922	923.5498	1845.1082	923.0577	1847.1001	924.0537	1848.1079	924.5576	16
9	797.4516	399.2294	780.4250	390.7162	779.4410	390.2241	814.4781	407.7427	V	1775.0551		1806.0973	903.5523	1789.0708	895.0390	1788.0868	894.5470	1790.0786	895.5429	1791.0864	896.0468	15
10	953.5778	477.2926	936.5513	468.7793	935.5673	468.2873	970.6044	485.8058	K	1604.9132		1707.0289	854.0181	1690.0024	845.5048	1689.0183	845.0128	1691.0102	846.0087	1692.0180	846.5126	14
11	1081.6728	541.3400	1064.6463	532.8268	1063.6622	532.3348	1098.6994	549.8533	K	1476.8182		1550.9026	775.9550	1533.8761	767.4417	1532.8921	766.9497	1534.8839	767.9456	1535.8917	768.4495	13
12	1178.7256	589.8664	1161.6990	581.3531	1160.7150	580.8611	1195.7521	598.3797	P	1379.7655		1422.8077	711.9075	1405.7811	703.3942	1404.7971	702.9022	1406.7890	703.8981	1407.7968	704.4020	12
13	1315.7845	658.3959	1298.7579	649.8826	1297.7739	649.3906	1332.8110	666.9092	H			1325.7549	663.3811	1308.7284	654.8678	1307.7443	654.3758	1309.7362	655.3717	1310.7440	655.8756	11
14	1471.8856	736.4464	1454.8590	727.9332	1453.8750	727.4412	1488.9121	744.9597	R	1086.6055		1188.6960	594.8516	1171.6695	586.3384	1170.6854	585.8464	1172.6773	586.8423	1173.6851	587.3462	10
15	1634.9489	817.9781	1617.9224	809.4648	1616.9384	808.9728	1651.9755	826.4914	Y			1032.5949	516.8011	1015.5683	508.2878	1014.5843	507.7958	1016.5762	508.7917	1017.5840	509.2956	9
16	1791.0500	896.0287	1774.0235	887.5154	1773.0395	887.0234	1808.0766	904.5419	R	767.4410		869.5316	435.2694	852.5050	426.7561	851.5210	426.2641	853.5128	427.2601	854.5207	427.7640	8
17	1888.1028	944.5550	1871.0762	936.0418	1870.0922	935.5498	1905.1293	953.0683	P	670.3883		713.4305	357.2189	696.4039	348.7056	695.4199	348.2136	697.4117	349.2095	698.4196	349.7134	7
18	1945.1243	973.0658	1928.0977	964.5525	1927.1137	964.0605	1962.1508	981.5790	G			616.3777	308.6925	599.3511	300.1792	598.3671	299.6872	600.3590	300.6831	601.3668	301.1870	6
19	2046.1719	1023.5896	2029.1454	1015.0763	2028.1614	1014.5843	2063.1985	1032.1029	T	526.3348	528.3140	559.3562	280.1817	542.3297	271.6685	541.3457	271.1765	543.3375	272.1724	544.3453	272.6763	5
20	2145.2404	1073.1238	2128.2138	1064.6105	2127.2298	1064.1185	2162.2669	1081.6371	V	427.2663		458.3085	229.6579	441.2820	221.1446			442.2898	221.6485	443.2976	222.1525	4
21	2216.2775	1108.6424	2199.2509	1100.1291	2198.2669	1099.6371	2233.3040	1117.1556	A			359.2401	180.1237	342.2136	171.6104			343.2214	172.1143	344.2292	172.6183	3
22	2329.3615	1165.1844	2312.3350	1156.6711	2311.3510	1156.1791	2346.3881	1173.6977	L	229.1295		288.2030	144.6051	271.1765	136.0919			272.1843	136.5958	273.1921	137.0997	2
23									R	73.0284		175.1190	88.0631	158.0924	79.5498			159.1002	80.0538	160.1081	80.5577	1

Monoisotopic mass of neutral peptide Mr(calc): 2502.4659
Fixed modifications: Carbamidomethyl (C) (apply to specified residues or termini only)
Variable modifications:
K1 : Dimethyl_KR (KR)
K10 : Dimethyl_KR (KR)
Ions Score: 101 **Expect:** 2.9e-07
Matches : 59/402 fragment ions using 45 most intense peaks (help)

Mascot Search Results: Peptide View

Kme2SAPATGGVKme2KPHRYRPGTVALR

MS/MS Fragmentation of KSAPATGGVKKPHRYRPGTVALR
Found in sp|P68433|H31_MOUSE in fgcz_10090, Histone H3.1 OS=Mus musculus GN=Hist1h3a

Match to Query 3897: 2488.450170 from(498.697310,5+) intensity(304093.7800) scans(3368) rtinseconds(1967.9339) index(2096)
Title: 2097: Scan 3368 (rt=32.7989) [s:\p749\Proteomics\ORBI_2\andreamizzi_20110517_sperm_trypsin_GluC_ETD_CID\20110517_01_NaCl_1.RAW]
Data file 20110517_01_NaCl_1.mgf

Click mouse within plot area to zoom in by factor of two about that point
Or, Plot from 100 to 1600 Da Full range
Label all possible matches ○ Label matches used for scoring ◉
Show Y-axis ☐

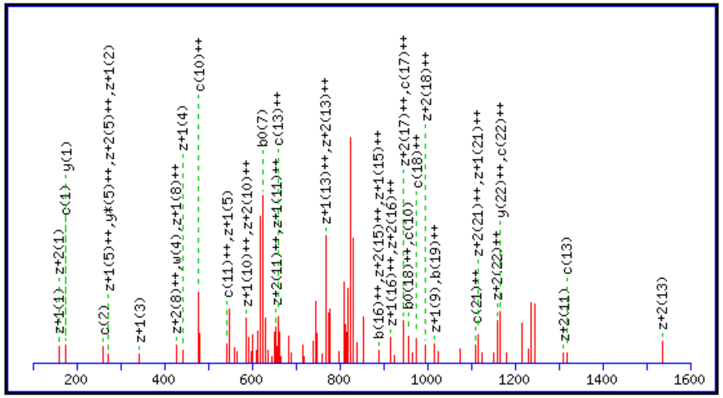

#	b	b^{++}	b^{*}	b^{*++}	b^{0}	b^{0++}	c	c^{++}	Seq.	w	w'	y	y^{++}	y^{*}	y^{*++}	y^{0}	y^{0++}	z+1	$z+1^{++}$	z+2	$z+2^{++}$	#
1	157.1335	79.0704	140.1070	70.5571			**174.1601**	87.5837	K													23
2	244.1656	122.5864	227.1390	114.0731	226.1550	113.5811	**261.1921**	131.0997	S	2300.3098		2333.3313	1167.1693	2316.3047	1158.6560	2315.3207	1158.1640	2317.3126	1159.1599	2318.3204	***1159.6638***	22
3	315.2027	158.1050	298.1761	149.5917	297.1921	149.0997	332.2292	166.6183	A			2246.2993	1123.6533	2229.2727	1115.1400	2228.2887	1114.6480	2230.2805	**1115.6439**	2231.2884	***1116.1478***	21
4	412.2554	206.6314	395.2289	198.1181	394.2449	197.6261	429.2820	215.1446	P	2132.2200		2175.2622	1088.1347	2158.2356	1079.6214	2157.2516	1079.1294	2159.2434	1080.1254	2160.2513	1080.6293	20
5	483.2926	242.1499	466.2660	233.6366	465.2820	233.1446	500.3191	250.6632	A			2078.2094	1039.6083	2061.1828	1031.0951	2060.1988	1030.6030	2062.1907	1031.5990	2063.1985	1032.1029	19
6	584.3402	292.6738	567.3137	284.1605	566.3297	283.6685	601.3668	301.1870	T	1974.1508	1976.1301	2007.1723	1004.0898	1990.1457	995.5765	1989.1617	995.0845	1991.1536	996.0804	1992.1614	***996.5843***	18
7	641.3617	321.1845	624.3352	312.6712	623.3511	312.1792	658.3883	329.6978	G			1906.1246	953.5659	1889.0980	945.0527	1888.1140	944.5607	1890.1059	945.5566	1891.1137	***946.0605***	17
8	698.3832	349.6952	681.3566	341.1819	680.3726	340.6899	715.4097	358.2085	G			1849.1031	925.0552	1832.0766	916.5419	1831.0926	916.0499	1833.0844	**917.0458**	1834.0922	***917.5498***	16
9	797.4516	399.2294	780.4250	390.7162	779.4410	390.2241	814.4781	407.7427	V	1761.0395		1792.0817	896.5445	1775.0551	888.0312	1774.0711	887.5392	1776.0629	**888.5351**	1777.0708	***889.0390***	15
10	939.5622	470.2847	922.5356	461.7715	921.5516	461.2795	**956.5887**	**478.7980**	K	1604.9132		1693.0133	847.0103	1675.9867	838.4970	1675.0027	838.0050	1676.9945	839.0009	1678.0024	839.5048	14
11	1067.6572	534.3322	1050.6306	525.8189	1049.6466	525.3269	1084.6837	**542.8455**	K	1476.8182		1550.9026	775.9550	1533.8761	767.4417	1532.8921	766.9497	1534.8839	**767.9456**	**1535.8917**	***768.4495***	13
12	1164.7099	582.8586	1147.6834	574.3453	1146.6994	573.8533	1181.7365	591.3719	P	1379.7655		1422.8077	711.9075	1405.7811	703.3942	1404.7971	702.9022	1406.7890	703.8981	1407.7968	704.4020	12
13	1301.7688	651.3881	1284.7423	642.8748	1283.7583	642.3828	**1318.7954**	**659.9013**	H			1325.7549	663.3811	1308.7284	654.8678	1307.7443	654.3758	1309.7362	**655.3717**	**1310.7440**	***655.8756***	11
14	1457.8699	729.4386	1440.8434	720.9253	1439.8594	720.4333	1474.8965	737.9519	R	1086.6055		1188.6960	594.8516	1171.6695	586.3384	1170.6854	585.8464	1172.6773	**586.8423**	1173.6851	***587.3462***	10
15	1620.9333	810.9703	1603.9067	802.4570	1602.9227	801.9650	1637.9598	819.4835	Y			1032.5949	516.8011	1015.5683	508.2878	1014.5843	507.7958	**1016.5762**	508.7917	1017.5840	509.2956	9
16	1777.0344	889.0208	1760.0078	880.5076	1759.0238	880.0155	1794.0609	897.5341	R	767.4410		869.5316	435.2694	852.5050	426.7561	851.5210	426.2641	853.5128	**427.2601**	854.5207	***427.7640***	8
17	1874.0871	937.5472	1857.0606	929.0339	1856.0766	928.5419	1891.1137	**946.0605**	P	670.3883		713.4305	357.2189	696.4039	348.7056	695.4199	348.2136	697.4117	349.2095	698.4196	349.7134	7
18	1931.1086	966.0579	1914.0821	957.5447	1913.0980	957.0527	1948.1352	**974.5712**	G			616.3777	308.6925	599.3511	300.1792	598.3671	299.6872	600.3590	300.6831	601.3668	301.1870	6
19	2032.1563	1016.5818	2015.1297	1008.0685	2014.1457	1007.5765	2049.1828	1025.0951	T	526.3348	528.3140	559.3562	280.1817	542.3297	271.6685	541.3457	271.1765	**543.3375**	**272.1724**	544.3453	***272.6763***	5
20	2131.2247	1066.1160	2114.1982	1057.6027	2113.2141	1057.1107	2148.2513	1074.6293	V	427.2663		458.3085	229.6579	441.2820	221.1446			**442.2898**	221.6485	443.2976	222.1525	4
21	2202.2618	1101.6345	2185.2353	1093.1213	2184.2513	1092.6293	2219.2884	**1110.1478**	A			359.2401	180.1237	342.2136	171.6104			**343.2214**	172.1143	344.2292	172.6183	3
22	2315.3459	1158.1766	2298.3193	1149.6633	2297.3353	1149.1713	2332.3724	**1166.6899**	L	229.1295		288.2030	144.6051	271.1765	136.0919			**272.1843**	136.5958	273.1921	137.0997	2
23									R	73.0284		175.1190	88.0631	158.0924	79.5498			**159.1002**	80.0538	**160.1081**	80.5577	1

Monoisotopic mass of neutral peptide Mr(calc): 2488.4503
Fixed modifications: Carbamidomethyl (C) (apply to specified residues or termini only)
Variable modifications:
K1 : Dimethyl_KR (KR)
K10 : Methyl_KR (KR)
Ions Score: 56 **Expect:** 0.00099
Matches : 47/402 fragment ions using 51 most intense peaks (help)

Mascot Search Results: Peptide View

Kme2SAPATGGVKme1KPHRYRPGTVALR

MS/MS Fragmentation of KSAPATGGVKKPHRYRPGTVALR
Found in sp|P68433|H31_MOUSE in fgcz_10090, Histone H3.1 OS=Mus musculus GN=Hist1h3a

Match to Query 3958: 2474.434336 from(619.615860,4+) intensity(225884.6300) scans(3134) rtinseconds(1935.5212) index(1903)
Title: 1904: Scan 3134 (rt=32.2587) [s:\p749\Proteomics\ORBI_2\andreamizzi_20110517_sperm_trypsin_GluC_ETD_CID\20110519_02_NaCl_ziptipped_2.RAW]
Data file 20110519_02_NaCl_ziptipped_2.mgf

Click mouse within plot area to zoom in by factor of two about that point
Or, Plot from 200 to 2000 Da Full range
Label all possible matches ○ Label matches used for scoring ◉
Show Y-axis ☐

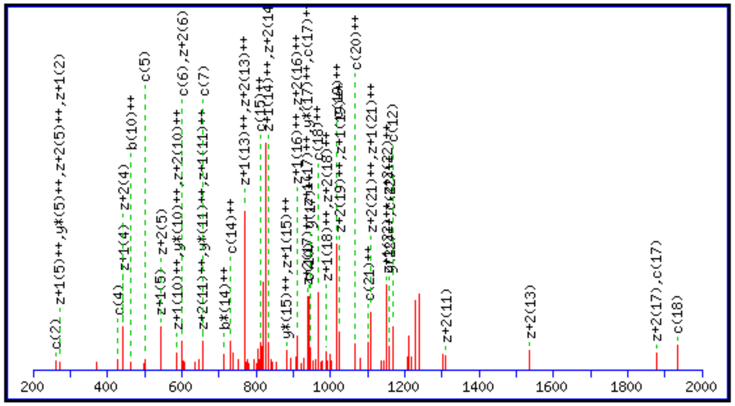

#	b	b^{++}	b^{*}	b^{*++}	b^{0}	b^{0++}	c	c^{++}	Seq.	w	w'	y	y^{++}	y^{*}	y^{*++}	y^{0}	y^{0++}	z+1	$z+1^{++}$	z+2	$z+2^{++}$	#
1	157.1335	79.0704	140.1070	70.5571			174.1601	87.5837	K													23
2	244.1656	122.5864	227.1390	114.0731	226.1550	113.5811	**261.1921**	131.0997	S	2286.2942		2319.3156	1160.1615	2302.2891	1151.6482	2301.3051	1151.1562	2303.2969	***1152.1521***	2304.3047	**1152.6560**	22
3	315.2027	158.1050	298.1761	149.5917	297.1921	149.0997	332.2292	166.6183	A			2232.2836	1116.6454	2215.2571	1108.1322	2214.2731	1107.6402	2216.2649	***1108.6361***	2217.2727	**1109.1400**	21
4	412.2554	206.6314	395.2289	198.1181	394.2449	197.6261	**429.2820**	215.1446	P	2118.2043		2161.2465	1081.1269	2144.2200	1072.6136	2143.2359	1072.1216	2145.2278	1073.1175	2146.2356	1073.6214	20
5	483.2926	242.1499	466.2660	233.6366	465.2820	233.1446	**500.3191**	250.6632	A			2064.1937	1032.6005	2047.1672	1024.0872	2046.1832	1023.5952	2048.1750	***1024.5911***	2049.1828	**1025.0951**	19
6	584.3402	292.6738	567.3137	284.1605	566.3297	283.6685	**601.3668**	301.1870	T	1960.1352	1962.1144	1993.1566	997.0820	1976.1301	988.5687	1975.1461	988.0767	1977.1379	***989.0726***	1978.1457	**989.5765**	18
7	641.3617	321.1845	624.3352	312.6712	623.3511	312.1792	**658.3883**	329.6978	G			1892.1089	946.5581	1875.0824	938.0448	1874.0984	937.5528	1876.0902	***938.5487***	**1877.0980**	**939.0527**	17
8	698.3832	349.6952	681.3566	341.1819	680.3726	340.6899	715.4097	358.2085	G			1835.0875	918.0474	1818.0609	909.5341	1817.0769	909.0421	1819.0688	***910.0380***	1820.0766	**910.5419**	16
9	797.4516	399.2294	780.4250	390.7162	779.4410	390.2241	814.4781	407.7427	V	1747.0238		1778.0660	889.5366	1761.0395	881.0234	1760.0555	880.5314	1762.0473	***881.5273***	1763.0551	882.0312	15
10	925.5465	463.2769	908.5200	454.7636	907.5360	454.2716	**942.5731**	471.7902	K	1604.9132		1678.9976	840.0024	1661.9711	831.4892	1660.9870	830.9972	1662.9789	***831.9931***	1663.9867	**832.4970**	14
11	1053.6415	527.3244	1036.6150	518.8111	1035.6309	518.3191	1070.6681	535.8377	K	1476.8182		1550.9026	775.9550	1533.8761	767.4417	1532.8921	766.9497	1534.8839	***767.9456***	**1535.8917**	**768.4495**	13
12	1150.6943	575.8508	1133.6677	567.3375	1132.6837	566.8455	**1167.7208**	584.3640	P	1379.7655		1422.8077	711.9075	1405.7811	703.3942	1404.7971	702.9022	1406.7890	703.8981	1407.7968	704.4020	12
13	1287.7532	644.3802	1270.7266	635.8670	1269.7426	635.3749	1304.7797	652.8935	H			1325.7549	663.3811	1308.7284	654.8678	1307.7443	654.3758	1309.7362	***655.3717***	**1310.7440**	**655.8756**	11
14	1443.8543	722.4308	1426.8277	713.9175	1425.8437	713.4255	1460.8808	***730.9441***	R	1086.6055		1188.6960	594.8516	1171.6695	586.3384	1170.6854	585.8464	1172.6773	***586.8423***	1173.6851	**587.3462**	10
15	1606.9176	803.9624	1589.8911	795.4492	1588.9071	794.9572	1623.9442	***812.4757***	Y			1032.5949	516.8011	1015.5683	508.2878	1014.5843	507.7958	1016.5762	508.7917	1017.5840	509.2956	9
16	1763.0187	882.0130	1745.9922	873.4997	1745.0082	873.0077	1780.0453	890.5263	R	767.4410		869.5316	435.2694	852.5050	426.7561	851.5210	426.2641	853.5128	427.2601	854.5207	427.7640	8
17	1860.0715	930.5394	1843.0449	922.0261	1842.0609	921.5341	**1877.0980**	***939.0527***	P	670.3883		713.4305	357.2189	696.4039	348.7056	695.4199	348.2136	697.4117	349.2095	698.4196	349.7134	7
18	1917.0930	959.0501	1900.0664	950.5368	1899.0824	950.0448	**1934.1195**	***967.5634***	G			616.3777	308.6925	599.3511	300.1792	598.3671	299.6872	600.3590	300.6831	**601.3668**	301.1870	6
19	2018.1406	1009.5740	2001.1141	1001.0607	2000.1301	1000.5687	2035.1672	***1018.0872***	T	526.3348	528.3140	559.3562	280.1817	542.3297	271.6685	541.3457	271.1765	**543.3375**	***272.1724***	**544.3453**	**272.6763**	5
20	2117.2091	1059.1082	2100.1825	1050.5949	2099.1985	1050.1029	2134.2356	***1067.6214***	V	427.2663		458.3085	229.6579	441.2820	221.1446			**442.2898**	221.6485	**443.2976**	222.1525	4
21	2188.2462	1094.6267	2171.2196	1086.1134	2170.2356	1085.6214	2205.2727	***1103.1400***	A			359.2401	180.1237	342.2136	171.6104			343.2214	172.1143	344.2292	172.6183	3
22	2301.3302	1151.1688	2284.3037	1142.6555	2283.3197	1142.1635	2318.3568	***1159.6820***	L	229.1295		288.2030	144.6051	271.1765	136.0919			**272.1843**	136.5958	273.1921	137.0997	2
23									R	73.0284		175.1190	88.0631	158.0924	79.5498			159.1002	80.0538	160.1081	80.5577	1

Monoisotopic mass of neutral peptide Mr(calc): 2474.4346
Fixed modifications: Carbamidomethyl (C) (apply to specified residues or termini only)
Variable modifications:
K1 : Dimethyl_KR (KR)
Ions Score: 101 **Expect:** 1.1e-07
Matches : 58/402 fragment ions using 50 most intense peaks (help)

Mascot Search Results: Peptide View

Kme2SAPATGGVKKPHRYRPGTVALR

MS/MS Fragmentation of **KSAPSTGGVKKPHR**
Found in **sp|P84244|H33_MOUSE** in **fgcz_10090**, Histone H3.3 OS=Mus musculus GN=H3f3a

Match to Query 2374: 1504.883016 from(377.228030,4+) intensity(30619.8240) scans(2356) rtinseconds(1425.8218) index(1401)
Title: 1402: Scan 2356 (rt=23.7637) [s:\p749\Proteomics\ORBI_2\andreamizzi_20110517_sperm_trypsin_GluC_ETD_CID\20110517_01_NaCl_1.RAW]
Data file 20110517_01_NaCl_1.mgf

Click mouse within plot area to zoom in by factor of two about that point
Or, Plot from 100 to 1600 Da Full range
Label all possible matches ○ Label matches used for scoring ◉
Show Y-axis ☐

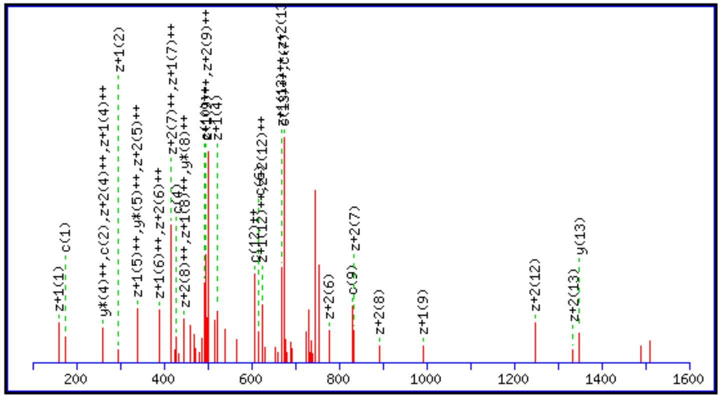

#	b	b++	b*	b*++	b0	b0++	c	c++	Seq.	w	w'	y	y++	y*	y*++	y0	y0++	z+1	z+1++	z+2	z+2++	#
1	157.1335	79.0704	140.1070	70.5571			174.1601	87.5837	K													14
2	244.1656	122.5864	227.1390	114.0731	226.1550	113.5811	261.1921	131.0997	S	1316.7433		1349.7648	675.3860	1332.7383	666.8728	1331.7542	666.3808	1333.7461	667.3767	1334.7539	667.8806	13
3	315.2027	158.1050	298.1761	149.5917	297.1921	149.0997	332.2292	166.6183	A			1262.7328	631.8700	1245.7062	623.3568	1244.7222	622.8647	1246.7141	623.8607	1247.7219	624.3646	12
4	412.2554	206.6314	395.2289	198.1181	394.2449	197.6261	429.2820	215.1446	P	1148.6535		1191.6957	596.3515	1174.6691	587.8382	1173.6851	587.3462	1175.6769	588.3421	1176.6848	588.8460	11
5	499.2875	250.1474	482.2609	241.6341	481.2769	241.1421	516.3140	258.6606	S	1061.6214		1094.6429	547.8251	1077.6164	539.3118	1076.6323	538.8198	1078.6242	539.8157	1079.6320	540.3196	10
6	600.3352	300.6712	583.3086	292.1579	582.3246	291.6659	617.3617	309.1845	T	974.5894	976.5687	1007.6109	504.3091	990.5843	495.7958	989.6003	495.3038	991.5922	496.2997	992.6000	496.8036	9
7	657.3566	329.1819	640.3301	320.6687	639.3461	320.1767	674.3832	337.6952	G			906.5632	453.7852	889.5366	445.2720			890.5445	445.7759	891.5523	446.2798	8
8	714.3781	357.6927	697.3515	349.1794	696.3675	348.6874	731.4046	366.2060	G			849.5417	425.2745	832.5152	416.7612			833.5230	417.2651	834.5308	417.7691	7
9	813.4465	407.2269	796.4199	398.7136	795.4359	398.2216	830.4730	415.7402	V	761.4781		792.5203	396.7638	775.4937	388.2505			776.5015	388.7544	777.5094	389.2583	6
10	969.5728	485.2900	952.5462	476.7767	951.5622	476.2847	986.5993	493.8033	K	591.3362		693.4519	347.2296	676.4253	338.7163			677.4331	339.2202	678.4410	339.7241	5
11	1097.6677	549.3375	1080.6412	540.8242	1079.6572	540.3322	1114.6943	557.8508	K	463.2412		537.3256	269.1664	520.2990	260.6532			521.3069	261.1571	522.3147	261.6610	4
12	1194.7205	597.8639	1177.6939	589.3506	1176.7099	588.8586	1211.7470	606.3772	P	366.1884		409.2306	205.1190	392.2041	196.6057			393.2119	197.1096	394.2197	197.6135	3
13	1331.7794	666.3933	1314.7528	657.8801	1313.7688	657.3881	1348.8059	674.9066	H			312.1779	156.5926	295.1513	148.0793			296.1591	148.5832	297.1670	149.0871	2
14									R	73.0284		175.1190	88.0631	158.0924	79.5498			159.1002	80.0538	160.1081	80.5577	1

Monoisotopic mass of neutral peptide Mr(calc): 1504.8838
Fixed modifications: Carbamidomethyl (C) (apply to specified residues or termini only)
Variable modifications:
K1 : Dimethyl_KR (KR)
K10 : Dimethyl_KR (KR)
Ions Score: 68 **Expect:** 0.00018
Matches : 38/226 fragment ions using 31 most intense peaks (help)

Mascot Search Results: Peptide View

Kme2SAPSTGGVKme2KPHR

MS/MS Fragmentation of **KSAPSTGGVKKPHRYRPGTVALR**
Found in **sp|P84244|H33_MOUSE** in **fgcz_10090**, Histone H3.3 OS=Mus musculus GN=H3f3a

Match to Query 3183: 2476.412970 from(496.289870,5+) intensity(421526.5000) scans(2942) rtinseconds(1837.045) index(1627)
Title: 1628: Scan 2942 (rt=30.6174) [s:\p749\Proteomics\ORBI_2\andreamizzi_20110310_sperm_trypsin_chymo_AspN_ETD_CID\20110310_11_NaCl_trypsin.RAW]
Data file \\fgcz-ms.uzh.ch\p749\Proteomics\ORBI_2\andreamizzi_20110310_sperm_trypsin_chymo_AspN_ETD_CID\mgf__low_res_MS2_nomerge\20110310_11_NaCl_trypsin.mgf

Click mouse within plot area to zoom in by factor of two about that point
Or, Plot from 100 to 1600 Da Full range
Label all possible matches ○ Label matches used for scoring ◉
Show Y-axis ☐

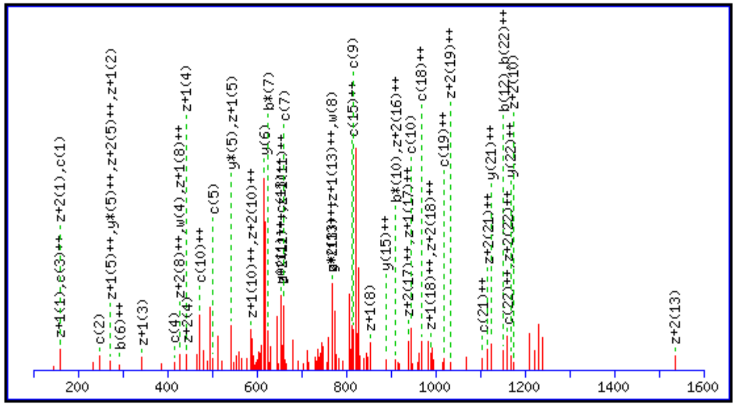

#	b	b^{++}	b^{*}	b^{*++}	b^{0}	b^{0++}	c	c^{++}	Seq.	w	w'	y	y^{++}	y^{*}	y^{*++}	y^{0}	y^{0++}	z+1	$z+1^{++}$	z+2	$z+2^{++}$	#
1	143.1179	72.0626	126.0913	63.5493			**160.1444**	80.5759	K													23
2	230.1499	115.5786	213.1234	107.0653	212.1394	106.5733	**247.1765**	124.0919	S	2302.2891		2335.3106	1168.1589	2318.2840	1159.6456	2317.3000	1159.1536	2319.2918	1160.1496	2320.2997	***1160.6535***	22
3	301.1870	151.0972	284.1605	142.5839	283.1765	142.0919	318.2136	**159.6104**	A			2248.2785	1124.6429	2231.2520	1116.1296	2230.2680	1115.6376	2232.2598	1116.6335	2233.2676	***1117.1375***	21
4	398.2398	199.6235	381.2132	191.1103	380.2292	190.6183	**415.2663**	208.1368	P	2134.1992		2177.2414	1089.1243	2160.2149	1080.6111	2159.2309	1080.1191	2161.2227	1081.1150	2162.2305	1081.6189	20
5	485.2718	243.1395	468.2453	234.6263	467.2613	234.1343	**502.2984**	251.6528	S	2047.1672		2080.1887	1040.5980	2063.1621	1032.0847	2062.1781	1031.5927	2064.1699	1032.5886	2065.1778	***1033.0925***	19
6	586.3195	293.6634	569.2930	285.1501	568.3089	284.6581	603.3461	302.1767	T	1960.1352	1962.1144	1993.1566	997.0820	1976.1301	988.5687	1975.1461	988.0767	1977.1379	**989.0726**	1978.1457	***989.5765***	18
7	643.3410	322.1741	626.3144	313.6608	625.3304	313.1688	**660.3675**	330.6874	G			1892.1089	946.5581	1875.0824	938.0448	1874.0984	937.5528	1876.0902	**938.5487**	1877.0980	***939.0527***	17
8	700.3624	350.6849	683.3359	342.1716	682.3519	341.6796	717.3890	359.1981	G			1835.0875	918.0474	1818.0609	909.5341	1817.0769	909.0421	1819.0688	910.0380	1820.0766	***910.5419***	16
9	799.4308	400.2191	782.4043	391.7058	781.4203	391.2138	**816.4574**	408.7323	V	1747.0238		1778.0660	889.5366	1761.0395	881.0234	1760.0555	880.5314	1762.0473	881.5273	1763.0551	882.0312	15
10	927.5258	464.2665	910.4993	455.7533	909.5152	455.2613	**944.5524**	**472.7798**	K	1604.9132		1678.9976	840.0024	1661.9711	831.4892	1660.9870	830.9972	1662.9789	831.9931	1663.9867	832.4970	14
11	1055.6208	528.3140	1038.5942	519.8007	1037.6102	519.3087	1072.6473	536.8273	K	1476.8182		1550.9026	775.9550	1533.8761	767.4417	1532.8921	766.9497	1534.8839	**767.9456**	**1535.8917**	***768.4495***	13
12	1152.6735	576.8404	1135.6470	568.3271	1134.6630	567.8351	1169.7001	585.3537	P	1379.7655		1422.8077	711.9075	1405.7811	703.3942	1404.7971	702.9022	1406.7890	703.8981	1407.7968	704.4020	12
13	1289.7324	645.3699	1272.7059	636.8566	1271.7219	636.3646	1306.7590	**653.8831**	H			1325.7549	663.3811	1308.7284	654.8678	1307.7443	654.3758	1309.7362	**655.3717**	1310.7440	***655.8756***	11
14	1445.8336	723.4204	1428.8070	714.9071	1427.8230	714.4151	1462.8601	731.9337	R	1086.6055		1188.6960	594.8516	1171.6695	586.3384	1170.6854	585.8464	1172.6773	**586.8423**	**1173.6851**	***587.3462***	10
15	1608.8969	804.9521	1591.8703	796.4388	1590.8863	795.9468	1625.9234	**813.4654**	Y			1032.5949	516.8011	1015.5683	508.2878	1014.5843	507.7958	1016.5762	508.7917	1017.5840	509.2956	9
16	1764.9980	883.0026	1747.9714	874.4894	1746.9874	873.9974	1782.0245	891.5159	R	767.4410		869.5316	435.2694	852.5050	426.7561	851.5210	426.2641	**853.5128**	**427.2601**	854.5207	***427.7640***	8
17	1862.0508	931.5290	1845.0242	923.0157	1844.0402	922.5237	1879.0773	940.0423	P	670.3883		713.4305	357.2189	696.4039	348.7056	695.4199	348.2136	697.4117	349.2095	698.4196	349.7134	7
18	1919.0722	960.0398	1902.0457	951.5265	1901.0617	951.0345	1936.0988	**968.5530**	G			616.3777	308.6925	599.3511	300.1792	598.3671	299.6872	600.3590	300.6831	601.3668	301.1870	6
19	2020.1199	1010.5636	2003.0934	1002.0503	2002.1093	1001.5583	2037.1465	**1019.0769**	T	526.3348	528.3140	559.3562	280.1817	542.3297	271.6685	541.3457	271.1765	**543.3375**	**272.1724**	544.3453	***272.6763***	5
20	2119.1883	1060.0978	2102.1618	1051.5845	2101.1778	1051.0925	2136.2149	1068.6111	V	427.2663		458.3085	229.6579	441.2820	221.1446			**442.2898**	221.6485	**443.2976**	222.1525	4
21	2190.2254	1095.6164	2173.1989	1087.1031	2172.2149	1086.6111	2207.2520	**1104.1296**	A			359.2401	180.1237	342.2136	171.6104			**343.2214**	172.1143	344.2292	172.6183	3
22	2303.3095	1152.1584	2286.2829	1143.6451	2285.2989	1143.1531	2320.3360	**1160.6717**	L	229.1295		288.2030	144.6051	271.1765	136.0919			**272.1843**	136.5958	273.1921	137.0997	2
23									R	73.0284		175.1190	88.0631	158.0924	79.5498			**159.1002**	80.0538	**160.1081**	80.5577	1

Monoisotopic mass of neutral peptide Mr(calc): 2476.4139
Fixed modifications: Carbamidomethyl (C) (apply to specified residues or termini only)
Variable modifications:
K1 : Methyl_KR (KR)
Ions Score: 46 **Expect:** 0.018
Matches : 58/403 fragment ions using 81 most intense peaks (help)

Mascot Search Results: Peptide View

Kme1SAPSTGGVKKPHRYRPGTVALR

MS/MS Fragmentation of **KSAPSTGGVKKPHRYRPGTVALR**
Found in **sp|P84244|H33_MOUSE** in **fgcz_10090**, Histone H3.3 OS=Mus musculus GN=H3f3a

Match to Query 5468: 2518.456720 from(504.698620,5+) intensity(182475.4700) scans(2290) rtinseconds(1874.7487) index(3765)
Title: 739: Scan 2290 (rt=31.2458) [s:\p749\Proteomics\ORBI_2\andreamizzi_20110310_sperm_trypsin_chymo_AspN_ETD_CID\20110311_34_NaCl_trypsin_SCX_FT.RAW]
Data file Y:\p749\Proteomics\Mascot\mgf\50 20110426 sperm rerun trypsin 2nd ETD run NaCl SCX\mascot_daemon_merge.mgf

Click mouse within plot area to zoom in by factor of two about that point
Or, Plot from 100 to 1600 Da Full range
Label all possible matches ○ Label matches used for scoring ◉
Show Y-axis ☐

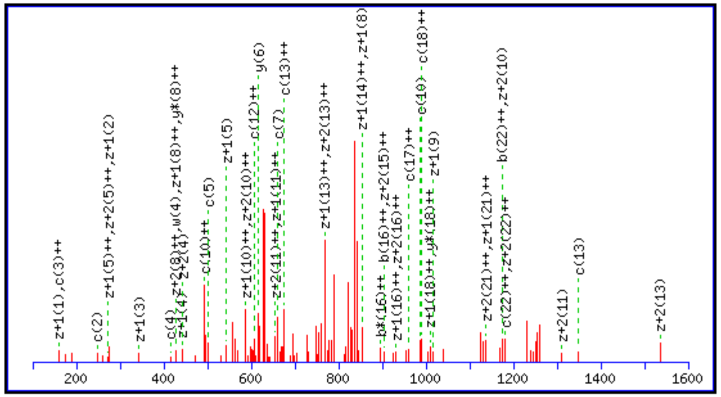

#	b	b++	b*	b*++	b0	b0++	c	c++	Seq.	w	w'	y	y++	y*	y*++	y0	y0++	z+1	z+1++	z+2	z+2++	#
1	143.1179	72.0626	126.0913	63.5493			160.1444	80.5759	K													23
2	230.1499	115.5786	213.1234	107.0653	212.1394	106.5733	**247.1765**	124.0919	S	2344.3360		2377.3575	1189.1824	2360.3310	1180.6691	2359.3469	1180.1771	2361.3388	1181.1730	2362.3466	***1181.6769***	22
3	301.1870	151.0972	284.1605	142.5839	283.1765	142.0919	318.2136	**159.6104**	A			2290.3255	1145.6664	2273.2989	1137.1531	2272.3149	1136.6611	2274.3068	***1137.6570***	2275.3146	***1138.1609***	21
4	398.2398	199.6235	381.2132	191.1103	380.2292	190.6183	**415.2663**	208.1368	P	2176.2462		2219.2884	1110.1478	2202.2618	1101.6345	2201.2778	1101.1425	2203.2696	1102.1385	2204.2775	1102.6424	20
5	485.2718	243.1395	468.2453	234.6263	467.2613	234.1343	**502.2984**	251.6528	S	2089.2141		2122.2356	1061.6214	2105.2091	1053.1082	2104.2250	1052.6162	2106.2169	1053.6121	2107.2247	1054.1160	19
6	586.3195	293.6634	569.2930	285.1501	568.3089	284.6581	603.3461	302.1767	T	2002.1821	2004.1614	2035.2036	1018.1054	2018.1770	1009.5922	2017.1930	1009.1001	2019.1849	***1010.0961***	2020.1927	1010.6000	18
7	643.3410	322.1741	626.3144	313.6608	625.3304	313.1688	**660.3675**	330.6874	G			1934.1559	967.5816	1917.1293	959.0683	1916.1453	958.5763	1918.1372	959.5722	1919.1450	960.0761	17
8	700.3624	350.6849	683.3359	342.1716	682.3519	341.6796	717.3890	359.1981	G			1877.1344	939.0709	1860.1079	930.5576	1859.1239	930.0656	1861.1157	***931.0615***	1862.1235	***931.5654***	16
9	799.4308	400.2191	782.4043	391.7058	781.4203	391.2138	816.4574	408.7323	V	1789.0708		1820.1130	910.5601	1803.0864	902.0468	1802.1024	901.5548	1804.0942	902.5508	1805.1021	***903.0547***	15
10	969.5728	485.2900	952.5462	476.7767	951.5622	476.2847	**986.5993**	**493.8033**	K	1604.9132		1721.0446	861.0259	1704.0180	852.5126	1703.0340	852.0206	1705.0258	***853.0166***	1706.0337	***853.5205***	14
11	1097.6677	549.3375	1080.6412	540.8242	1079.6572	540.3322	1114.6943	557.8508	K	1476.8182		1550.9026	775.9550	1533.8761	767.4417	1532.8921	766.9497	1534.8839	***767.9456***	**1535.8917**	***768.4495***	13
12	1194.7205	597.8639	1177.6939	589.3506	1176.7099	588.8586	1211.7470	**606.3772**	P	1379.7655		1422.8077	711.9075	1405.7811	703.3942	1404.7971	702.9022	1406.7890	703.8981	1407.7968	704.4020	12
13	1331.7794	666.3933	1314.7528	657.8801	1313.7688	657.3881	**1348.8059**	**674.9066**	H			1325.7549	663.3811	1308.7284	654.8678	1307.7443	654.3758	1309.7362	***655.3717***	**1310.7440**	***655.8756***	11
14	1487.8805	744.4439	1470.8540	735.9306	1469.8699	735.4386	1504.9071	752.9572	R	1086.6055		1188.6960	594.8516	1171.6695	586.3384	1170.6854	585.8464	1172.6773	***586.8423***	**1173.6851**	***587.3462***	10
15	1650.9438	825.9756	1633.9173	817.4623	1632.9333	816.9703	1667.9704	834.4888	Y			1032.5949	516.8011	1015.5683	508.2878	1014.5843	507.7958	***1016.5762***	508.7917	1017.5840	509.2956	9
16	1807.0449	904.0261	1790.0184	895.5128	1789.0344	895.0208	1824.0715	912.5394	R	767.4410		869.5316	435.2694	852.5050	426.7561	851.5210	426.2641	***853.5128***	***427.2601***	854.5207	***427.7640***	8
17	1904.0977	952.5525	1887.0712	944.0392	1886.0871	943.5472	1921.1243	**961.0658**	P	670.3883		713.4305	357.2189	696.4039	348.7056	695.4199	348.2136	697.4117	349.2095	698.4196	349.7134	7
18	1961.1192	981.0632	1944.0926	972.5500	1943.1086	972.0579	1978.1457	**989.5765**	G			616.3777	308.6925	599.3511	300.1792	598.3671	299.6872	600.3590	300.6831	601.3668	301.1870	6
19	2062.1669	1031.5871	2045.1403	1023.0738	2044.1563	1022.5818	2079.1934	1040.1003	T	526.3348	528.3140	559.3562	280.1817	542.3297	271.6685	541.3457	271.1765	***543.3375***	***272.1724***	544.3453	***272.6763***	5
20	2161.2353	1081.1213	2144.2087	1072.6080	2143.2247	1072.1160	2178.2618	1089.6345	V	427.2663		458.3085	229.6579	441.2820	221.1446			***442.2898***	221.6485	**443.2976**	222.1525	4
21	2232.2724	1116.6398	2215.2458	1108.1266	2214.2618	1107.6345	2249.2989	1125.1531	A			359.2401	180.1237	342.2136	171.6104			***343.2214***	172.1143	344.2292	172.6183	3
22	2345.3564	1173.1819	2328.3299	1164.6686	2327.3459	1164.1766	2362.3830	**1181.6951**	L	229.1295		288.2030	144.6051	271.1765	136.0919			***272.1843***	136.5958	273.1921	137.0997	2
23									R	73.0284		175.1190	88.0631	158.0924	79.5498			***159.1002***	80.0538	160.1081	80.5577	1

Monoisotopic mass of neutral peptide Mr(calc): 2518.4608
Fixed modifications: Carbamidomethyl (C) (apply to specified residues or termini only)
Variable modifications:
K1 : Methyl_KR (KR)
K10 : Trimethyl (K)
Ions Score: 46 **Expect:** 0.014
Matches : 50/403 fragment ions using 67 most intense peaks (help)

Mascot Search Results: Peptide View

Kme1SAPSTGGVKme3KPHRYRPGTVALR

MS/MS Fragmentation of KSAPSTGGVKKPHRYRPGTVALR
Found in sp|P84244|H33_MOUSE in fgcz_10090, Histone H3.3 OS=Mus musculus GN=H3f3a

Match to Query 3918: 2504.439020 from(501.895080,5+) intensity(434500.2200) scans(3361) rtinseconds(1964.7743) index(2090)
Title: 2091: Scan 3361 (rt=32.7462) [s:\p749\Proteomics\ORBI_2\andreamizzi_20110517_sperm_trypsin_GluC_ETD_CID\20110517_01_NaCl_1.RAW]
Data file 20110517_01_NaCl_1.mgf

Click mouse within plot area to zoom in by factor of two about that point
Or, Plot from 100 to 1600 Da Full range
Label all possible matches ○ Label matches used for scoring ◉
Show Y-axis ☐

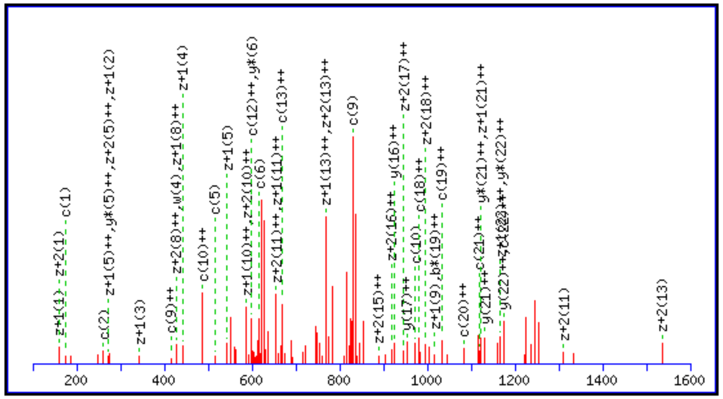

#	b	b^{++}	b^*	b^{*++}	b^0	b^{0++}	c	c^{++}	Seq.	w	w'	y	y^{++}	y^*	y^{*++}	y^0	y^{0++}	z+1	$z+1^{++}$	z+2	$z+2^{++}$	#
1	157.1335	79.0704	140.1070	70.5571			**174.1601**	87.5837	K													23
2	244.1656	122.5864	227.1390	114.0731	226.1550	113.5811	**261.1921**	131.0997	S	2316.3047		2349.3262	1175.1667	2332.2997	1166.6535	2331.3156	1166.1615	2333.3075	***1167.1574***	2334.3153	1167.6613	22
3	315.2027	158.1050	298.1761	149.5917	297.1921	149.0997	332.2292	166.6183	A			2262.2942	1131.6507	2245.2676	1123.1375	2244.2836	1122.6454	2246.2755	***1123.6414***	2247.2833	1124.1453	21
4	412.2554	206.6314	395.2289	198.1181	394.2449	197.6261	429.2820	215.1446	P	2148.2149		2191.2571	1096.1322	2174.2305	1087.6189	2173.2465	1087.1269	2175.2383	1088.1228	2176.2462	1088.6267	20
5	499.2875	250.1474	482.2609	241.6341	481.2769	241.1421	**516.3140**	258.6606	S	2061.1828		2094.2043	1047.6058	2077.1778	1039.0925	2076.1937	1038.6005	2078.1856	1039.5964	2079.1934	1040.1003	19
6	600.3352	300.6712	583.3086	292.1579	582.3246	291.6659	**617.3617**	309.1845	T	1974.1508	1976.1301	2007.1723	1004.0898	1990.1457	995.5765	1989.1617	995.0845	1991.1536	996.0804	1992.1614	***996.5843***	18
7	657.3566	329.1819	640.3301	320.6687	639.3461	320.1767	674.3832	337.6952	G			1906.1246	953.5659	1889.0980	945.0527	1888.1140	944.5607	1890.1059	945.5566	1891.1137	***946.0605***	17
8	714.3781	357.6927	697.3515	349.1794	696.3675	348.6874	731.4046	366.2060	G			1849.1031	925.0552	1832.0766	916.5419	1831.0926	916.0499	1833.0844	917.0458	1834.0922	***917.5498***	16
9	813.4465	407.2269	796.4199	398.7136	795.4359	398.2216	**830.4730**	***415.7402***	V	1761.0395		1792.0817	896.5445	1775.0551	888.0312	1774.0711	887.5392	1776.0629	888.5351	1777.0708	***889.0390***	15
10	955.5571	478.2822	938.5306	469.7689	937.5465	469.2769	**972.5837**	***486.7955***	K	1604.9132		1693.0133	847.0103	1675.9867	838.4970	1675.0027	838.0050	1676.9945	839.0009	1678.0024	839.5048	14
11	1083.6521	542.3297	1066.6255	533.8164	1065.6415	533.3244	1100.6786	550.8429	K	1476.8182		1550.9026	775.9550	1533.8761	767.4417	1532.8921	766.9497	1534.8839	***767.9456***	**1535.8917**	***768.4495***	13
12	1180.7048	590.8561	1163.6783	582.3428	1162.6943	581.8508	1197.7314	***599.3693***	P	1379.7655		1422.8077	711.9075	1405.7811	703.3942	1404.7971	702.9022	1406.7890	703.8981	1407.7968	704.4020	12
13	1317.7637	659.3855	1300.7372	650.8722	1299.7532	650.3802	1334.7903	***667.8988***	H			1325.7549	663.3811	1308.7284	654.8678	1307.7443	654.3758	1309.7362	***655.3717***	**1310.7440**	***655.8756***	11
14	1473.8649	737.4361	1456.8383	728.9228	1455.8543	728.4308	1490.8914	745.9493	R	1086.6055		1188.6960	594.8516	1171.6695	586.3384	1170.6854	585.8464	1172.6773	***586.8423***	1173.6851	***587.3462***	10
15	1636.9282	818.9677	1619.9016	810.4545	1618.9176	809.9624	1653.9547	827.4810	Y			1032.5949	516.8011	1015.5683	508.2878	1014.5843	507.7958	**1016.5762**	508.7917	1017.5840	509.2956	9
16	1793.0293	897.0183	1776.0027	888.5050	1775.0187	888.0130	1810.0558	905.5316	R	767.4410		869.5316	435.2694	852.5050	426.7561	851.5210	426.2641	853.5128	***427.2601***	854.5207	***427.7640***	8
17	1890.0821	945.5447	1873.0555	937.0314	1872.0715	936.5394	1907.1086	954.0579	P	670.3883		713.4305	357.2189	696.4039	348.7056	695.4199	348.2136	697.4117	349.2095	698.4196	349.7134	7
18	1947.1035	974.0554	1930.0770	965.5421	1929.0930	965.0501	1964.1301	***982.5687***	G			616.3777	308.6925	599.3511	300.1792	598.3671	299.6872	600.3590	300.6831	601.3668	301.1870	6
19	2048.1512	1024.5792	2031.1247	1016.0660	2030.1406	1015.5740	2065.1778	***1033.0925***	T	526.3348	528.3140	559.3562	280.1817	542.3297	271.6685	541.3457	271.1765	**543.3375**	***272.1724***	544.3453	***272.6763***	5
20	2147.2196	1074.1134	2130.1931	1065.6002	2129.2091	1065.1082	2164.2462	***1082.6267***	V	427.2663		458.3085	229.6579	441.2820	221.1446			**442.2898**	221.6485	443.2976	222.1525	4
21	2218.2567	1109.6320	2201.2302	1101.1187	2200.2462	1100.6267	2235.2833	***1118.1453***	A			359.2401	180.1237	342.2136	171.6104			**343.2214**	172.1143	344.2292	172.6183	3
22	2331.3408	1166.1740	2314.3142	1157.6608	2313.3302	1157.1688	2348.3673	***1174.6873***	L	229.1295		288.2030	144.6051	271.1765	136.0919			**272.1843**	136.5958	273.1921	137.0997	2
23									R	73.0284		175.1190	88.0631	158.0924	79.5498			**159.1002**	80.0538	**160.1081**	80.5577	1

Monoisotopic mass of neutral peptide Mr(calc): 2504.4452
Fixed modifications: Carbamidomethyl (C) (apply to specified residues or termini only)
Variable modifications:
K1 : Dimethyl_KR (KR)
K10 : Methyl_KR (KR)
Ions Score: 53 **Expect:** 0.0015
Matches : 50/403 fragment ions using 67 most intense peaks (help)

Mascot Search Results: Peptide View

Kme2SAPSTGGVKme1KPHRYRPGTVALR

MS/MS Fragmentation of KSAPSTGGVKKPHRYRPGTVALR
Found in sp|P84244|H33_MOUSE in fgcz_10090, Histone H3.3 OS=Mus musculus GN=H3f3a

Match to Query 3695: 2518.459470 from(504.699170,5+) intensity(204046.8900) scans(3054) rtinseconds(1973.2304) index(1851)
Title: 1852: Scan 3054 (rt=32.8872) [s:\p749\Proteomics\ORBI_2\andreamizzi_20110517_sperm_trypsin_GluC_ETD_CID\20110519_01_NaCl_ziptipped_1.RAW]
Data file 20110519_01_NaCl_ziptipped_1.mgf

Click mouse within plot area to zoom in by factor of two about that point
Or, Plot from 100 to 1600 Da Full range
Label all possible matches ○ Label matches used for scoring ◉
Show Y-axis ☐

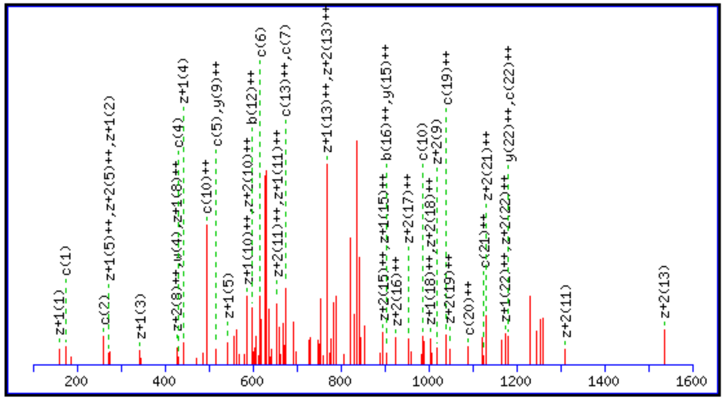

#	b	b^{++}	b^{*}	b^{*++}	b^{0}	b^{0++}	c	c^{++}	Seq.	w	w'	y	y^{++}	y^{*}	y^{*++}	y^{0}	y^{0++}	z+1	$z+1^{++}$	z+2	$z+2^{++}$	#
1	157.1335	79.0704	140.1070	70.5571			174.1601	87.5837	K													23
2	244.1656	122.5864	227.1390	114.0731	226.1550	113.5811	261.1921	131.0997	S	2330.3204		2363.3419	1182.1746	2346.3153	1173.6613	2345.3313	1173.1693	2347.3231	1174.1652	2348.3310	1174.6691	22
3	315.2027	158.1050	298.1761	149.5917	297.1921	149.0997	332.2292	166.6183	A			2276.3098	1138.6586	2259.2833	1130.1453	2258.2993	1129.6533	2260.2911	1130.6492	2261.2989	1131.1531	21
4	412.2554	206.6314	395.2289	198.1181	394.2449	197.6261	429.2820	215.1446	P	2162.2305		2205.2727	1103.1400	2188.2462	1094.6267	2187.2622	1094.1347	2189.2540	1095.1306	2190.2618	1095.6345	20
5	499.2875	250.1474	482.2609	241.6341	481.2769	241.1421	516.3140	258.6606	S	2075.1985		2108.2200	1054.6136	2091.1934	1046.1003	2090.2094	1045.6083	2092.2012	1046.6043	2093.2091	1047.1082	19
6	600.3352	300.6712	583.3086	292.1579	582.3246	291.6659	617.3617	309.1845	T	1988.1665	1990.1457	2021.1879	1011.0976	2004.1614	1002.5843	2003.1774	1002.0923	2005.1692	1003.0882	2006.1770	1003.5922	18
7	657.3566	329.1819	640.3301	320.6687	639.3461	320.1767	674.3832	337.6952	G			1920.1402	960.5738	1903.1137	952.0605	1902.1297	951.5685	1904.1215	952.5644	1905.1293	953.0683	17
8	714.3781	357.6927	697.3515	349.1794	696.3675	348.6874	731.4046	366.2060	G			1863.1188	932.0630	1846.0922	923.5498	1845.1082	923.0577	1847.1001	924.0537	1848.1079	924.5576	16
9	813.4465	407.2269	796.4199	398.7136	795.4359	398.2216	830.4730	415.7402	V	1775.0551		1806.0973	903.5523	1789.0708	895.0390	1788.0868	894.5470	1790.0786	895.5429	1791.0864	896.0468	15
10	969.5728	485.2900	952.5462	476.7767	951.5622	476.2847	986.5993	493.8033	K	1604.9132		1707.0289	854.0181	1690.0024	845.5048	1689.0183	845.0128	1691.0102	846.0087	1692.0180	846.5126	14
11	1097.6677	549.3375	1080.6412	540.8242	1079.6572	540.3322	1114.6943	557.8508	K	1476.8182		1550.9026	775.9550	1533.8761	767.4417	1532.8921	766.9497	1534.8839	767.9456	1535.8917	768.4495	13
12	1194.7205	597.8639	1177.6939	589.3506	1176.7099	588.8586	1211.7470	606.3772	P	1379.7655		1422.8077	711.9075	1405.7811	703.3942	1404.7971	702.9022	1406.7890	703.8981	1407.7968	704.4020	12
13	1331.7794	666.3933	1314.7528	657.8801	1313.7688	657.3881	1348.8059	674.9066	H			1325.7549	663.3811	1308.7284	654.8678	1307.7443	654.3758	1309.7362	655.3717	1310.7440	655.8756	11
14	1487.8805	744.4439	1470.8540	735.9306	1469.8699	735.4386	1504.9071	752.9572	R	1086.6055		1188.6960	594.8516	1171.6695	586.3384	1170.6854	585.8464	1172.6773	586.8423	1173.6851	587.3462	10
15	1650.9438	825.9756	1633.9173	817.4623	1632.9333	816.9703	1667.9704	834.4888	Y			1032.5949	516.8011	1015.5683	508.2878	1014.5843	507.7958	1016.5762	508.7917	1017.5840	509.2956	9
16	1807.0449	904.0261	1790.0184	895.5128	1789.0344	895.0208	1824.0715	912.5394	R	767.4410		869.5316	435.2694	852.5050	426.7561	851.5210	426.2641	853.5128	427.2601	854.5207	427.7640	8
17	1904.0977	952.5525	1887.0712	944.0392	1886.0871	943.5472	1921.1243	961.0658	P	670.3883		713.4305	357.2189	696.4039	348.7056	695.4199	348.2136	697.4117	349.2095	698.4196	349.7134	7
18	1961.1192	981.0632	1944.0926	972.5500	1943.1086	972.0579	1978.1457	989.5765	G			616.3777	308.6925	599.3511	300.1792	598.3671	299.6872	600.3590	300.6831	601.3668	301.1870	6
19	2062.1669	1031.5871	2045.1403	1023.0738	2044.1563	1022.5818	2079.1934	1040.1003	T	526.3348	528.3140	559.3562	280.1817	542.3297	271.6685	541.3457	271.1765	543.3375	272.1724	544.3453	272.6763	5
20	2161.2353	1081.1213	2144.2087	1072.6080	2143.2247	1072.1160	2178.2618	1089.6345	V	427.2663		458.3085	229.6579	441.2820	221.1446			442.2898	221.6485	443.2976	222.1525	4
21	2232.2724	1116.6398	2215.2458	1108.1266	2214.2618	1107.6345	2249.2989	1125.1531	A			359.2401	180.1237	342.2136	171.6104			343.2214	172.1143	344.2292	172.6183	3
22	2345.3564	1173.1819	2328.3299	1164.6686	2327.3459	1164.1766	2362.3830	1181.6951	L	229.1295		288.2030	144.6051	271.1765	136.0919			272.1843	136.5958	273.1921	137.0997	2
23									R	73.0284		175.1190	88.0631	158.0924	79.5498			159.1002	80.0538	160.1081	80.5577	1

Monoisotopic mass of neutral peptide Mr(calc): 2518.4608
Fixed modifications: Carbamidomethyl (C) (apply to specified residues or termini only)
Variable modifications:
K1 : Dimethyl_KR (KR)
K10 : Dimethyl_KR (KR)
Ions Score: 62 **Expect:** 0.0029
Matches : 47/403 fragment ions using 61 most intense peaks (help)

Mascot Search Results: Peptide View

Kme2SAPSTGGVKme2KPHRYRPGTVALR

MS/MS Fragmentation of KSAPSTGGVKKPHRYRPGTVALR
Found in sp|P84244|H33_MOUSE in fgcz_10090, Histone H3.3 OS=Mus musculus GN=H3f3a

Match to Query 4067: 2532.474270 from(507.502130,5+) intensity(251277.0500) scans(3140) rtinseconds(1938.1316) index(1908)
Title: 1909: Scan 3140 (rt=32.3022) [s:\p749\Proteomics\ORBI_2\andreamizzi_20110517_sperm_trypsin_GluC_ETD_CID\20110519_02_NaCl_ziptipped_2.RAW]
Data file 20110519_02_NaCl_ziptipped_2.mgf

Click mouse within plot area to zoom in by factor of two about that point
Or, Plot from 100 to 1600 Da Full range
Label all possible matches ○ Label matches used for scoring ◉
Show Y-axis ☐

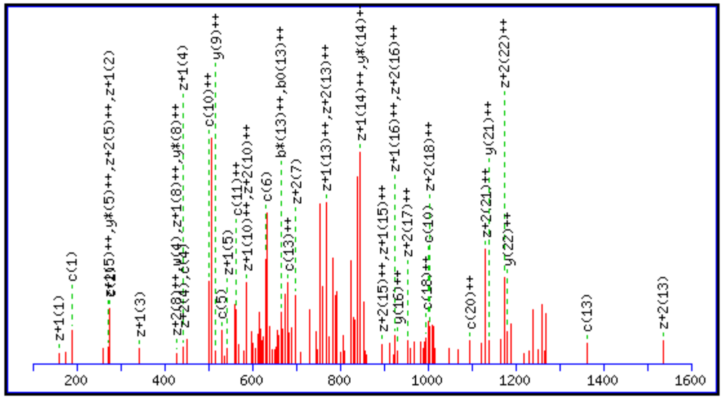

#	b	b^{++}	b^{*}	b^{*++}	b^{0}	b^{0++}	c	c^{++}	Seq.	w	w'	y	y^{++}	y^{*}	y^{*++}	y^{0}	y^{0++}	z+1	$z+1^{++}$	z+2	$z+2^{++}$	#
1	171.1492	86.0782	154.1226	77.5650			188.1757	94.5915	K													23
2	258.1812	129.5942	241.1547	121.0810	240.1707	120.5890	275.2078	138.1075	S	2330.3204		2363.3419	1182.1746	2346.3153	1173.6613	2345.3313	1173.1693	2347.3231	1174.1652	2348.3310	1174.6691	22
3	329.2183	165.1128	312.1918	156.5995	311.2078	156.1075	346.2449	173.6261	A			2276.3098	1138.6586	2259.2833	1130.1453	2258.2993	1129.6533	2260.2911	1130.6492	2261.2989	1131.1531	21
4	426.2711	213.6392	409.2445	205.1259	408.2605	204.6339	443.2976	222.1525	P	2162.2305		2205.2727	1103.1400	2188.2462	1094.6267	2187.2622	1094.1347	2189.2540	1095.1306	2190.2618	1095.6345	20
5	513.3031	257.1552	496.2766	248.6419	495.2926	248.1499	530.3297	265.6685	S	2075.1985		2108.2200	1054.6136	2091.1934	1046.1003	2090.2094	1045.6083	2092.2012	1046.6043	2093.2091	1047.1082	19
6	614.3508	307.6790	597.3243	299.1658	596.3402	298.6738	631.3774	316.1923	T	1988.1665	1990.1457	2021.1879	1011.0976	2004.1614	1002.5843	2003.1774	1002.0923	2005.1692	1003.0882	2006.1770	1003.5922	18
7	671.3723	336.1898	654.3457	327.6765	653.3617	327.1845	688.3988	344.7030	G			1920.1402	960.5738	1903.1137	952.0605	1902.1297	951.5685	1904.1215	952.5644	1905.1293	953.0683	17
8	728.3937	364.7005	711.3672	356.1872	710.3832	355.6952	745.4203	373.2138	G			1863.1188	932.0630	1846.0922	923.5498	1845.1082	923.0577	1847.1001	924.0537	1848.1079	924.5576	16
9	827.4621	414.2347	810.4356	405.7214	809.4516	405.2294	844.4887	422.7480	V	1775.0551		1806.0973	903.5523	1789.0708	895.0390	1788.0868	894.5470	1790.0786	895.5429	1791.0864	896.0468	15
10	983.5884	492.2978	966.5619	483.7846	965.5778	483.2926	1000.6150	500.8111	K	1604.9132		1707.0289	854.0181	1690.0024	845.5048	1689.0183	845.0128	1691.0102	846.0087	1692.0180	846.5126	14
11	1111.6834	556.3453	1094.6568	547.8320	1093.6728	547.3400	1128.7099	564.8586	K	1476.8182		1550.9026	775.9550	1533.8761	767.4417	1532.8921	766.9497	1534.8839	767.9456	1535.8917	768.4495	13
12	1208.7361	604.8717	1191.7096	596.3584	1190.7256	595.8664	1225.7627	613.3850	P	1379.7655		1422.8077	711.9075	1405.7811	703.3942	1404.7971	702.9022	1406.7890	703.8981	1407.7968	704.4020	12
13	1345.7950	673.4012	1328.7685	664.8879	1327.7845	664.3959	1362.8216	681.9144	H			1325.7549	663.3811	1308.7284	654.8678	1307.7443	654.3758	1309.7362	655.3717	1310.7440	655.8756	11
14	1501.8962	751.4517	1484.8696	742.9384	1483.8856	742.4464	1518.9227	759.9650	R	1086.6055		1188.6960	594.8516	1171.6695	586.3384	1170.6854	585.8464	1172.6773	586.8423	1173.6851	587.3462	10
15	1664.9595	832.9834	1647.9329	824.4701	1646.9489	823.9781	1681.9860	841.4967	Y			1032.5949	516.8011	1015.5683	508.2878	1014.5843	507.7958	1016.5762	508.7917	1017.5840	509.2956	9
16	1821.0606	911.0339	1804.0340	902.5207	1803.0500	902.0287	1838.0871	919.5472	R	767.4410		869.5316	435.2694	852.5050	426.7561	851.5210	426.2641	853.5128	427.2601	854.5207	427.7640	8
17	1918.1134	959.5603	1901.0868	951.0470	1900.1028	950.5550	1935.1399	968.0736	P	670.3883		713.4305	357.2189	696.4039	348.7056	695.4199	348.2136	697.4117	349.2095	698.4196	349.7134	7
18	1975.1348	988.0711	1958.1083	979.5578	1957.1243	979.0658	1992.1614	996.5843	G			616.3777	308.6925	599.3511	300.1792	598.3671	299.6872	600.3590	300.6831	601.3668	301.1870	6
19	2076.1825	1038.5949	2059.1560	1030.0816	2058.1719	1029.5896	2093.2091	1047.1082	T	526.3348	528.3140	559.3562	280.1817	542.3297	271.6685	541.3457	271.1765	543.3375	272.1724	544.3453	272.6763	5
20	2175.2509	1088.1291	2158.2244	1079.6158	2157.2404	1079.1238	2192.2775	1096.6424	V	427.2663		458.3085	229.6579	441.2820	221.1446			442.2898	221.6485	443.2976	222.1525	4
21	2246.2880	1123.6477	2229.2615	1115.1344	2228.2775	1114.6424	2263.3146	1132.1609	A			359.2401	180.1237	342.2136	171.6104			343.2214	172.1143	344.2292	172.6183	3
22	2359.3721	1180.1897	2342.3455	1171.6764	2341.3615	1171.1844	2376.3986	1188.7030	L	229.1295		288.2030	144.6051	271.1765	136.0919			272.1843	136.5958	273.1921	137.0997	2
23									R	73.0284		175.1190	88.0631	158.0924	79.5498			159.1002	80.0538	160.1081	80.5577	1

Monoisotopic mass of neutral peptide Mr(calc): 2532.4765
Fixed modifications: Carbamidomethyl (C) (apply to specified residues or termini only)
Variable modifications:
K1 : Trimethyl (K)
K10 : Dimethyl_KR (KR)
Ions Score: 43 **Expect:** 0.05
Matches : 47/403 fragment ions using 64 most intense peaks (help)

Mascot Search Results: Peptide View

Kme3SAPSTGGVKme2KPHRYRPGTVALR

MS/MS Fragmentation of KSTGGKAPRKQLATK
Found in sp|P68433|H31_MOUSE in fgcz_10090, Histone H3.1 OS=Mus musculus GN=Hist1h3a

Match to Query 2583: 1611.977016 from(404.001530,4+) intensity(103038.7900) scans(2242) rtinseconds(1320.9594) index(1420)
Title: 1421: Scan 2242 (rt=22.016) [s:\p749\Proteomics\ORBI_2\andreamizzi_20110517_sperm_trypsin_GluC_ETD_CID\20110517_02_NaCl_2.RAW]
Data file 20110517_02_NaCl_2.mgf

Click mouse within plot area to zoom in by factor of two about that point
Or, Plot from 100 to 1500 Da Full range
Label all possible matches ○ Label matches used for scoring ◉
Show Y-axis ☐

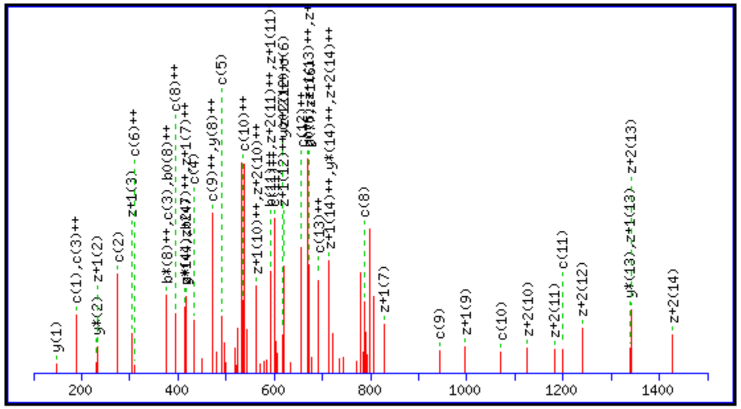

#	b	b++	b*	b*++	b0	b0++	c	c++	Seq.	w	w'	y	y++	y*	y*++	y0	y0++	z+1	z+1++	z+2	z+2++	#
1	171.1492	86.0782	154.1226	77.5650			188.1757	94.5915	K													15
2	258.1812	129.5942	241.1547	121.0810	240.1707	120.5890	275.2078	138.1075	S	1409.8223		1442.8438	721.9255	1425.8172	713.4123	1424.8332	712.9203	1426.8251	713.9162	1427.8329	714.4201	14
3	359.2289	180.1181	342.2023	171.6048	341.2183	171.1128	376.2554	188.6314	T	1322.7903	1324.7696	1355.8118	678.4095	1338.7852	669.8962	1337.8012	669.4042	1339.7930	670.4002	1340.8009	670.9041	13
4	416.2504	208.6288	399.2238	200.1155	398.2398	199.6235	433.2769	217.1421	G			1254.7641	627.8857	1237.7375	619.3724	1236.7535	618.8804	1238.7454	619.8763	1239.7532	620.3802	12
5	473.2718	237.1395	456.2453	228.6263	455.2613	228.1343	490.2984	245.6528	G			1197.7426	599.3749	1180.7161	590.8617	1179.7321	590.3697	1181.7239	591.3656	1182.7317	591.8695	11
6	601.3668	301.1870	584.3402	292.6738	583.3562	292.1817	618.3933	309.7003	K	1066.6368		1140.7212	570.8642	1123.6946	562.3509	1122.7106	561.8589	1124.7024	562.8549	1125.7103	563.3588	10
7	672.4039	336.7056	655.3774	328.1923	654.3933	327.7003	689.4305	345.2189	A			1012.6262	506.8167	995.5996	498.3035	994.6156	497.8115	996.6075	498.8074	997.6153	499.3113	9
8	769.4567	385.2320	752.4301	376.7187	751.4461	376.2267	786.4832	393.7452	P	898.5469		941.5891	471.2982	924.5625	462.7849	923.5785	462.2929	925.5704	463.2888	926.5782	463.7927	8
9	925.5578	463.2825	908.5312	454.7693	907.5472	454.2772	942.5843	471.7958	R	742.4458		844.5363	422.7718	827.5098	414.2585	826.5257	413.7665	828.5176	414.7624	829.5254	415.2663	7
10	1053.6527	527.3300	1036.6262	518.8167	1035.6422	518.3247	1070.6793	535.8433	K	614.3508		688.4352	344.7212	671.4087	336.2080	670.4246	335.7160	672.4165	336.7119	673.4243	337.2158	6
11	1181.7113	591.3593	1164.6848	582.8460	1163.7008	582.3540	1198.7379	599.8726	Q	486.2922		560.3402	280.6738	543.3137	272.1605	542.3297	271.6685	544.3215	272.6644	545.3293	273.1683	5
12	1294.7954	647.9013	1277.7688	639.3881	1276.7848	638.8960	1311.8219	656.4146	L	373.2082		432.2817	216.6445	415.2551	208.1312	414.2711	207.6392	416.2629	208.6351	417.2708	209.1390	4
13	1365.8325	683.4199	1348.8059	674.9066	1347.8219	674.4146	1382.8590	691.9332	A			319.1976	160.1024	302.1710	151.5892	301.1870	151.0972	303.1789	152.0931	304.1867	152.5970	3
14	1466.8802	733.9437	1449.8536	725.4305	1448.8696	724.9384	1483.9067	742.4570	T	215.1390	217.1183	248.1605	124.5839	231.1339	116.0706	230.1499	115.5786	232.1418	116.5745	233.1496	117.0784	2
15									K	73.0284		147.1128	74.0600	130.0863	65.5468			131.0941	66.0507	132.1019	66.5546	1

Monoisotopic mass of neutral peptide Mr(calc): 1611.9784
Fixed modifications: Carbamidomethyl (C) (apply to specified residues or termini only)
Variable modifications:
K1 : Trimethyl (K)
Ions Score: 81 **Expect:** 1.3e-05
Matches : 55/260 fragment ions using 47 most intense peaks (help)

Mascot Search Results: Peptide View

Kme3STGGKAPRKQLATK

MS/MS Fragmentation of KSTGGKAPRKQLATK
Found in sp|P68433|H31_MOUSE in fgcz_10090, Histone H3.1 OS=Mus musculus GN=Hist1h3a

Match to Query 2561: 1597.961416 from(400.497630,4+) intensity(135076.5200) scans(2247) rtinseconds(1322.8751) index(1424)
Title: 1425: Scan 2247 (rt=22.0479) [s:\p749\Proteomics\ORBI_2\andreamizzi_20110517_sperm_trypsin_GluC_ETD_CID\20110517_02_NaCl_2.RAW]
Data file 20110517_02_NaCl_2.mgf

Click mouse within plot area to zoom in by factor of two about that point
Or, Plot from 100 to 1500 Da Full range
Label all possible matches ○ Label matches used for scoring ⦿
Show Y-axis ☐

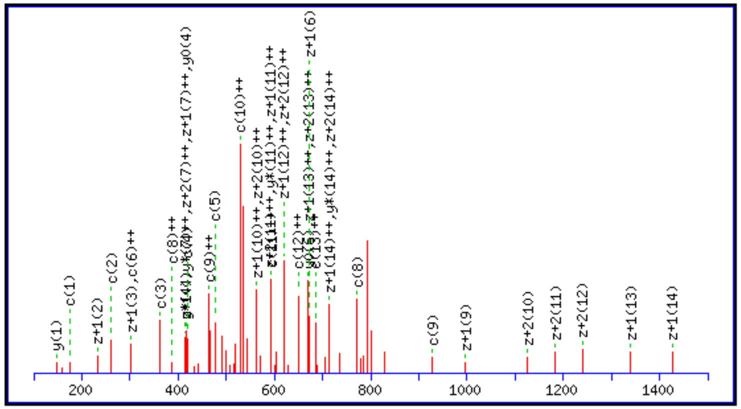

#	b	b++	b*	b*++	b0	b0++	c	c++	Seq.	w	w'	y	y++	y*	y*++	y0	y0++	z+1	z+1++	z+2	z+2++	#
1	157.1335	79.0704	140.1070	70.5571			174.1601	87.5837	K													15
2	244.1656	122.5864	227.1390	114.0731	226.1550	113.5811	261.1921	131.0997	S	1409.8223		1442.8438	721.9255	1425.8172	713.4123	1424.8332	712.9203	1426.8251	713.9162	1427.8329	714.4201	14
3	345.2132	173.1103	328.1867	164.5970	327.2027	164.1050	362.2398	181.6235	T	1322.7903	1324.7696	1355.8118	678.4095	1338.7852	669.8962	1337.8012	669.4042	1339.7930	670.4002	1340.8009	670.9041	13
4	402.2347	201.6210	385.2082	193.1077	384.2241	192.6157	419.2613	210.1343	G			1254.7641	627.8857	1237.7375	619.3724	1236.7535	618.8804	1238.7454	619.8763	1239.7532	620.3802	12
5	459.2562	230.1317	442.2296	221.6185	441.2456	221.1264	476.2827	238.6450	G			1197.7426	599.3749	1180.7161	590.8617	1179.7321	590.3697	1181.7239	591.3656	1182.7317	591.8695	11
6	587.3511	294.1792	570.3246	285.6659	569.3406	285.1739	604.3777	302.6925	K	1066.6368		1140.7212	570.8642	1123.6946	562.3509	1122.7106	561.8589	1124.7024	562.8549	1125.7103	563.3588	10
7	658.3883	329.6978	641.3617	321.1845	640.3777	320.6925	675.4148	338.2110	A			1012.6262	506.8167	995.5996	498.3035	994.6156	497.8115	996.6075	498.8074	997.6153	499.3113	9
8	755.4410	378.2241	738.4145	369.7109	737.4304	369.2189	772.4676	386.7374	P	898.5469		941.5891	471.2982	924.5625	462.7849	923.5785	462.2929	925.5704	463.2888	926.5782	463.7927	8
9	911.5421	456.2747	894.5156	447.7614	893.5316	447.2694	928.5687	464.7880	R	742.4458		844.5363	422.7718	827.5098	414.2585	826.5257	413.7665	828.5176	414.7624	829.5254	415.2663	7
10	1039.6371	520.3222	1022.6105	511.8089	1021.6265	511.3169	1056.6636	528.8355	K	614.3508		688.4352	344.7212	671.4087	336.2080	670.4246	335.7160	672.4165	336.7119	673.4243	337.2158	6
11	1167.6957	584.3515	1150.6691	575.8382	1149.6851	575.3462	1184.7222	592.8647	Q	486.2922		560.3402	280.6738	543.3137	272.1605	542.3297	271.6685	544.3215	272.6644	545.3293	273.1683	5
12	1280.7797	640.8935	1263.7532	632.3802	1262.7692	631.8882	1297.8063	649.4068	L	373.2082		432.2817	216.6445	415.2551	208.1312	414.2711	207.6392	416.2629	208.6351	417.2708	209.1390	4
13	1351.8168	676.4121	1334.7903	667.8988	1333.8063	667.4068	1368.8434	684.9253	A			319.1976	160.1024	302.1710	151.5892	301.1870	151.0972	303.1789	152.0931	304.1867	152.5970	3
14	1452.8645	726.9359	1435.8380	718.4226	1434.8540	717.9306	1469.8911	735.4492	T	215.1390	217.1183	248.1605	124.5839	231.1339	116.0706	230.1499	115.5786	232.1418	116.5745	233.1496	117.0784	2
15									K	73.0284		147.1128	74.0600	130.0863	65.5468			131.0941	66.0507	132.1019	66.5546	1

Monoisotopic mass of neutral peptide Mr(calc): 1597.9627
Fixed modifications: Carbamidomethyl (C) (apply to specified residues or termini only)
Variable modifications:
K1 : Dimethyl_KR (KR)
Ions Score: 72 **Expect:** 2.8e-05
Matches : 43/260 fragment ions using 38 most intense peaks (help)

Mascot Search Results: Peptide View

Kme2STGGKAPRKQLATK

MS/MS Fragmentation of **KSTGGKAPRKQLATK**
Found in **sp|P68433|H31_MOUSE** in **fgcz_10090**, Histone H3.1 OS=Mus musculus GN=Hist1h3a

Match to Query 1789: 1583.947336 from(396.994110,4+) intensity(22334.8790) scans(1701) rtinseconds(1315.3596) index(874)
Title: 875: Scan 1701 (rt=21.9227) [s:\p749\Proteomics\ORBI_2\andreamizzi_20110517_sperm_trypsin_GluC_ETD_CID\20110519_01_NaCl_ziptipped_1.RAW]
Data file 20110519_01_NaCl_ziptipped_1.mgf

Click mouse within plot area to zoom in by factor of two about that point
Or, Plot from 100 to 1600 Da Full range
Label all possible matches ○ Label matches used for scoring ◉
Show Y-axis ☐

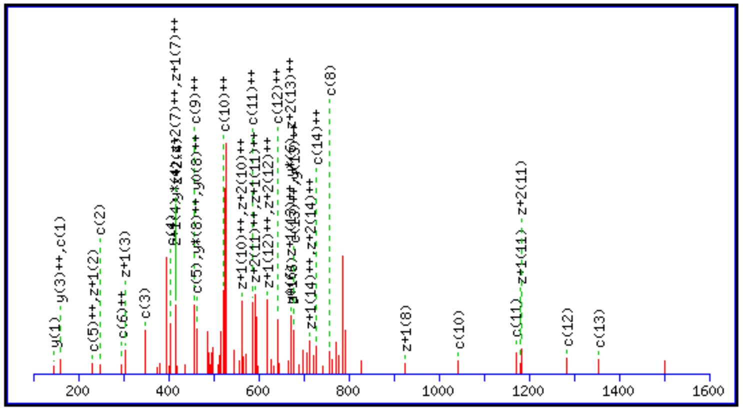

#	b	b^{++}	b^{*}	b^{*++}	b^{0}	b^{0++}	c	c^{++}	Seq.	w	w'	y	y^{++}	y^{*}	y^{*++}	y^{0}	y^{0++}	z+1	$z+1^{++}$	z+2	$z+2^{++}$	#
1	143.1179	72.0626	126.0913	63.5493			***160.1444***	80.5759	K													15
2	230.1499	115.5786	213.1234	107.0653	212.1394	106.5733	***247.1765***	124.0919	S	1409.8223		1442.8438	721.9255	1425.8172	713.4123	1424.8332	712.9202	1426.8251	**713.9162**	1427.8329	**714.4201**	14
3	331.1976	166.1024	314.1710	157.5892	313.1870	157.0972	***348.2241***	174.6157	T	1322.7903	1324.7696	1355.8118	678.4095	1338.7852	669.8962	1337.8012	669.4042	1339.7930	**670.4002**	1340.8009	**670.9041**	13
4	388.2191	194.6132	371.1925	186.0999	370.2085	185.6079	***405.2456***	203.1264	G			1254.7641	627.8857	1237.7375	619.3724	1236.7535	618.8804	1238.7454	**619.8763**	1239.7532	**620.3802**	12
5	445.2405	223.1239	428.2140	214.6106	427.2300	214.1186	***462.2671***	**231.6372**	G			1197.7426	599.3749	1180.7161	590.8617	1179.7321	590.3697	**1181.7239**	**591.3656**	1182.7317	**591.8695**	11
6	573.3355	287.1714	556.3089	278.6581	555.3249	278.1661	590.3620	**295.6847**	K	1066.6368		1140.7212	570.8642	1123.6946	562.3509	1122.7106	561.8589	1124.7024	**562.8549**	1125.7103	**563.3588**	10
7	644.3726	322.6899	627.3461	314.1767	626.3620	313.6847	661.3992	331.2032	A			1012.6262	506.8167	995.5996	498.3035	994.6156	497.8115	996.6075	498.8074	997.6153	499.3113	9
8	741.4254	371.2163	724.3988	362.7030	723.4148	362.2110	***758.4519***	379.7296	P	898.5469		941.5891	471.2982	924.5625	462.7849	923.5785	462.2929	**925.5704**	463.2888	926.5782	463.7927	8
9	897.5265	449.2669	880.4999	440.7536	879.5159	440.2616	914.5530	**457.7802**	R	742.4458		844.5363	422.7718	827.5098	414.2585	826.5257	413.7665	828.5176	**414.7624**	829.5254	**415.2663**	7
10	1025.6214	513.3144	1008.5949	504.8011	1007.6109	504.3091	***1042.6480***	**521.8276**	K	614.3508		688.4352	344.7212	671.4087	336.2080	670.4246	335.7160	**672.4165**	336.7119	673.4243	337.2158	6
11	1153.6800	577.3436	1136.6535	568.8304	1135.6695	568.3384	***1170.7066***	**585.8569**	Q	486.2922		560.3402	280.6738	543.3137	272.1605	542.3297	271.6685	544.3215	272.6644	545.3293	273.1683	5
12	1266.7641	633.8857	1249.7375	625.3724	1248.7535	624.8804	***1283.7906***	**642.3990**	L	373.2082		432.2817	216.6445	415.2551	208.1312	414.2711	207.6392	**416.2629**	208.6351	417.2708	209.1390	4
13	1337.8012	669.4042	1320.7746	660.8910	1319.7906	660.3990	***1354.8277***	**677.9175**	A			319.1976	160.1024	302.1710	151.5892	301.1870	151.0972	**303.1789**	152.0931	304.1867	152.5970	3
14	1438.8489	719.9281	1421.8223	711.4148	1420.8383	710.9228	1455.8754	**728.4413**	T	215.1390	217.1183	248.1605	124.5839	231.1339	116.0706	230.1499	115.5786	**232.1418**	116.5745	233.1496	117.0784	2
15									K	73.0284		147.1128	74.0600	130.0863	65.5468			131.0941	66.0507	132.1019	66.5546	1

Monoisotopic mass of neutral peptide Mr(calc): 1583.9471
Fixed modifications: Carbamidomethyl (C) (apply to specified residues or termini only)
Variable modifications:
K1 : Methyl_KR (KR)
Ions Score: 70 **Expect:** 4.4e-05
Matches : 46/260 fragment ions using 45 most intense peaks (help)

Mascot Search Results: Peptide View

Kme1STGGKAPRKQLATK

MS/MS Fragmentation of GGAKRHRKVLR
Found in sp|P62806|H4_MOUSE in fgcz_10090, Histone H4 OS=Mus musculus GN=Hist1h4a

Match to Query 1447: 1304.826296 from(327.213850,4+) intensity(54474.4140) scans(1245) rtinseconds(857.6954) index(627)
Title: 628: Scan 1245 (rt=14.2949) [s:\p749\Proteomics\ORBI_2\andreamizzi_20110310_sperm_trypsin_chymo_AspN_ETD_CID\20110310_11_NaCl_trypsin.RAW]
Data file \\fgcz-ms.uzh.ch\p749\Proteomics\ORBI_2\andreamizzi_20110310_sperm_trypsin_chymo_AspN_ETD_CID\mgf__low_res_MS2_nomerge\20110310_11_NaCl_trypsin.mgf

Click mouse within plot area to zoom in by factor of two about that point
Or, Plot from 100 to 1300 Da Full range
Label all possible matches ○ Label matches used for scoring ⦿
Show Y-axis ☐

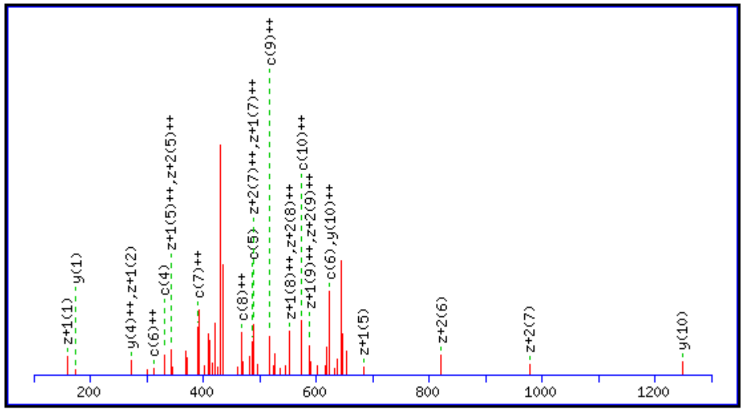

#	b	b^{++}	b*	b^{*++}	c	c^{++}	Seq.	w	y	y^{++}	y*	y^{*++}	z+1	$z+1^{++}$	z+2	$z+2^{++}$	#
1	58.0287	29.5180			75.0553	38.0313	G										11
2	115.0502	58.0287			132.0768	66.5420	G		**1248.8124**	624.9098	1231.7858	616.3965	1232.7936	616.9005	1233.8015	617.4044	10
3	186.0873	93.5473			203.1139	102.0606	A		1191.7909	596.3991	1174.7644	587.8858	1175.7722	***588.3897***	1176.7800	**588.8936**	9
4	314.1823	157.5948	297.1557	149.0815	**331.2088**	166.1081	K	1046.6694	1120.7538	560.8805	1103.7272	552.3673	1104.7351	***552.8712***	1105.7429	**553.3751**	8
5	470.2834	235.6453	453.2568	227.1321	**487.3099**	244.1586	R	890.5683	992.6588	496.8331	975.6323	488.3198	976.6401	***488.8237***	**977.6479**	**489.3276**	7
6	607.3423	304.1748	590.3158	295.6615	**624.3689**	***312.6881***	H		836.5577	418.7825	819.5312	410.2692	820.5390	410.7731	**821.5468**	411.2770	6
7	763.4434	382.2253	746.4169	373.7121	780.4700	***390.7386***	R	597.4083	699.4988	350.2530	682.4723	341.7398	**683.4801**	***342.2437***	684.4879	**342.7476**	5
8	919.5697	460.2885	902.5431	451.7752	936.5962	***468.8018***	K	441.2820	543.3977	272.2025	526.3711	263.6892	527.3790	264.1931	528.3868	264.6970	4
9	1018.6381	509.8227	1001.6115	501.3094	1035.6646	***518.3360***	V	356.2292	387.2714	194.1394	370.2449	185.6261	371.2527	186.1300	372.2605	186.6339	3
10	1131.7222	566.3647	1114.6956	557.8514	1148.7487	***574.8780***	L	229.1295	288.2030	144.6051	271.1765	136.0919	**272.1843**	136.5958	273.1921	137.0997	2
11							R	73.0284	**175.1190**	88.0631	158.0924	79.5498	**159.1002**	80.0538	160.1081	80.5577	1

Monoisotopic mass of neutral peptide Mr(calc): 1304.8265
Fixed modifications: Carbamidomethyl (C) (apply to specified residues or termini only)
Variable modifications:
K8 : Dimethyl_KR (KR)
Ions Score: 42 **Expect:** 0.02
Matches : 25/141 fragment ions using 26 most intense peaks (help)

Mascot Search Results: Peptide View

GGAKRHRKme2VLR

MS/MS Fragmentation of GKGGKGLGKGGAKR
Found in sp|P62806|H4_MOUSE in fgcz_10090, Histone H4 OS=Mus musculus GN=Hist1h4a

Match to Query 1549: 1395.794202 from(466.272010,3+) intensity(35282.6720) scans(2761) rtinseconds(1633.4172) index(1438)
Title: 1439: Scan 2761 (rt=27.2236) [s:\p749\Proteomics\ORBI_2\andreamizzi_201110127_sperm_trypsin_ETD_CID\20110127_08_ON7_saltex.RAW]
Data file \\fgcz-ms.uzh.ch\p749\Proteomics\ORBI_2\andreamizzi_20110127_sperm_trypsin_ETD_CID\mgf__low_res_MS2_nomerge\20110127_08_ON7_saltex.mgf

Click mouse within plot area to zoom in by factor of two about that point
Or, Plot from 200 to 1400 Da Full range
Label all possible matches ○ Label matches used for scoring ◉
Show Y-axis ☐

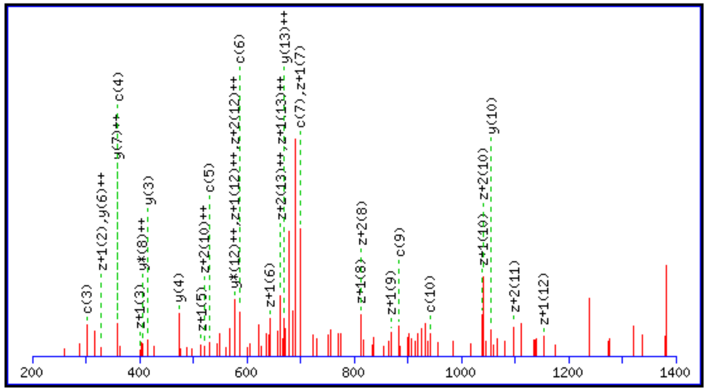

#	b	b^{++}	b*	b^{*++}	c	c^{++}	Seq.	w	y	y^{++}	y*	y^{*++}	z+1	$z+1^{++}$	z+2	$z+2^{++}$	#
1	58.0287	29.5180			75.0553	38.0313	G										14
2	228.1343	114.5708	211.1077	106.0575	245.1608	123.0840	K	1223.6855	1339.7805	670.3939	1322.7539	661.8806	1323.7617	662.3845	1324.7696	**662.8884**	13
3	285.1557	143.0815	268.1292	134.5682	**302.1823**	151.5948	G		1169.6749	585.3411	1152.6484	576.8278	***1153.6562***	577.3317	1154.6640	**577.8357**	12
4	342.1772	171.5922	325.1506	163.0790	**359.2037**	180.1055	G		1112.6535	556.8304	1095.6269	548.3171	1096.6347	548.8210	**1097.6426**	549.3249	11
5	512.2827	256.6450	495.2562	248.1317	**529.3093**	265.1583	K	939.5370	**1055.6320**	528.3196	1038.6055	519.8064	***1039.6133***	520.3103	**1040.6211**	**520.8142**	10
6	569.3042	285.1557	552.2776	276.6425	**586.3307**	293.6690	G		885.5265	443.2669	868.4999	434.7536	***869.5078***	435.2575	870.5156	435.7614	9
7	682.3883	341.6978	665.3617	333.1845	**699.4148**	350.2110	L	769.4315	828.5050	414.7561	811.4785	406.2429	***812.4863***	406.7468	**813.4941**	407.2507	8
8	739.4097	370.2085	722.3832	361.6952	756.4363	378.7218	G		715.4209	358.2141	698.3944	349.7008	***699.4022***	350.2048	700.4101	350.7087	7
9	867.5047	434.2560	850.4781	425.7427	**884.5312**	442.7693	K	584.3151	658.3995	329.7034	641.3729	321.1901	***642.3808***	321.6940	643.3886	322.1979	6
10	924.5261	462.7667	907.4996	454.2534	**941.5527**	471.2800	G		530.3045	265.6559	513.2780	257.1426	***514.2858***	257.6465	515.2936	258.1504	5
11	981.5476	491.2774	964.5211	482.7642	998.5742	499.7907	G		**473.2831**	237.1452	456.2565	228.6319	457.2643	229.1358	458.2722	229.6397	4
12	1052.5847	526.7960	1035.5582	518.2827	1069.6113	535.3093	A		**416.2616**	208.6344	399.2350	200.1212	***400.2429***	200.6251	401.2507	201.1290	3
13	1222.6902	611.8488	1205.6637	603.3355	1239.7168	620.3620	K	229.1295	345.2245	173.1159	328.1979	164.6026	***329.2058***	165.1065	330.2136	165.6104	2
14							R	73.0284	175.1190	88.0631	158.0924	79.5498	159.1002	80.0538	160.1081	80.5577	1

```
Monoisotopic mass of neutral peptide Mr(calc): 1395.7946
Fixed modifications: Carbamidomethyl (C) (apply to specified residues or termini only)
Variable modifications:
K2     : Acetyl (K)
K5     : Acetyl (K)
K13    : Acetyl (K)
Ions Score: 45  Expect: 0.002
Matches : 32/186 fragment ions using 62 most intense peaks   (help)
```

Mascot Search Results: Peptide View

GKacGGKacGLGKGGAKacR

MS/MS Fragmentation of **GKGGKGLGKGGAKR**
Found in **sp|P62806|H4_MOUSE** in **fgcz_10090**, Histone H4 OS=Mus musculus GN=Hist1h4a

Match to Query 1643: 1437.806048 from(719.910300,2+) intensity(118046.9800) scans(3659) rtinseconds(2166.5045) index(2337)
Title: 2338: Scan 3659 (rt=36.1084) [s:\p749\Proteomics\ORBI_2\andreamizzi_20110517_sperm_trypsin_GluC_ETD_CID\20110519_02_NaCl_ziptipped_2.RAW]
Data file 20110519_02_NaCl_ziptipped_2.mgf

Click mouse within plot area to zoom in by factor of two about that point
Or, Plot from 400 to 1500 Da Full range
Label all possible matches ○ Label matches used for scoring ◉
Show Y-axis ☐

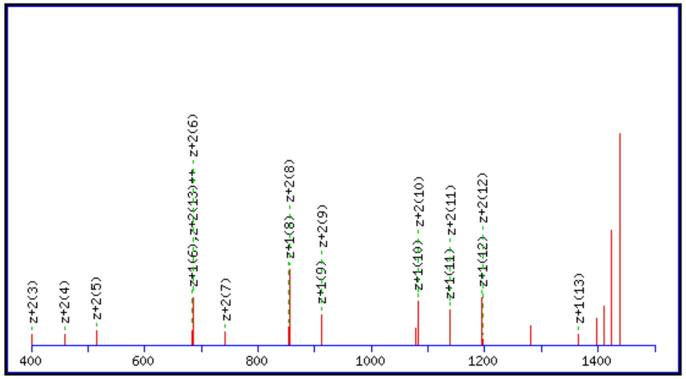

#	b	b^{++}	b*	b^{*++}	c	c^{++}	Seq.	w	y	y^{++}	y*	y^{*++}	z+1	$z+1^{++}$	z+2	$z+2^{++}$	#
1	58.0287	29.5180			75.0553	38.0313	G										14
2	228.1343	114.5708	211.1077	106.0575	245.1608	123.0840	K	1265.6961	1381.7910	691.3992	1364.7645	682.8859	**1365.7723**	683.3898	1366.7801	683.8937	13
3	285.1557	143.0815	268.1292	134.5682	302.1823	151.5948	G		1211.6855	606.3464	1194.6589	597.8331	**1195.6668**	598.3370	***1196.6746***	598.8409	12
4	342.1772	171.5922	325.1506	163.0790	359.2037	180.1055	G		1154.6640	577.8357	1137.6375	569.3224	**1138.6453**	569.8263	***1139.6531***	570.3302	11
5	512.2827	256.6450	495.2562	248.1317	529.3093	265.1583	K	981.5476	1097.6426	549.3249	1080.6160	540.8116	**1081.6238**	541.3156	***1082.6317***	541.8195	10
6	569.3042	285.1557	552.2776	276.6425	586.3307	293.6690	G		927.5370	464.2722	910.5105	455.7589	**911.5183**	456.2628	***912.5261***	456.7667	9
7	682.3883	341.6978	665.3617	333.1845	699.4148	350.2110	L	811.4421	870.5156	435.7614	853.4890	427.2482	**854.4969**	427.7521	***855.5047***	428.2560	8
8	739.4097	370.2085	722.3832	361.6952	756.4363	378.7218	G		757.4315	379.2194	740.4050	370.7061	741.4128	371.2100	***742.4206***	371.7139	7
9	909.5152	455.2613	892.4887	446.7480	926.5418	463.7745	K	584.3151	700.4101	350.7087	683.3835	342.1954	**684.3913**	342.6993	***685.3992***	343.2032	6
10	966.5367	483.7720	949.5102	475.2587	983.5633	492.2853	G		530.3045	265.6559	513.2780	257.1426	514.2858	257.6465	***515.2936***	258.1504	5
11	1023.5582	512.2827	1006.5316	503.7694	1040.5847	520.7960	G		473.2831	237.1452	456.2565	228.6319	457.2643	229.1358	***458.2722***	229.6397	4
12	1094.5953	547.8013	1077.5687	539.2880	1111.6218	556.3146	A		416.2616	208.6344	399.2350	200.1212	400.2429	200.6251	***401.2507***	201.1290	3
13	1264.7008	632.8540	1247.6743	624.3408	1281.7274	641.3673	K	229.1295	345.2245	173.1159	328.1979	164.6026	329.2058	165.1065	330.2136	165.6104	2
14							R	73.0284	175.1190	88.0631	158.0924	79.5498	159.1002	80.0538	160.1081	80.5577	1

Monoisotopic mass of neutral peptide Mr(calc): 1437.8052
Fixed modifications: Carbamidomethyl (C) (apply to specified residues or termini only)
Variable modifications:
K2 : Acetyl (K)
K5 : Acetyl (K)
K9 : Acetyl (K)
K13 : Acetyl (K)
Ions Score: 95 **Expect:** 5.8e-07
Matches : 18/186 fragment ions using 22 most intense peaks (help)

Mascot Search Results: Peptide View

GKacGGKacGLGKacGGAKacR

MS/MS Fragmentation of HRKVLRDNIQGITKPAIR
Found in sp|P62806|H4_MOUSE in fgcz_10090, Histone H4 OS=Mus musculus GN=Hist1h4a

Match to Query 2483: 2156.303496 from(540.083150,4+) intensity(203420.1600) scans(3026) rtinseconds(1908.7141) index(1494)
Title: 1495: Scan 3026 (rt=31.8119) [s:\p749\Proteomics\ORBI_2\andreamizzi_201110127_sperm_trypsin_ETD_CID\20110127_09_2h7_saltex.RAW]
Data file \\fgcz-ms.uzh.ch\p749\Proteomics\ORBI_2\andreamizzi_20110127_sperm_trypsin_ETD_CID\mgf__low_res_MS2_nomerge\20110127_09_2h7_saltex.mgf

Click mouse within plot area to zoom in by factor of two about that point
Or, Plot from 200 to 1900 Da Full range
Label all possible matches ○ Label matches used for scoring ◉
Show Y-axis ☐

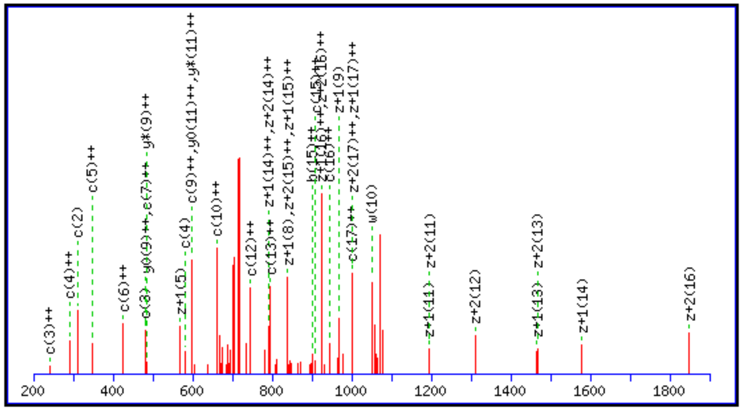

#	b	b^{++}	b*	b^{*++}	b^0	b^{0++}	c	c^{++}	Seq.	w	w'	y	y^{++}	y*	y^{*++}	y^0	y^{0++}	z+1	$z+1^{++}$	z+2	$z+2^{++}$	#
1	138.0662	69.5367					155.0927	78.0500	H													18
2	294.1673	147.5873	277.1408	139.0740			311.1938	156.1006	R	1918.1596		2020.2502	1010.6287	2003.2236	1002.1155	2002.2396	1001.6234	2004.2315	1002.6194	2005.2393	1003.1233	17
3	464.3092	232.6582	447.2827	224.1450			481.3358	241.1715	K	1748.0177		1864.1491	932.5782	1847.1225	924.0649	1846.1385	923.5729	1848.1304	924.5688	1849.1382	925.0727	16
4	563.3776	282.1925	546.3511	273.6792			580.4042	290.7057	V	1662.9650		1694.0072	847.5072	1676.9806	838.9939	1675.9966	838.5019	1677.9884	839.4979	1678.9963	840.0018	15
5	676.4617	338.7345	659.4351	330.2212			693.4882	347.2478	L	1535.8653		1594.9388	797.9730	1577.9122	789.4597	1576.9282	788.9677	1578.9200	789.9637	1579.9279	790.4676	14
6	832.5628	416.7850	815.5363	408.2718			849.5894	425.2983	R	1379.7641		1481.8547	741.4310	1464.8281	732.9177	1463.8441	732.4257	1465.8360	733.4216	1466.8438	733.9255	13
7	947.5897	474.2985	930.5632	465.7852	929.5792	465.2932	964.6163	482.8118	D	1264.7372		1325.7536	663.3804	1308.7270	654.8672	1307.7430	654.3751	1309.7349	655.3711	1310.7427	655.8750	12
8	1061.6327	531.3200	1044.6061	522.8067	1043.6221	522.3147	1078.6592	539.8332	N	1150.6943		1210.7266	605.8670	1193.7001	597.3537	1192.7161	596.8617	1194.7079	597.8576	1195.7157	598.3615	11
9	1174.7167	587.8620	1157.6902	579.3487	1156.7062	578.8567	1191.7433	596.3753	I	1051.6259	1065.6415	1096.6837	548.8455	1079.6572	540.3322	1078.6731	539.8402	1080.6650	540.8361	1081.6728	541.3400	10
10	1302.7753	651.8913	1285.7488	643.3780	1284.7647	642.8860	1319.8019	660.4046	Q	909.5516		983.5996	492.3035	966.5731	483.7902	965.5891	483.2982	967.5809	484.2941	968.5887	484.7980	9
11	1359.7968	680.4020	1342.7702	671.8888	1341.7862	671.3967	1376.8233	688.9153	G			855.5411	428.2742	838.5145	419.7609	837.5305	419.2689	839.5223	420.2648	840.5302	420.7687	8
12	1472.8808	736.9441	1455.8543	728.4308	1454.8703	727.9388	1489.9074	745.4573	I	753.4618	767.4774	798.5196	399.7634	781.4931	391.2502	780.5090	390.7582	782.5009	391.7541	783.5087	392.2580	7
13	1573.9285	787.4679	1556.9020	778.9546	1555.9180	778.4626	1590.9551	795.9812	T	652.4141	654.3933	685.4355	343.2214	668.4090	334.7081	667.4250	334.2161	669.4168	335.2120	670.4246	335.7160	6
14	1702.0235	851.5154	1684.9969	843.0021	1684.0129	842.5101	1719.0500	860.0287	K	510.3035		584.3879	292.6976	567.3613	284.1843			568.3691	284.6882	569.3770	285.1921	5
15	1799.0762	900.0418	1782.0497	891.5285	1781.0657	891.0365	1816.1028	908.5550	P	413.2507		456.2929	228.6501	439.2663	220.1368			440.2742	220.6407	441.2820	221.1446	4
16	1870.1134	935.5603	1853.0868	927.0470	1852.1028	926.5550	1887.1399	944.0736	A			359.2401	180.1237	342.2136	171.6104			343.2214	172.1143	344.2292	172.6183	3
17	1983.1974	992.1024	1966.1709	983.5891	1965.1869	983.0971	2000.2240	1000.6156	I	243.1452	257.1608	288.2030	144.6051	271.1765	136.0919			272.1843	136.5958	273.1921	137.0997	2
18									R	73.0284		175.1190	88.0631	158.0924	79.5498			159.1002	80.0538	160.1081	80.5577	1

Monoisotopic mass of neutral peptide Mr(calc): 2156.3018
Fixed modifications: Carbamidomethyl (C) (apply to specified residues or termini only)
Variable modifications:
K3 : Trimethyl (K)
Ions Score: 95 **Expect:** 5.5e-07
Matches : 39/301 fragment ions using 39 most intense peaks (help)

Mascot Search Results: Peptide View

HRKme3VLRDNIQGITKPAIR

MS/MS Fragmentation of HRKVLRDNIQGITKPAIR
Found in sp|P62806|H4_MOUSE in fgcz_10090, Histone H4 OS=Mus musculus GN=Hist1h4a

Match to Query 2834: 2142.287456 from(536.579140,4+) intensity(238197.0900) scans(3650) rtinseconds(2156.5633) index(2180)
Title: 2181: Scan 3650 (rt=35.9427) [s:\p749\Proteomics\ORBI_2\andreamizzi_20110310_sperm_trypsin_chymo_AspN_ETD_CID\20110310_11_NaCl_trypsin.RAW]
Data file \\fgcz-ms.uzh.ch\p749\Proteomics\ORBI_2\andreamizzi_20110310_sperm_trypsin_chymo_AspN_ETD_CID\mgf__low_res_MS2_nomerge\20110310_11_NaCl_trypsin.mgf

Click mouse within plot area to zoom in by factor of two about that point
Or, Plot from 200 to 2000 Da Full range
Label all possible matches ○ Label matches used for scoring ◉
Show Y-axis ☐

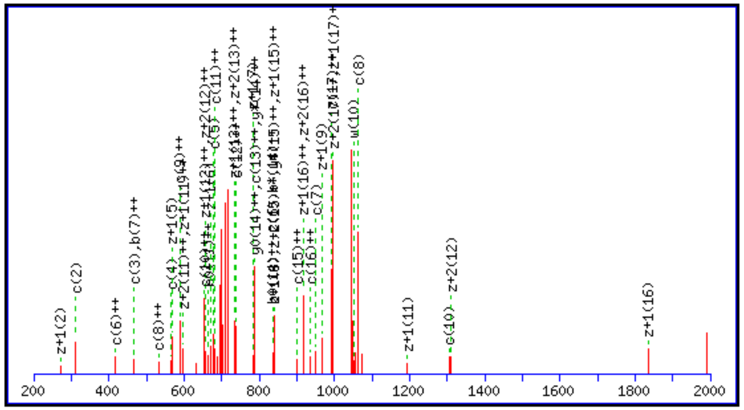

#	b	b^{++}	b^{*}	b^{*++}	b^{0}	b^{0++}	c	c^{++}	Seq.	w	w'	y	y^{++}	y^{*}	y^{*++}	y^{0}	y^{0++}	z+1	$z+1^{++}$	z+2	$z+2^{++}$	#
1	138.0662	69.5367					155.0927	78.0500	H													18
2	294.1673	147.5873	277.1408	139.0740			311.1938	156.1006	R	1904.1440		2006.2345	1003.6209	1989.2080	995.1076	1988.2240	994.6156	1990.2158	995.6115	1991.2236	996.1155	17
3	450.2936	225.6504	433.2670	217.1371			467.3201	234.1637	K	1748.0177		1850.1334	925.5704	1833.1069	917.0571	1832.1229	916.5651	1834.1147	917.5610	1835.1225	918.0649	16
4	549.3620	275.1846	532.3354	266.6714			566.3885	283.6979	V	1662.9650		1694.0072	847.5072	1676.9806	838.9939	1675.9966	838.5019	1677.9884	839.4979	1678.9963	840.0018	15
5	662.4460	331.7267	645.4195	323.2134			679.4726	340.2399	L	1535.8653		1594.9388	797.9730	1577.9122	789.4597	1576.9282	788.9677	1578.9200	789.9637	1579.9279	790.4676	14
6	818.5472	409.7772	801.5206	401.2639			835.5737	418.2905	R	1379.7641		1481.8547	741.4310	1464.8281	732.9177	1463.8441	732.4257	1465.8360	733.4216	1466.8438	733.9255	13
7	933.5741	467.2907	916.5475	458.7774	915.5635	458.2854	950.6006	475.8040	D	1264.7372		1325.7536	663.3804	1308.7270	654.8672	1307.7430	654.3751	1309.7349	655.3711	1310.7427	655.8750	12
8	1047.6170	524.3121	1030.5905	515.7989	1029.6065	515.3069	1064.6436	532.8254	N	1150.6943		1210.7266	605.8670	1193.7001	597.3537	1192.7161	596.8617	1194.7079	597.8576	1195.7157	598.3615	11
9	1160.7011	580.8542	1143.6745	572.3409	1142.6905	571.8489	1177.7276	589.3675	I	1051.6259	1065.6415	1096.6837	548.8455	1079.6572	540.3322	1078.6731	539.8402	1080.6650	540.8361	1081.6728	541.3400	10
10	1288.7597	644.8835	1271.7331	636.3702	1270.7491	635.8782	1305.7862	653.3967	Q	909.5516		983.5996	492.3035	966.5731	483.7902	965.5891	483.2982	967.5809	484.2941	968.5887	484.7980	9
11	1345.7811	673.3942	1328.7546	664.8809	1327.7706	664.3889	1362.8077	681.9075	G			855.5411	428.2742	838.5145	419.7609	837.5305	419.2689	839.5223	420.2648	840.5302	420.7687	8
12	1458.8652	729.9362	1441.8386	721.4230	1440.8546	720.9310	1475.8917	738.4495	I	753.4618	767.4774	798.5196	399.7634	781.4931	391.2502	780.5090	390.7582	782.5009	391.7541	783.5087	392.2580	7
13	1559.9129	780.4601	1542.8863	771.9468	1541.9023	771.4548	1576.9394	788.9733	T	652.4141	654.3933	685.4355	343.2214	668.4090	334.7081	667.4250	334.2161	669.4168	335.2120	670.4246	335.7160	6
14	1688.0078	844.5076	1670.9813	835.9943	1669.9973	835.5023	1705.0344	853.0208	K	510.3035		584.3879	292.6976	567.3613	284.1843			568.3691	284.6882	569.3770	285.1921	5
15	1785.0606	893.0339	1768.0340	884.5207	1767.0500	884.0287	1802.0871	901.5472	P	413.2507		456.2929	228.6501	439.2663	220.1368			440.2742	220.6407	441.2820	221.1446	4
16	1856.0977	928.5525	1839.0712	920.0392	1838.0871	919.5472	1873.1243	937.0658	A			359.2401	180.1237	342.2136	171.6104			343.2214	172.1143	344.2292	172.6183	3
17	1969.1818	985.0945	1952.1552	976.5813	1951.1712	976.0892	1986.2083	993.6078	I	243.1452	257.1608	288.2030	144.6051	271.1765	136.0919			272.1843	136.5958	273.1921	137.0997	2
18									R	73.0284		175.1190	88.0631	158.0924	79.5498			159.1002	80.0538	160.1081	80.5577	1

Monoisotopic mass of neutral peptide Mr(calc): 2142.2861
Fixed modifications: Carbamidomethyl (C) (apply to specified residues or termini only)
Variable modifications:
K3 : Dimethyl_KR (KR)
Ions Score: 71 **Expect:** 0.00016
Matches : 47/301 fragment ions using 46 most intense peaks (help)

Mascot Search Results: Peptide View

HRKme2VLRDNIQGITKPAIR

MS/MS Fragmentation of **HRKVLRDNIQGITKPAIR**
Found in **sp|P62806|H4_MOUSE** in **fgcz_10090**, Histone H4 OS=Mus musculus GN=Hist1h4a

Match to Query 4484: 2128.270536 from(533.074910,4+) intensity(15082.3940) scans(3179) rtinseconds(2214.3129) index(1492)
Title: 1348: Scan 3179 (rt=36.9052) [s:\p749\Proteomics\ORBI_2\andreamizzi_20110310_sperm_trypsin_chymo_AspN_ETD_CID\20110311_37_NaCl_trypsin_SCX_3.RAW]
Data file Y:\p749\Proteomics\Mascot\mgf\50 20110426 sperm rerun trypsin 2nd ETD run NaCl SCX\mascot_daemon_merge.mgf

Click mouse within plot area to zoom in by factor of two about that point
Or, Plot from 200 to 2000 Da Full range
Label all possible matches ○ Label matches used for scoring ⦿
Show Y-axis ☐

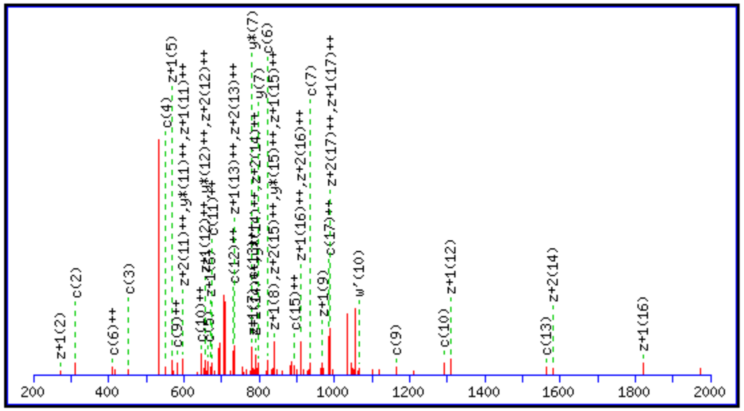

#	b	b++	b*	b*++	b0	b0++	c	c++	Seq.	w	w'	y	y++	y*	y*++	y0	y0++	z+1	z+1++	z+2	z+2++	#
1	138.0662	69.5367					155.0927	78.0500	H													18
2	294.1673	147.5873	277.1408	139.0740			**311.1938**	156.1006	R	1890.1283		1992.2189	996.6131	1975.1923	988.0998	1974.2083	987.6078	1976.2002	**988.6037**	1977.2080	**989.1076**	17
3	436.2779	218.6426	419.2514	210.1293			**453.3045**	227.1559	K	1748.0177		1836.1178	918.5625	1819.0912	910.0493	1818.1072	909.5572	**1820.0991**	**910.5532**	1821.1069	**911.0571**	16
4	535.3463	268.1768	518.3198	259.6635			**552.3729**	276.6901	V	1662.9650		1694.0072	847.5072	1676.9806	838.9939	1675.9966	838.5019	1677.9884	**839.4979**	1678.9963	**840.0018**	15
5	648.4304	324.7188	631.4038	316.2056			**665.4569**	333.2321	L	1535.8653		1594.9388	797.9730	1577.9122	789.4597	1576.9282	788.9677	1578.9200	**789.9637**	1579.9279	**790.4676**	14
6	804.5315	402.7694	787.5050	394.2561			**821.5581**	**411.2827**	R	1379.7641		1481.8547	741.4310	1464.8281	732.9177	1463.8441	732.4257	1465.8360	**733.4216**	1466.8438	**733.9255**	13
7	919.5584	460.2829	902.5319	451.7696	901.5479	451.2776	**936.5850**	468.7961	D	1264.7372		1325.7536	663.3804	1308.7270	654.8672	1307.7430	654.3751	**1309.7349**	**655.3711**	1310.7427	**655.8750**	12
8	1033.6014	517.3043	1016.5748	508.7910	1015.5908	508.2990	1050.6279	525.8176	N	1150.6943		1210.7266	605.8670	1193.7001	597.3537	1192.7161	596.8617	1194.7079	**597.8576**	1195.7157	**598.3615**	11
9	1146.6854	573.8464	1129.6589	565.3331	1128.6749	564.8411	**1163.7120**	**582.3596**	I	1051.6259	1065.6415	1096.6837	548.8455	1079.6572	540.3322	1078.6731	539.8402	1080.6650	540.8361	1081.6728	541.3400	10
10	1274.7440	637.8756	1257.7175	629.3624	1256.7334	628.8704	**1291.7706**	**646.3889**	Q	909.5516		983.5996	492.3035	966.5731	483.7902	965.5891	483.2982	**967.5809**	484.2941	968.5887	484.7980	9
11	1331.7655	666.3864	1314.7389	657.8731	1313.7549	657.3811	1348.7920	**674.8997**	G			855.5411	428.2742	838.5145	419.7609	837.5305	419.2689	**839.5223**	420.2648	840.5302	420.7687	8
12	1444.8495	722.9284	1427.8230	714.4151	1426.8390	713.9231	1461.8761	**731.4417**	I	753.4618	767.4774	798.5196	399.7634	781.4931	391.2502	780.5090	390.7582	**782.5009**	391.7541	783.5087	392.2580	7
13	1545.8972	773.4522	1528.8707	764.9390	1527.8867	764.4470	**1562.9238**	**781.9655**	T	652.4141	654.3933	685.4355	343.2214	668.4090	334.7081	667.4250	334.2161	**669.4168**	335.2120	670.4246	335.7160	6
14	1673.9922	837.4997	1656.9656	828.9865	1655.9816	828.4944	1691.0187	846.0130	K	510.3035		584.3879	292.6976	567.3613	284.1843			**568.3691**	284.6882	569.3770	285.1921	5
15	1771.0449	886.0261	1754.0184	877.5128	1753.0344	877.0208	1788.0715	**894.5394**	P	413.2507		456.2929	228.6501	439.2663	220.1368			440.2742	220.6407	441.2820	221.1446	4
16	1842.0821	921.5447	1825.0555	913.0314	1824.0715	912.5394	1859.1086	930.0579	A			359.2401	180.1237	342.2136	171.6104			343.2214	172.1143	344.2292	172.6183	3
17	1955.1661	978.0867	1938.1396	969.5734	1937.1556	969.0814	1972.1927	**986.6000**	I	243.1452	257.1608	288.2030	144.6051	271.1765	136.0919			**272.1843**	136.5958	273.1921	137.0997	2
18									R	73.0284		175.1190	88.0631	158.0924	79.5498			159.1002	80.0538	160.1081	80.5577	1

Monoisotopic mass of neutral peptide Mr(calc): 2128.2705
Fixed modifications: Carbamidomethyl (C) (apply to specified residues or termini only)
Variable modifications:
K3 : Methyl_KR (KR)
Ions Score: 58 **Expect:** 0.00025
Matches : 47/301 fragment ions using 53 most intense peaks (help)

Mascot Search Results: Peptide View

HRKme1VLRDNIQGITKPAIR

MS/MS Fragmentation of **HRKVLRDNIQGITKPAIRR**
Found in **sp|P62806|H4_MOUSE** in **fgcz_10090**, Histone H4 OS=Mus musculus GN=Hist1h4a

Match to Query 3344: 2298.382570 from(460.683790,5+) intensity(12884.6850) scans(3807) rtinseconds(2306.6157) index(2337)
Title: 2338: Scan 3807 (rt=38.4436) [s:\p749\Proteomics\ORBI_2\andreamizzi_20110517_sperm_trypsin_GluC_ETD_CID\20110517_05_swimup_NaCl_1.RAW]
Data file 20110517_05_swimup_NaCl_1.mgf

Click mouse within plot area to zoom in by factor of two about that point
Or, Plot from 100 to 1500 Da Full range
Label all possible matches ○ Label matches used for scoring ◉
Show Y-axis ☐

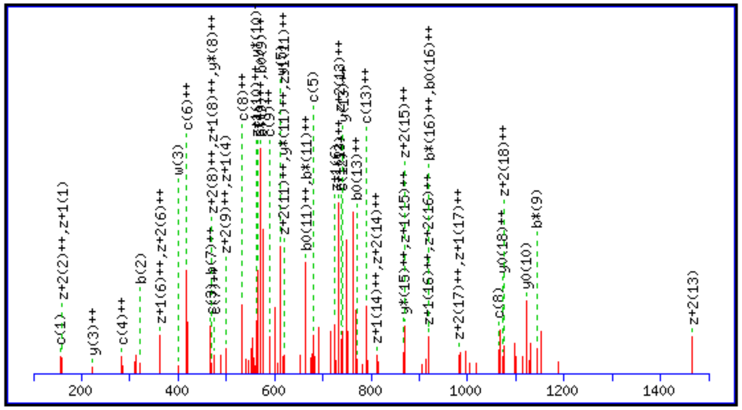

#	b	b^{++}	b^{*}	b^{*++}	b^{0}	b^{0++}	c	c^{++}	Seq.	w	w'	y	y^{++}	y^{*}	y^{*++}	y^{0}	y^{0++}	z+1	$z+1^{++}$	z+2	$z+2^{++}$	#
1	138.0662	69.5367					155.0927	78.0500	H													19
2	322.1986	161.6029	305.1721	153.0897			339.2251	170.1162	R	2032.2138		2162.3356	1081.6715	2145.3091	1073.1582	2144.3251	1072.6662	2146.3169	1073.6621	2147.3248	1074.1660	18
3	450.2936	225.6504	433.2670	217.1371			467.3201	234.1637	K	1904.1188		1978.2032	989.6053	1961.1767	981.0920	1960.1927	980.6000	1962.1845	981.5959	1963.1923	982.0998	17
4	549.3620	275.1846	532.3354	266.6714			566.3885	283.6979	V	1819.0661		1850.1083	925.5578	1833.0817	917.0445	1832.0977	916.5525	1834.0896	917.5484	1835.0974	918.0523	16
5	662.4460	331.7267	645.4195	323.2134			679.4726	340.2399	L	1691.9664		1751.0399	876.0236	1734.0133	867.5103	1733.0293	867.0183	1735.0211	868.0142	1736.0290	868.5181	15
6	818.5472	409.7772	801.5206	401.2639			835.5737	418.2905	R	1535.8653		1637.9558	819.4815	1620.9292	810.9683	1619.9452	810.4763	1621.9371	811.4722	1622.9449	811.9761	14
7	933.5741	467.2907	916.5475	458.7774	915.5635	458.2854	950.6006	475.8040	D	1420.8383		1481.8547	741.4310	1464.8281	732.9177	1463.8441	732.4257	1465.8360	733.4216	1466.8438	733.9255	13
8	1047.6170	524.3121	1030.5905	515.7989	1029.6065	515.3069	1064.6436	532.8254	N	1306.7954		1366.8277	683.9175	1349.8012	675.4042	1348.8172	674.9122	1350.8090	675.9081	1351.8168	676.4121	12
9	1160.7011	580.8542	1143.6745	572.3409	1142.6905	571.8489	1177.7276	589.3675	I	1207.7270	1221.7426	1252.7848	626.8960	1235.7583	618.3828	1234.7743	617.8908	1236.7661	618.8867	1237.7739	619.3906	11
10	1288.7597	644.8835	1271.7331	636.3702	1270.7491	635.8782	1305.7862	653.3967	Q	1065.6527		1139.7008	570.3540	1122.6742	561.8407	1121.6902	561.3487	1123.6820	562.3447	1124.6899	562.8486	10
11	1345.7811	673.3942	1328.7546	664.8809	1327.7706	664.3889	1362.8077	681.9075	G			1011.6422	506.3247	994.6156	497.8115	993.6316	497.3194	995.6235	498.3154	996.6313	498.8193	9
12	1458.8652	729.9362	1441.8386	721.4230	1440.8546	720.9310	1475.8917	738.4495	I	909.5629	923.5785	954.6207	477.8140	937.5942	469.3007	936.6101	468.8087	938.6020	469.8046	939.6098	470.3085	8
13	1559.9129	780.4601	1542.8863	771.9468	1541.9023	771.4548	1576.9394	788.9733	T	808.5152	810.4944	841.5366	421.2720	824.5101	412.7587	823.5261	412.2667	825.5179	413.2626	826.5257	413.7665	7
14	1688.0078	844.5076	1670.9813	835.9943	1669.9973	835.5023	1705.0344	853.0208	K	666.4046		740.4890	370.7481	723.4624	362.2348			724.4702	362.7388	725.4781	363.2427	6
15	1785.0606	893.0339	1768.0340	884.5207	1767.0500	884.0287	1802.0871	901.5472	P	569.3518		612.3940	306.7006	595.3675	298.1874			596.3753	298.6913	597.3831	299.1952	5
16	1856.0977	928.5525	1839.0712	920.0392	1838.0871	919.5472	1873.1243	937.0658	A			515.3412	258.1743	498.3147	249.6610			499.3225	250.1649	500.3303	250.6688	4
17	1969.1818	985.0945	1952.1552	976.5813	1951.1712	976.0892	1986.2083	993.6078	I	399.2463	413.2619	444.3041	222.6557	427.2776	214.1424			428.2854	214.6463	429.2932	215.1503	3
18	2125.2829	1063.1451	2108.2563	1054.6318	2107.2723	1054.1398	2142.3094	1071.6584	R	229.1295		331.2201	166.1137	314.1935	157.6004			315.2013	158.1043	316.2092	158.6082	2
19									R	73.0284		175.1190	88.0631	158.0924	79.5498			159.1002	80.0538	160.1081	80.5577	1

Monoisotopic mass of neutral peptide Mr(calc): 2298.3872
Fixed modifications: Carbamidomethyl (C) (apply to specified residues or termini only)
Variable modifications:
R2 : Dimethyl_KR (KR)
Ions Score: 38 **Expect:** 0.0096
Matches : 56/318 fragment ions using 75 most intense peaks (help)

Mascot Search Results: Peptide View

HRme2KVLRDNIQGITKPAIRR

MS/MS Fragmentation of HRKVLRDNIQGITKPAIRR
Found in sp|P62806|H4_MOUSE in fgcz_10090, Histone H4 OS=Mus musculus GN=Hist1h4a

Match to Query 4895: 2312.401720 from(463.487620,5+) intensity(248928.0800) scans(2816) rtinseconds(2118.4265) index(4168)
Title: 1142: Scan 2816 (rt=35.3071) [s:\p749\Proteomics\ORBI_2\andreamizzi_20110310_sperm_trypsin_chymo_AspN_ETD_CID\20110311_34_NaCl_trypsin_SCX_FT.RAW]
Data file Y:\p749\Proteomics\Mascot\mgf\50 20110426 sperm rerun trypsin 2nd ETD run NaCl SCX\mascot_daemon_merge.mgf

Click mouse within plot area to zoom in by factor of two about that point
Or, Plot from 100 to 1500 Da Full range
Label all possible matches ○ Label matches used for scoring ◉
Show Y-axis ☐

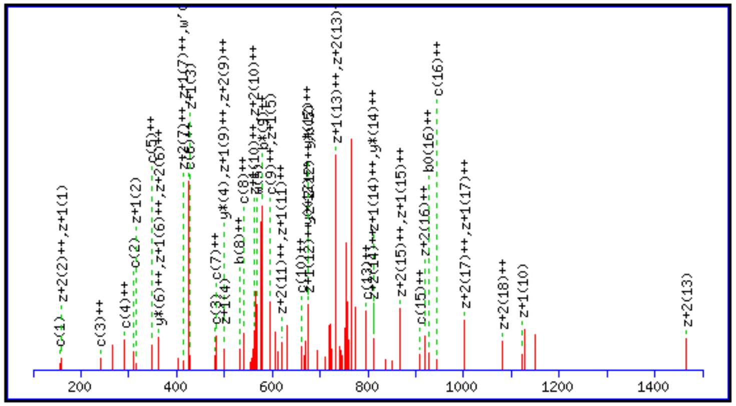

#	b	b++	b*	b*++	b0	b0++	c	c++	Seq.	w	w'	y	y++	y*	y*++	y0	y0++	z+1	z+1++	z+2	z+2++	#
1	138.0662	69.5367					155.0927	78.0500	H													19
2	294.1673	147.5873	277.1408	139.0740			311.1938	156.1006	R	2074.2608		2176.3513	1088.6793	2159.3248	1080.1660	2158.3407	1079.6740	2160.3326	1080.6699	2161.3404	1081.1738	18
3	464.3092	232.6582	447.2827	224.1450			481.3358	241.1715	K	1904.1188		2020.2502	1010.6287	2003.2236	1002.1155	2002.2396	1001.6234	2004.2315	1002.6194	2005.2393	1003.1233	17
4	563.3776	282.1925	546.3511	273.6792			580.4042	290.7057	V	1819.0661		1850.1083	925.5578	1833.0817	917.0445	1832.0977	916.5525	1834.0896	917.5484	1835.0974	918.0523	16
5	676.4617	338.7345	659.4351	330.2212			693.4882	347.2478	L	1691.9664		1751.0399	876.0236	1734.0133	867.5103	1733.0293	867.0183	1735.0211	868.0142	1736.0290	868.5181	15
6	832.5628	416.7850	815.5363	408.2718			849.5894	425.2983	R	1535.8653		1637.9558	819.4815	1620.9292	810.9683	1619.9452	810.4763	1621.9371	811.4722	1622.9449	811.9761	14
7	947.5897	474.2985	930.5632	465.7852	929.5792	465.2932	964.6163	482.8118	D	1420.8383		1481.8547	741.4310	1464.8281	732.9177	1463.8441	732.4257	1465.8360	733.4216	1466.8438	733.9255	13
8	1061.6327	531.3200	1044.6061	522.8067	1043.6221	522.3147	1078.6592	539.8332	N	1306.7954		1366.8277	683.9175	1349.8012	675.4042	1348.8172	674.9122	1350.8090	675.9081	1351.8168	676.4121	12
9	1174.7167	587.8620	1157.6902	579.3487	1156.7062	578.8567	1191.7433	596.3753	I	1207.7270	1221.7426	1252.7848	626.8960	1235.7583	618.3828	1234.7743	617.8908	1236.7661	618.8867	1237.7739	619.3906	11
10	1302.7753	651.8913	1285.7488	643.3780	1284.7647	642.8860	1319.8019	660.4046	Q	1065.6527		1139.7008	570.3540	1122.6742	561.8407	1121.6902	561.3487	1123.6820	562.3447	1124.6899	562.8486	10
11	1359.7968	680.4020	1342.7702	671.8888	1341.7862	671.3967	1376.8233	688.9153	G			1011.6422	506.3247	994.6156	497.8115	993.6316	497.3194	995.6235	498.3154	996.6313	498.8193	9
12	1472.8808	736.9441	1455.8543	728.4308	1454.8703	727.9388	1489.9074	745.4573	I	909.5629	923.5785	954.6207	477.8140	937.5942	469.3007	936.6101	468.8087	938.6020	469.8046	939.6098	470.3085	8
13	1573.9285	787.4679	1556.9020	778.9546	1555.9180	778.4626	1590.9551	795.9812	T	808.5152	810.4944	841.5366	421.2720	824.5101	412.7587	823.5261	412.2667	825.5179	413.2626	826.5257	413.7665	7
14	1702.0235	851.5154	1684.9969	843.0021	1684.0129	842.5101	1719.0500	860.0287	K	666.4046		740.4890	370.7481	723.4624	362.2348			724.4702	362.7388	725.4781	363.2427	6
15	1799.0762	900.0418	1782.0497	891.5285	1781.0657	891.0365	1816.1028	908.5550	P	569.3518		612.3940	306.7006	595.3675	298.1874			596.3753	298.6913	597.3831	299.1952	5
16	1870.1134	935.5603	1853.0868	927.0470	1852.1028	926.5550	1887.1399	944.0736	A			515.3412	258.1743	498.3147	249.6610			499.3225	250.1649	500.3303	250.6688	4
17	1983.1974	992.1024	1966.1709	983.5891	1965.1869	983.0971	2000.2240	1000.6156	I	399.2463	413.2619	444.3041	222.6557	427.2776	214.1424			428.2854	214.6463	429.2932	215.1503	3
18	2139.2985	1070.1529	2122.2720	1061.6396	2121.2880	1061.1476	2156.3251	1078.6662	R	229.1295		331.2201	166.1137	314.1935	157.6004			315.2013	158.1043	316.2092	158.6082	2
19									R	73.0284		175.1190	88.0631	158.0924	79.5498			159.1002	80.0538	160.1081	80.5577	1

Monoisotopic mass of neutral peptide Mr(calc): 2312.4029
Fixed modifications: Carbamidomethyl (C) (apply to specified residues or termini only)
Variable modifications:
K3 : Trimethyl (K)
Ions Score: 75 **Expect:** 3.9e-05
Matches : 55/318 fragment ions using 63 most intense peaks (help)

Mascot Search Results: Peptide View

HRKme3VLRDNIQGITKPAIRR

MS/MS Fragmentation of HRKVLRDNIQGITKPAIRR
Found in sp|P62806|H4_MOUSE in fgcz_10090, Histone H4 OS=Mus musculus GN=Hist1h4a

Match to Query 3669: 2298.376720 from(460.682620,5+) intensity(315840.6300) scans(3829) rtinseconds(2173.965) index(2452)
Title: 2453: Scan 3829 (rt=36.2328) [s:\p749\Proteomics\ORBI_2\andreamizzi_20110517_sperm_trypsin_GluC_ETD_CID\20110517_01_NaCl_1.RAW]
Data file 20110517_01_NaCl_1.mgf

Click mouse within plot area to zoom in by factor of two about that point
Or, Plot from 100 to 1500 Da Full range
Label all possible matches ○ Label matches used for scoring ◉
Show Y-axis ☐

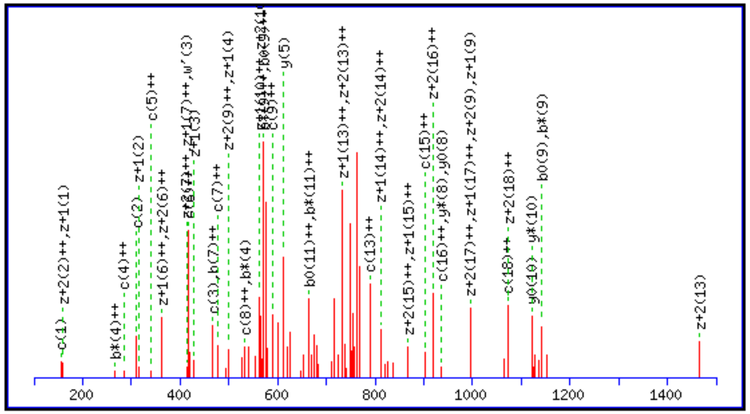

#	b	b^{++}	b^{*}	b^{*++}	b^{0}	b^{0++}	c	c^{++}	Seq.	w	w'	y	y^{++}	y^{*}	y^{*++}	y^{0}	y^{0++}	z+1	$z+1^{++}$	z+2	$z+2^{++}$	#
1	138.0662	69.5367					155.0927	78.0500	H													19
2	294.1673	147.5873	277.1408	139.0740			311.1938	156.1006	R	2060.2451		2162.3356	1081.6715	2145.3091	1073.1582	2144.3251	1072.6662	2146.3169	1073.6621	2147.3248	1074.1660	18
3	450.2936	225.6504	433.2670	217.1371			467.3201	234.1637	K	1904.1188		2006.2345	1003.6209	1989.2080	995.1076	1988.2240	994.6156	1990.2158	995.6115	1991.2236	996.1155	17
4	549.3620	275.1846	532.3354	266.6714			566.3885	283.6979	V	1819.0661		1850.1083	925.5578	1833.0817	917.0445	1832.0977	916.5525	1834.0896	917.5484	1835.0974	918.0523	16
5	662.4460	331.7267	645.4195	323.2134			679.4726	340.2399	L	1691.9664		1751.0399	876.0236	1734.0133	867.5103	1733.0293	867.0183	1735.0211	868.0142	1736.0290	868.5181	15
6	818.5472	409.7772	801.5206	401.2639			835.5737	418.2905	R	1535.8653		1637.9558	819.4815	1620.9292	810.9683	1619.9452	810.4763	1621.9371	811.4722	1622.9449	811.9761	14
7	933.5741	467.2907	916.5475	458.7774	915.5635	458.2854	950.6006	475.8040	D	1420.8383		1481.8547	741.4310	1464.8281	732.9177	1463.8441	732.4257	1465.8360	733.4216	1466.8438	733.9255	13
8	1047.6170	524.3121	1030.5905	515.7989	1029.6065	515.3069	1064.6436	532.8254	N	1306.7954		1366.8277	683.9175	1349.8012	675.4042	1348.8172	674.9122	1350.8090	675.9081	1351.8168	676.4121	12
9	1160.7011	580.8542	1143.6745	572.3409	1142.6905	571.8489	1177.7276	589.3675	I	1207.7270	1221.7426	1252.7848	626.8960	1235.7583	618.3828	1234.7743	617.8908	1236.7661	618.8867	1237.7739	619.3906	11
10	1288.7597	644.8835	1271.7331	636.3702	1270.7491	635.8782	1305.7862	653.3967	Q	1065.6527		1139.7008	570.3540	1122.6742	561.8407	1121.6902	561.3487	1123.6820	562.3447	1124.6899	562.8486	10
11	1345.7811	673.3942	1328.7546	664.8809	1327.7706	664.3889	1362.8077	681.9075	G			1011.6422	506.3247	994.6156	497.8115	993.6316	497.3194	995.6235	498.3154	996.6313	498.8193	9
12	1458.8652	729.9362	1441.8386	721.4230	1440.8546	720.9310	1475.8917	738.4495	I	909.5629	923.5785	954.6207	477.8140	937.5942	469.3007	936.6101	468.8087	938.6020	469.8046	939.6098	470.3085	8
13	1559.9129	780.4601	1542.8863	771.9468	1541.9023	771.4548	1576.9394	788.9733	T	808.5152	810.4944	841.5366	421.2720	824.5101	412.7587	823.5261	412.2667	825.5179	413.2626	826.5257	413.7665	7
14	1688.0078	844.5076	1670.9813	835.9943	1669.9973	835.5023	1705.0344	853.0208	K	666.4046		740.4890	370.7481	723.4624	362.2348			724.4702	362.7388	725.4781	363.2427	6
15	1785.0606	893.0339	1768.0340	884.5207	1767.0500	884.0287	1802.0871	901.5472	P	569.3518		612.3940	306.7006	595.3675	298.1874			596.3753	298.6913	597.3831	299.1952	5
16	1856.0977	928.5525	1839.0712	920.0392	1838.0871	919.5472	1873.1243	937.0658	A			515.3412	258.1743	498.3147	249.6610			499.3225	250.1649	500.3303	250.6688	4
17	1969.1818	985.0945	1952.1552	976.5813	1951.1712	976.0892	1986.2083	993.6078	I	399.2463	413.2619	444.3041	222.6557	427.2776	214.1424			428.2854	214.6463	429.2932	215.1503	3
18	2125.2829	1063.1451	2108.2563	1054.6318	2107.2723	1054.1398	2142.3094	1071.6584	R	229.1295		331.2201	166.1137	314.1935	157.6004			315.2013	158.1043	316.2092	158.6082	2
19									R	73.0284		175.1190	88.0631	158.0924	79.5498			159.1002	80.0538	160.1081	80.5577	1

Monoisotopic mass of neutral peptide Mr(calc): 2298.3872
Fixed modifications: Carbamidomethyl (C) (apply to specified residues or termini only)
Variable modifications:
K3 : Dimethyl_KR (KR)
Ions Score: 76 **Expect:** 5.5e-05
Matches : 53/318 fragment ions using 44 most intense peaks (help)

Mascot Search Results: Peptide View

HRKme2VLRDNIQGITKPAIRR

MS/MS Fragmentation of KVLRDNIQGITKPAIR
Found in sp|P62806|H4_MOUSE in fgcz_10090, Histone H4 OS=Mus musculus GN=Hist1h4a

Match to Query 2310: 1835.109856 from(459.784740,4+) intensity(620718.1300) scans(3597) rtinseconds(2041.9003) index(1995)
Title: 1996: Scan 3597 (rt=34.0317) [s:\p749\Proteomics\ORBI_2\andreamizzi_201110127_sperm_trypsin_ETD_CID\20110127_08_ON7_saltex.RAW]
Data file \\fgcz-ms.uzh.ch\p749\Proteomics\ORBI_2\andreamizzi_20110127_sperm_trypsin_ETD_CID\mgf__low_res_MS2_nomerge\20110127_08_ON7_saltex.mgf

Click mouse within plot area to zoom in by factor of two about that point
Or, Plot from 100 to 1700 Da Full range
Label all possible matches ○ Label matches used for scoring ◉
Show Y-axis ☐

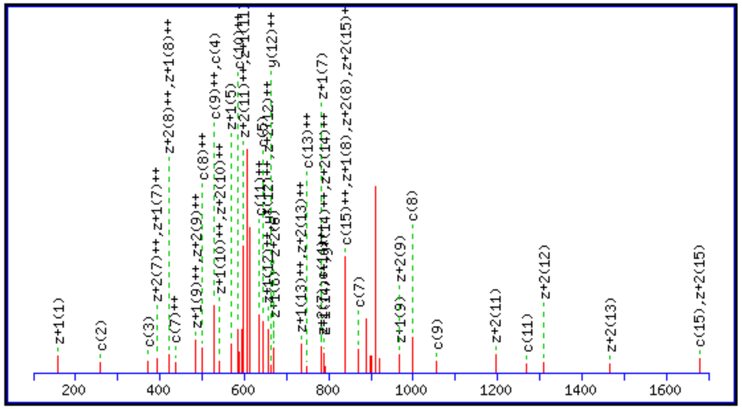

#	b	b^{++}	b^{*}	b^{*++}	b^{0}	b^{0++}	c	c^{++}	Seq.	w	w'	y	y^{++}	y^{*}	y^{*++}	y^{0}	y^{0++}	z+1	$z+1^{++}$	z+2	$z+2^{++}$	#
1	143.1179	72.0626	126.0913	63.5493			160.1444	80.5759	K													16
2	242.1863	121.5968	225.1598	113.0835			259.2129	130.1101	V	1662.9650		1694.0072	847.5072	1676.9806	838.9939	1675.9966	838.5019	1677.9884	839.4979	1678.9963	840.0018	15
3	355.2704	178.1388	338.2438	169.6255			372.2969	186.6521	L	1535.8653		1594.9388	797.9730	1577.9122	789.4597	1576.9282	788.9677	1578.9200	789.9637	1579.9279	790.4676	14
4	511.3715	256.1894	494.3449	247.6761			528.3980	264.7027	R	1379.7641		1481.8547	741.4310	1464.8281	732.9177	1463.8441	732.4257	1465.8360	733.4216	1466.8438	733.9255	13
5	626.3984	313.7028	609.3719	305.1896	608.3879	304.6976	643.4250	322.2161	D	1264.7372		1325.7536	663.3804	1308.7270	654.8672	1307.7430	654.3751	1309.7349	655.3711	1310.7427	655.8750	12
6	740.4413	370.7243	723.4148	362.2110	722.4308	361.7190	757.4679	379.2376	N	1150.6943		1210.7266	605.8670	1193.7001	597.3537	1192.7161	596.8617	1194.7079	597.8576	1195.7157	598.3615	11
7	853.5254	427.2663	836.4989	418.7531	835.5148	418.2611	870.5520	435.7796	I	1051.6259	1065.6415	1096.6837	548.8455	1079.6572	540.3322	1078.6731	539.8402	1080.6650	540.8361	1081.6728	541.3400	10
8	981.5840	491.2956	964.5574	482.7824	963.5734	482.2904	998.6105	499.8089	Q	909.5516		983.5996	492.3035	966.5731	483.7902	965.5891	483.2982	967.5809	484.2941	968.5887	484.7980	9
9	1038.6055	519.8064	1021.5789	511.2931	1020.5949	510.8011	1055.6320	528.3196	G			855.5411	428.2742	838.5145	419.7609	837.5305	419.2689	839.5223	420.2648	840.5302	420.7687	8
10	1151.6895	576.3484	1134.6630	567.8351	1133.6790	567.3431	1168.7161	584.8617	I	753.4618	767.4774	798.5196	399.7634	781.4931	391.2502	780.5090	390.7582	782.5009	391.7541	783.5087	392.2580	7
11	1252.7372	626.8722	1235.7106	618.3590	1234.7266	617.8670	1269.7637	635.3855	T	652.4141	654.3933	685.4355	343.2214	668.4090	334.7081	667.4250	334.2161	669.4168	335.2120	670.4246	335.7160	6
12	1380.8322	690.9197	1363.8056	682.4064	1362.8216	681.9144	1397.8587	699.4330	K	510.3035		584.3879	292.6976	567.3613	284.1843			568.3691	284.6882	569.3770	285.1921	5
13	1477.8849	739.4461	1460.8584	730.9328	1459.8744	730.4408	1494.9115	747.9594	P	413.2507		456.2929	228.6501	439.2663	220.1368			440.2742	220.6407	441.2820	221.1446	4
14	1548.9220	774.9647	1531.8955	766.4514	1530.9115	765.9594	1565.9486	783.4779	A			359.2401	180.1237	342.2136	171.6104			343.2214	172.1143	344.2292	172.6183	3
15	1662.0061	831.5067	1644.9796	822.9934	1643.9955	822.5014	1679.0327	840.0200	I	243.1452	257.1608	288.2030	144.6051	271.1765	136.0919			272.1843	136.5958	273.1921	137.0997	2
16									R	73.0284		175.1190	88.0631	158.0924	79.5498			159.1002	80.0538	160.1081	80.5577	1

Monoisotopic mass of neutral peptide Mr(calc): 1835.1105
Fixed modifications: Carbamidomethyl (C) (apply to specified residues or termini only)
Variable modifications:
K1 : Methyl_KR (KR)
Ions Score: 84 **Expect:** 6.7e-06
Matches : 52/269 fragment ions using 46 most intense peaks (help)

Mascot Search Results: Peptide View

Kme1VLRDNIQGITKPAIR

MS/MS Fragmentation of **KVLRDNIQGITKPAIR**
Found in **sp|P62806|H4_MOUSE** in **fgcz_10090**, Histone H4 OS=Mus musculus GN=Hist1h4a

Match to Query 3895: 1863.141176 from(466.792570,4+) intensity(80800.3440) scans(3445) rtinseconds(2382.7402) index(1605)
Title: 1461: Scan 3445 (rt=39.7123) [s:\p749\Proteomics\ORBI_2\andreamizzi_20110310_sperm_trypsin_chymo_AspN_ETD_CID\20110311_37_NaCl_trypsin_SCX_3.RAW]
Data file Y:\p749\Proteomics\Mascot\mgf\50 20110426 sperm rerun trypsin 2nd ETD run NaCl SCX\mascot_daemon_merge.mgf

Click mouse within plot area to zoom in by factor of two about that point
Or, Plot from 100 to 1900 Da Full range
Label all possible matches ○ Label matches used for scoring ◉
Show Y-axis ☐

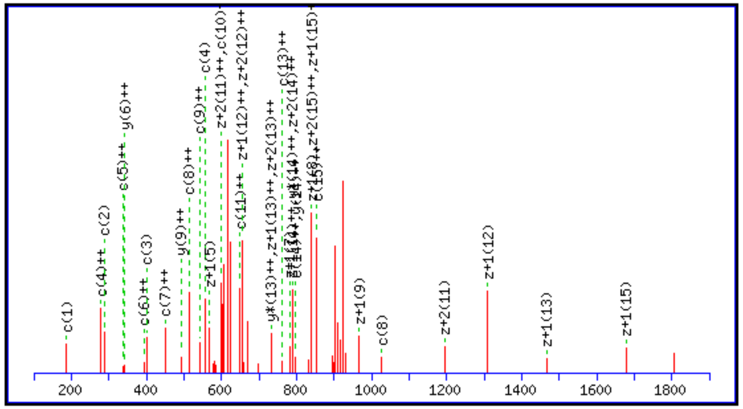

#	b	b^{++}	b^{*}	b^{*++}	b^{0}	b^{0++}	c	c^{++}	Seq.	w	w'	y	y^{++}	y^{*}	y^{*++}	y^{0}	y^{0++}	z+1	$z+1^{++}$	z+2	$z+2^{++}$	#
1	171.1492	86.0782	154.1226	77.5650			188.1757	94.5915	K													16
2	270.2176	135.6124	253.1911	127.0992			287.2442	144.1257	V	1662.9650		1694.0072	847.5072	1676.9806	838.9939	1675.9966	838.5019	1677.9884	839.4979	1678.9963	840.0018	15
3	383.3017	192.1545	366.2751	183.6412			400.3282	200.6677	L	1535.8653		1594.9388	797.9730	1577.9122	789.4597	1576.9282	788.9677	1578.9200	789.9637	1579.9279	790.4676	14
4	539.4028	270.2050	522.3762	261.6918			556.4293	278.7183	R	1379.7641		1481.8547	741.4310	1464.8281	732.9177	1463.8441	732.4257	1465.8360	733.4216	1466.8438	733.9255	13
5	654.4297	327.7185	637.4032	319.2052	636.4192	318.7132	671.4563	336.2318	D	1264.7372		1325.7536	663.3804	1308.7270	654.8672	1307.7430	654.3751	1309.7349	655.3711	1310.7427	655.8750	12
6	768.4726	384.7400	751.4461	376.2267	750.4621	375.7347	785.4992	393.2532	N	1150.6943		1210.7266	605.8670	1193.7001	597.3537	1192.7161	596.8617	1194.7079	597.8576	1195.7157	598.3615	11
7	881.5567	441.2820	864.5302	432.7687	863.5461	432.2767	898.5833	449.7953	I	1051.6259	1065.6415	1096.6837	548.8455	1079.6572	540.3322	1078.6731	539.8402	1080.6650	540.8361	1081.6728	541.3400	10
8	1009.6153	505.3113	992.5887	496.7980	991.6047	496.3060	1026.6418	513.8246	Q	909.5516		983.5996	492.3035	966.5731	483.7902	965.5891	483.2982	967.5809	484.2941	968.5887	484.7980	9
9	1066.6368	533.8220	1049.6102	525.3087	1048.6262	524.8167	1083.6633	542.3353	G			855.5411	428.2742	838.5145	419.7609	837.5305	419.2689	839.5223	420.2648	840.5302	420.7687	8
10	1179.7208	590.3640	1162.6943	581.8508	1161.7103	581.3588	1196.7474	598.8773	I	753.4618	767.4774	798.5196	399.7634	781.4931	391.2502	780.5090	390.7582	782.5009	391.7541	783.5087	392.2580	7
11	1280.7685	640.8879	1263.7419	632.3746	1262.7579	631.8826	1297.7950	649.4012	T	652.4141	654.3933	685.4355	343.2214	668.4090	334.7081	667.4250	334.2161	669.4168	335.2120	670.4246	335.7160	6
12	1408.8635	704.9354	1391.8369	696.4221	1390.8529	695.9301	1425.8900	713.4486	K	510.3035		584.3879	292.6976	567.3613	284.1843			568.3691	284.6882	569.3770	285.1921	5
13	1505.9162	753.4618	1488.8897	744.9485	1487.9057	744.4565	1522.9428	761.9750	P	413.2507		456.2929	228.6501	439.2663	220.1368			440.2742	220.6407	441.2820	221.1446	4
14	1576.9533	788.9803	1559.9268	780.4670	1558.9428	779.9750	1593.9799	797.4936	A			359.2401	180.1237	342.2136	171.6104			343.2214	172.1143	344.2292	172.6183	3
15	1690.0374	845.5223	1673.0109	837.0091	1672.0268	836.5171	1707.0640	854.0356	I	243.1452	257.1608	288.2030	144.6051	271.1765	136.0919			272.1843	136.5958	273.1921	137.0997	2
16									R	73.0284		175.1190	88.0631	158.0924	79.5498			159.1002	80.0538	160.1081	80.5577	1

Monoisotopic mass of neutral peptide Mr(calc): 1863.1418
Fixed modifications: Carbamidomethyl (C) (apply to specified residues or termini only)
Variable modifications:
K1 : Trimethyl (K)
Ions Score: 84 **Expect:** 5.4e-06
Matches : 39/269 fragment ions using 44 most intense peaks (help)

Mascot Search Results: Peptide View

Kme3VLRDNIQGITKPAIR

MS/MS Fragmentation of KVLRDNIQGITKPAIR
Found in sp|P62806|H4_MOUSE in fgcz_10090, Histone H4 OS=Mus musculus GN=Hist1h4a

Match to Query 2466: 1863.139336 from(466.792110,4+) intensity(1156701.1000) scans(3847) rtinseconds(2260.0869) index(2233)
Title: 2234: Scan 3847 (rt=37.6681) [s:\p749\Proteomics\ORBI_2\andreamizzi_20110310_sperm_trypsin_chymo_AspN_ETD_CID\20110310_05_rep_ON7.RAW]
Data file \\fgcz-ms.uzh.ch\p749\Proteomics\ORBI_2\andreamizzi_20110310_sperm_trypsin_chymo_AspN_ETD_CID\mgf__low_res_MS2_nomerge\20110310_05_rep_ON7.mgf

Click mouse within plot area to zoom in by factor of two about that point
Or, Plot from 150 to 1150 Da Full range
Label all possible matches ○ Label matches used for scoring ◉
Show Y-axis ☐

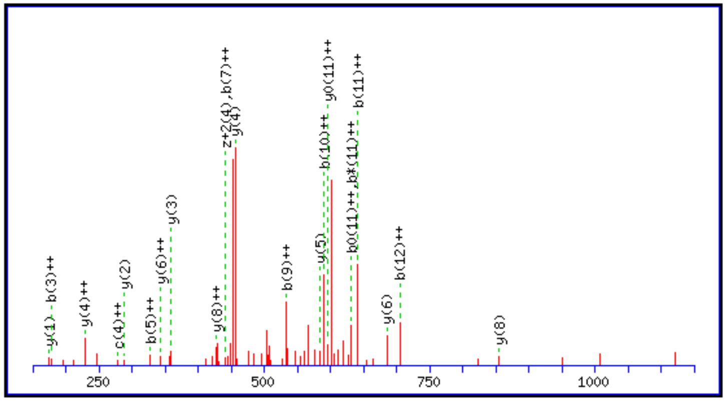

#	b	b++	b*	b*++	b0	b0++	c	c++	Seq.	w	w'	y	y++	y*	y*++	y0	y0++	z+1	z+1++	z+2	z+2++	#
1	143.1179	72.0626	126.0913	63.5493			160.1444	80.5759	K													16
2	242.1863	121.5968	225.1598	113.0835			259.2129	130.1101	V	1690.9963		1722.0385	861.5229	1705.0119	853.0096	1704.0279	852.5176	1706.0197	853.5135	1707.0276	854.0174	15
3	355.2704	178.1388	338.2438	169.6255			372.2969	186.6521	L	1563.8966		1622.9701	811.9887	1605.9435	803.4754	1604.9595	802.9834	1606.9513	803.9793	1607.9592	804.4832	14
4	539.4028	270.2050	522.3762	261.6918			556.4293	278.7183	R	1379.7641		1509.8860	755.4466	1492.8594	746.9334	1491.8754	746.4413	1493.8673	747.4373	1494.8751	747.9412	13
5	654.4297	327.7185	637.4032	319.2052	636.4192	318.7132	671.4563	336.2318	D	1264.7372		1325.7536	663.3804	1308.7270	654.8672	1307.7430	654.3751	1309.7349	655.3711	1310.7427	655.8750	12
6	768.4726	384.7400	751.4461	376.2267	750.4621	375.7347	785.4992	393.2532	N	1150.6943		1210.7266	605.8670	1193.7001	597.3537	1192.7161	596.8617	1194.7079	597.8576	1195.7157	598.3615	11
7	881.5567	441.2820	864.5302	432.7687	863.5461	432.2767	898.5833	449.7953	I	1051.6259	1065.6415	1096.6837	548.8455	1079.6572	540.3322	1078.6731	539.8402	1080.6650	540.8361	1081.6728	541.3400	10
8	1009.6153	505.3113	992.5887	496.7980	991.6047	496.3060	1026.6418	513.8246	Q	909.5516		983.5996	492.3035	966.5731	483.7902	965.5891	483.2982	967.5809	484.2941	968.5887	484.7980	9
9	1066.6368	533.8220	1049.6102	525.3087	1048.6262	524.8167	1083.6633	542.3353	G			855.5411	428.2742	838.5145	419.7609	837.5305	419.2689	839.5223	420.2648	840.5302	420.7687	8
10	1179.7208	590.3640	1162.6943	581.8508	1161.7103	581.3588	1196.7474	598.8773	I	753.4618	767.4774	798.5196	399.7634	781.4931	391.2502	780.5090	390.7582	782.5009	391.7541	783.5087	392.2580	7
11	1280.7685	640.8879	1263.7419	632.3746	1262.7579	631.8826	1297.7950	649.4012	T	652.4141	654.3933	685.4355	343.2214	668.4090	334.7081	667.4250	334.2161	669.4168	335.2120	670.4246	335.7160	6
12	1408.8635	704.9354	1391.8369	696.4221	1390.8529	695.9301	1425.8900	713.4486	K	510.3035		584.3879	292.6976	567.3613	284.1843			568.3691	284.6882	569.3770	285.1921	5
13	1505.9162	753.4618	1488.8897	744.9485	1487.9057	744.4565	1522.9428	761.9750	P	413.2507		456.2929	228.6501	439.2663	220.1368			440.2742	220.6407	441.2820	221.1446	4
14	1576.9533	788.9803	1559.9268	780.4670	1558.9428	779.9750	1593.9799	797.4936	A			359.2401	180.1237	342.2136	171.6104			343.2214	172.1143	344.2292	172.6183	3
15	1690.0374	845.5223	1673.0109	837.0091	1672.0268	836.5171	1707.0640	854.0356	I	243.1452	257.1608	288.2030	144.6051	271.1765	136.0919			272.1843	136.5958	273.1921	137.0997	2
16									R	73.0284		175.1190	88.0631	158.0924	79.5498			159.1002	80.0538	160.1081	80.5577	1

Monoisotopic mass of neutral peptide Mr(calc): 1863.1418
Fixed modifications: Carbamidomethyl (C) (apply to specified residues or termini only)
Variable modifications:
K1 : Methyl_KR (KR)
R4 : Dimethyl_KR (KR)
Ions Score: 35 **Expect:** 0.042
Matches : 23/269 fragment ions using 48 most intense peaks (help)

Mascot Search Results: Peptide View

Kme1VLRme2DNIQGITKPAIR

MS/MS Fragmentation of KVLRDNIQGITKPAIR
Found in sp|P62806|H4_MOUSE in fgcz_10090, Histone H4 OS=Mus musculus GN=Hist1h4a

Match to Query 3077: 1849.125492 from(617.382440,3+) intensity(522432.8100) scans(4360) rtinseconds(2417.348) index(2873)
Title: 2874: Scan 4360 (rt=40.2891) [s:\p749\Proteomics\ORBI_2\andreamizzi_20110517_sperm_trypsin_GluC_ETD_CID\20110517_01_NaCl_1.RAW]
Data file 20110517_01_NaCl_1.mgf

Click mouse within plot area to zoom in by factor of two about that point
Or, Plot from 200 to 1900 Da Full range
Label all possible matches ○ Label matches used for scoring ◉
Show Y-axis ☐

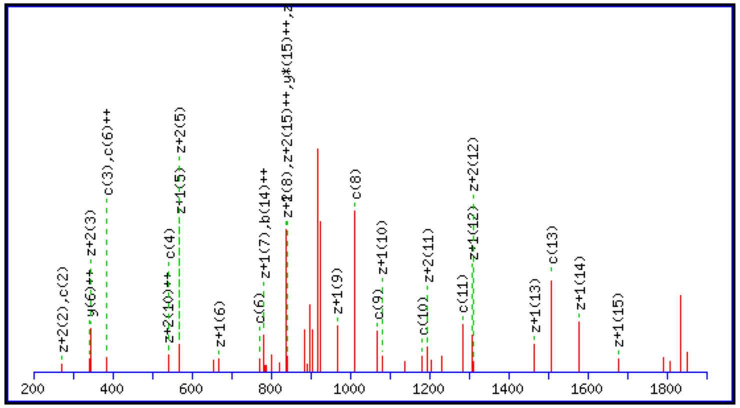

#	b	b^{++}	b*	b^{*++}	b^0	b^{0++}	c	c^{++}	Seq.	w	w'	y	y^{++}	y*	y^{*++}	y^0	y^{0++}	z+1	$z+1^{++}$	z+2	$z+2^{++}$	#
1	157.1335	79.0704	140.1070	70.5571			174.1601	87.5837	K													16
2	256.2020	128.6046	239.1754	120.0913			**273.2285**	137.1179	V	1662.9650		1694.0072	847.5072	1676.9806	838.9939	1675.9966	838.5019	***1677.9884***	839.4979	1678.9963	840.0018	15
3	369.2860	185.1466	352.2595	176.6334			**386.3126**	193.6599	L	1535.8653		1594.9388	797.9730	1577.9122	789.4597	1576.9282	788.9677	***1578.9200***	789.9637	1579.9279	790.4676	14
4	525.3871	263.1972	508.3606	254.6839			**542.4137**	271.7105	R	1379.7641		1481.8547	741.4310	1464.8281	732.9177	1463.8441	732.4257	***1465.8360***	733.4216	1466.8438	733.9255	13
5	640.4141	320.7107	623.3875	312.1974	622.4035	311.7054	657.4406	329.2239	D	1264.7372		1325.7536	663.3804	1308.7270	654.8672	1307.7430	654.3751	***1309.7349***	655.3711	**1310.7427**	655.8750	12
6	754.4570	377.7321	737.4304	369.2189	736.4464	368.7269	**771.4835**	386.2454	N	1150.6943		1210.7266	605.8670	1193.7001	597.3537	1192.7161	596.8617	1194.7079	597.8576	**1195.7157**	598.3615	11
7	867.5411	434.2742	850.5145	425.7609	849.5305	425.2689	884.5676	442.7874	I	1051.6259	1065.6415	1096.6837	548.8455	1079.6572	540.3322	1078.6731	539.8402	***1080.6650***	540.8361	1081.6728	541.3400	10
8	995.5996	498.3035	978.5731	489.7902	977.5891	489.2982	**1012.6262**	506.8167	Q	909.5516		983.5996	492.3035	966.5731	483.7902	965.5891	483.2982	***967.5809***	484.2941	968.5887	484.7980	9
9	1052.6211	526.8142	1035.5946	518.3009	1034.6105	517.8089	**1069.6477**	535.3275	G			855.5411	428.2742	838.5145	419.7609	837.5305	419.2689	***839.5223***	420.2648	**840.5302**	420.7687	8
10	1165.7052	583.3562	1148.6786	574.8429	1147.6946	574.3509	**1182.7317**	591.8695	I	753.4618	767.4774	798.5196	399.7634	781.4931	391.2502	780.5090	390.7582	***782.5009***	391.7541	783.5087	392.2580	7
11	1266.7528	633.8801	1249.7263	625.3668	1248.7423	624.8748	**1283.7794**	642.3933	T	652.4141	654.3933	685.4355	343.2214	668.4090	334.7081	667.4250	334.2161	***669.4168***	335.2120	670.4246	335.7160	6
12	1394.8478	697.9275	1377.8213	689.4143	1376.8372	688.9223	1411.8744	706.4408	K	510.3035		584.3879	292.6976	567.3613	284.1843			***568.3691***	284.6882	**569.3770**	285.1921	5
13	1491.9006	746.4539	1474.8740	737.9407	1473.8900	737.4486	**1508.9271**	754.9672	P	413.2507		456.2929	228.6501	439.2663	220.1368			440.2742	220.6407	441.2820	221.1446	4
14	1562.9377	781.9725	1545.9111	773.4592	1544.9271	772.9672	1579.9642	790.4858	A			359.2401	180.1237	342.2136	171.6104			***343.2214***	172.1143	**344.2292**	172.6183	3
15	1676.0218	838.5145	1658.9952	830.0012	1658.0112	829.5092	1693.0483	847.0278	I	243.1452	257.1608	288.2030	144.6051	271.1765	136.0919			272.1843	136.5958	**273.1921**	137.0997	2
16									R	73.0284		175.1190	88.0631	158.0924	79.5498			159.1002	80.0538	160.1081	80.5577	1

Monoisotopic mass of neutral peptide Mr(calc): 1849.1261
Fixed modifications: Carbamidomethyl (C) (apply to specified residues or termini only)
Variable modifications:
K1 : Dimethyl_KR (KR)
Ions Score: 83 **Expect:** 3e-06
Matches : 33/269 fragment ions using 38 most intense peaks (help)

Mascot Search Results: Peptide View

Kme2VLRDNIQGITKPAIR

MS/MS Fragmentation of KVLRDNIQGITKPAIR
Found in sp|P62806|H4_MOUSE in fgcz_10090, Histone H4 OS=Mus musculus GN=Hist1h4a

Match to Query 2075: 1849.126416 from(463.288880,4+) intensity(309250.4400) scans(4176) rtinseconds(2380.369) index(2556)
Title: 2557: Scan 4176 (rt=39.6728) [s:\p749\Proteomics\ORBI_2\andreamizzi_20110517_sperm_trypsin_GluC_ETD_CID\20110519_04_trypsin_swimup_NaCl_2.RAW]
Data file \\fgcz-ms.uzh.ch\p749\Proteomics\ORBI_2\andreamizzi_20110517_sperm_trypsin_GluC_ETD_CID\mgf__low_res_MS2_nomerge\20110519_04_trypsin_swimup_NaCl_2.mgf

Click mouse within plot area to zoom in by factor of two about that point
Or, Plot from 150 to 800 Da Full range
Label all possible matches ○ Label matches used for scoring ◉
Show Y-axis ☐

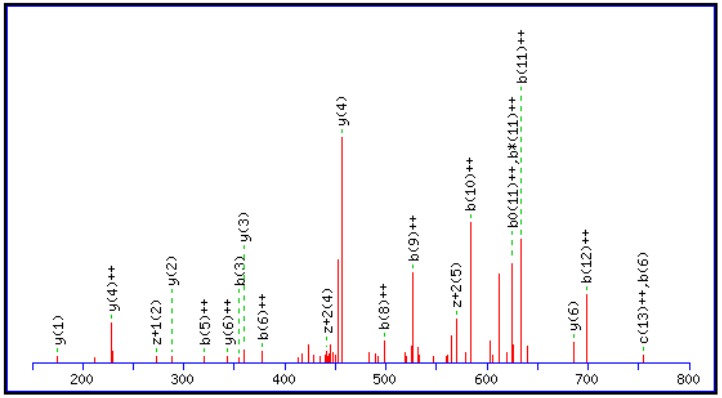

#	b	b^{++}	b^{*}	b^{*++}	b^{0}	b^{0++}	c	c^{++}	Seq.	w	w'	y	y^{++}	y^{*}	y^{*++}	y^{0}	y^{0++}	z+1	$z+1^{++}$	z+2	$z+2^{++}$	#
1	143.1179	72.0626	126.0913	63.5493			160.1444	80.5759	K													16
2	242.1863	121.5968	225.1598	113.0835			259.2129	130.1101	V	1676.9806		1708.0228	854.5150	1690.9963	846.0018	1690.0122	845.5098	1692.0041	846.5057	1693.0119	847.0096	15
3	**355.2704**	178.1388	338.2438	169.6255			372.2969	186.6521	L	1549.8809		1608.9544	804.9808	1591.9279	796.4676	1590.9438	795.9756	1592.9357	796.9715	1593.9435	797.4754	14
4	525.3871	263.1972	508.3606	254.6839			542.4137	271.7105	R	1379.7641		1495.8703	748.4388	1478.8438	739.9255	1477.8598	739.4335	1479.8516	740.4294	1480.8594	740.9334	13
5	640.4141	***320.7107***	623.3875	312.1974	622.4035	311.7054	657.4406	329.2239	D	1264.7372		1325.7536	663.3804	1308.7270	654.8672	1307.7430	654.3751	1309.7349	655.3711	1310.7427	655.8750	12
6	**754.4570**	***377.7321***	737.4304	369.2189	736.4464	368.7269	771.4835	386.2454	N	1150.6943		1210.7266	605.8670	1193.7001	597.3537	1192.7161	596.8617	1194.7079	597.8576	1195.7157	598.3615	11
7	867.5411	434.2742	850.5145	425.7609	849.5305	425.2689	884.5676	442.7874	I	1051.6259	1065.6415	1096.6837	548.8455	1079.6572	540.3322	1078.6731	539.8402	1080.6650	540.8361	1081.6728	541.3400	10
8	995.5996	***498.3035***	978.5731	489.7902	977.5891	489.2982	1012.6262	506.8167	Q	909.5516		983.5996	492.3035	966.5731	483.7902	965.5891	483.2982	967.5809	484.2941	968.5887	484.7980	9
9	1052.6211	***526.8142***	1035.5946	518.3009	1034.6105	517.8089	1069.6477	535.3275	G			855.5411	428.2742	838.5145	419.7609	837.5305	419.2689	839.5223	420.2648	840.5302	420.7687	8
10	1165.7052	***583.3562***	1148.6786	574.8429	1147.6946	574.3509	1182.7317	591.8695	I	753.4618	767.4774	798.5196	399.7634	781.4931	391.2502	780.5090	390.7582	782.5009	391.7541	783.5087	392.2580	7
11	1266.7528	***633.8801***	1249.7263	625.3668	1248.7423	624.8748	1283.7794	642.3933	T	652.4141	654.3933	***685.4355***	343.2214	668.4090	334.7081	667.4250	334.2161	669.4168	335.2120	670.4246	335.7160	6
12	1394.8478	***697.9275***	1377.8213	689.4143	1376.8372	688.9223	1411.8744	706.4408	K	510.3035		584.3879	292.6976	567.3613	284.1843			568.3691	284.6882	**569.3770**	285.1921	5
13	1491.9006	746.4539	1474.8740	737.9407	1473.8900	737.4486	1508.9271	754.9672	P	413.2507		***456.2929***	228.6501	439.2663	220.1368			440.2742	220.6407	**441.2820**	221.1446	4
14	1562.9377	781.9725	1545.9111	773.4592	1544.9271	772.9672	1579.9642	790.4858	A			***359.2401***	180.1237	342.2136	171.6104			**343.2214**	172.1143	344.2292	172.6183	3
15	1676.0218	838.5145	1658.9952	830.0012	1658.0112	829.5092	1693.0483	847.0278	I	243.1452	257.1608	***288.2030***	144.6051	271.1765	136.0919			**272.1843**	136.5958	273.1921	137.0997	2
16									R	73.0284		***175.1190***	88.0631	158.0924	79.5498			159.1002	80.0538	160.1081	80.5577	1

Monoisotopic mass of neutral peptide Mr(calc): 1849.1261
Fixed modifications: Carbamidomethyl (C) (apply to specified residues or termini only)
Variable modifications:
K1 : Methyl_KR (KR)
R4 : Methyl_KR (KR)
Ions Score: 48 **Expect:** 0.014
Matches : 23/269 fragment ions using 32 most intense peaks (help)

Mascot Search Results: Peptide View

Kme1VLRme1DNIQGITKPAIR

MS/MS Fragmentation of KVLRDNIQGITKPAIR
Found in sp|P62806|H4_MOUSE in fgcz_10090, Histone H4 OS=Mus musculus GN=Hist1h4a

Match to Query 3835: 1849.126216 from(463.288830,4+) intensity(190233.0500) scans(3455) rtinseconds(2388.7616) index(1611)
Title: 1467: Scan 3455 (rt=39.8127) [s:\p749\Proteomics\ORBI_2\andreamizzi_20110310_sperm_trypsin_chymo_AspN_ETD_CID\20110311_37_NaCl_trypsin_SCX_3.RAW]
Data file Y:\p749\Proteomics\Mascot\mgf\50 20110426 sperm rerun trypsin 2nd ETD run NaCl SCX\mascot_daemon_merge.mgf

Click mouse within plot area to zoom in by factor of two about that point
Or, Plot from 150 to 800 Da Full range
Label all possible matches ○ Label matches used for scoring ◉
Show Y-axis ☐

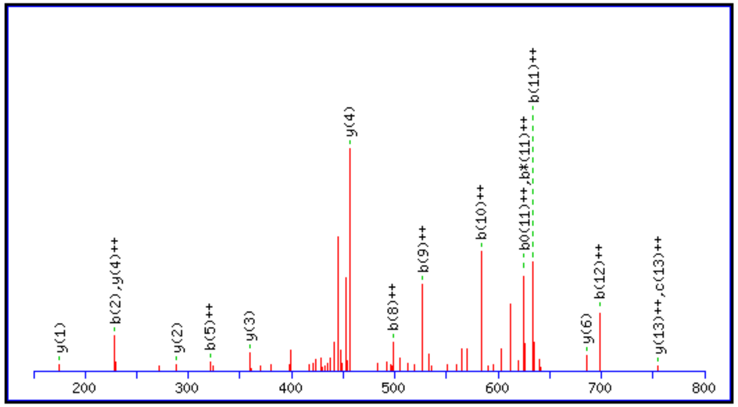

#	b	b^{++}	b^{*}	b^{*++}	b^{0}	b^{0++}	c	c^{++}	Seq.	w	w'	y	y^{++}	y^{*}	y^{*++}	y^{0}	y^{0++}	z+1	$z+1^{++}$	z+2	$z+2^{++}$	#
1	129.1022	65.0548	112.0757	56.5415			146.1288	73.5680	K													16
2	228.1707	114.5890	211.1441	106.0757			245.1972	123.1022	V	1690.9963		1722.0385	861.5229	1705.0119	853.0096	1704.0279	852.5176	1706.0197	853.5135	1707.0276	854.0174	15
3	341.2547	171.1310	324.2282	162.6177			358.2813	179.6443	L	1563.8966		1622.9701	811.9887	1605.9435	803.4754	1604.9595	802.9834	1606.9513	803.9793	1607.9592	804.4832	14
4	525.3871	263.1972	508.3606	254.6839			542.4137	271.7105	R	1379.7641		1509.8860	755.4466	1492.8594	746.9334	1491.8754	746.4413	1493.8673	747.4373	1494.8751	747.9412	13
5	640.4141	**320.7107**	623.3875	312.1974	622.4035	311.7054	657.4406	329.2239	D	1264.7372		1325.7536	663.3804	1308.7270	654.8672	1307.7430	654.3751	1309.7349	655.3711	1310.7427	655.8750	12
6	754.4570	377.7321	737.4304	369.2189	736.4464	368.7269	771.4835	386.2454	N	1150.6943		1210.7266	605.8670	1193.7001	597.3537	1192.7161	596.8617	1194.7079	597.8576	1195.7157	598.3615	11
7	867.5411	434.2742	850.5145	425.7609	849.5305	425.2689	884.5676	442.7874	I	1051.6259	1065.6415	1096.6837	548.8455	1079.6572	540.3322	1078.6731	539.8402	1080.6650	540.8361	1081.6728	541.3400	10
8	995.5996	**498.3035**	978.5731	489.7902	977.5891	489.2982	1012.6262	506.8167	Q	909.5516		983.5996	492.3035	966.5731	483.7902	965.5891	483.2982	967.5809	484.2941	968.5887	484.7980	9
9	1052.6211	**526.8142**	1035.5946	518.3009	1034.6105	517.8089	1069.6477	535.3275	G			855.5411	428.2742	838.5145	419.7609	837.5305	419.2689	839.5223	420.2648	840.5302	420.7687	8
10	1165.7052	**583.3562**	1148.6786	574.8429	1147.6946	574.3509	1182.7317	591.8695	I	753.4618	767.4774	798.5196	399.7634	781.4931	391.2502	780.5090	390.7582	782.5009	391.7541	783.5087	392.2580	7
11	1266.7528	**633.8801**	1249.7263	625.3668	1248.7423	624.8748	1283.7794	642.3933	T	652.4141	654.3933	**685.4355**	343.2214	668.4090	334.7081	667.4250	334.2161	669.4168	335.2120	670.4246	335.7160	6
12	1394.8478	**697.9275**	1377.8213	689.4143	1376.8372	688.9223	1411.8744	706.4408	K	510.3035		584.3879	292.6976	567.3613	284.1843			568.3691	284.6882	569.3770	285.1921	5
13	1491.9006	746.4539	1474.8740	737.9407	1473.8900	737.4486	1508.9271	754.9672	P	413.2507		**456.2929**	228.6501	439.2663	220.1368			440.2742	220.6407	441.2820	221.1446	4
14	1562.9377	781.9725	1545.9111	773.4592	1544.9271	772.9672	1579.9642	790.4858	A			**359.2401**	180.1237	342.2136	171.6104			343.2214	172.1143	344.2292	172.6183	3
15	1676.0218	838.5145	1658.9952	830.0012	1658.0112	829.5092	1693.0483	847.0278	I	243.1452	257.1608	**288.2030**	144.6051	271.1765	136.0919			272.1843	136.5958	273.1921	137.0997	2
16									R	73.0284		**175.1190**	88.0631	158.0924	79.5498			159.1002	80.0538	160.1081	80.5577	1

Monoisotopic mass of neutral peptide Mr(calc): 1849.1261
Fixed modifications: Carbamidomethyl (C) (apply to specified residues or termini only)
Variable modifications:
R4 : Dimethyl_KR (KR)
Ions Score: 49 **Expect:** 0.011
Matches : 17/269 fragment ions using 18 most intense peaks (help)

Mascot Search Results: Peptide View

KVLRme2DNIQGITKPAIR

MS/MS Fragmentation of KVLRDNIQGITKPAIR
Found in sp|P62806|H4_MOUSE in fgcz_10090, Histone H4 OS=Mus musculus GN=Hist1h4a

Match to Query 3894: 1863.141016 from(466.792530,4+) intensity(1253669.0000) scans(3322) rtinseconds(2361.4256) index(4541)
Title: 1515: Scan 3322 (rt=39.3571) [s:\p749\Proteomics\ORBI_2\andreamizzi_20110310_sperm_trypsin_chymo_AspN_ETD_CID\20110311_34_NaCl_trypsin_SCX_FT.RAW]
Data file Y:\p749\Proteomics\Mascot\mgf\50 20110426 sperm rerun trypsin 2nd ETD run NaCl SCX\mascot_daemon_merge.mgf

Click mouse within plot area to zoom in by factor of two about that point
Or, Plot from 150 to 1150 Da Full range
Label all possible matches ○ Label matches used for scoring ⦿
Show Y-axis ☐

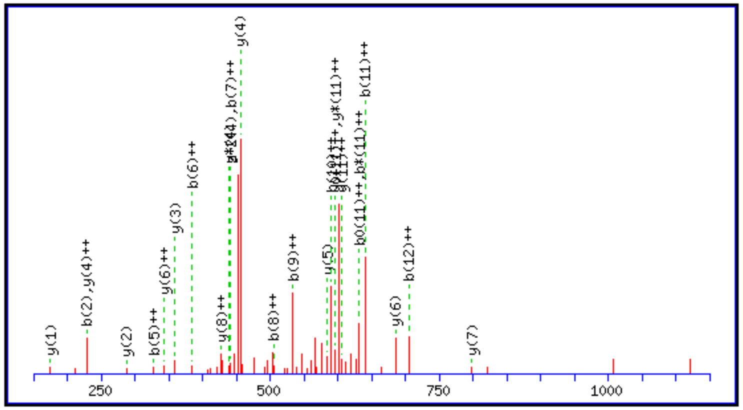

#	b	b^{++}	b^{*}	b^{*++}	b^{0}	b^{0++}	c	c^{++}	Seq.	w	w'	y	y^{++}	y^{*}	y^{*++}	y^{0}	y^{0++}	z+1	$z+1^{++}$	z+2	$z+2^{++}$	#
1	129.1022	65.0548	112.0757	56.5415			146.1288	73.5680	K													16
2	228.1707	114.5890	211.1441	106.0757			245.1972	123.1022	V	1705.0119		1736.0541	868.5307	1719.0276	860.0174	1718.0435	859.5254	1720.0354	860.5213	1721.0432	861.0252	15
3	341.2547	171.1310	324.2282	162.6177			358.2813	179.6443	L	1577.9122		1636.9857	818.9965	1619.9592	810.4832	1618.9751	809.9912	1620.9670	810.9871	1621.9748	811.4910	14
4	539.4028	270.2050	522.3762	261.6918			556.4293	278.7183	R	1379.7641		1523.9016	762.4545	1506.8751	753.9412	1505.8911	753.4492	1507.8829	754.4451	1508.8907	754.9490	13
5	654.4297	**327.7185**	637.4032	319.2052	636.4192	318.7132	671.4563	336.2318	D	1264.7372		1325.7536	663.3804	1308.7270	654.8672	1307.7430	654.3751	1309.7349	655.3711	1310.7427	655.8750	12
6	768.4726	**384.7400**	751.4461	376.2267	750.4621	375.7347	785.4992	393.2532	N	1150.6943		1210.7266	605.8670	1193.7001	597.3537	1192.7161	596.8617	1194.7079	597.8576	1195.7157	598.3615	11
7	881.5567	**441.2820**	864.5302	432.7687	863.5461	432.2767	898.5833	449.7953	I	1051.6259	1065.6415	1096.6837	548.8455	1079.6572	540.3322	1078.6731	539.8402	1080.6650	540.8361	1081.6728	541.3400	10
8	1009.6153	**505.3113**	992.5887	496.7980	991.6047	496.3060	1026.6418	513.8246	Q	909.5516		983.5996	492.3035	966.5731	483.7902	965.5891	483.2982	967.5809	484.2941	968.5887	484.7980	9
9	1066.6368	**533.8220**	1049.6102	525.3087	1048.6262	524.8167	1083.6633	542.3353	G			855.5411	428.2742	838.5145	419.7609	837.5305	419.2689	839.5223	420.2648	840.5302	420.7687	8
10	1179.7208	**590.3640**	1162.6943	581.8508	1161.7103	581.3588	1196.7474	598.8773	I	753.4618	767.4774	**798.5196**	399.7634	781.4931	391.2502	780.5090	390.7582	782.5009	391.7541	783.5087	392.2580	7
11	1280.7685	**640.8879**	1263.7419	632.3746	1262.7579	631.8826	1297.7950	649.4012	T	652.4141	654.3933	**685.4355**	343.2214	668.4090	334.7081	667.4250	334.2161	669.4168	335.2120	670.4246	335.7160	6
12	1408.8635	**704.9354**	1391.8369	696.4221	1390.8529	695.9301	1425.8900	713.4486	K	510.3035		**584.3879**	292.6976	567.3613	284.1843			568.3691	284.6882	569.3770	285.1921	5
13	1505.9162	753.4618	1488.8897	744.9485	1487.9057	744.4565	1522.9428	761.9750	P	413.2507		**456.2929**	228.6501	439.2663	220.1368			440.2742	220.6407	441.2820	221.1446	4
14	1576.9533	788.9803	1559.9268	780.4670	1558.9428	779.9750	1593.9799	797.4936	A			**359.2401**	180.1237	342.2136	171.6104			343.2214	172.1143	344.2292	172.6183	3
15	1690.0374	845.5223	1673.0109	837.0091	1672.0268	836.5171	1707.0640	854.0356	I	243.1452	257.1608	**288.2030**	144.6051	271.1765	136.0919			272.1843	136.5958	273.1921	137.0997	2
16									R	73.0284		**175.1190**	88.0631	158.0924	79.5498			159.1002	80.0538	160.1081	80.5577	1

Monoisotopic mass of neutral peptide Mr(calc): 1863.1418
Fixed modifications: Carbamidomethyl (C) (apply to specified residues or termini only)
Variable modifications:
R4 : Trimethyl (R)
Ions Score: 48 **Expect:** 0.0049
Matches : 27/269 fragment ions using 42 most intense peaks (help)

Mascot Search Results: Peptide View

(K)VL(Rme3)DNIQGITKPAIR

MS/MS Fragmentation of KVLRDNIQGITKPAIRR
Found in sp|P62806|H4_MOUSE in fgcz_10090, Histone H4 OS=Mus musculus GN=Hist1h4a

Match to Query 2705: 1991.211420 from(399.249560,5+) intensity(223752.4100) scans(3629) rtinseconds(2158.2574) index(2061)
Title: 2062: Scan 3629 (rt=35.971) [s:\p749\Proteomics\ORBI_2\andreamizzi_20110310_sperm_trypsin_chymo_AspN_ETD_CID\20110310_05_rep_ON7.RAW]
Data file \\fgcz-ms.uzh.ch\p749\Proteomics\ORBI_2\andreamizzi_20110310_sperm_trypsin_chymo_AspN_ETD_CID\mgf__low_res_MS2_nomerge\20110310_05_rep_ON7.mgf

Click mouse within plot area to zoom in by factor of two about that point
Or, Plot from 100 to 1300 Da Full range
Label all possible matches ○ Label matches used for scoring ◉
Show Y-axis ☐

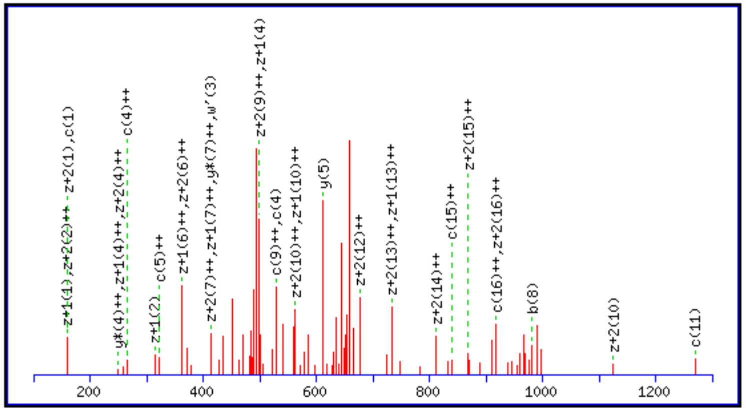

#	b	b^{++}	b^{*}	b^{*++}	b^{0}	b^{0++}	c	c^{++}	Seq.	w	w'	y	y^{++}	y^{*}	y^{*++}	y^{0}	y^{0++}	z+1	$z+1^{++}$	z+2	$z+2^{++}$	#
1	143.1179	72.0626	126.0913	63.5493			160.1444	80.5759	K													17
2	242.1863	121.5968	225.1598	113.0835			259.2129	130.1101	V	1819.0661		1850.1083	925.5578	1833.0817	917.0445	1832.0977	916.5525	1834.0896	917.5484	1835.0974	918.0523	16
3	355.2704	178.1388	338.2438	169.6255			372.2969	186.6521	L	1691.9664		1751.0399	876.0236	1734.0133	867.5103	1733.0293	867.0183	1735.0211	868.0142	1736.0290	868.5181	15
4	511.3715	256.1894	494.3449	247.6761			528.3980	264.7027	R	1535.8653		1637.9558	819.4815	1620.9292	810.9683	1619.9452	810.4763	1621.9371	811.4722	1622.9449	811.9761	14
5	626.3984	313.7028	609.3719	305.1896	608.3879	304.6976	643.4250	322.2161	D	1420.8383		1481.8547	741.4310	1464.8281	732.9177	1463.8441	732.4257	1465.8360	733.4216	1466.8438	733.9255	13
6	740.4413	370.7243	723.4148	362.2110	722.4308	361.7190	757.4679	379.2376	N	1306.7954		1366.8277	683.9175	1349.8012	675.4042	1348.8172	674.9122	1350.8090	675.9081	1351.8168	676.4121	12
7	853.5254	427.2663	836.4989	418.7531	835.5148	418.2611	870.5520	435.7796	I	1207.7270	1221.7426	1252.7848	626.8960	1235.7583	618.3828	1234.7743	617.8908	1236.7661	618.8867	1237.7739	619.3906	11
8	981.5840	491.2956	964.5574	482.7824	963.5734	482.2904	998.6105	499.8089	Q	1065.6527		1139.7008	570.3540	1122.6742	561.8407	1121.6902	561.3487	1123.6820	562.3447	1124.6899	562.8486	10
9	1038.6055	519.8064	1021.5789	511.2931	1020.5949	510.8011	1055.6320	528.3196	G			1011.6422	506.3247	994.6156	497.8115	993.6316	497.3194	995.6235	498.3154	996.6313	498.8193	9
10	1151.6895	576.3484	1134.6630	567.8351	1133.6790	567.3431	1168.7161	584.8617	I	909.5629	923.5785	954.6207	477.8140	937.5942	469.3007	936.6101	468.8087	938.6020	469.8046	939.6098	470.3085	8
11	1252.7372	626.8722	1235.7106	618.3590	1234.7266	617.8670	1269.7637	635.3855	T	808.5152	810.4944	841.5366	421.2720	824.5101	412.7587	823.5261	412.2667	825.5179	413.2626	826.5257	413.7665	7
12	1380.8322	690.9197	1363.8056	682.4064	1362.8216	681.9144	1397.8587	699.4330	K	666.4046		740.4890	370.7481	723.4624	362.2348			724.4702	362.7388	725.4781	363.2427	6
13	1477.8849	739.4461	1460.8584	730.9328	1459.8744	730.4408	1494.9115	747.9594	P	569.3518		612.3940	306.7006	595.3675	298.1874			596.3753	298.6913	597.3831	299.1952	5
14	1548.9220	774.9647	1531.8955	766.4514	1530.9115	765.9594	1565.9486	783.4779	A			515.3412	258.1743	498.3147	249.6610			499.3225	250.1649	500.3303	250.6688	4
15	1662.0061	831.5067	1644.9796	822.9934	1643.9955	822.5014	1679.0327	840.0200	I	399.2463	413.2619	444.3041	222.6557	427.2776	214.1424			428.2854	214.6463	429.2932	215.1503	3
16	1818.1072	909.5572	1801.0807	901.0440	1800.0966	900.5520	1835.1338	918.0705	R	229.1295		331.2201	166.1137	314.1935	157.6004			315.2013	158.1043	316.2092	158.6082	2
17									R	73.0284		175.1190	88.0631	158.0924	79.5498			159.1002	80.0538	160.1081	80.5577	1

Monoisotopic mass of neutral peptide Mr(calc): 1991.2116
Fixed modifications: Carbamidomethyl (C) (apply to specified residues or termini only)
Variable modifications:
K1 : Methyl_KR (KR)
Ions Score: 82 **Expect:** 8e-06
Matches : 34/286 fragment ions using 29 most intense peaks (help)

Mascot Search Results: Peptide View

Kme1VLRDNIQGITKPAIRR

MS/MS Fragmentation of KVLRDNIQGITKPAIRR
Found in sp|P62806|H4_MOUSE in fgcz_10090, Histone H4 OS=Mus musculus GN=Hist1h4a

Match to Query 4236: 2005.227536 from(502.314160,4+) intensity(262872.3100) scans(3091) rtinseconds(2250.3844) index(4371)
Title: 1345: Scan 3091 (rt=37.5064) [s:\p749\Proteomics\ORBI_2\andreamizzi_20110310_sperm_trypsin_chymo_AspN_ETD_CID\20110311_34_NaCl_trypsin_SCX_FT.RAW]
Data file Y:\p749\Proteomics\Mascot\mgf\50 20110426 sperm rerun trypsin 2nd ETD run NaCl SCX\mascot_daemon_merge.mgf

Click mouse within plot area to zoom in by factor of two about that point
Or, Plot from 200 to 2000 Da Full range
Label all possible matches ○ Label matches used for scoring ◉
Show Y-axis ☐

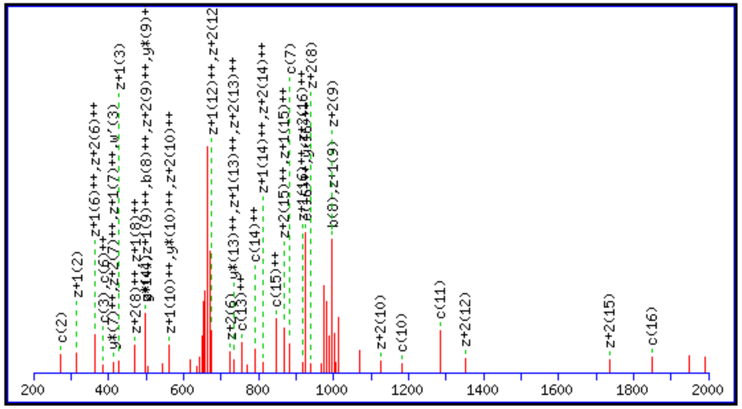

#	b	b^{++}	b^{*}	b^{*++}	b^{0}	b^{0++}	c	c^{++}	Seq.	w	w'	y	y^{++}	y^{*}	y^{*++}	y^{0}	y^{0++}	z+1	$z+1^{++}$	z+2	$z+2^{++}$	#
1	157.1335	79.0704	140.1070	70.5571			174.1601	87.5837	K													17
2	256.2020	128.6046	239.1754	120.0913			273.2285	137.1179	V	1819.0661		1850.1083	925.5578	1833.0817	917.0445	1832.0977	916.5525	1834.0896	917.5484	1835.0974	918.0523	16
3	369.2860	185.1466	352.2595	176.6334			386.3126	193.6599	L	1691.9664		1751.0399	876.0236	1734.0133	867.5103	1733.0293	867.0183	1735.0211	868.0142	1736.0290	868.5181	15
4	525.3871	263.1972	508.3606	254.6839			542.4137	271.7105	R	1535.8653		1637.9558	819.4815	1620.9292	810.9683	1619.9452	810.4763	1621.9371	811.4722	1622.9449	811.9761	14
5	640.4141	320.7107	623.3875	312.1974	622.4035	311.7054	657.4406	329.2239	D	1420.8383		1481.8547	741.4310	1464.8281	732.9177	1463.8441	732.4257	1465.8360	733.4216	1466.8438	733.9255	13
6	754.4570	377.7321	737.4304	369.2189	736.4464	368.7269	771.4835	386.2454	N	1306.7954		1366.8277	683.9175	1349.8012	675.4042	1348.8172	674.9122	1350.8090	675.9081	1351.8168	676.4121	12
7	867.5411	434.2742	850.5145	425.7609	849.5305	425.2689	884.5676	442.7874	I	1207.7270	1221.7426	1252.7848	626.8960	1235.7583	618.3828	1234.7743	617.8908	1236.7661	618.8867	1237.7739	619.3906	11
8	995.5996	498.3035	978.5731	489.7902	977.5891	489.2982	1012.6262	506.8167	Q	1065.6527		1139.7008	570.3540	1122.6742	561.8407	1121.6902	561.3487	1123.6820	562.3447	1124.6899	562.8486	10
9	1052.6211	526.8142	1035.5946	518.3009	1034.6105	517.8089	1069.6477	535.3275	G			1011.6422	506.3247	994.6156	497.8115	993.6316	497.3194	995.6235	498.3154	996.6313	498.8193	9
10	1165.7052	583.3562	1148.6786	574.8429	1147.6946	574.3509	1182.7317	591.8695	I	909.5629	923.5785	954.6207	477.8140	937.5942	469.3007	936.6101	468.8087	938.6020	469.8046	939.6098	470.3085	8
11	1266.7528	633.8801	1249.7263	625.3668	1248.7423	624.8748	1283.7794	642.3933	T	808.5152	810.4944	841.5366	421.2720	824.5101	412.7587	823.5261	412.2667	825.5179	413.2626	826.5257	413.7665	7
12	1394.8478	697.9275	1377.8213	689.4143	1376.8372	688.9223	1411.8744	706.4408	K	666.4046		740.4890	370.7481	723.4624	362.2348			724.4702	362.7388	725.4781	363.2427	6
13	1491.9006	746.4539	1474.8740	737.9407	1473.8900	737.4486	1508.9271	754.9672	P	569.3518		612.3940	306.7006	595.3675	298.1874			596.3753	298.6913	597.3831	299.1952	5
14	1562.9377	781.9725	1545.9111	773.4592	1544.9271	772.9672	1579.9642	790.4858	A			515.3412	258.1743	498.3147	249.6610			499.3225	250.1649	500.3303	250.6688	4
15	1676.0218	838.5145	1658.9952	830.0012	1658.0112	829.5092	1693.0483	847.0278	I	399.2463	413.2619	444.3041	222.6557	427.2776	214.1424			428.2854	214.6463	429.2932	215.1503	3
16	1832.1229	916.5651	1815.0963	908.0518	1814.1123	907.5598	1849.1494	925.0783	R	229.1295		331.2201	166.1137	314.1935	157.6004			315.2013	158.1043	316.2092	158.6082	2
17									R	73.0284		175.1190	88.0631	158.0924	79.5498			159.1002	80.0538	160.1081	80.5577	1

Monoisotopic mass of neutral peptide Mr(calc): 2005.2272
Fixed modifications: Carbamidomethyl (C) (apply to specified residues or termini only)
Variable modifications:
K1 : Dimethyl_KR (KR)
Ions Score: 84 **Expect:** 4.6e-06
Matches : 50/286 fragment ions using 39 most intense peaks (help)

Mascot Search Results: Peptide View

Kme2VLRDNIQGITKPAIRR

MS/MS Fragmentation of KVLRDNIQGITKPAIRR
Found in sp|P62806|H4_MOUSE in fgcz_10090, Histone H4 OS=Mus musculus GN=Hist1h4a

Match to Query 3391: 2019.244170 from(404.856110,5+) intensity(155560.1600) scans(4136) rtinseconds(2257.4328) index(2835)
Title: 2836: Scan 4136 (rt=37.6239) [s:\p749\Proteomics\ORBI_2\andreamizzi_20110517_sperm_trypsin_GluC_ETD_CID\20110517_02_NaCl_2.RAW]
Data file 20110517_02_NaCl_2.mgf

Click mouse within plot area to zoom in by factor of two about that point
Or, Plot from 100 to 1500 Da Full range
Label all possible matches ○ Label matches used for scoring ◉
Show Y-axis ☐

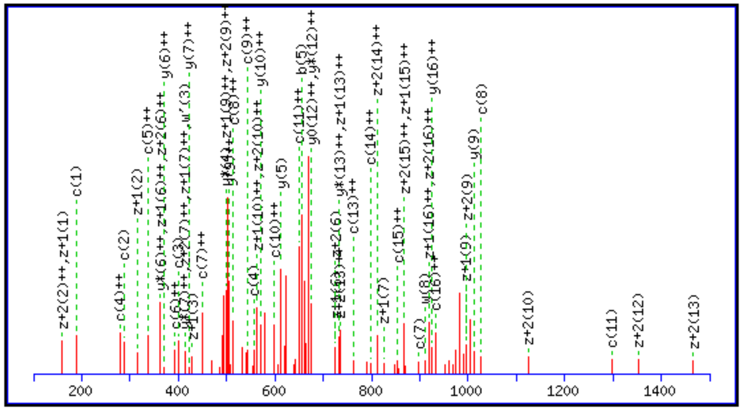

#	b	b^{++}	b^{*}	b^{*++}	b^{0}	b^{0++}	c	c^{++}	Seq.	w	w'	y	y^{++}	y^{*}	y^{*++}	y^{0}	y^{0++}	z+1	$z+1^{++}$	z+2	$z+2^{++}$	#
1	171.1492	86.0782	154.1226	77.5650			188.1757	94.5915	K													17
2	270.2176	135.6124	253.1911	127.0992			287.2442	144.1257	V	1819.0661		1850.1083	925.5578	1833.0817	917.0445	1832.0977	916.5525	1834.0896	917.5484	1835.0974	918.0523	16
3	383.3017	192.1545	366.2751	183.6412			400.3282	200.6677	L	1691.9664		1751.0399	876.0236	1734.0133	867.5103	1733.0293	867.0183	1735.0211	868.0142	1736.0290	868.5181	15
4	539.4028	270.2050	522.3762	261.6918			556.4293	278.7183	R	1535.8653		1637.9558	819.4815	1620.9292	810.9683	1619.9452	810.4763	1621.9371	811.4722	1622.9449	811.9761	14
5	654.4297	327.7185	637.4032	319.2052	636.4192	318.7132	671.4563	336.2318	D	1420.8383		1481.8547	741.4310	1464.8281	732.9177	1463.8441	732.4257	1465.8360	733.4216	1466.8438	733.9255	13
6	768.4726	384.7400	751.4461	376.2267	750.4621	375.7347	785.4992	393.2532	N	1306.7954		1366.8277	683.9175	1349.8012	675.4042	1348.8172	674.9122	1350.8090	675.9081	1351.8168	676.4121	12
7	881.5567	441.2820	864.5302	432.7687	863.5461	432.2767	898.5833	449.7953	I	1207.7270	1221.7426	1252.7848	626.8960	1235.7583	618.3828	1234.7743	617.8908	1236.7661	618.8867	1237.7739	619.3906	11
8	1009.6153	505.3113	992.5887	496.7980	991.6047	496.3060	1026.6418	513.8246	Q	1065.6527		1139.7008	570.3540	1122.6742	561.8407	1121.6902	561.3487	1123.6820	562.3447	1124.6899	562.8486	10
9	1066.6368	533.8220	1049.6102	525.3087	1048.6262	524.8167	1083.6633	542.3353	G			1011.6422	506.3247	994.6156	497.8115	993.6316	497.3194	995.6235	498.3154	996.6313	498.8193	9
10	1179.7208	590.3640	1162.6943	581.8508	1161.7103	581.3588	1196.7474	598.8773	I	909.5629	923.5785	954.6207	477.8140	937.5942	469.3007	936.6101	468.8087	938.6020	469.8046	939.6098	470.3085	8
11	1280.7685	640.8879	1263.7419	632.3746	1262.7579	631.8826	1297.7950	649.4012	T	808.5152	810.4944	841.5366	421.2720	824.5101	412.7587	823.5261	412.2667	825.5179	413.2626	826.5257	413.7665	7
12	1408.8635	704.9354	1391.8369	696.4221	1390.8529	695.9301	1425.8900	713.4486	K	666.4046		740.4890	370.7481	723.4624	362.2348			724.4702	362.7388	725.4781	363.2427	6
13	1505.9162	753.4618	1488.8897	744.9485	1487.9057	744.4565	1522.9428	761.9750	P	569.3518		612.3940	306.7006	595.3675	298.1874			596.3753	298.6913	597.3831	299.1952	5
14	1576.9533	788.9803	1559.9268	780.4670	1558.9428	779.9750	1593.9799	797.4936	A			515.3412	258.1743	498.3147	249.6610			499.3225	250.1649	500.3303	250.6688	4
15	1690.0374	845.5223	1673.0109	837.0091	1672.0268	836.5171	1707.0640	854.0356	I	399.2463	413.2619	444.3041	222.6557	427.2776	214.1424			428.2854	214.6463	429.2932	215.1503	3
16	1846.1385	923.5729	1829.1120	915.0596	1828.1279	914.5676	1863.1651	932.0862	R	229.1295		331.2201	166.1137	314.1935	157.6004			315.2013	158.1043	316.2092	158.6082	2
17									R	73.0284		175.1190	88.0631	158.0924	79.5498			159.1002	80.0538	160.1081	80.5577	1

Monoisotopic mass of neutral peptide Mr(calc): 2019.2429
Fixed modifications: Carbamidomethyl (C) (apply to specified residues or termini only)
Variable modifications:
K1 : Trimethyl (K)
Ions Score: 70 **Expect:** 6.7e-05
Matches : 62/286 fragment ions using 77 most intense peaks (help)

Mascot Search Results: Peptide View

Kme3VLRDNIQGITKPAIRR

MS/MS Fragmentation of **KVLRDNIQGITKPAIRR**
Found in **sp|P62806|H4_MOUSE** in **fgcz_10090**, Histone H4 OS=Mus musculus GN=Hist1h4a

Match to Query 2758: 2019.241670 from(404.855610,5+) intensity(689196.2500) scans(3813) rtinseconds(2319.3631) index(2457)
Title: 2458: Scan 3813 (rt=38.6561) [s:\p749\Proteomics\ORBI_2\andreamizzi_20110517_sperm_trypsin_GluC_ETD_CID\20110519_01_NaCl_ziptipped_1.RAW]
Data file 20110519_01_NaCl_ziptipped_1.mgf

Click mouse within plot area to zoom in by factor of two about that point
Or, Plot from 100 to 700 Da Full range
Label all possible matches ○ Label matches used for scoring ◉
Show Y-axis ☐

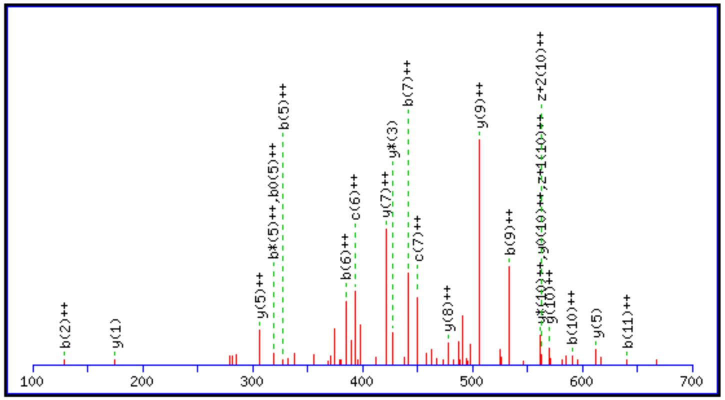

#	b	b++	b*	b*++	b0	b0++	c	c++	Seq.	w	w'	y	y++	y*	y*++	y0	y0++	z+1	z+1++	z+2	z+2++	#
1	157.1335	79.0704	140.1070	70.5571			174.1601	87.5837	K													17
2	256.2020	**128.6046**	239.1754	120.0913			273.2285	137.1179	V	1833.0817		1864.1239	932.5656	1847.0974	924.0523	1846.1134	923.5603	1848.1052	924.5562	1849.1130	925.0602	16
3	369.2860	185.1466	352.2595	176.6334			386.3126	193.6599	L	1705.9820		1765.0555	883.0314	1748.0290	874.5181	1747.0449	874.0261	1749.0368	875.0220	1750.0446	875.5259	15
4	539.4028	270.2050	522.3762	261.6918			556.4293	278.7183	R	1535.8653		1651.9714	826.4894	1634.9449	817.9761	1633.9609	817.4841	1635.9527	818.4800	1636.9605	818.9839	14
5	654.4297	**327.7185**	637.4032	319.2052	636.4192	318.7132	671.4563	336.2318	D	1420.8383		1481.8547	741.4310	1464.8281	732.9177	1463.8441	732.4257	1465.8360	733.4216	1466.8438	733.9255	13
6	768.4726	**384.7400**	751.4461	376.2267	750.4621	375.7347	785.4992	**393.2532**	N	1306.7954		1366.8277	683.9175	1349.8012	675.4042	1348.8172	674.9122	1350.8090	675.9081	1351.8168	676.4121	12
7	881.5567	**441.2820**	864.5302	432.7687	863.5461	432.2767	898.5833	**449.7953**	I	1207.7270	1221.7426	1252.7848	626.8960	1235.7583	618.3828	1234.7743	617.8908	1236.7661	618.8867	1237.7739	619.3906	11
8	1009.6153	505.3113	992.5887	496.7980	991.6047	496.3060	1026.6418	513.8246	Q	1065.6527		1139.7008	570.3540	1122.6742	561.8407	1121.6902	561.3487	1123.6820	562.3447	1124.6899	562.8486	10
9	1066.6368	**533.8220**	1049.6102	525.3087	1048.6262	524.8167	1083.6633	542.3353	G			1011.6422	506.3247	994.6156	497.8115	993.6316	497.3194	995.6235	498.3154	996.6313	498.8193	9
10	1179.7208	**590.3640**	1162.6943	581.8508	1161.7103	581.3588	1196.7474	598.8773	I	909.5629	923.5785	954.6207	477.8140	937.5942	469.3007	936.6101	468.8087	938.6020	469.8046	939.6098	470.3085	8
11	1280.7685	**640.8879**	1263.7419	632.3746	1262.7579	631.8826	1297.7950	649.4012	T	808.5152	810.4944	841.5366	421.2720	824.5101	412.7587	823.5261	412.2667	825.5179	413.2626	826.5257	413.7665	7
12	1408.8635	704.9354	1391.8369	696.4221	1390.8529	695.9301	1425.8900	713.4486	K	666.4046		740.4890	370.7481	723.4624	362.2348			724.4702	362.7388	725.4781	363.2427	6
13	1505.9162	753.4618	1488.8897	744.9485	1487.9057	744.4565	1522.9428	761.9750	P	569.3518		**612.3940**	306.7006	595.3675	298.1874			596.3753	298.6913	597.3831	299.1952	5
14	1576.9533	788.9803	1559.9268	780.4670	1558.9428	779.9750	1593.9799	797.4936	A			515.3412	258.1743	498.3147	249.6610			499.3225	250.1649	500.3303	250.6688	4
15	1690.0374	845.5223	1673.0109	837.0091	1672.0268	836.5171	1707.0640	854.0356	I	399.2463	413.2619	444.3041	222.6557	427.2776	214.1424			428.2854	214.6463	429.2932	215.1503	3
16	1846.1385	923.5729	1829.1120	915.0596	1828.1279	914.5676	1863.1651	932.0862	R	229.1295		331.2201	166.1137	314.1935	157.6004			315.2013	158.1043	316.2092	158.6082	2
17									R	73.0284		**175.1190**	88.0631	158.0924	79.5498			159.1002	80.0538	160.1081	80.5577	1

Monoisotopic mass of neutral peptide Mr(calc): 2019.2429
Fixed modifications: Carbamidomethyl (C) (apply to specified residues or termini only)
Variable modifications:
K1 : Dimethyl_KR (KR)
R4 : Methyl_KR (KR)
Ions Score: 44 **Expect:** 0.041
Matches : 23/286 fragment ions using 28 most intense peaks (help)

Mascot Search Results: Peptide View

Kme2VLRme1DNIQGITKPAIRR

MS/MS Fragmentation of KVLRDNIQGITKPAIRR
Found in sp|P62806|H4_MOUSE in fgcz_10090, Histone H4 OS=Mus musculus GN=Hist1h4a

Match to Query 2326: 2019.242820 from(404.855840,5+) intensity(838191.9400) scans(3135) rtinseconds(2144.1266) index(1666)
Title: 1667: Scan 3135 (rt=35.7354) [s:\p749\Proteomics\ORBI_2\andreamizzi_20110310_sperm_trypsin_chymo_AspN_ETD_CID\20110310_04_rep_2h7.RAW]
Data file \\fgcz-ms.uzh.ch\p749\Proteomics\ORBI_2\andreamizzi_20110310_sperm_trypsin_chymo_AspN_ETD_CID\mgf__low_res_MS2_nomerge\20110310_04_rep_2h7.mgf

Click mouse within plot area to zoom in by factor of two about that point
Or, Plot from 150 to 750 Da Full range
Label all possible matches ○ Label matches used for scoring ◉
Show Y-axis ☐

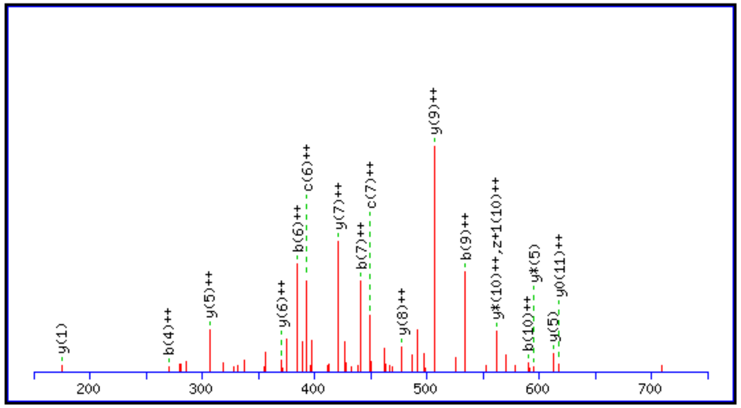

#	b	b^{++}	b^{*}	b^{*++}	b^{0}	b^{0++}	c	c^{++}	Seq.	w	w'	y	y^{++}	y^{*}	y^{*++}	y^{0}	y^{0++}	z+1	$z+1^{++}$	z+2	$z+2^{++}$	#
1	129.1022	65.0548	112.0757	56.5415			146.1288	73.5680	K													17
2	228.1707	114.5890	211.1441	106.0757			245.1972	123.1022	V	1861.1130		1892.1552	946.5813	1875.1287	938.0680	1874.1447	937.5760	1876.1365	938.5719	1877.1443	939.0758	16
3	341.2547	171.1310	324.2282	162.6177			358.2813	179.6443	L	1734.0133		1793.0868	897.0470	1776.0603	888.5338	1775.0762	888.0418	1777.0681	889.0377	1778.0759	889.5416	15
4	539.4028	**270.2050**	522.3762	261.6918			556.4293	278.7183	R	1535.8653		1680.0027	840.5050	1662.9762	831.9917	1661.9922	831.4997	1663.9840	832.4956	1664.9918	832.9996	14
5	654.4297	327.7185	637.4032	319.2052	636.4192	318.7132	671.4563	336.2318	D	1420.8383		1481.8547	741.4310	1464.8281	732.9177	1463.8441	732.4257	1465.8360	733.4216	1466.8438	733.9255	13
6	768.4726	**384.7400**	751.4461	376.2267	750.4621	375.7347	785.4992	**393.2532**	N	1306.7954		1366.8277	683.9175	1349.8012	675.4042	1348.8172	674.9122	1350.8090	675.9081	1351.8168	676.4121	12
7	881.5567	**441.2820**	864.5302	432.7687	863.5461	432.2767	898.5833	**449.7953**	I	1207.7270	1221.7426	1252.7848	626.8960	1235.7583	618.3828	1234.7743	617.8908	1236.7661	618.8867	1237.7739	619.3906	11
8	1009.6153	505.3113	992.5887	496.7980	991.6047	496.3060	1026.6418	513.8246	Q	1065.6527		1139.7008	570.3540	1122.6742	561.8407	1121.6902	561.3487	1123.6820	562.3447	1124.6899	562.8486	10
9	1066.6368	**533.8220**	1049.6102	525.3087	1048.6262	524.8167	1083.6633	542.3353	G			1011.6422	506.3247	994.6156	497.8115	993.6316	497.3194	995.6235	498.3154	996.6313	498.8193	9
10	1179.7208	**590.3640**	1162.6943	581.8508	1161.7103	581.3588	1196.7474	598.8773	I	909.5629	923.5785	954.6207	477.8140	937.5942	469.3007	936.6101	468.8087	938.6020	469.8046	939.6098	470.3085	8
11	1280.7685	640.8879	1263.7419	632.3746	1262.7579	631.8826	1297.7950	649.4012	T	808.5152	810.4944	841.5366	421.2720	824.5101	412.7587	823.5261	412.2667	825.5179	413.2626	826.5257	413.7665	7
12	1408.8635	704.9354	1391.8369	696.4221	1390.8529	695.9301	1425.8900	713.4486	K	666.4046		740.4890	370.7481	723.4624	362.2348			724.4702	362.7388	725.4781	363.2427	6
13	1505.9162	753.4618	1488.8897	744.9485	1487.9057	744.4565	1522.9428	761.9750	P	569.3518		**612.3940**	306.7006	595.3675	298.1874			596.3753	298.6913	597.3831	299.1952	5
14	1576.9533	788.9803	1559.9268	780.4670	1558.9428	779.9750	1593.9799	797.4936	A			515.3412	258.1743	498.3147	249.6610			499.3225	250.1649	500.3303	250.6688	4
15	1690.0374	845.5223	1673.0109	837.0091	1672.0268	836.5171	1707.0640	854.0356	I	399.2463	413.2619	444.3041	222.6557	427.2776	214.1424			428.2854	214.6463	429.2932	215.1503	3
16	1846.1385	923.5729	1829.1120	915.0596	1828.1279	914.5676	1863.1651	932.0862	R	229.1295		331.2201	166.1137	314.1935	157.6004			315.2013	158.1043	316.2092	158.6082	2
17									R	73.0284		**175.1190**	88.0631	158.0924	79.5498			159.1002	80.0538	160.1081	80.5577	1

Monoisotopic mass of neutral peptide Mr(calc): 2019.2429
Fixed modifications: Carbamidomethyl (C) (apply to specified residues or termini only)
Variable modifications:
R4 : Trimethyl (R)
Ions Score: 41 **Expect:** 0.013
Matches : 18/286 fragment ions using 23 most intense peaks (help)

Mascot Search Results: Peptide View

(K)VL(Rme3)DNIQGITKPAIRR

MS/MS Fragmentation of KVLRDNIQGITKPAIRRLAR
Found in sp|P62806|H4_MOUSE in fgcz_10090, Histone H4 OS=Mus musculus GN=Hist1h4a

Match to Query 5013: 2345.444484 from(391.914690,6+) intensity(16222.5950) scans(3598) rtinseconds(2484.1767) index(1674)
Title: 1530: Scan 3598 (rt=41.4029) [s:\p749\Proteomics\ORBI_2\andreamizzi_20110310_sperm_trypsin_chymo_AspN_ETD_CID\20110311_37_NaCl_trypsin_SCX_3.RAW]
Data file Y:\p749\Proteomics\Mascot\mgf\50 20110426 sperm rerun trypsin 2nd ETD run NaCl SCX\mascot_daemon_merge.mgf

Click mouse within plot area to zoom in by factor of two about that point
Or, Plot from 250 to 1200 Da Full range
Label all possible matches ○ Label matches used for scoring ◉
Show Y-axis ☐

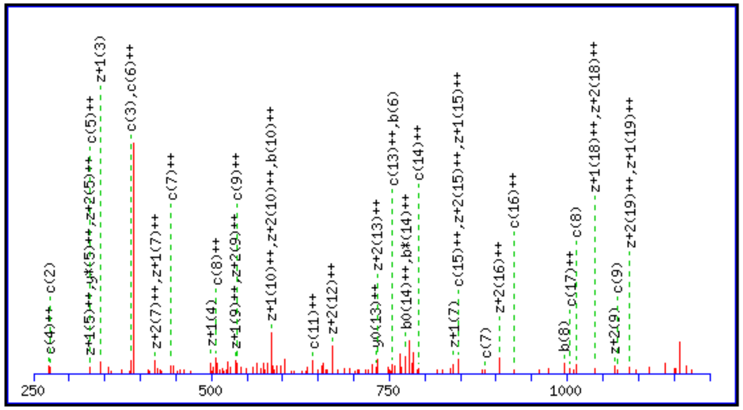

#	b	b^{++}	b^{*}	b^{*++}	b^{0}	b^{0++}	c	c^{++}	Seq.	w	w'	y	y^{++}	y^{*}	y^{*++}	y^{0}	y^{0++}	z+1	$z+1^{++}$	z+2	$z+2^{++}$	#
1	157.1335	79.0704	140.1070	70.5571			174.1601	87.5837	K													20
2	256.2020	128.6046	239.1754	120.0913			273.2285	137.1179	V	2159.2884		2190.3306	1095.6689	2173.3040	1087.1556	2172.3200	1086.6636	2174.3118	1087.6596	2175.3197	1088.1635	19
3	369.2860	185.1466	352.2595	176.6334			386.3126	193.6599	L	2032.1887		2091.2622	1046.1347	2074.2356	1037.6214	2073.2516	1037.1294	2075.2434	1038.1254	2076.2513	1038.6293	18
4	525.3871	263.1972	508.3606	254.6839			542.4137	271.7105	R	1876.0875		1978.1781	989.5927	1961.1515	981.0794	1960.1675	980.5874	1962.1594	981.5833	1963.1672	982.0872	17
5	640.4141	320.7107	623.3875	312.1974	622.4035	311.7054	657.4406	329.2239	D	1761.0606		1822.0770	911.5421	1805.0504	903.0289	1804.0664	902.5368	1806.0583	903.5328	1807.0661	904.0367	16
6	754.4570	377.7321	737.4304	369.2189	736.4464	368.7269	771.4835	386.2454	N	1647.0177		1707.0500	854.0287	1690.0235	845.5154	1689.0395	845.0234	1691.0313	846.0193	1692.0391	846.5232	15
7	867.5411	434.2742	850.5145	425.7609	849.5305	425.2689	884.5676	442.7874	I	1547.9493	1561.9649	1593.0071	797.0072	1575.9806	788.4939	1574.9965	788.0019	1576.9884	788.9978	1577.9962	789.5017	14
8	995.5996	498.3035	978.5731	489.7902	977.5891	489.2982	1012.6262	506.8167	Q	1405.8750		1479.9230	740.4652	1462.8965	731.9519	1461.9125	731.4599	1463.9043	732.4558	1464.9121	732.9597	13
9	1052.6211	526.8142	1035.5946	518.3009	1034.6105	517.8089	1069.6477	535.3275	G			1351.8645	676.4359	1334.8379	667.9226	1333.8539	667.4306	1335.8457	668.4265	1336.8536	668.9304	12
10	1165.7052	583.3562	1148.6786	574.8429	1147.6946	574.3509	1182.7317	591.8695	I	1249.7852	1263.8008	1294.8430	647.9251	1277.8165	639.4119	1276.8324	638.9199	1278.8243	639.9158	1279.8321	640.4197	11
11	1266.7528	633.8801	1249.7263	625.3668	1248.7423	624.8748	1283.7794	642.3933	T	1148.7375	1150.7167	1181.7589	591.3831	1164.7324	582.8698	1163.7484	582.3778	1165.7402	583.3737	1166.7480	583.8777	10
12	1394.8478	697.9275	1377.8213	689.4143	1376.8372	688.9223	1411.8744	706.4408	K	1006.6269		1080.7113	540.8593	1063.6847	532.3460			1064.6925	532.8499	1065.7004	533.3538	9
13	1491.9006	746.4539	1474.8740	737.9407	1473.8900	737.4486	1508.9271	754.9672	P	909.5741		952.6163	476.8118	935.5897	468.2985			936.5976	468.8024	937.6054	469.3063	8
14	1562.9377	781.9725	1545.9111	773.4592	1544.9271	772.9672	1579.9642	790.4858	A			855.5635	428.2854	838.5370	419.7721			839.5448	420.2760	840.5526	420.7800	7
15	1676.0218	838.5145	1658.9952	830.0012	1658.0112	829.5092	1693.0483	847.0278	I	739.4686	753.4842	784.5264	392.7668	767.4999	384.2536			768.5077	384.7575	769.5155	385.2614	6
16	1832.1229	916.5651	1815.0963	908.0518	1814.1123	907.5598	1849.1494	925.0783	R	569.3518		671.4424	336.2248	654.4158	327.7115			655.4236	328.2155	656.4315	328.7194	5
17	1988.2240	994.6156	1971.1974	986.1024	1970.2134	985.6103	2005.2505	1003.1289	R	413.2507		515.3412	258.1743	498.3147	249.6610			499.3225	250.1649	500.3303	250.6688	4
18	2101.3080	1051.1577	2084.2815	1042.6444	2083.2975	1042.1524	2118.3346	1059.6709	L	300.1666		359.2401	180.1237	342.2136	171.6104			343.2214	172.1143	344.2292	172.6183	3
19	2172.3452	1086.6762	2155.3186	1078.1629	2154.3346	1077.6709	2189.3717	1095.1895	A			246.1561	123.5817	229.1295	115.0684			230.1373	115.5723	231.1452	116.0762	2
20									R	73.0284		175.1190	88.0631	158.0924	79.5498			159.1002	80.0538	160.1081	80.5577	1

Monoisotopic mass of neutral peptide Mr(calc): 2345.4495
Fixed modifications: Carbamidomethyl (C) (apply to specified residues or termini only)
Variable modifications:
K1 : Dimethyl_KR (KR)
Ions Score: 54 **Expect:** 0.00064
Matches : 45/336 fragment ions using 62 most intense peaks (help)

Mascot Search Results: Peptide View

Kme2VLRDNIQGITKPAIRRLAR

MS/MS Fragmentation of KVLRDNIQGITKPAIRRLAR
Found in sp|P62806|H4_MOUSE in fgcz_10090, Histone H4 OS=Mus musculus GN=Hist1h4a

Match to Query 5065: 2359.465370 from(472.900350,5+) intensity(49007.9100) scans(3580) rtinseconds(2472.0198) index(1665)
Title: 1521: Scan 3580 (rt=41.2003) [s:\p749\Proteomics\ORBI_2\andreamizzi_20110310_sperm_trypsin_chymo_AspN_ETD_CID\20110311_37_NaCl_trypsin_SCX_3.RAW]
Data file Y:\p749\Proteomics\Mascot\mgf\50 20110426 sperm rerun trypsin 2nd ETD run NaCl SCX\mascot_daemon_merge.mgf

Click mouse within plot area to zoom in by factor of two about that point
Or, Plot from 100 to 1200 Da Full range
Label all possible matches ○ Label matches used for scoring ◉
Show Y-axis ☐

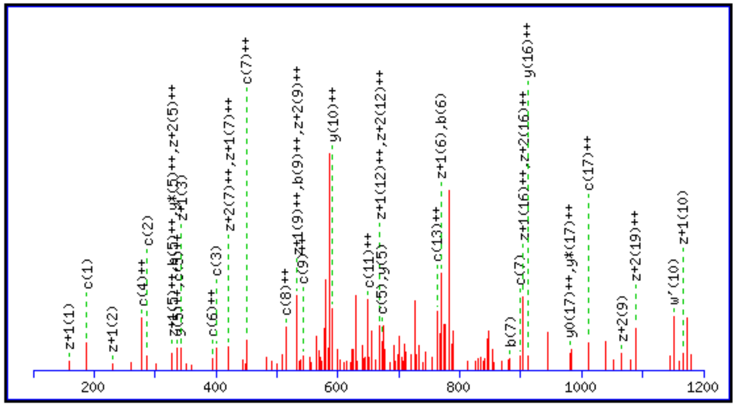

#	b	b++	b*	b*++	b0	b0++	c	c++	Seq.	w	w'	y	y++	y*	y*++	y0	y0++	z+1	z+1++	z+2	z+2++	#
1	171.1492	86.0782	154.1226	77.5650			188.1757	94.5915	K													20
2	270.2176	135.6124	253.1911	127.0992			287.2442	144.1257	V	2159.2884		2190.3306	1095.6689	2173.3040	1087.1556	2172.3200	1086.6636	2174.3118	1087.6596	2175.3197	1088.1635	19
3	383.3017	192.1545	366.2751	183.6412			400.3282	200.6677	L	2032.1887		2091.2622	1046.1347	2074.2356	1037.6214	2073.2516	1037.1294	2075.2434	1038.1254	2076.2513	1038.6293	18
4	539.4028	270.2050	522.3762	261.6918			556.4293	278.7183	R	1876.0875		1978.1781	989.5927	1961.1515	981.0794	1960.1675	980.5874	1962.1594	981.5833	1963.1672	982.0872	17
5	654.4297	327.7185	637.4032	319.2052	636.4192	318.7132	671.4563	336.2318	D	1761.0606		1822.0770	911.5421	1805.0504	903.0289	1804.0664	902.5368	1806.0583	903.5328	1807.0661	904.0367	16
6	768.4726	384.7400	751.4461	376.2267	750.4621	375.7347	785.4992	393.2532	N	1647.0177		1707.0500	854.0287	1690.0235	845.5154	1689.0395	845.0234	1691.0313	846.0193	1692.0391	846.5232	15
7	881.5567	441.2820	864.5302	432.7687	863.5461	432.2767	898.5833	449.7953	I	1547.9493	1561.9649	1593.0071	797.0072	1575.9806	788.4939	1574.9965	788.0019	1576.9884	788.9978	1577.9962	789.5017	14
8	1009.6153	505.3113	992.5887	496.7980	991.6047	496.3060	1026.6418	513.8246	Q	1405.8750		1479.9230	740.4652	1462.8965	731.9519	1461.9125	731.4599	1463.9043	732.4558	1464.9121	732.9597	13
9	1066.6368	533.8220	1049.6102	525.3087	1048.6262	524.8167	1083.6633	542.3353	G			1351.8645	676.4359	1334.8379	667.9226	1333.8539	667.4306	1335.8457	668.4265	1336.8536	668.9304	12
10	1179.7208	590.3640	1162.6943	581.8508	1161.7103	581.3588	1196.7474	598.8773	I	1249.7852	1263.8008	1294.8430	647.9251	1277.8165	639.4119	1276.8324	638.9199	1278.8243	639.9158	1279.8321	640.4197	11
11	1280.7685	640.8879	1263.7419	632.3746	1262.7579	631.8826	1297.7950	649.4012	T	1148.7375	1150.7167	1181.7589	591.3831	1164.7324	582.8698	1163.7484	582.3778	1165.7402	583.3737	1166.7480	583.8777	10
12	1408.8635	704.9354	1391.8369	696.4221	1390.8529	695.9301	1425.8900	713.4486	K	1006.6269		1080.7113	540.8593	1063.6847	532.3460			1064.6925	532.8499	1065.7004	533.3538	9
13	1505.9162	753.4618	1488.8897	744.9485	1487.9057	744.4565	1522.9428	761.9750	P	909.5741		952.6163	476.8118	935.5897	468.2985			936.5976	468.8024	937.6054	469.3063	8
14	1576.9533	788.9803	1559.9268	780.4670	1558.9428	779.9750	1593.9799	797.4936	A			855.5635	428.2854	838.5370	419.7721			839.5448	420.2760	840.5526	420.7800	7
15	1690.0374	845.5223	1673.0109	837.0091	1672.0268	836.5171	1707.0640	854.0356	I	739.4686	753.4842	784.5264	392.7668	767.4999	384.2536			768.5077	384.7575	769.5155	385.2614	6
16	1846.1385	923.5729	1829.1120	915.0596	1828.1279	914.5676	1863.1651	932.0862	R	569.3518		671.4424	336.2248	654.4158	327.7115			655.4236	328.2155	656.4315	328.7194	5
17	2002.2396	1001.6234	1985.2131	993.1102	1984.2291	992.6182	2019.2662	1010.1367	R	413.2507		515.3412	258.1743	498.3147	249.6610			499.3225	250.1649	500.3303	250.6688	4
18	2115.3237	1058.1655	2098.2971	1049.6522	2097.3131	1049.1602	2132.3502	1066.6788	L	300.1666		359.2401	180.1237	342.2136	171.6104			343.2214	172.1143	344.2292	172.6183	3
19	2186.3608	1093.6840	2169.3343	1085.1708	2168.3502	1084.6788	2203.3874	1102.1973	A			246.1561	123.5817	229.1295	115.0684			230.1373	115.5723	231.1452	116.0762	2
20									R	73.0284		175.1190	88.0631	158.0924	79.5498			159.1002	80.0538	160.1081	80.5577	1

Monoisotopic mass of neutral peptide Mr(calc): 2359.4651
Fixed modifications: Carbamidomethyl (C) (apply to specified residues or termini only)
Variable modifications:
K1 : Trimethyl (K)
Ions Score: 44 **Expect:** 0.024
Matches : 43/336 fragment ions using 52 most intense peaks (help)

Mascot Search Results: Peptide View

Kme3VLRDNIQGITKPAIRRLAR

MS/MS Fragmentation of **SGRGKGGKGLGK**
Found in **sp|P62806|H4_MOUSE** in **fgcz_10090**, Histone H4 OS=Mus musculus GN=Hist1h4a

Match to Query 618: 1142.654682 from(381.892170,3+) intensity(29678.1660) scans(1977) rtinseconds(1354.6946) index(1041)
Title: 1042: Scan 1977 (rt=22.5782) [s:\p749\Proteomics\ORBI_2\andreamizzi_20110517_sperm_trypsin_GluC_ETD_CID\20110517_06_swimup_NaCl_1.RAW]
Data file 20110517_06_swimup_NaCl_1.mgf

Click mouse within plot area to zoom in by factor of two about that point
Or, Plot from 300 to 1150 Da Full range
Label all possible matches ○ Label matches used for scoring ◉
Show Y-axis ☐

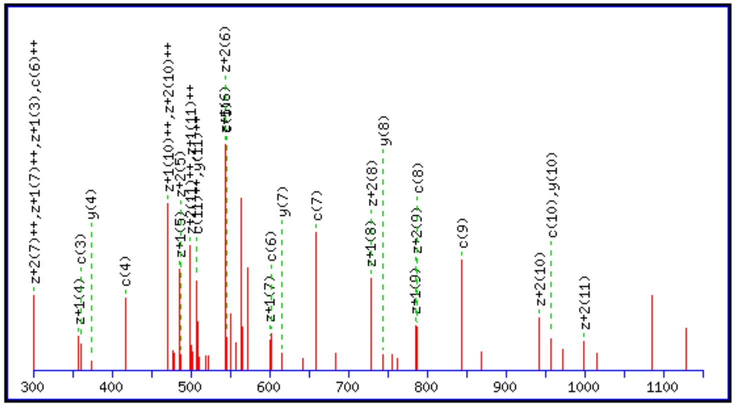

#	b	b^{++}	b^{*}	b^{*++}	b^{0}	b^{0++}	c	c^{++}	Seq.	w	y	y^{++}	y^{*}	y^{*++}	z+1	$z+1^{++}$	z+2	$z+2^{++}$	#
1	130.0499	65.5286			112.0393	56.5233	147.0764	74.0418	S										12
2	187.0713	94.0393			169.0608	85.0340	204.0979	102.5526	G		1014.6167	507.8120	997.5901	499.2987	998.5980	**499.8026**	***999.6058***	**500.3065**	11
3	343.1724	172.0899	326.1459	163.5766	325.1619	163.0846	***360.1990***	180.6031	R	855.5047	**957.5952**	479.3013	940.5687	470.7880	941.5765	**471.2919**	***942.5843***	**471.7958**	10
4	400.1939	200.6006	383.1674	192.0873	382.1833	191.5953	***417.2205***	209.1139	G		801.4941	401.2507	784.4676	392.7374	**785.4754**	393.2413	***786.4832***	393.7452	9
5	528.2889	264.6481	511.2623	256.1348	510.2783	255.6428	***545.3154***	273.1613	K	670.3883	**744.4727**	372.7400	727.4461	364.2267	**728.4539**	364.7306	***729.4618***	365.2345	8
6	585.3103	293.1588	568.2838	284.6455	567.2998	284.1535	***602.3369***	301.6721	G		**616.3777**	308.6925	599.3511	300.1792	**600.3590**	**300.6831**	601.3668	**301.1870**	7
7	642.3318	321.6695	625.3053	313.1563	624.3212	312.6643	***659.3583***	330.1828	G		559.3562	280.1817	542.3297	271.6685	**543.3375**	272.1724	***544.3453***	272.6763	6
8	770.4268	385.7170	753.4002	377.2037	752.4162	376.7117	***787.4533***	394.2303	K	428.2504	502.3348	251.6710	485.3082	243.1577	**486.3160**	243.6617	***487.3239***	244.1656	5
9	827.4482	414.2278	810.4217	405.7145	809.4377	405.2225	***844.4748***	422.7410	G		**374.2398**	187.6235	357.2132	179.1103	**358.2211**	179.6142	359.2289	180.1181	4
10	940.5323	470.7698	923.5057	462.2565	922.5217	461.7645	***957.5588***	479.2831	L	258.1448	317.2183	159.1128	300.1918	150.5995	**301.1996**	151.1034	302.2074	151.6074	3
11	997.5538	499.2805	980.5272	490.7672	979.5432	490.2752	1014.5803	507.7938	G		204.1343	102.5708	187.1077	94.0575	188.1155	94.5614	189.1234	95.0653	2
12									K	73.0284	147.1128	74.0600	130.0863	65.5468	131.0941	66.0507	132.1019	66.5546	1

Monoisotopic mass of neutral peptide Mr(calc): 1142.6520
Fixed modifications: Carbamidomethyl (C) (apply to specified residues or termini only)
Variable modifications:
S1 : Acetyl_STY (STY)
Ions Score: 58 **Expect:** 0.00093
Matches : 34/177 fragment ions using 45 most intense peaks (help)

Mascot Search Results: Peptide View

SacGRGKGGKGLGK
