# Supplementary material for: Epigenetic marking of sperm by post-translational modification of histones and protamines
Source: Epigenetics Chromatin. 2014 Jan 20;7:2. doi: 10.1186/1756-8935-7-2 (PMC3904194; doi:10.1186/1756-8935-7-2)

Supplementary Figure 1 ABC, Brunner et al.

A

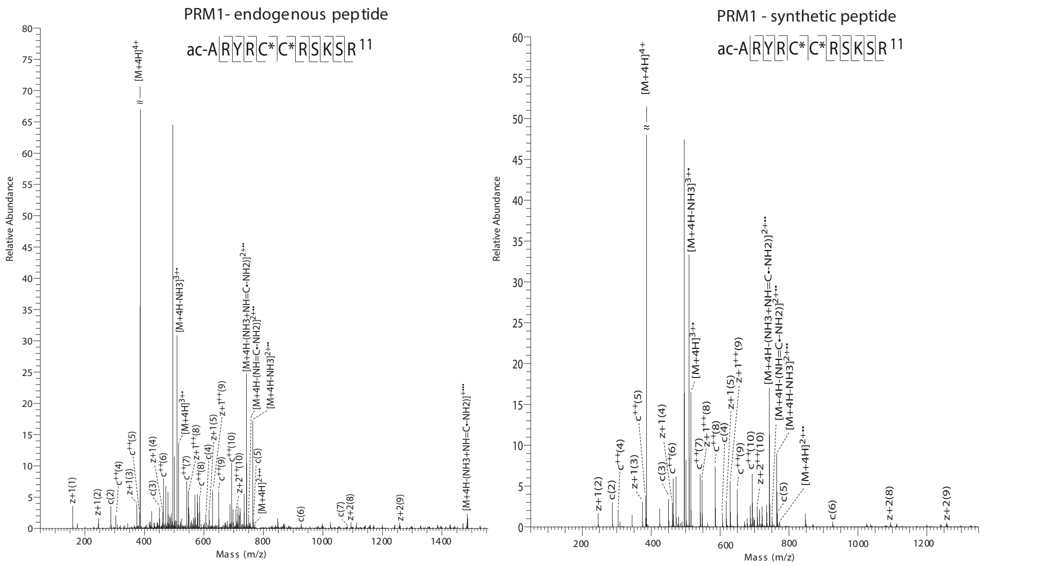

B

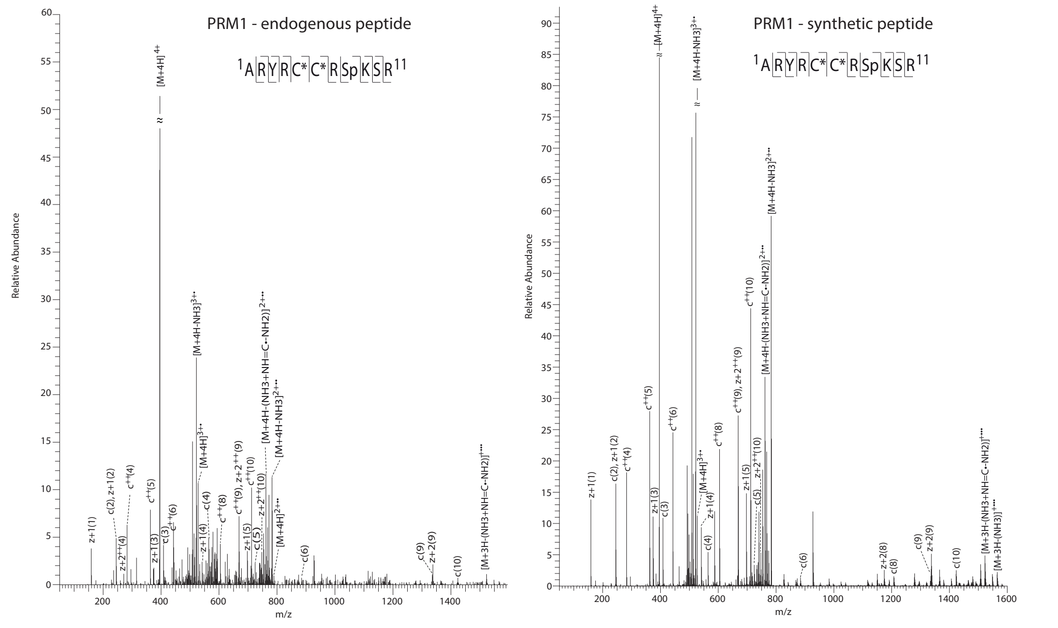

C

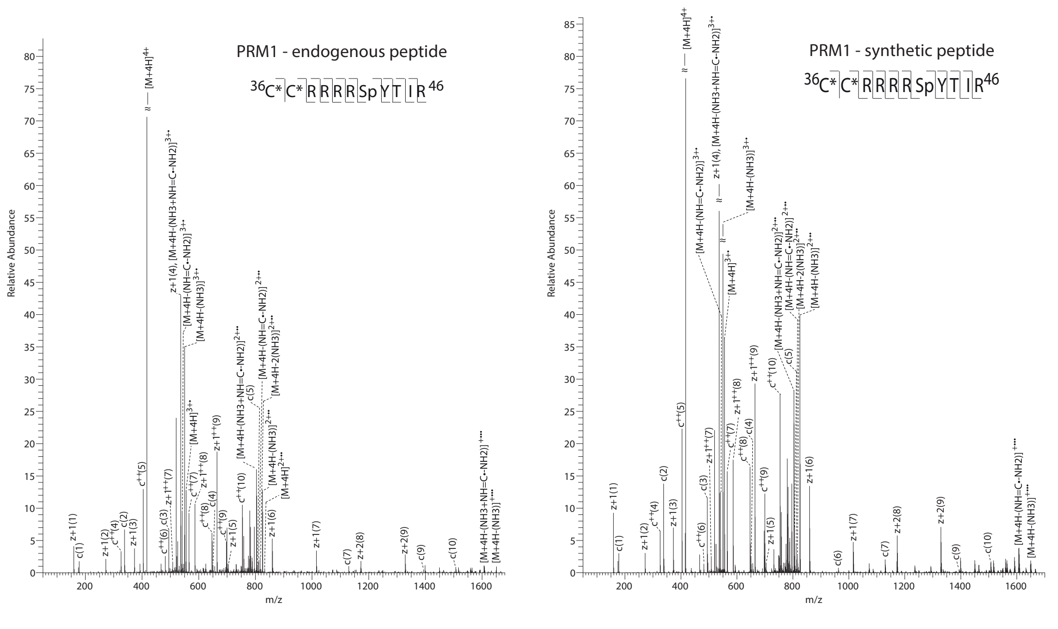

Supplementary Figure 1 DEF, Brunner et al.

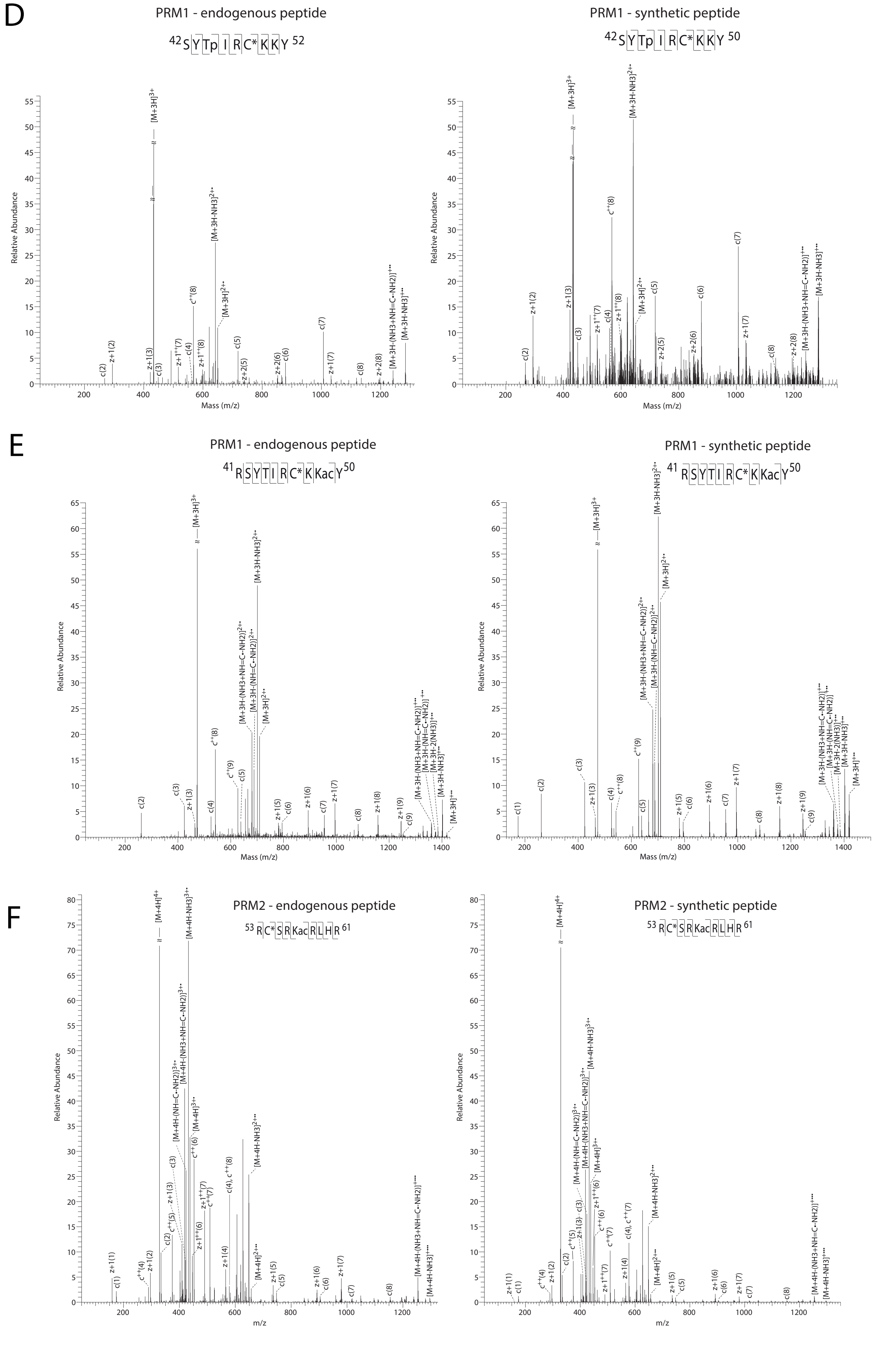

Supplementary Figure 1 G, Brunner et al.

G

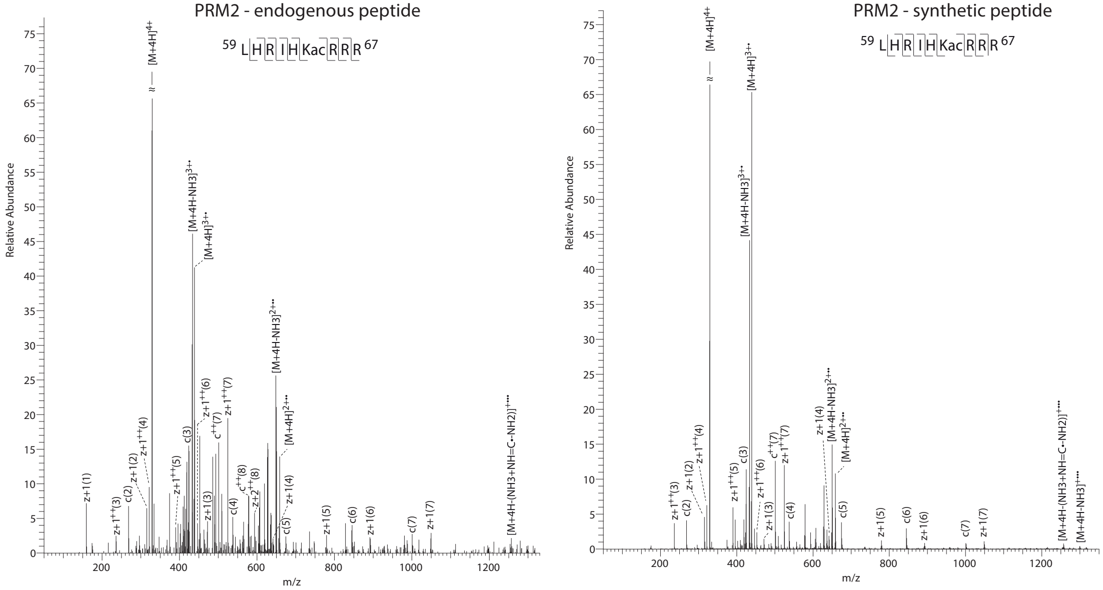

Supplement: Additional file 4: Figure S1 — Protamine post-translational modification (PTM) site validation by spectral comparison with synthetic peptides. Mass spectra of identified endogenous protamine peptides with novel PTM sites and their synthetic counterparts. Major peaks are labeled in the spectra and the fragment ions indicated in the peptide sequence. A) A novel site of acetylation at the N-terminus of PRM1. B) A novel site of serine phosphorylation on residue S8 of PRM1. C) A novel site of serine phosphorylation on residue S42 of PRM1. D) A novel site of threonine phosphorylation on residue T44 of PRM1. E) A novel site of lysine acetylation on residue K49 of PRM1. F) A novel site of lysine acetylation on residue K57 of PRM2. G) A novel site of lysine acetylation on residue K64 of PRM2. [file 1756-8935-7-2-S4.pdf]
