# Supplementary material for: Epigenetic marking of sperm by post-translational modification of histones and protamines
Source: Epigenetics Chromatin. 2014 Jan 20;7:2. doi: 10.1186/1756-8935-7-2 (PMC3904194; doi:10.1186/1756-8935-7-2)
Supplement: Additional file 5: Figure S3 — MS2 spectra of intact PRM1 and PRM2 forms. [file 1756-8935-7-2-S5.pdf]

Supplementary Figure 3, Brunner et al.

PRM2 (2 – 106)

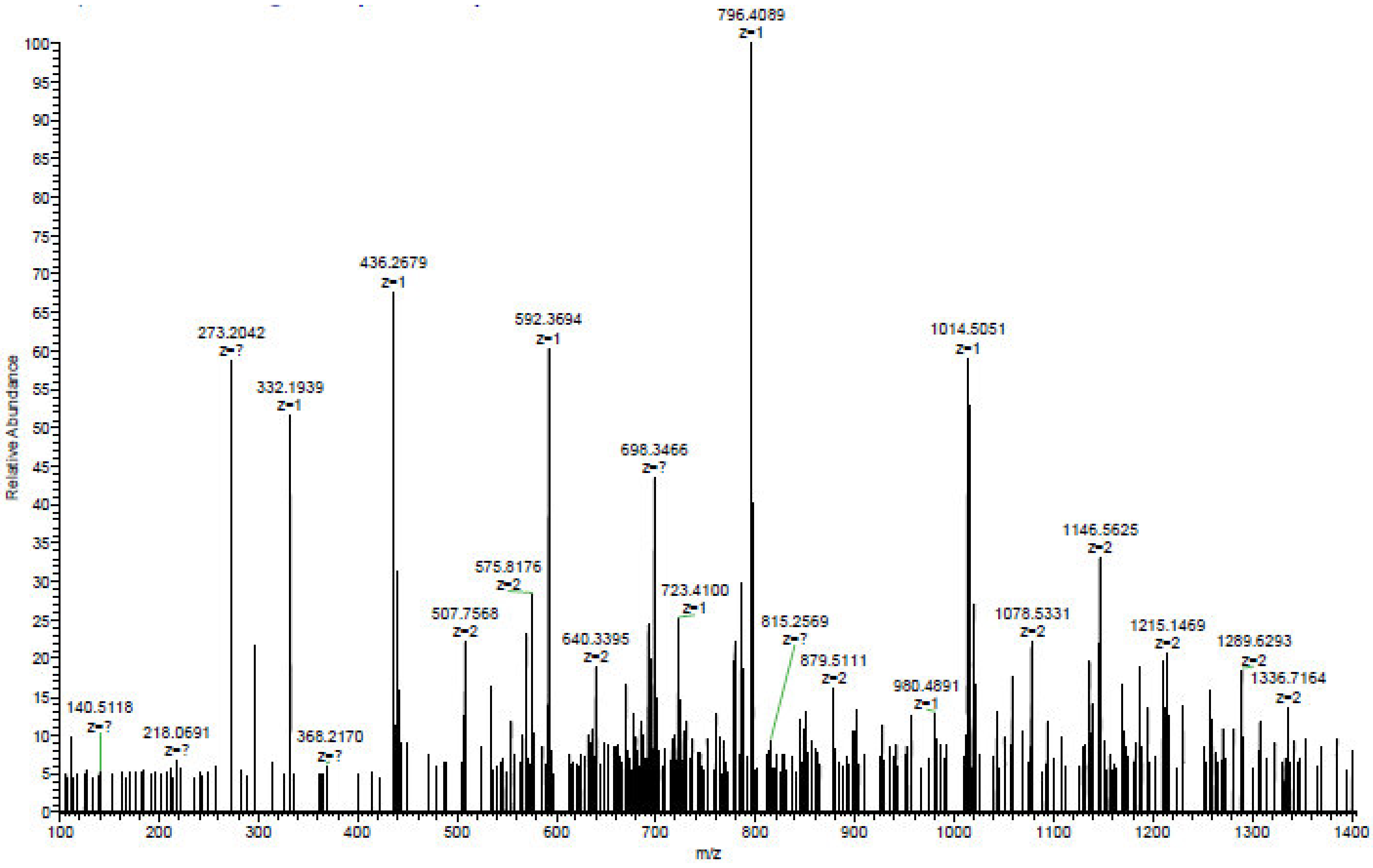

PRM2 (44 – 106)

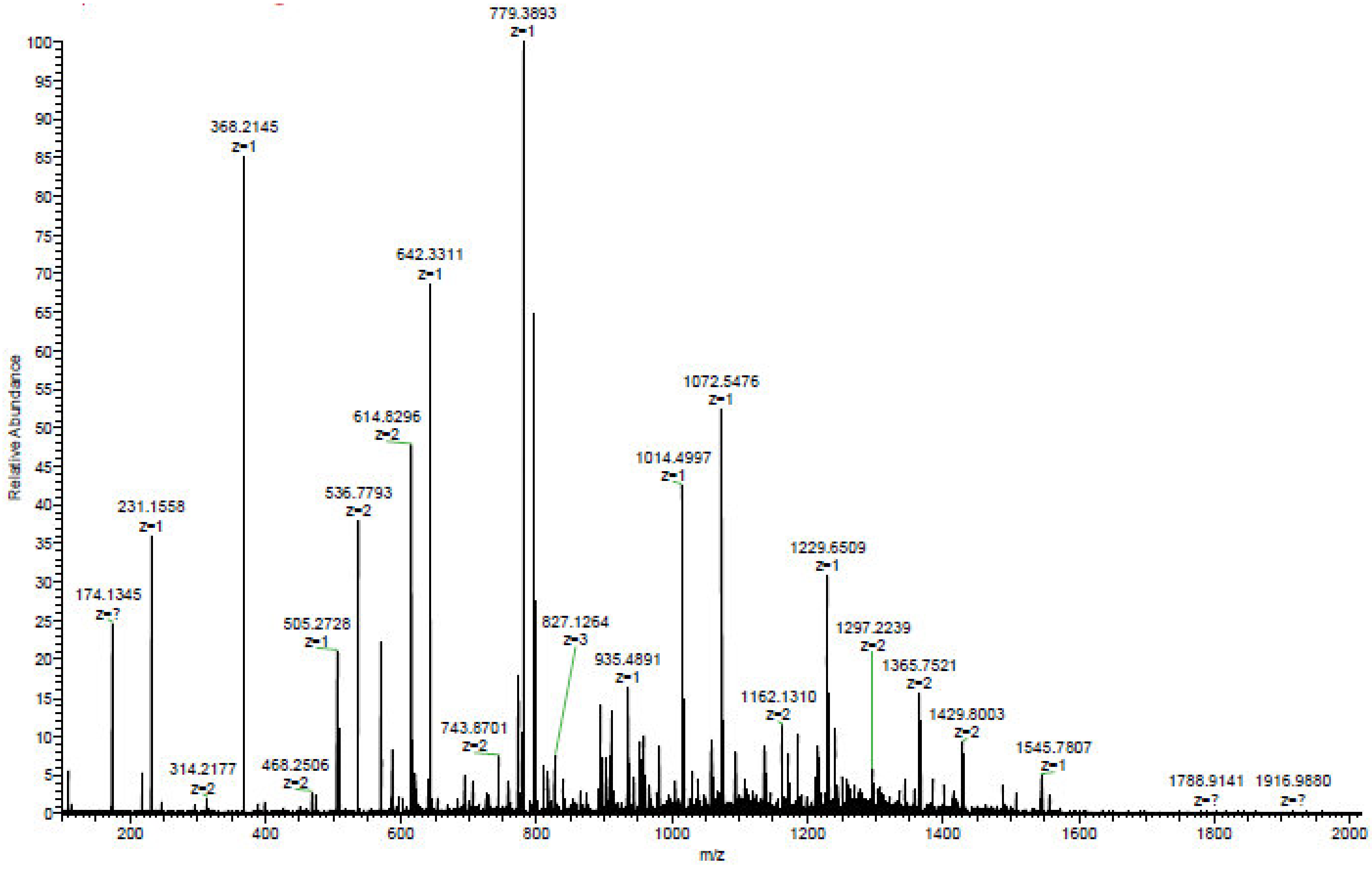

779.3893
z=1
368.2145
z=1
642.3311
z=1
1072.5476
z=1
614.8296
z=2
1014.4997
z=1
536.7793
z=2
231.1558
z=1
1229.6509
z=1
174.1345
z=?
505.2728
z=1
827.1264
z=3
1297.2239
z=2
935.4891
z=1
1365.7521
z=2
1162.1310
z=2
1429.8003
z=2
743.8701
z=2
1545.7807
z=1
314.2177
z=2
468.2506
z=2
1788.9141
z=?
1916.9880
z=?
Relative Abundance
m/z

PRM2 (44 – 106)

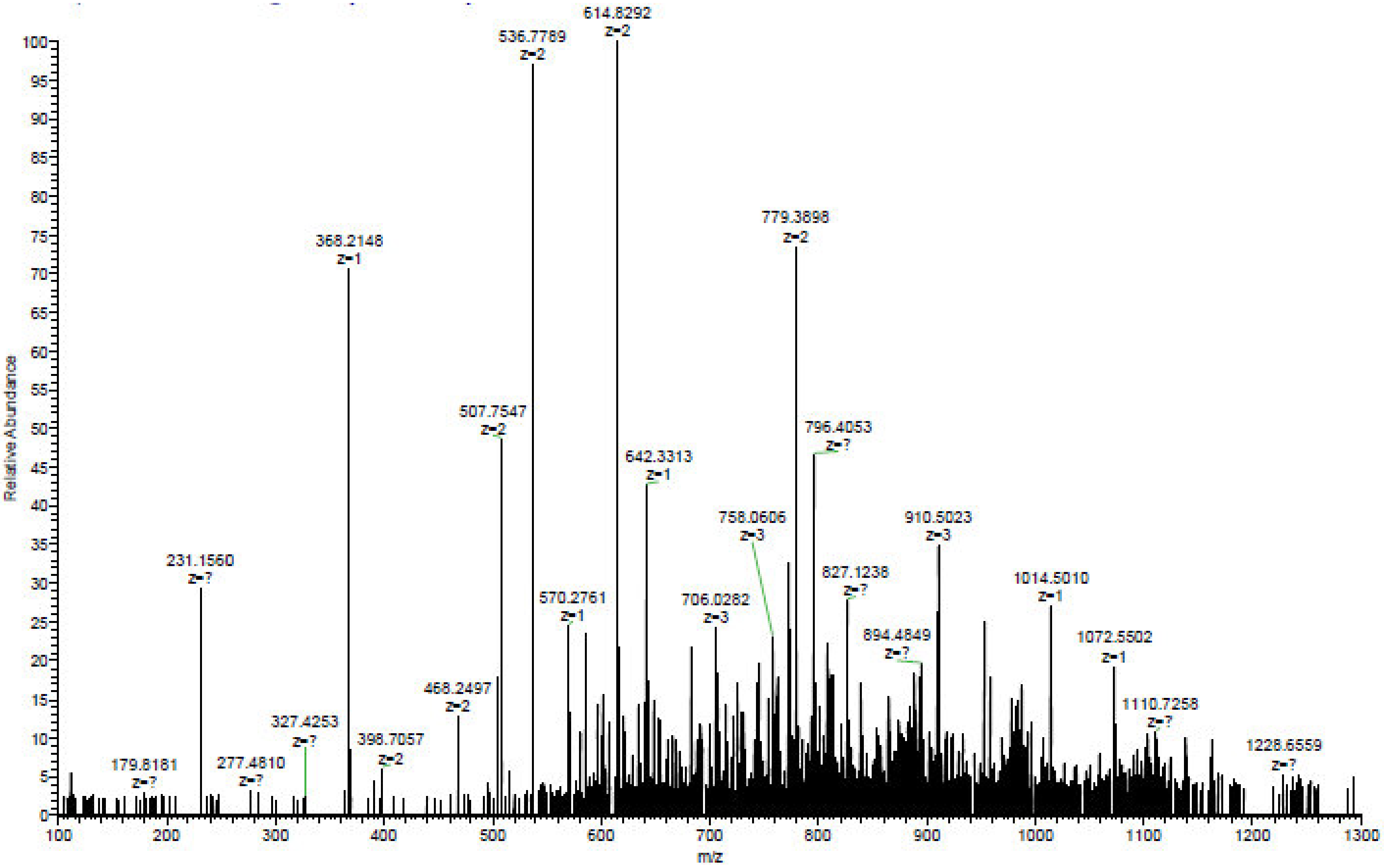

614.8292 z=2
536.7789 z=2
368.2148 z=1
779.3898 z=2
507.7547 z=2
796.4053 z=?
642.3313 z=1
758.0606 z=3
910.5023 z=3
231.1560 z=?
827.1238 z=?
1014.5010 z=1
570.2761 z=1
706.0282 z=3
894.4849 z=?
1072.5502 z=1
468.2497 z=2
1110.7258 z=?
327.4253 z=?
398.7057 z=2
1228.6559 z=?
179.8181 z=?
277.4810 z=?
Relative Abundance
m/z

PRM2 (44 – 106) S55p

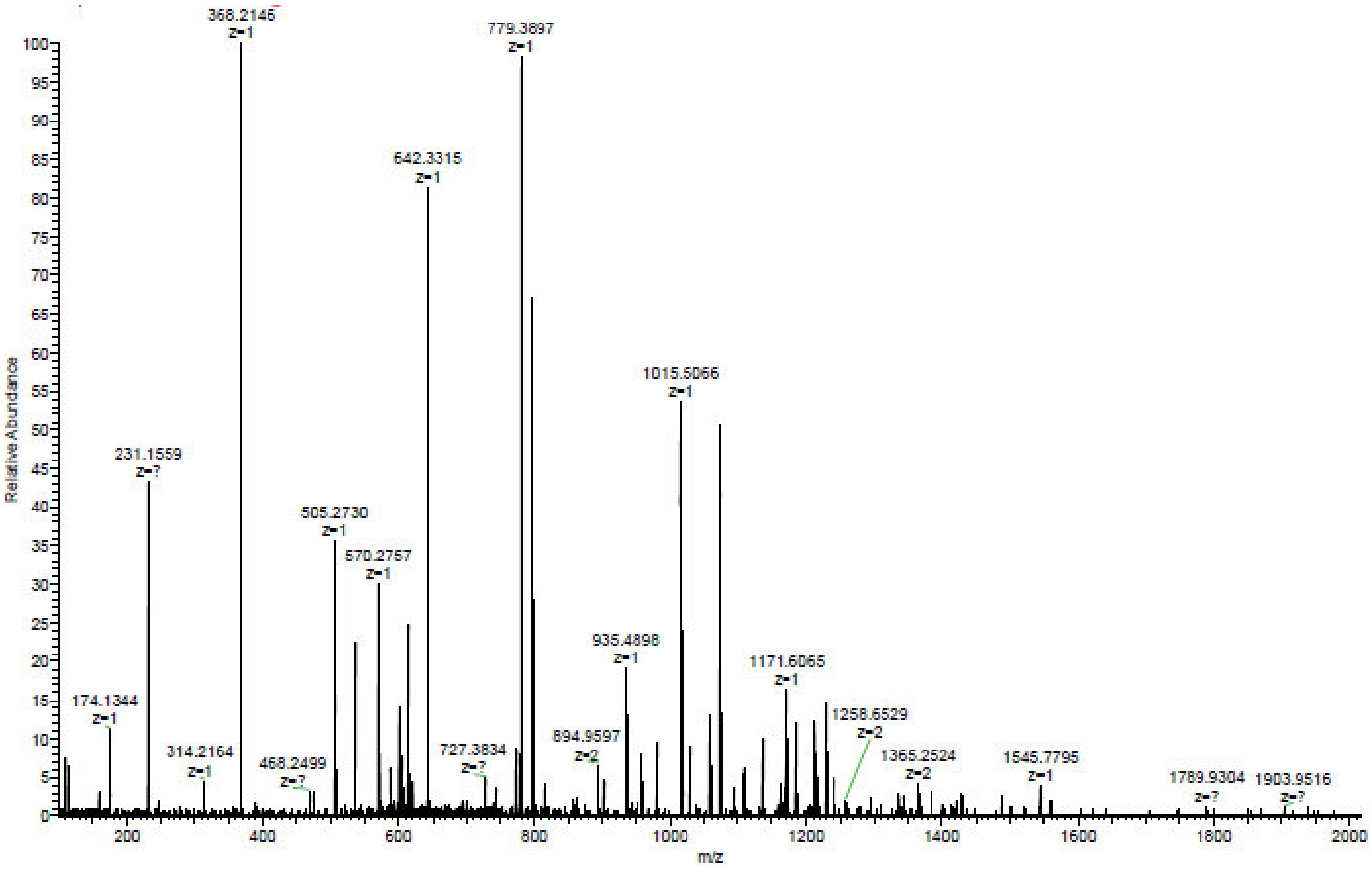

PRM2 (44 – 106) S55p

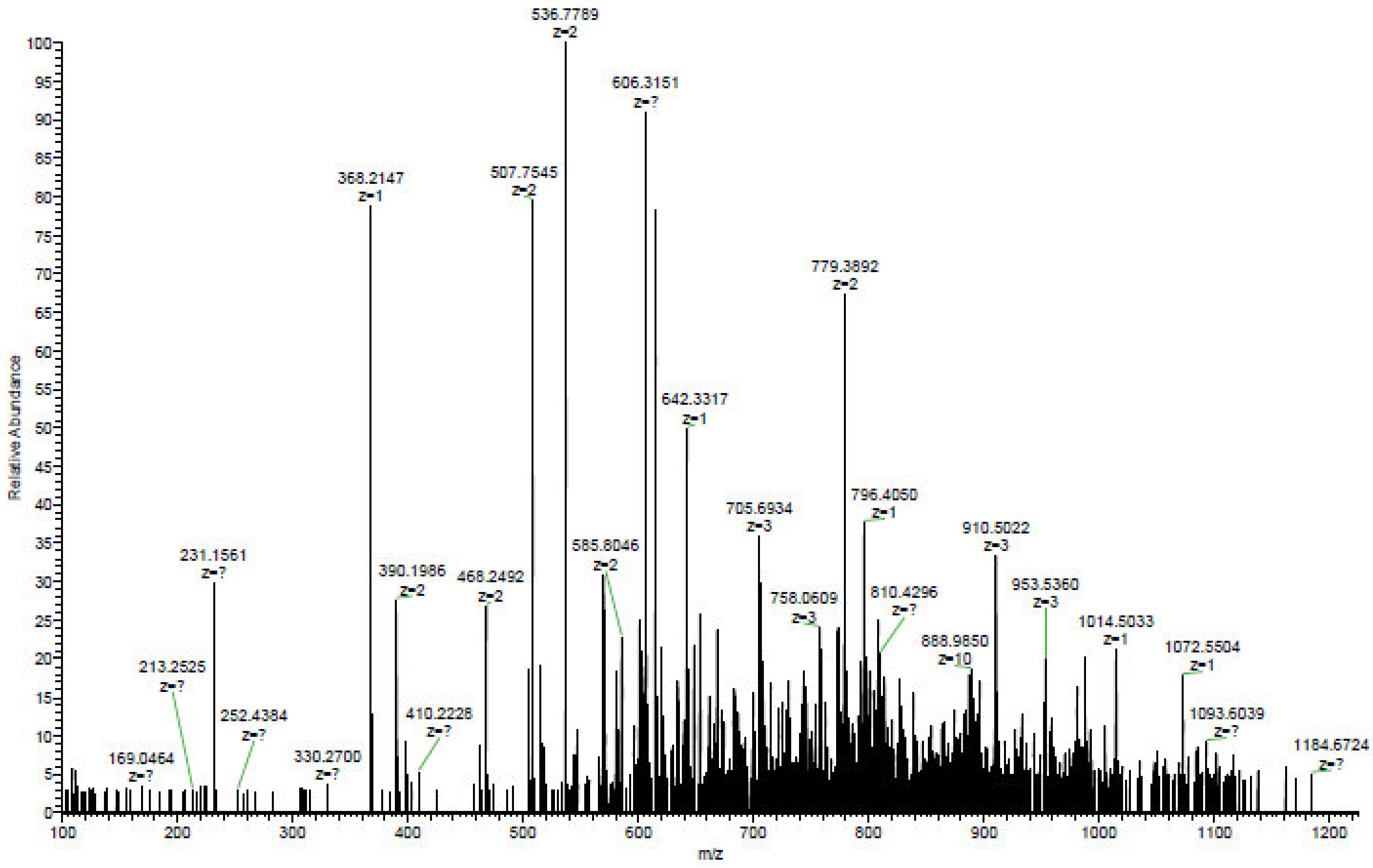

PRM2 (44 – 106) S55ac;
K57ac;

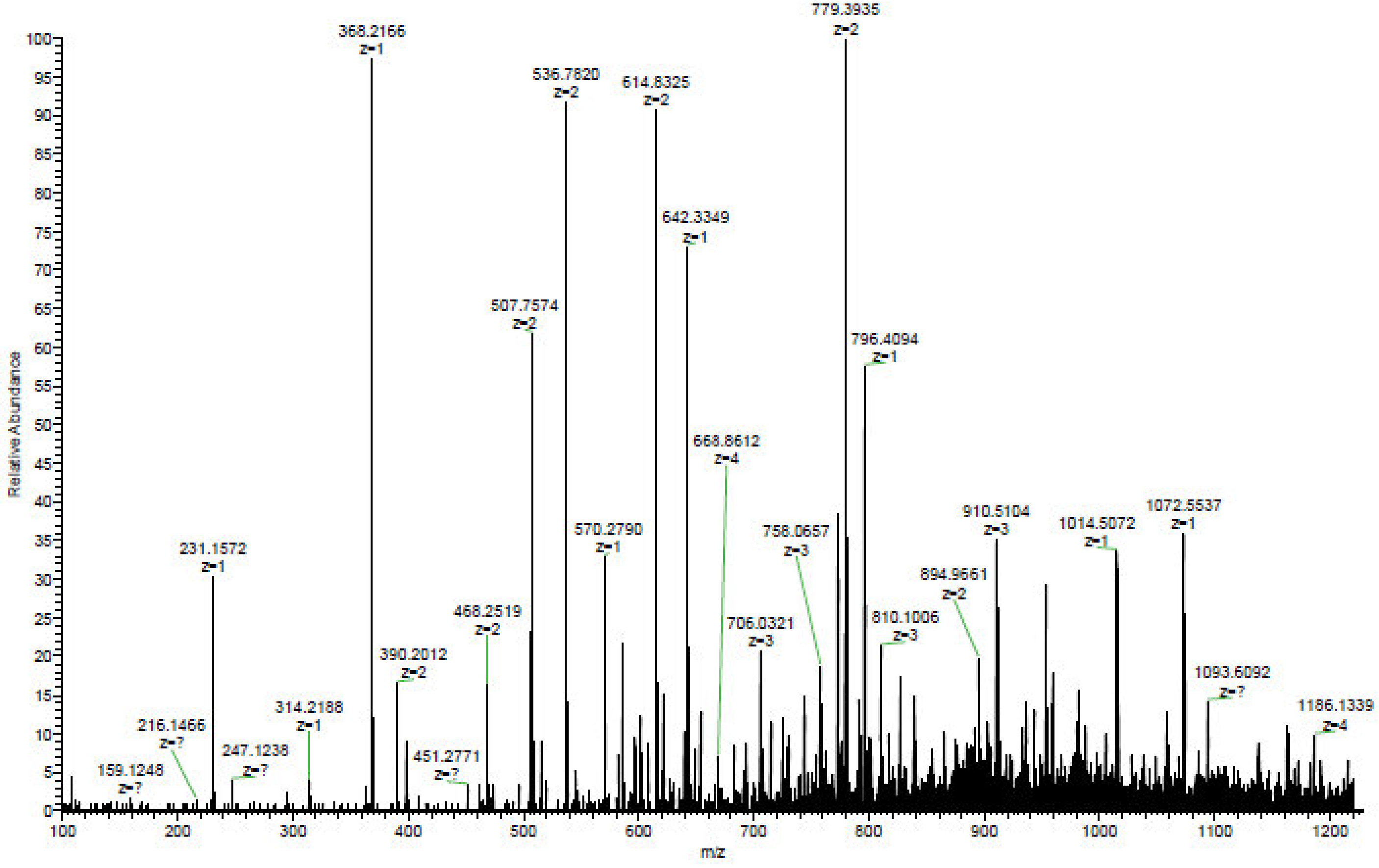

PRM2 (44 – 106) S55ac and K57ac;
S55/K57ac and K64ac;
S55/K57ac and K90ac

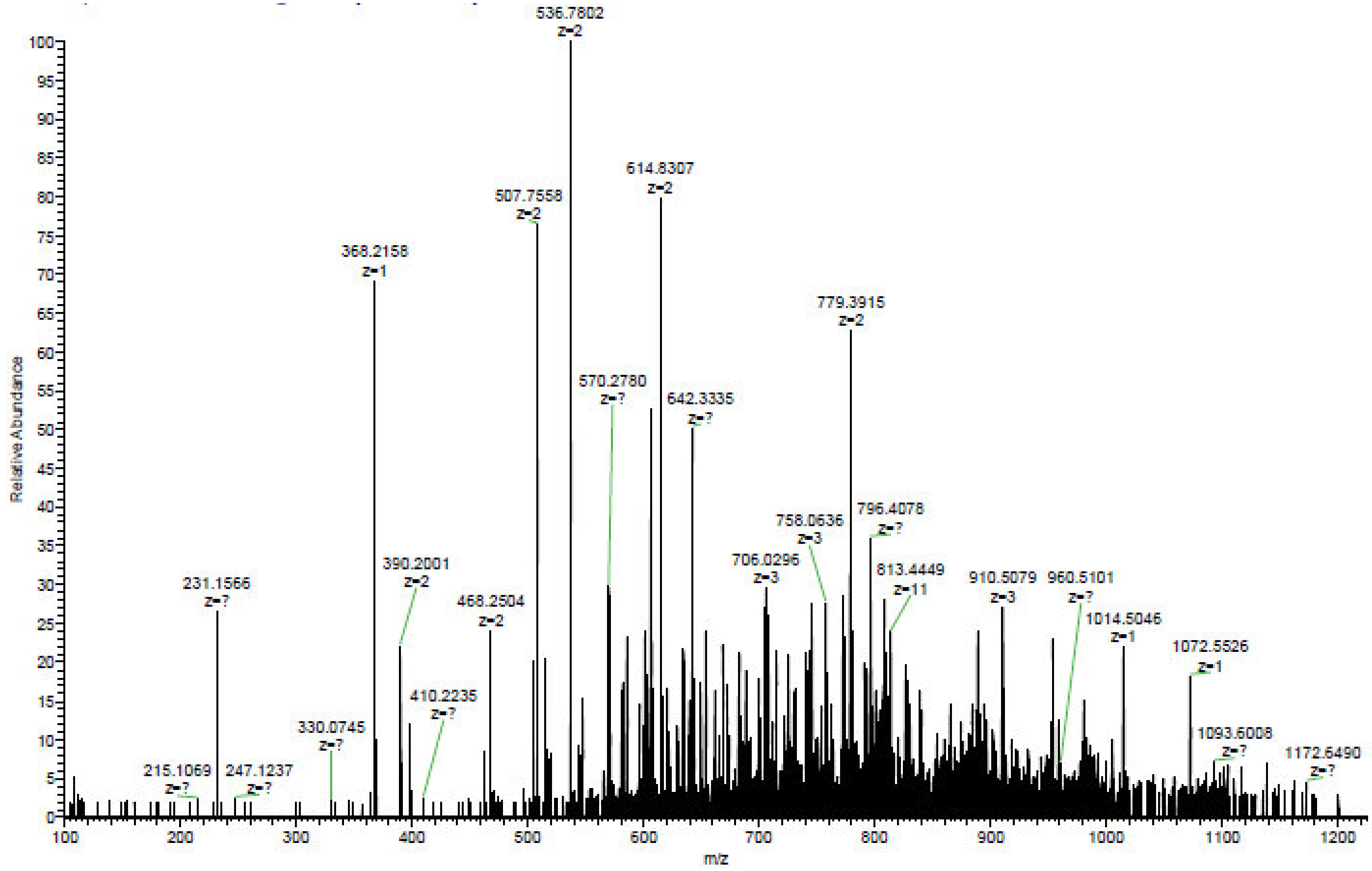

PRM2 (44 – 106) S55ac, S90ac and (R89me1);
K57ac, S90ac and (R89me1);

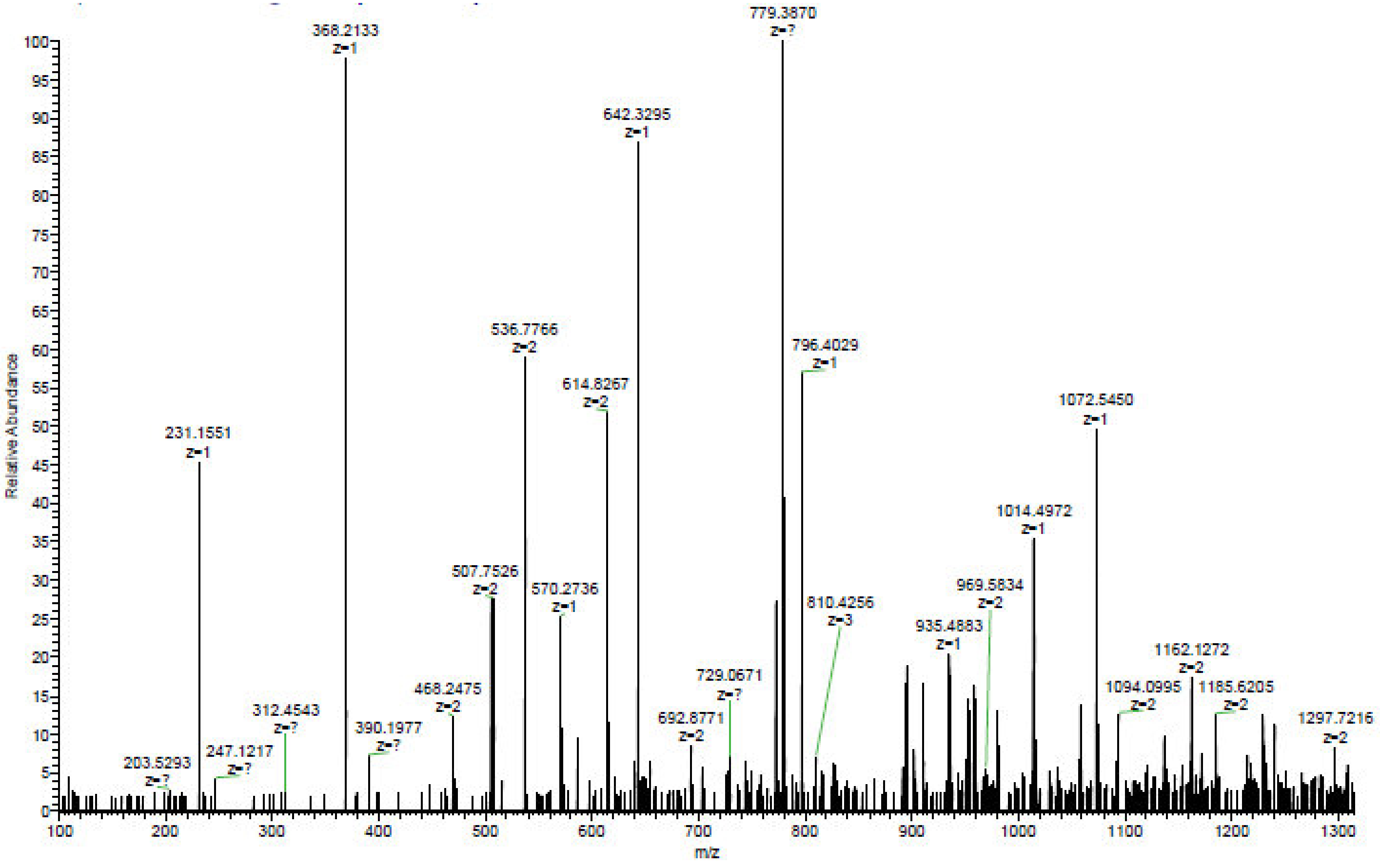

PRM2 (44 – 106) S55ac, S90ac and (R89me1);
K57ac, S90ac and (R89me1);

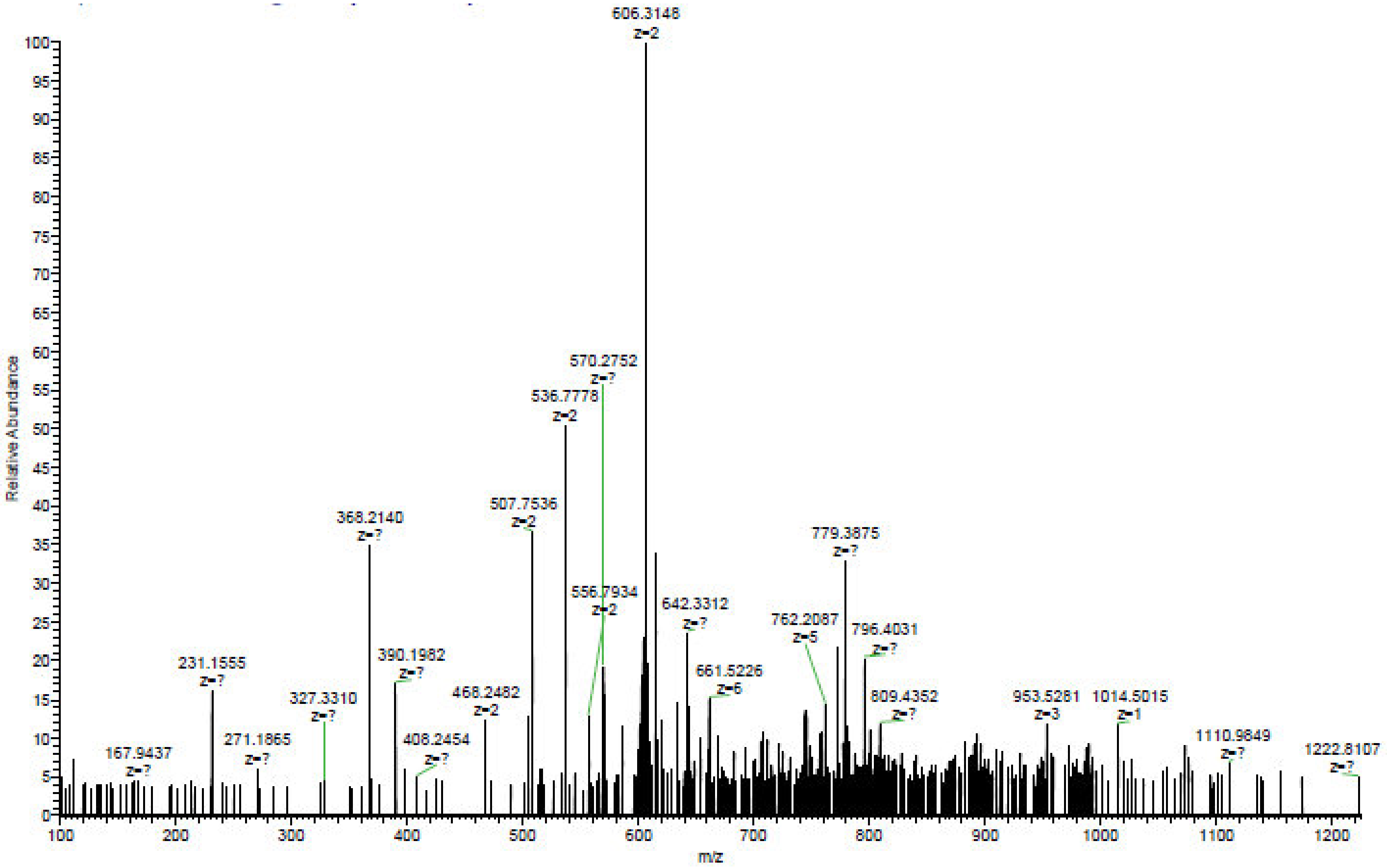

PRM1 (2-51)

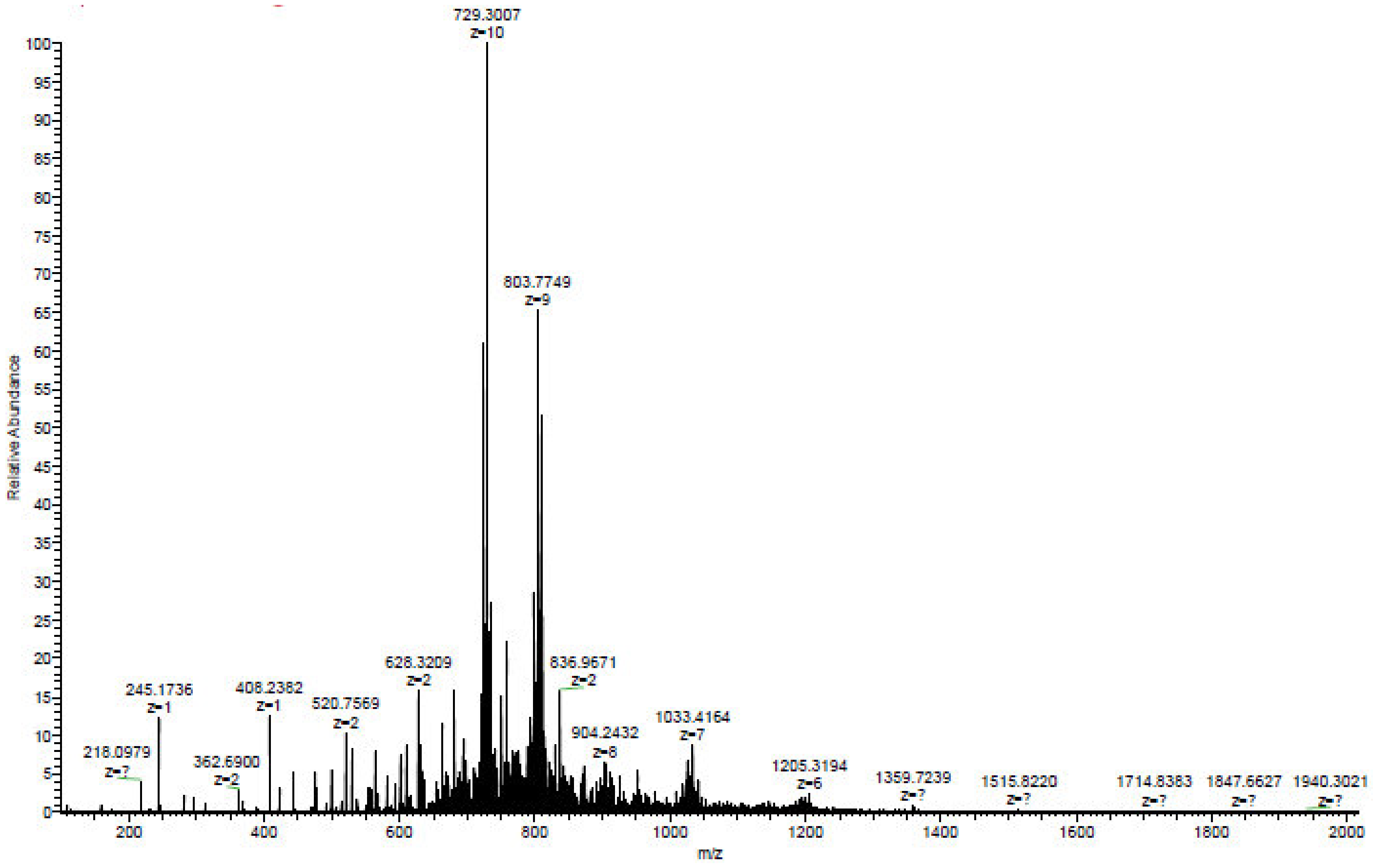

729.3007
z=10
803.7749
z=9
836.9671
z=2
628.3209
z=2
245.1736
z=1
408.2382
z=1
520.7569
z=2
218.0979
z=?
362.6900
z=2
904.2432
z=8
1033.4164
z=7
1205.3194
z=6
1359.7239
z=?
1515.8220
z=?
1714.8383
z=?
1847.6627
z=?
1940.3021
z=?
Relative Abundance
m/z

PRM1 (2- 51)

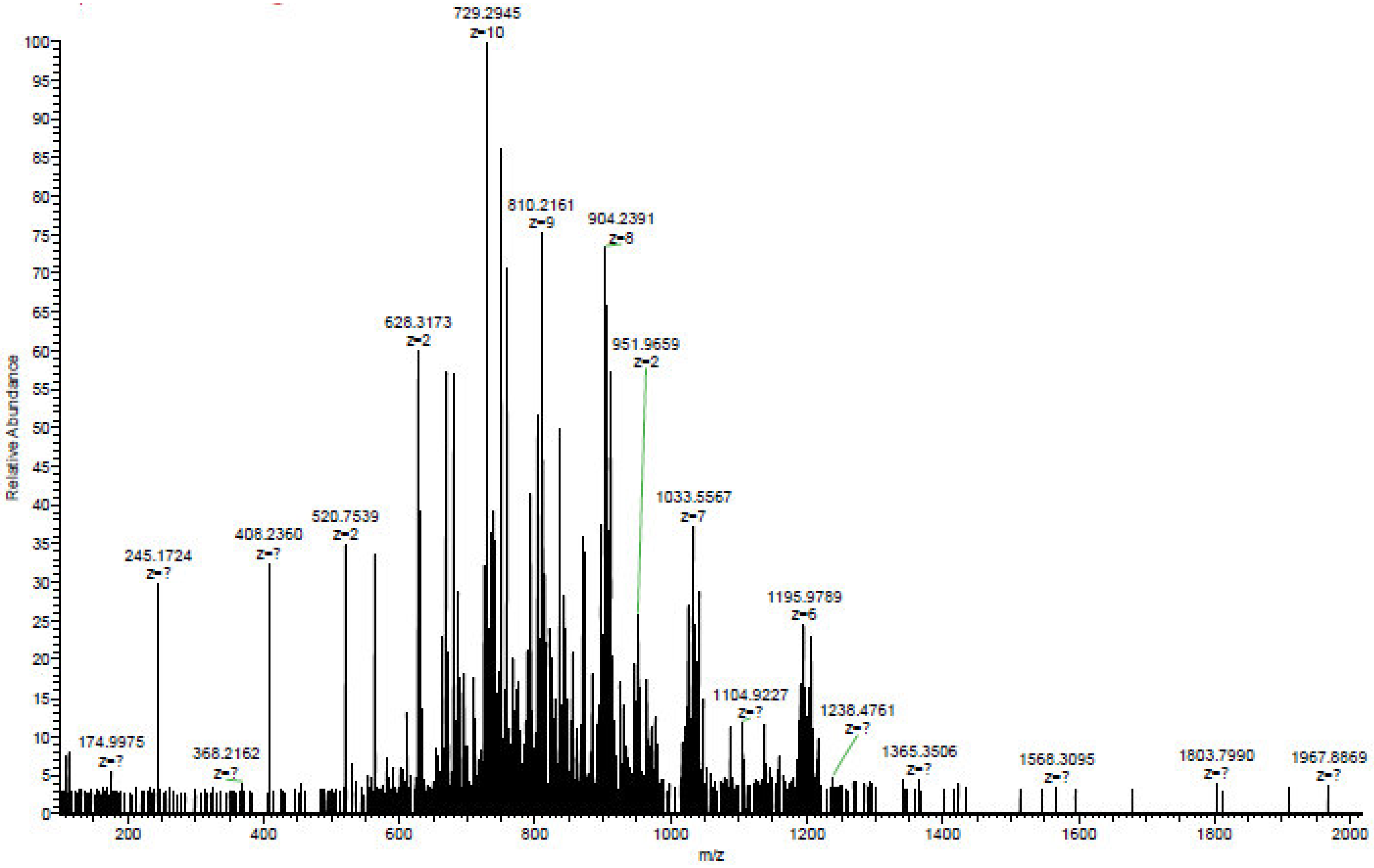
729.2945
z=10
810.2161
z=9
904.2391
z=8
628.3173
z=2
951.9659
z=2
1033.5567
z=7
520.7539
z=2
408.2360
z=?
245.1724
z=?
1195.9789
z=6
1104.9227
z=?
1238.4761
z=?
174.9975
z=?
368.2162
z=?
1365.3506
z=?
1568.3095
z=?
1803.7990
z=?
1967.8869
z=?
Relative Abundance
m/z
